# Transition-Metal-Free *C*-Diarylations to Reach All-Carbon Quaternary Centers

**DOI:** 10.1021/jacsau.4c00500

**Published:** 2024-08-05

**Authors:** Shobhan Mondal, Benjamin Gunschera, Berit Olofsson

**Affiliations:** Department of Organic Chemistry, Arrhenius Laboratory, Stockholm University, 106 91 Stockholm, Sweden

**Keywords:** iodonium salts, carbon nucleophiles, difunctionalization, quaternary center, zwitterionic iodonium compounds

## Abstract

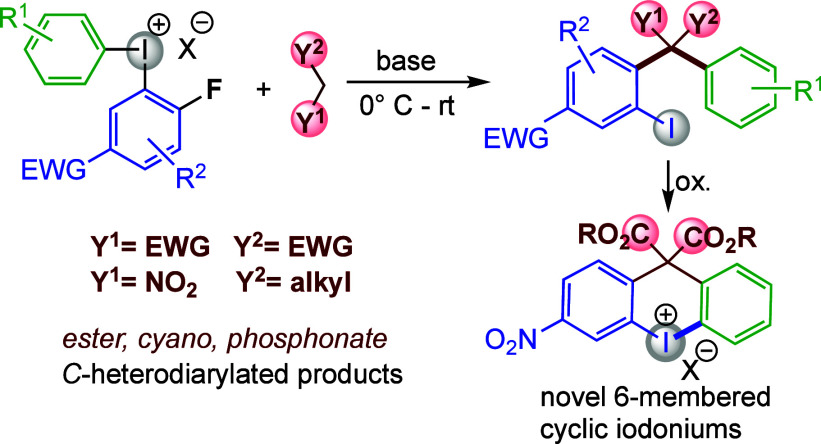

Herein, we disclose a convenient protocol for the α-diarylation of carbon nucleophiles to yield heavily functionalized quaternary products. Diaryliodonium salts are utilized to transfer both aryl groups under transition-metal-free conditions, which enables an atom-efficient and high-yielding method with broad functional group tolerance. The methodology is amenable to a wide variety of carbon nucleophiles and can be utilized in late-stage functionalization of complex arenes. Furthermore, it is compatible with a new class of zwitterionic iodonium reagents, which gives access to phenols with an *ortho*-quaternary center. The diarylated products bear an *ortho*-iodo substituent that can be utilized in further transformations, including the formation of novel, functionalized six-membered cyclic iodonium salts.

The α-arylated carboxylic acid derivatives are a ubiquitous class of compounds that constitutes the backbone for various nonsteroidal anti-inflammatory drugs (NSAIDs), such as ibuprofen and naproxen.^1−3^ Organic structures with an all-carbon quaternary center pose unique importance because of their bioactivities and constraint arrangements.^4,5^ α-Arylation of carbon nucleophiles to generate quaternary carbon centers and enrich molecular complexity has attracted significant attention, furnishing a vast range of arylated organic molecules with medicinal importance, such as methadone, isopropamide, and diphenoxylate (Scheme 1 A).^6−11^

**Scheme 1:**
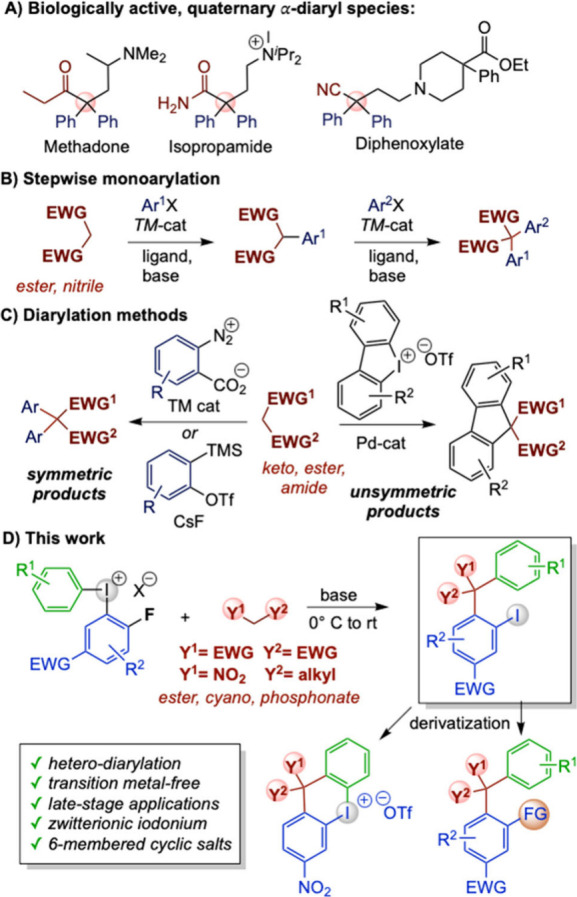
Diarylation of Carbon Nucleophiles

The primary challenges for the synthesis of quaternary carbon centers are steric congestion and conformational restrictions.^12^ Quaternary α-monoarylated centers can be obtained from β-dicarbonyl compounds and nitroalkanes via transition metal catalysis,^13−16^ and metal-free strategies include S_N_Ar, aryne chemistry, and sulfoxide-mediated arylation.^12,17−19^ Diaryliodonium salts, which are mild electrophilic arylating reagents,^20−23^ have also proved efficient in metal-free α-arylation of β-keto esters, malonates, and nitroalkanes.^24−31^

While α-monoarylation of carbonyl compounds is well investigated, methods to synthesize α-diarylated quaternary centers are more scarce and generally require stepwise, metal-catalyzed monofunctionalization methods (Scheme 1B).^32−34^ Symmetric diarylation has only been achieved through the Pd-catalyzed diarylation of ethylcyanoacetate,^35^ through aryne reactions with β-dicarbonyl compounds^36,37^ (Scheme 1C), and through α-diarylation of enol ethers or pyrazolinones with diaryliodonium salts.^38,39^ Unsymmetric α-diarylation of carbon nucleophiles has only been reported to yield fluorenes with cyclic diaryliodonium salts under Pd-catalyzed conditions (Scheme 1C).^40,41^

Our group previously reported a novel strategy to enable one-pot, atom-efficient diarylation of heteroatom nucleophiles under transition-metal-free conditions.^42−44^ The method employs a special type of diaryliodonium salt to unlock S_N_Ar reactivity, and the approach proved successful in the synthesis of a wide variety of unsymmetric diaryl amines, ethers, and sulfides with the *ortho*-iodo substituent retained in the products. Herein, we describe the extension of this strategy to carbon nucleophiles to allow for straightforward synthesis of targets bearing an all-carbon quaternary center decorated with two different aryl groups (Scheme 1D). The retained *ortho*-iodo substituent can be used for further transformations, including one-pot synthesis of six-membered cyclic diaryliodonium salts, which form valuable doubly functionalized diaryl motifs upon reaction with various nucleophiles.^45−47^ The current methodology for synthesis of six-membered cyclic salts requires multistep routes or functionalized starting materials, which limits the availability of such reagents.^45,46^

The investigations were focused on the use of stabilized carbon nucleophiles because too basic nucleophiles can result in byproducts via aryne formation.^48^ The reaction of diaryliodonium salt **1a** and diethyl malonate (**2a**) was first evaluated with various bases and solvents at 100 °C, which resulted in the formation of desired product **3a** in low yield, along with a diaryl ether byproduct **3a′**.^49^ To our delight, the diaryl ether formation was completely suppressed when the enolate was preformed from **2a** and added dropwise to a solution of **1a** at room temperature (Table 1, entry 1). A base screen showed that sodium hydride enabled almost quantitative yield of **3a** (entries 2–4), whereas other solvents proved to be less efficient (entries 5 and 6). In the interest of atom efficiency, the equivalents of **2a** were reduced but this resulted in decreased yield (entry 7).^49^ Interestingly, the reactivity was improved when pentane-washed sodium hydride was used (entries 8 and 9), and product **3a** could be isolated in 87% yield using only a slight excess of **2a** when the reaction was performed at 0 °C to rt (entry 10). For sustainability reasons, these conditions were generally employed in the scope studies, while the results using the conditions in entry 4 are reported for selected products.

**Table 1:**
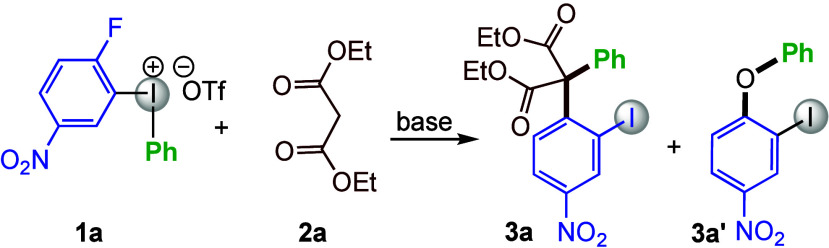
Optimization of the Reaction Conditions^a^

entry	**2a** (equiv)	base (equiv)^b^	solvent	*T* (°C)	yield **3a** (%)^c^
1	2.0	NaO^*t*^Bu (2)	DMF	rt	89
2	2.0	KO^*t*^Bu (2)	DMF	rt	67
3	2.0	NaOTMS (2)	DMF	rt	89
4	2.0	NaH (2)	DMF	rt	99
5	2.0	NaH (2)	THF	rt	27
6	2.0	NaH (2)	Et_2_O	rt	29
7	1.1	NaH (2)	DMF	rt	71
8	1.0	NaH_w_ (2)	DMF	rt	78
9	1.0	NaH_w_ (2)	DMA	rt	79
10^d^	1.3	NaH_w_ (2.4)	DMA	0 °C to rt	93 (87)

a Reaction conditions: **2a** and base were prestirred before adding to **1a** (0.025 mmol) in solvent (0.03–0.06 M) for 12–14 h reaction time.

b NaH = sodium hydride 60% in mineral oil; NaH_w_ = sodium hydride washed with pentane.

c ^1^H NMR yield using 1,3,5-trimethoxybenzene (TMB) as internal standard (isolated yield in parentheses).

d Reaction scale at 0.2 mmol. DMF = *N*,*N*-dimethylformamide; DMA = *N*,*N*-dimethylacetamide.

The generality of the methodology was evaluated by variation of diaryliodonium salts **1** (Scheme 2A). To our delight, **3a** was isolated in 90% yield when the reaction was performed at gram scale. Alkyl-substituted **1** furnished diaryl malonates **3b**–**3d** in high yields and iodonium salts with halide substituents exhibited excellent reactivity to provide diaryl malonates **3e**–**3g** in excellent yields. This feature makes the methodology complementary to metal-catalyzed cross couplings, which have scope limitations in the synthesis of halide-substituted products^15^ or require the use of super-stoichiometric Cu salts^33,50,51^ or unstable diazo esters.^52^

**Scheme 2:**
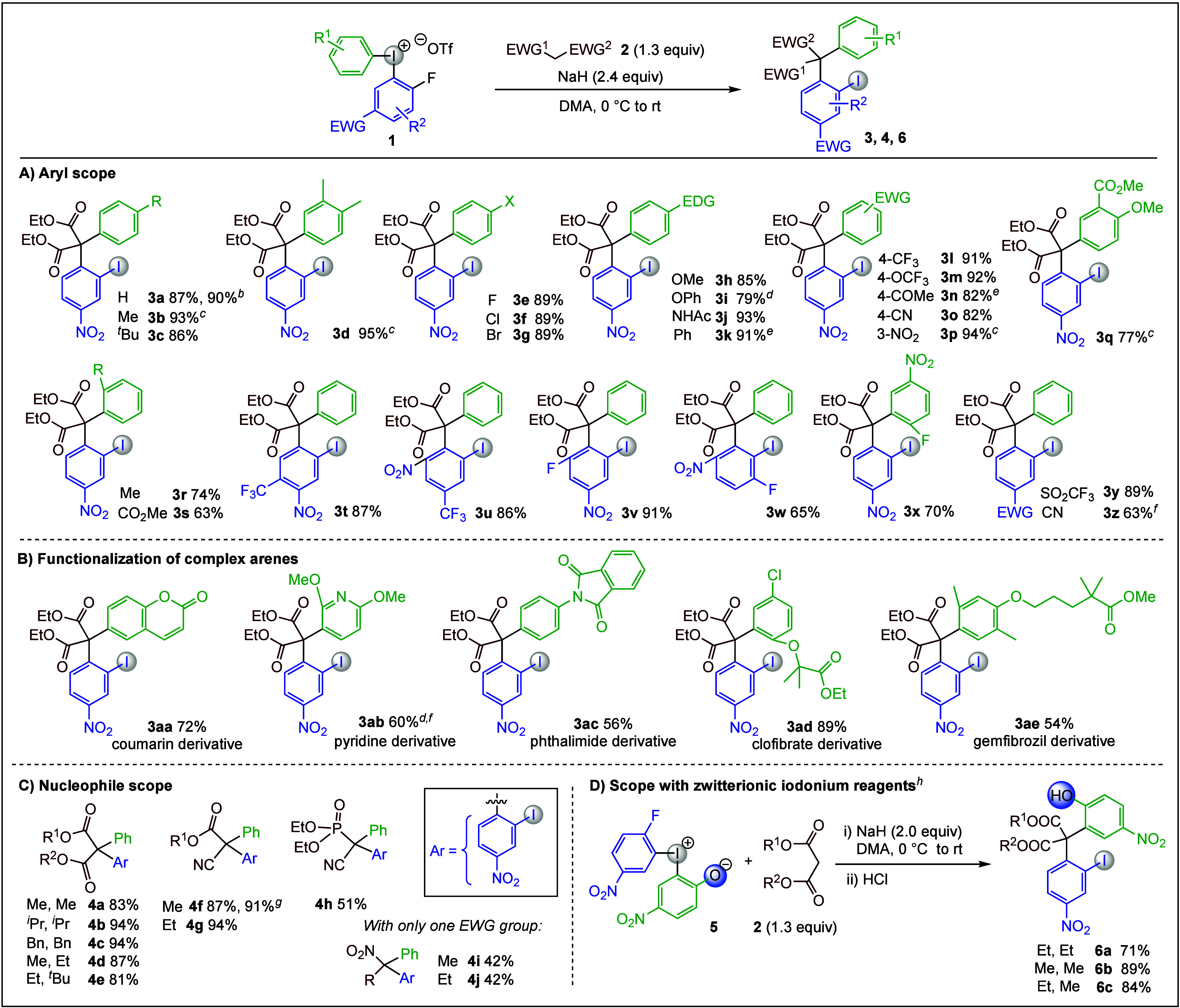
Diarylation Scope Reaction conditions: **1** (0.20 mmol), **2a** (0.26 mmol), NaH (washed, 0.48 mmol), and DMA (0.06–0.07 M) under N_2_ atmosphere at 0 °C were stirred for 2–4 h at 0 °C and 12–14 h at rt. Gram-scale synthesis. Conditions from entry 4 used: **1** (0.20 mmol), **2a** (0.40 mmol), NaH (unwashed, 0.40 mmol), DMF (0.6–0.07 M). OTs^–^ salt was used. BF_4_^–^ salt was used. Compound **2a** (0.40 mmol) was used. Reaction scale of 1.5 mmol. Reaction conditions as above followed by acidic workup with HCl (1 M).

Electronic effects in **1** were next evaluated, and the electron-donating groups (EDG) OMe, OPh, NHAc, and Ph were well tolerated to produce **3h**–**3k**. The use of electron-withdrawing groups (EWG), like CF_3_, OCF_3_, COMe, CN, and NO_2_, also led to the desired malonates **3l**–**3p** in high to excellent yields, and the combination of EDG and EWG gave decorated malonate derivative **3q**. To our delight, *ortho*-substituted salt **1** was productive in the diarylation and provided malonate **3r** with little influence on the yield compared to the corresponding *para*-substituted salt (see **3b** vs **3r**). Additionally, an *ortho*-ester substituent was tolerated and gave the product **3s**.

The scope of possible substituents in the electron-deficient aryl ring was investigated by adding further functional groups. Insertion of a CF_3_ group resulted in a smooth reaction to provide malonate derivative **3t** in excellent yield. Likewise, the NO_2_ group could be placed *ortho* to the fluoride with CF_3_ in the *para*-position (**3u**). Reactions with salts **1** decorated with two fluorides were employed to evaluate the regioselectivity of the S_N_Ar step (**3v**–**3x**). Importantly, complete regioselectivity was observed in the formation of products **3v** and **3w**. A highly electron-deficient, symmetrical iodonium salt could be used to provide malonate derivative **3x**, thereby illustrating that the internal aryl transfer is preferred over a second S_N_Ar reaction and that another S_N_Ar on the product does not take place under the reaction conditions. The S_N_Ar reactivity was also enabled by other strong EWG groups, as demonstrated by products **3y** and **3z**.

The broad application possibilities and wide functional group tolerance of the methodology was illustrated by the use of more complex arenes. Heterocycles, bioactive compounds and drug molecules were first converted with complete regioselectivity to the corresponding diaryliodonium salts using established one-pot methods.^53−56^ Subsequent diarylation yielded complex heterodiarylated products with an all-carbon quaternary center (Scheme 2B). In this fashion, a coumarin-derived iodonium salt underwent diarylation to give target product **3aa** in a good yield. An electron-rich pyridine moiety and a phthalimide derivative were also tolerated to furnish **3ab** and **3ac**. Furthermore, late-stage functionalization of the drug molecules clofibrate and gemfibrozil provided the corresponding diaryl malonates **3ad** and **3ae**. To the best of our knowledge, these applications are the first examples where complex heterodiarylated products are formed by a regioselective insertion of an active methylene onto arene scaffolds with further functionalization opportunities (*vide infra*).

The scope of activated methylene compounds was subsequently explored (Scheme 2C). Common symmetric malonates, such as dimethyl, diisopropyl, and dibenzyl reacted smoothly to give the desired products **4a**–**4c** in excellent yields. Similar reactivities were observed with unsymmetric malonates, which furnished their diarylated derivatives **4d** and **4e**. α-Cyano esters were found to be compatible carbon nucleophiles under the same reaction conditions, which provided their derivatives **4f** and **4g** in excellent yields. Additionally, the synthesis of **4f** was scaled up to 1.5 mmol, which resulted in a slightly elevated yield. Because of the strong precedence of phosphonate esters as nucleophiles,^57,58^ a cyano-substituted phosphonate ester was tested and delivered diarylated species **4h** in moderate yield. While monoarylation of nitro-alkanes is well investigated,^59,60^ α-diarylation methods have not been explored. Hence, the successful diarylation of nitroalkanes to provide quaternary nitroalkanes **4i** and **4j** constitutes a new route to such targets.

Aryliodonium ylides are special types of iodine(III) reagents that are generally formed from dicarbonyl compounds. Such reagents have been used in *C*-functionalizations via, e.g., fluorination, trifluoromethylation, cycloaddition reactions, and as carbene precursors in metal-catalyzed reactions.^61−64^ Zwitterionic iodonium compounds with a phenoxy moiety are a related compound class that can undergo intramolecular aryl transfer to form diaryl ethers.^65−67^ The utility of such reagents in diarylations to transfer both aryls of the reagent remains unexplored. To our delight, reactions with zwitterionic reagent **5** smoothly delivered the corresponding diarylated malonates **6a**–**6c** decorated with two different handles for further functionalization (Scheme 2D).

We have previously studied the mechanism in diarylation of heteroatom nucleophiles and supported the proposed S_N_Ar pathway by isolation of the diaryliodonium intermediate, as well as through DFT studies.^42−44^ Moreover, β-dicarbonyls are well known nucleophiles for S_N_Ar reactions under basic reaction conditions.^17,68^ Based on those results and NMR studies of the current system,^49^ we propose that the reaction proceeds by facile deprotonation of the malonate to give enolate **I**, which might coordinate to the iodine or directly undergo S_N_Ar reaction to give α-arylated malonate species **II** (Scheme 3). This is quickly deprotonated by the remaining base to give intermediate **III**, which was observed during low-temperature NMR studies and could also be trapped by HCl in diethyl ether to give **II**.^49^ Finally, intramolecular transfer of the phenyl group to the enolate carbon with concomitant breaking of the C–I bond leads to product **3a.**

**Scheme 3:**
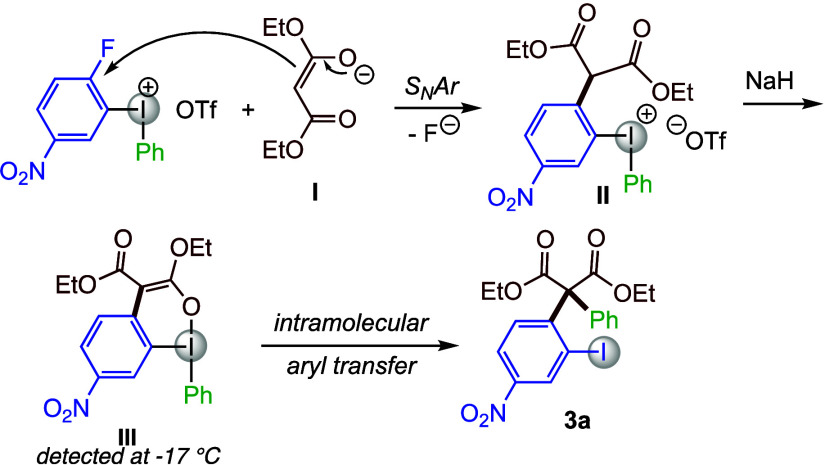
Proposed Mechanism

To evaluate the ease of postsynthetic transformations, products **3a** and **4a** were derivatized through a range of reactions (Scheme 4). Transition-metal-free reduction of **3a** gave the corresponding amine derivative **7a** in excellent yield. Decarboxylation proceeded smoothly to provide diarylated ester **7b**, thereby highlighting the ease of obtaining α-diarylated products from enolates that were unsuitable nucleophiles in the diarylation.^49^

**Scheme 4:**
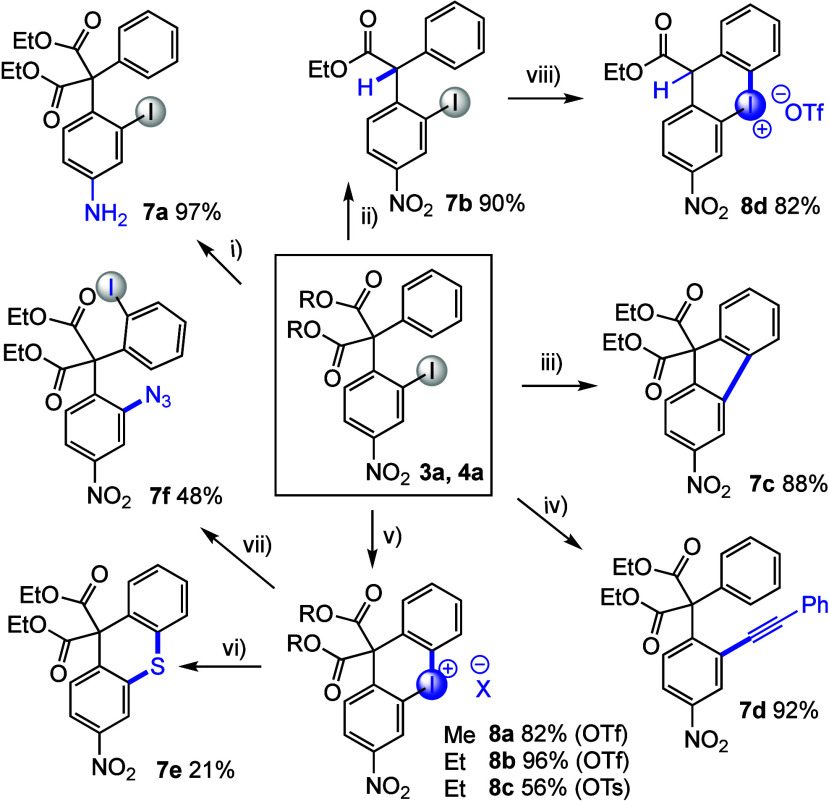
Postsynthetic Applications of Malonate **3a** Reaction conditions: (i) B_2_(OH)_4_, 2,2′-bipyridine, DMF, 40 °C, 4 h; (ii) LiCN, KCN, H_2_O, DMSO, 100 °C, 3 h; (iii) Pd(OAc)_2_, PCy_3_, K_2_CO_3_, PivOH, 120 °C, 12 h; (iv) Pd(PPh_3_)_4_, CuI, NEt_3_, 100 °C, 3 h; (v) *m*CPBA (*meta*-chloroperoxybenzoic acid), TfOH or TsOH, CH_2_Cl_2_, rt, 12 h; (vi) BnNHCS_2_NHEt_3_, CuSO_4_, 2,2-bipyridine, MeCN, 50 °C, 3 h (from **8b**); (vii) NaN_3_, DMA, 120 °C, 30 min (from **8b**); (viii) *m*CPBA, TfOH, CH_2_Cl_2_, rt, 2 h.

Next, derivatizations using the iodine handle were evaluated, and a Pd-catalyzed cyclization delivered fluorene derivative **7c**, whereas a Sonogaschira coupling produced **7d** in excellent yield. The *ortho*-iodo motif was also utilized in the synthesis of novel six-membered cyclic iodonium salts **8a**–**8c** under our standard reaction conditions for iodonium salt synthesis.^42^ Preliminary evaluation of the reactivity of **8b** revealed that Cu-catalyzed insertion of a sulfur bridge to give **7e** and metal-free azidation to give *ortho*-difunctionalized product **7f** were feasible. Furthermore, cyclic iodonium triflate **8d** was easily synthesized from monoester product **7b**.

In conclusion, we have developed an efficient protocol for the *C*-diarylation of carbon nucleophiles under mild, transition metal-free conditions. Using this procedure, unsymmetrically diarylated malonates, nitroalkanes, cyano-substituted esters, and phosphonate esters were attainable. Both diaryliodonium salts and novel zwitterionic iodonium compounds were utilized in the transformation. Furthermore, a variety of functional groups were well tolerated on both the iodonium salts and the carbon nucleophiles. The various functionalities present on the diaryated products allow diverse downfield diversification and even the formation of a novel class of six-membered iodonium salts, which will be further explored. We anticipate that this efficient *C*-diarylation methodology will become a versatile tool for mild and metal-free synthesis of complex molecules containing quaternary centers.

## Supporting Information Available

Experimental procedures, mechanistic studies, and spectral data (PDF)
