# Supplementary material for: Transition-Metal-Free C-Diarylations to Reach All-Carbon Quaternary Centers
Source: JACS Au. 2024 Aug 5;4(8):2832–7. doi: 10.1021/jacsau.4c00500 (PMC11350576; doi:10.1021/jacsau.4c00500)

Supporting Information

Transition Metal-Free *C*-Diarylations to reach All-Carbon Quaternary Centers

Shobhan Mondal, Benjamin Gunschera, and Berit Olofsson*

Department of Organic Chemistry
Arrhenius Laboratory, Stockholm University
106 91 Stockholm (Sweden)

Email: berit.olofsson@su.se

Table of Contents

1 General Information 3

2 Optimization studies 4

2.1 Optimization at high temperature 4

2.2 Optimization with preformed malonate solution 5

2.3 Optimization with portion-wise addition of base 6

2.4 Optimization performed with pentane-washed NaH 7

2.5 Optimization with different iodonium salts 7

3 Synthesis of diaryl malonates 3 10

3.1 Synthetic details and analytical data 12

3.2 Large scale synthesis 26

4 Mechanistic investigations 28

4.1 Mechanistic hypothesis 28

4.2 Investigation of the S_NAr in pathway 2 29

4.3 Characterization and trapping of intermediates 32

4.4 Intramolecular aryl transfer 37

5 Post-Synthetic transformations 39

5.1 Reductions and cyclizations 39

5.2 Synthesis and application of cyclic salts 41

6 Synthesis of starting materials 45

6.1 Synthesis of diaryliodonium salts 1 45

6.2 Synthesis of zwitterionic iodonium reagent 5a: 52

6.3 Synthesis of other iodine(III) reagents 52

7 References 53

8 NMR spectra 54

1 General Information

Unless otherwise stated, all air and moisture sensitive reactions were carried out under N_2 atmosphere in oven-dried microwave tubes. Solvents were obtained as p. a. grade and dried with a VAC solvent purification system and stored over molecular sieves (4 Å) if necessary. *N*,*N*-Dimethylformamide (DMF), dichloromethane, and tetrahydrofuran (THF) were collected from the solvent purification system and stored over molecular sieves. Dimethylacetamide (DMA) was purchased from TCI Europe and stored over molecular sieves under nitrogen atmosphere.

All reagents were prepared following literature procedures and commercially available chemicals were purchased from Aldrich Chemical Co., Alfa Aesar, Fluorochem, TCI Europe and used as received. *m*CPBA (Aldrich, 77% active oxidant) was dried under vacuum and was iodometric titrated to determine the active oxidant.[1] Trifluoromethanesulfonic acid (TfOH) was stored and handled under N_2, and liquid carbon nucleophiles were distilled prior to the use. NaH (60% in paraffin oil) was washed with pentane three times to remove the oil, dried and stored under nitrogen atmosphere (caution! NaH should be handled with proper precautions, should be kept away from moisture). Analytical thin layer chromatography (TLC) was performed on silica gel 60 F_{254} aluminum plates (Merck). TLC plates were visualized by exposure to short-wave ultraviolet light (254 nm). Flash chromatography was performed on VWR silica gel by standard techniques. Melting points were measured using a STUART SMP3 and are reported uncorrected. All NMR spectra were recorded on 400 or 500 MHz Bruker AVANCE II with a BBO probe at 298 K using $CDCl_3$, DMSO-d_6 or CD_3OD as solvents. Chemical shifts are given in ppm relative to the residual solvent peak (^{1}H NMR: $CDCl_3$ δ 7.26; DMSO-d_6 2.50; ^{13}C NMR: $CDCl_3$ δ 77.16; DMSO-d_6 39.52). Shift values are reported in ppm and all coupling constants (*J*) are printed in Hertz (Hz) with their multiplicity: s (singlet), br (broad signal), d (doublet), t (triplet), q (quartet), pent (pentet), m (multiplet), app (apparent). HRMS Mass spectra were measured on a Bruker microTOF with electron spray ionization (ESI). Mass calibration was carried out directly before the measurement of the sample using clusters of sodium formate.

2 Optimization studies

The optimization of the reaction conditions began with conditions based upon previous work in our group and literature reports,[2] and performed using diaryliodonium salt **1a** and malonate **2a**, base and solvent (Scheme S1). Reactions were performed in oven dried microwave vials that were sealed under N_2 atmosphere. Then the reaction mixture was extracted with ethyl acetate and water (for DMF, DMA), and volatalities were removed under reduced pressure. Crude ^{1}H-NMR was measured using 1,3,5-trimethoxybenzene (TMB) as internal standard.

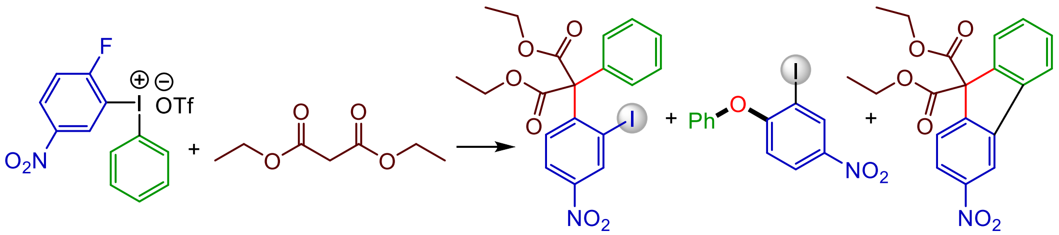

1a **2a** **3a** **3a'** **9a**

Scheme S1: Optimization reaction.

2.1 Optimization at high temperature

After an oven dried microwave vial was charged with **1a**, **2a** and base, the vessel was sealed and connected to the vacuum line and solvent was added under N_2 atmosphere. Following that, the reaction mixture was stirred in a pre-heated oil bath at 100 °C for the tabulated time (Table S1).

Table S1: Base & solvent screening at high temperature[a]

entry	2a (equiv)	base (equiv)	solvent	T (°C)	time (h)	yield of 3a (%)[b]	yield of 3a' (%)[b]	yield of 9a (%)[b]
1	2	^{t}BuONa (2)	DMF	100	15	40	24	7
2	1	K_3PO_4 (2)	DMF	100	15	10	22	8
3	1	K_3PO_4 (2)	EtOAc	100	15	17	14	1
4	1	K_3PO_4 (2)	DCE	100	15	10	5	0
5	1	K_3PO_4 (2)	1,4-dioxane	100	15	0	24	0
6	3	K_3PO_4 (2)	DMF	100	15	9	20	11
7	2	K_3PO_4 (2)	DMF	100	15	14	35	12
8	3	K_3PO_4 (3)	DMF	100	15	6	6	11
9	2	^{t}BuOK (2)	DMF	100	15	10	12	0

[a] Reaction conditions: **1a** (0.025 mmol), base, solvent (0.05 M) were used under N_2 atmosphere. [b] Yields were determined by ^{1}H-NMR analysis using 1,3,5-trimethoxybenzene (TMB) as internal standard.

2.2 Optimization with preformed malonate solution

An oven dried scintillation vial was charged with base, then solvent (0.4 mL) was added, followed by **2a**. The solution was stirred for 30 min at room temperature and was referred to as the "enolate solution". After an oven dried microwave vial was charged with **1a**, the vessel was sealed, connected to the vacuum line and solvent (0.4 mL) was added under N_2 atmosphere. Following that, the enolate solution was added dropwise and reaction mixture was allowed to stir for the tabulated time. Then the reaction mixture was extracted with ethyl acetate and water (for DMF, DMA), and volatiles were removed under reduced pressure. Crude ^{1}H-NMR was measured afterwards using TMB as internal standard.

Table S2: Optimization with pre-formed malonate solution[a]

entry	2a (equiv)	base (equiv)	solvent	T (°C)	time (h)	yield of 3a (%)[b]	yield of 3a' (%)[b]	yield of 9a (%)[b]
1	**2**	^{t}BuOK (2)	DMF	100 °C	15	9	14	0
2	**2**	^{t}BuOK (2)	DMF	rt	15	67	0	0
3	**2**	^{t}BuONa (2)	DMF	rt	15	89	0	0
4	**2**	^{t}BuOLi (2)	DMF	rt	15	68	0	0
5	**2**	NaOEt (2)	DMF	rt	15	0	52	0
6	**2**	NaOTMS (2)	DMF	rt	15	72	0	0
7	**2**	^{t}BuONa (2)	Acetone	rt	15	35	0	0
8	**2**	^{t}BuONa (2)	MeCN	rt	15	36	0	0
9	**2**	^{t}BuONa (2)	Et_2O	rt	15	33	0	0

[a] Reaction conditions: **1a** (0.025 mmol), base (2.0 equiv), solvent (0.033 M) were used under N_2 atmosphere. [b] Yields were determined by ^{1}H-NMR analysis using TMB as internal standard.

Follwing the above mentioned procedure in Section 2.2, the following reactions were carried out and anlyzed with varying equivalences of base and malonate **2a**.

Table S3: Effect of base and malonate stoichiometry[a]

entry	2a (equiv)	base (equiv)	solvent	T (°C)	time (h)	yield of 3a (%)[b]	yield of 3a' (%)[b]	yield of 9a (%)[b]
1	**1**	^{t}BuONa (1)	DMF	rt	15	51	0	0
2	**1.2**	^{t}BuONa (2)	DMF	rt	15	57	0	0
3	**2**	^{t}BuONa (2)	DMF	rt	15	89	0	0
4	**3**	^{t}BuONa (2)	DMF	rt	15	86	0	0

5	**2**	NaOTMS (2)	DMF	rt	15	89	0	0
6	**1.1**	NaH (2)	DMF	rt	15	71	0	0
7	**2**	NaH (2)	DMF	rt	15	99	0	0
8	**2**	NaH (2)	THF	rt	15	27	0	0
9	**2**	NaH (2)	Et_2O	rt	15	29	0	0
10	**1.4**	NaH (2)	DMF	rt	15	86	0	0

[a] Reaction conditions: **1a** (0.05 mmol), base (2.0 equiv), solvent (0.03 M) were used under N_2 atmosphere unless otherwise stated. [b] Yields were determined by ^{1}H-NMR analysis using TMB as internal standard.

2.3 Optimization with portion-wise addition of base

An oven dried scintillation vial was charged with ‘base 1’, then DMF (0.5 mL) was added, followed by **2a**. The solution was stirred for 30 min at room temperature and was referred to as the “enolate solution”. After an oven dried microwave vial was charged with **1a**, the vessel was sealed, connected to the vacuum line and DMF (1.0 mL) was added under N_2 atmosphere. Following that, the enolate solution was added dropwise and the reaction mixture was allowed to stir for ‘time 1’. After that, ‘base 2’ was added and reaction was continued for ‘time 2’. Then the reaction mixture was extracted with ethyl acetate and water, volatiles were removed under reduced pressure. Crude ^{1}H-NMR was measured afterwards using TMB as internal standard. Selected optimization reactions are listed below.

Table S4: Optimization with portion-wise addition of bases[a]

**entry**	**2a (equiv)**	**‘base 1’ (equiv)**	**‘base 2’ (equiv)**	**‘time 1’ (h)**	**‘time 2’ (h)**	**yield of 3a (%)[b]**	**yield of 3a’ (%)[b]**	**yield of 9a (%)[b]**
1	**1.2**	NaH (1.1)	NaH (1.0)	3	15	32	0	0
2	**1.2**	NaOTMS (1.1)	NaOTMS (1.1)	3	15	59	0	0
3	**1.2**	NaH (1.2)	K_2CO_3 (1.0)	3	15	49	15	0

[a] Reaction conditions: **1a** (0.05 mmol), base, DMF (0.03 M) were used under N_2 atmosphere unless otherwise stated. [b] Yields were determined by ^{1}H-NMR analysis using TMB as internal standard.

2.4 Optimization performed with pentane-washed NaH

The reactions were set up following the procedure from section 2.2 using pentane washed NaH and stirred for 15 h at the tabulated temperature. Then the reaction mixture was extracted with ethyl acetate and water, and volatiles were removed under reduced pressure. Crude ^{1}H-NMR was measured afterwards using TMB as internal standard. Selected optimization reactions are listed below.

Table S5: Optimization reactions with pentane-washed base[a]

entry	2a (equiv)	NaH_w (equiv)	scale 1a (mmol)	T (°C)	solvent	yield of 3a (%)[b]	yield of 3a' (%)[b]	yield of 9a (%)[b]
1	**1.0**	1.0	0.05	rt	DMA	47	0	0
2	**1.0**	2.0	0.05	rt	DMF	78	0	0
3	**1.0**	2.0	0.05	rt	DMA	79	0	0
4	**1.1**	2.4	0.2	0 °C to rt	DMA	70	0	0
5	**1.3**	2.0	0.2	0 °C to rt	DMA	82	0	0
6	**1.3**	2.4	0.2	0 °C to rt	DMA	93 (87)	0	0
7	**1.3**	2.5	0.2	0 °C to rt	DMA	88	0	0

[a] Reaction conditions: **1a,** pentane-washed NaH, solvent (0.03-0.06 M) were used under N_2 atmosphere unless otherwise stated. [b] Yields were determined by ^{1}H-NMR analysis using TMB as internal standard. Isolated yield in parenthesis.

2.5 Optimization with different iodonium salts

The reactions were set up and analyzed following conditions from section 2.2 using salt **1** and NaH (2.0 equiv) with different counter anions. Crude ^{1}H-NMR was measured afterwards using TMB as internal standard. Selected optimization reactions are listed below.

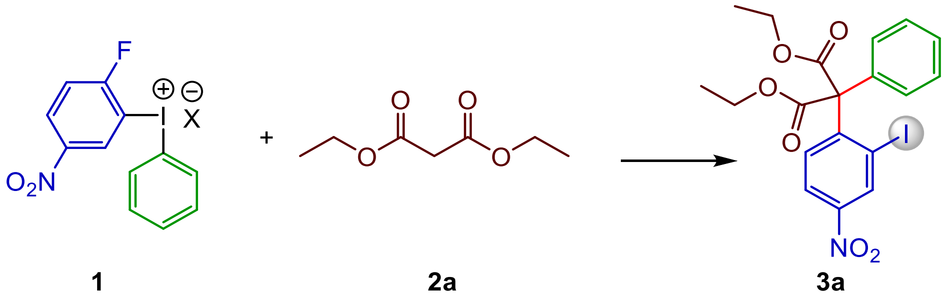

Scheme S2: reactivity of different counter ions.

Table S6: Investigating reactivity of other counter-anions[a]

entry	counter anion	2a (equiv)	scale (mmol)	solvent	T (°C)	Yield of 3a (%)[b]
1	OTs⁻	2.0	0.05	DMF	rt	93
2	Br⁻	2.0	0.05	DMF	rt	84
3	TFA⁻	2.0	0.05	DMF	rt	81
4	OTf⁻	2.0	0.05	DMF	rt	99

[a] Reaction conditions: Unless otherwise stated, **1a** (0.05 mmol), NaH (2.0 equiv), solvent (0.03 M) were used under N_2 atmosphere. [b] Yields were determined by ^{1}H-NMR analysis using TMB as internal standard.

The reactivity-difference of iodonium salts with different anions is well studied, and triflate and tosylate salts often give better results than bromide and carboxylate, as the latter can behave as nucleophiles and also show different solubility. In the present reaction, the anion could influence the formation of key intermediate **III** where weakly coordinating anions (OTf⁻, OTs⁻) are easily displaced by the enolate species.

As proof of S_NAr reactivity a diaryliodonium salt bearing chloride instead of fluoride (**1a-Cl**) (0.05 mmol) was reacted under the reaction conditions (entry 1, Table S6) with 2a (0.1 mmol), NaH (0.1 mmol). Unsurprisingly, the yield of **3a** dropped to 20% (Figure S1).

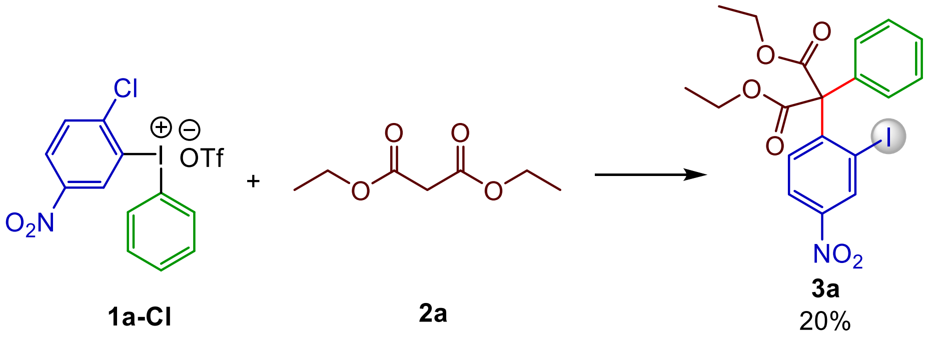

Scheme S3: Reactivity of diaryliodonium salt with Cl⁻ as leaving group.

Unsuccessful salts

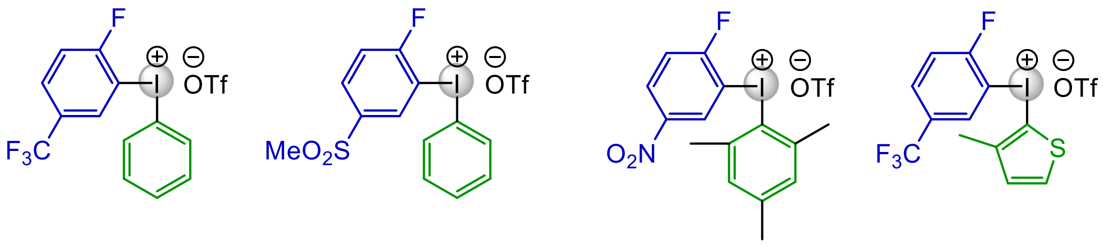

Unsuccessful carbon nucleophiles

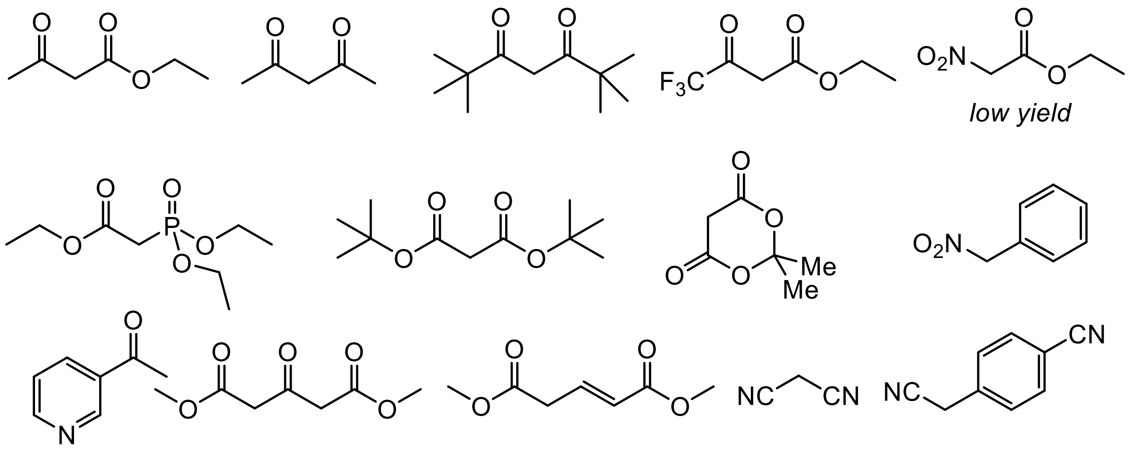

Figure S1: List of unsuccessful substrates

3 Synthesis of diaryl malonates 3

General Procedure 1A1

An oven dried vial was charged with pentane-washed dried sodium hydride (2.4 equiv) under an N_2 atmosphere, then DMA (1.0 mL) was added, followed by dropwise addition of malonate derivative **2** (1.3 equiv). The resulting solution was kept for 30 min – 1h for the complete formation of sodium malonate derivative at room temperature. An oven-dried microwave vial was charged with iodonium salt **1** (1.0 equiv), followed by DMA (1.0 mL) under N_2 atmosphere, the resulting solution was cooled down to 0 °C. The pre-formed enolate solution was added dropwise to the iodonium salt solution over 30 min while keeping the temperature at 0 °C. Then the reaction mixture was left to come to room temperature and stirred overnight. After that, the reaction mixture was quenched with water (40 mL) and organic compounds were extracted with ethyl acetate (2 x 40 mL), volatiles were removed under reduced pressure and the desired compound was isolated via flash chromatography (ethyl acetate/petroleum ether or ethyl acetate/n-pentane).

General Procedure 1A2

The reaction conditions from Table 1, entry 4 were used for some products. The reactions were performed as described for Gerneral Procedure 1A1, but with unwashed sodium hydride (16.0 mg, 0.40 mmol, 2.0 equiv, 60% dispersed in mineral oil), and **2a** (64.0 mg or 60 µL, 0.40 mmol, 2.0 equiv) in DMF (3.0 mL).

General Procedure 1B

Following general procedure 1A1 the reaction was performed and following work-up procedure was adapted. After overnight stirring, the reaction mixture was quenched with 1 N HCl solution (40 mL) and organic compounds were extracted with ethyl acetate (2 x 40 mL), volatiles were removed under reduced pressure and desired compound was isolated via column chromatography (ethyl acetate/petroleum ether or ethyl acetate/n-pentane).

For sustainability reasons, the conditions in GP 1A1 were generally employed in the scope studies, as they require less excess nucleophile. Product yields following General procedure 1A1 and 1A2 are compared in Scheme S3. For some products (**3a-3d**, **3l-3q**), higher yields were obtained with procedure 1A2 compared to procedure 1A1, for others lower yields (**3e**, **3f**) were obtained following procedure 1A2.

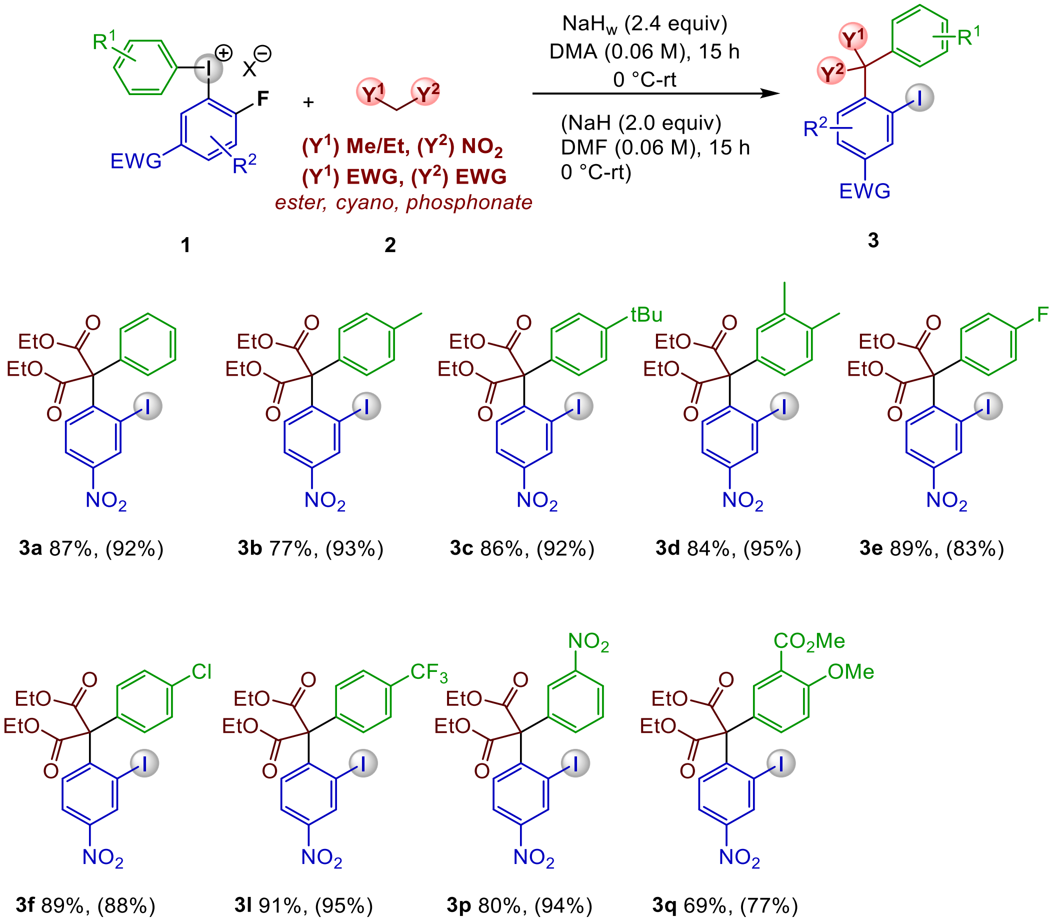

Scheme S4: General procedure for the synthesis of products 3. Yields are reported following General procedure 1A1, yields in parenthesis are reported following general procedure 1A2.

3.1 Synthetic details and analytical data

(Diethyl 2-(2-iodo-4-nitrophenyl)-2-phenylmalonate (3a):

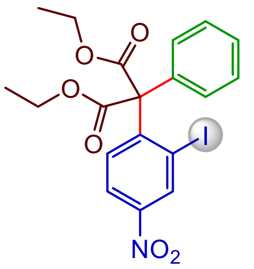

Following the general procedure **1A1** iodonium salt **1a** (98.6 mg, 0.20 mmol, 1.0 equiv), NaH (11.5 mg, 0.48 mmol, 2.4 equiv) and diethyl malonate (41.6 mg or 39 µL, 0.13 mmol, 1.3 equiv) were reacted in DMA (3.0 mL) for 16 h. Product **3a** was isolated via column chromatography (10% ethyl acetate in pentane) as colorless oil which gradually turned into solid (84.4 mg, 0.18 mmol, 87%); m.p. 88.7-89.0 °C. **R*f*** 0.27 (10% ethyl acetate in pentane); **^{1}H NMR (400 MHz, $CDCl_3$)** δ 8.77 (d, *J* = 2.4 Hz, 1H), 8.14 (dd, *J* = 8.7, 2.5 Hz, 1H), 7.45 – 7.39 (m, 3H), 7.36 (ddt, *J* = 6.2, 3.0, 1.8 Hz, 2H), 7.08 (d, *J* = 8.7 Hz, 1H), 4.42 – 4.28 (m, 4H), 1.30 (t, *J* = 7.1 Hz, 6H); **^{13}C NMR (101 MHz, $CDCl_3$)** δ 167.9 (2C), 150.0, 146.6, 136.5, 135.8, 131.1, 129.1 (4C), 128.7, 122.6, 100.7, 72.6, 63.1 (2C), 13.9 (2C); **HRMS** $[M+Na]^+$ calcd. for $C_{19}H_{18}INNaO_6^+$ 506.0071, found 506.0073.

Reported yield following general procedure 1A2: (89.3 mg, 0.19 mmol, 92%).

Diethyl 2-(2-iodo-4-nitrophenyl)-2-(*p*-tolyl)malonate (3b):

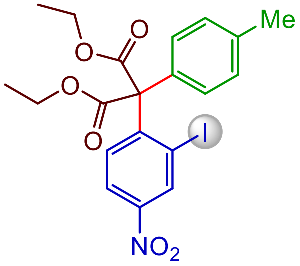

Following the general procedure **1A1** iodonium salt **1b** (101.4 mg, 0.20 mmol, 1.0 equiv), NaH (11.5 mg, 0.48 mmol, 2.4 equiv) and diethyl malonate (41.6 mg or 39 µL, 0.13 mmol, 1.3 equiv) were reacted in DMA (3.0 mL) for 16 h. Product **3b** was isolated via column chromatography (10% ethyl acetate in petroleum ether) as colorless oil which gradually turned into solid (76.6 mg, 0.15 mmol, 77%); **m.p.** 75.4-78.9 °C; **R*f*** 0.30 (10% ethyl acetate in pentane); **^{1}H NMR (400 MHz, $CDCl_3$)** δ 8.74 (dd, *J* = 2.4, 0.8 Hz, 1H), 8.10 (dd, *J* = 8.7, 2.4 Hz, 1H), 7.20 (s, 4H), 7.05 (d, *J* = 8.8 Hz, 1H), 4.40 – 4.23 (m, 4H), 2.37 (s, 3H), 1.27 (t, *J* = 7.1 Hz, 6H); **^{13}C NMR (101 MHz, $CDCl_3$)** δ 167.9 (2C), 150.3, 146.6, 138.7, 135.7, 133.5, 131.1, 129.8 (2C), 128.8 (2C), 122.6, 100.7, 72.3, 63.0 (2C), 21.2, 14.0 (2C); **HRMS** $[M+Na]^+$ calcd. for $C_{20}H_{20}INNaO_6^+$ 520.0228, found 520.0225.

Reported yield following general procedure 1A2: (92.7 mg, 0.19 mmol, 93%).

Diethyl 2-(4-(*tert*-butyl)phenyl)-2-(2-iodo-4-nitrophenyl)malonate (3c):

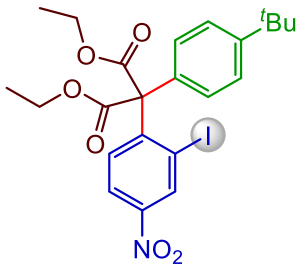

Following the general procedure **1A1** iodonium salt **1c** (109.9 mg, 0.20 mmol, 1.0 equiv) NaH (11.5 mg, 0.48 mmol, 2.4 equiv) and diethyl malonate (41.6 mg or 39 µL, 0.13 mmol, 1.3 equiv) were reacted in DMA (3.0 mL) for 16 h. Product **3c** was isolated via column chromatography (10% ethyl acetate in pentane) as colorless oil which gradually turned into solid (92.8 mg, 0.17 mmol, 86%); **m.p.** 102.8-103.6 °C; **R*f*** 0.40 (10% ethyl acetate in pentane); **^{1}H NMR (400 MHz, $CDCl_3$)** δ 8.74 (d, *J* = 2.4 Hz, 1H), 8.10 (dd, *J* = 8.8, 2.4 Hz, 1H), 7.43 – 7.38 (m, 2H), 7.23 – 7.18 (m, 2H), 6.98 (d, *J* = 8.7 Hz, 1H), 4.42 – 4.23 (m, 4H), 1.33 (s, 9H), 1.28 (t, *J* = 7.1 Hz, 6H); **^{13}C NMR (101 MHz, $CDCl_3$)** δ 167.9 (2C), 151.9, 150.5, 146.6, 135.5, 133.4, 131.2, 128.5 (2C), 126.2 (2C), 122.6, 100.8, 72.4, 63.0 (2C), 34.7, 31.4 (3C), 14.0 (2C); **HRMS** $[M+Na]^+$ calcd. for $C_{23}H_{26}INNaO_6^+$ 562.0697, found 562.0698.

Reported yield following general procedure 1A2: (98.7 mg, 0.18 mmol, 92%).

Diethyl 2-(3,4-dimethylphenyl)-2-(2-iodo-4-nitrophenyl)malonate (3d):

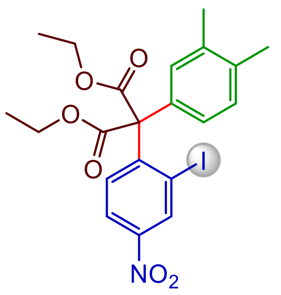

Following the general procedure **1A1** iodonium salt **1d** (102.2 mg, 0.20 mmol, 1.0 equiv), NaH (11.5 mg, 0.48 mmol, 2.4 equiv) and diethyl malonate (41.6 mg or 39 µL, 0.13 mmol, 1.3 equiv) were reacted in DMA (3.0 mL) for 16 h. Product **3d** was isolated via column chromatography (10% ethyl acetate in pentane) as colorless oil (85.9 mg, 0.17 mmol, 84%); **m.p.** 91.7-92.9 °C; **R_f** 0.30 (10% ethyl acetate in pentane); **^{1}H NMR (400 MHz, $CDCl_3$)** δ 8.73 (d, J = 2.4 Hz, 1H), 8.08 (dd, J = 8.8, 2.4 Hz, 1H), 7.15 (d, J = 8.0 Hz, 1H), 7.06 (d, J = 2.3 Hz, 1H), 7.02 – 6.95 (m, 2H), 4.40 – 4.25 (m, 4H), 2.28 (s, 3H), 2.25 (s, 3H), 1.28 (t, J = 7.1 Hz, 6H); **^{13}C NMR (101 MHz, $CDCl_3$)** δ 167.9 (2C), 150.6, 146.5, 137.5, 137.5, 135.5, 133.8, 131.2, 130.4, 129.8, 126.2, 122.5, 100.8, 72.4, 62.9 (2C), 20.3, 19.6, 14.0 (2C); **HRMS:** $[M+Na]^+$ calcd. for $C_{21}H_{22}INNaO_6^+$ 534.0384, found 534.0380.
Reported yield following general procedure 1A2: (97.0 mg, 0.19 mmol, 95%).

Diethyl 2-(4-fluorophenyl)-2-(2-iodo-4-nitrophenyl)malonate (3e):

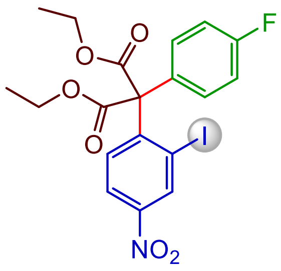

Following the general procedure **1A1** iodonium salt **1e** (102.2 mg, 0.20 mmol, 1.0 equiv), NaH (11.5 mg, 0.48 mmol, 2.4 equiv) and diethyl malonate (41.6 mg or 39 µL, 0.13 mmol, 1.3 equiv) were reacted in DMA (3.0 mL) for 16 h. Product **3e** was isolated via column chromatography (10% ethyl acetate in pentane) as colorless oil (89.2 mg, 0.18 mmol, 89%); **m.p.** 125.2-126.5 °C; **R_f** 0.30 (10% ethyl acetate in pentane); **^{1}H NMR (400 MHz, $CDCl_3$)** δ 8.75 (d, J = 2.5 Hz, 1H), 8.18 (dd, J = 8.7, 2.4 Hz, 1H), 7.41 – 7.34 (m, 2H), 7.21 (d, J = 8.7 Hz, 1H), 7.09 – 7.02 (m, 2H), 4.31 (q, J = 7.1 Hz, 4H), 1.26 (t, J = 7.2 Hz, 6H); **^{19}F NMR (377 MHz, $CDCl_3$)** δ -113.1; **^{13}C NMR (101 MHz, $CDCl_3$)** δ 168.0 (2C), 162.6 (d, J = 249.3 Hz), 149.3, 146.7, 136.2, 132.2 (d, J = 3.5 Hz), 131.3 (d, J = 8.3 Hz, 2C), 130.9, 122.8, 115.8 (d, J = 21.5 Hz, 2C), 100.4, 71.7, 63.2 (2C), 13.9 (2C); **HRMS** $[M+Na]^+$ calcd. for $C_{19}H_{17}FINNaO_6^+$ 523.9977, found 523.9979.
Reported yield following general procedure 1A2: (83.2 mg, 0.17 mmol, 83%).

Diethyl 2-(4-chlorophenyl)-2-(2-iodo-4-nitrophenyl)malonate (3f):

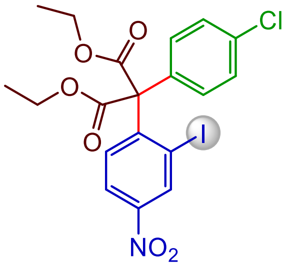

Following the general procedure **1A1** iodonium salt **1f** (102.2 mg, 0.20 mmol, 1.0 equiv), NaH (11.5 mg, 0.48 mmol, 2.4 equiv) and diethyl malonate (41.6 mg or 39 µL, 0.13 mmol, 1.3 equiv) were reacted in DMA (3.0 mL) for 16 h. Product **3f** was isolated via column chromatography (10% ethyl acetate in pentane) as colorless oil (89.2 mg, 0.18 mmol, 89%); **R_f** 0.37 (10% ethyl acetate in pentane); **^{1}H NMR (400 MHz, $CDCl_3$)** δ 8.75 (d, J = 2.4 Hz, 1H), 8.19 (dd, J = 8.8, 2.5 Hz, 1H), 7.34 (s, 4H), 7.24 (d, J = 8.7 Hz, 1H), 4.31 (q, J = 7.1 Hz, 4H), 1.26 (t, J = 7.1 Hz, 6H); **^{13}C NMR (101 MHz, $CDCl_3$)** δ 167.8 (2C), 148.9, 146.7, 136.3, 134.9, 134.7, 130.9, 130.9 (2C), 128.9 (2C), 122.8, 100.3, 71.8, 63.3 (2C), 13.9 (2C); **HRMS** $[M+Na]^+$ calcd. for $C_{19}H_{17}ClINNaO_6^+$ 539.9681, found 539.9682.
Reported yield following general procedure 1A2: (90.8 mg, 0.18 mmol, 88%).

Diethyl 2-(4-bromophenyl)-2-(2-iodo-4-nitrophenyl)malonate (3g):

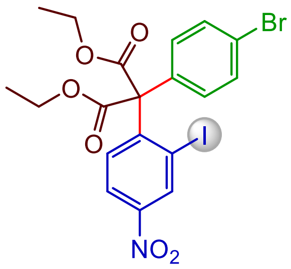

Following the general procedure **1A1** iodonium salt **1g** (114.4 mg, 0.20 mmol, 1.0 equiv), NaH (11.5 mg, 0.48 mmol, 2.4 equiv) and diethyl malonate (41.6 mg or 39 µL, 0.26 mmol, 1.3 equiv) were reacted in DMA (3.0 mL) for 16 h. Product **3g** was isolated via column chromatography (10% diethyl ether in petroleum ether) as colorless sticky solid. (99.5 mg, 0.18 mmol, 89%); **R*f*** 0.29 (10% ethyl acetate in pentane); **^{1}H NMR (400 MHz, CDCl$_3$)** δ 8.77 (d, *J* = 2.5 Hz, 1H), 8.21 (dd, *J* = 8.7, 2.5 Hz, 1H), 7.55 – 7.47 (m, 2H), 7.32 – 7.28 (m, 2H), 7.27 (d, *J* = 8.7 Hz, 1H), 4.33 (q, *J* = 7.1 Hz, 4H), 1.29 (t, *J* = 7.1 Hz, 6H); **^{13}C NMR (101 MHz, CDCl$_3$)** δ 167.7 (2C), 148.8, 146.7, 136.2, 135.4, 131.8 (2C), 131.1 (2C), 130.9, 122.9, 122.8, 100.3, 71.9, 63.3 (2C), 13.9 (2C); **HRMS** [M+Na]$^+$ calcd. for $C_{19}H_{17}BrINNaO_6^+$ 583.9176, found 583.9176.

Diethyl 2-(2-iodo-4-nitrophenyl)-2-(4-methoxyphenyl)malonate (3h):

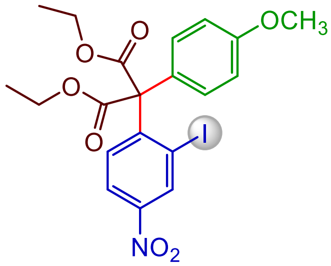

Following the general procedure **1A1** iodonium salt **1h** (104.6 mg, 0.20 mmol, 1.0 equiv), NaH (11.5 mg, 0.48 mmol, 2.4 equiv) and diethyl malonate (41.6 mg or 39 µL, 0.26 mmol, 1.3 equiv) were reacted in DMA (3.0 mL) for 16 h. Product **3h** was isolated via column chromatography (2% diethyl ether in petroleum ether) as light yellow sticky solid (87.2 mg, 0.17 mmol, 85%); **R*f*** 0.67 (40% ethyl acetate in pentane); **^{1}H NMR (400 MHz, CDCl$_3$)** δ 8.76 (d, *J* = 2.4 Hz, 1H), 8.14 (dd, *J* = 8.8, 2.4 Hz, 1H), 7.30 – 7.24 (m, 2H), 7.13 (d, *J* = 8.7 Hz, 1H), 6.96 – 6.90 (m, 2H), 4.34 (app qd, *J* = 7.1, 4.4 Hz, 4H), 3.85 (s, 3H), 1.29 (t, *J* = 7.1 Hz, 6H); **^{13}C NMR (101 MHz, CDCl$_3$)** δ 168.1 (2C), 159.6, 150.3, 146.6, 135.8, 131.1, 130.3 (2C), 128.4, 122.6, 114.3 (2C), 100.6, 71.9, 63.0 (2C), 55.5, 14.0 (2C); **HRMS** [M+Na]$^+$ calcd. for $C_{20}H_{20}INNaO_7^+$ 536.0177, found 536.0172.

Diethyl 2-(2-iodo-4-nitrophenyl)-2-(4-phenoxyphenyl)malonate (3i):

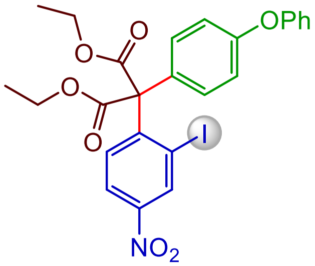

Following the general procedure **1A1** iodonium tosylate **1i** (121.5 mg, 0.20 mmol, 1.0 equiv), NaH (11.5 mg, 0.48 mmol, 2.4 equiv) and diethyl malonate (41.6 mg or 39 µL, 0.13 mmol, 1.3 equiv) were reacted in DMA (3.0 mL) for 16 h. Product **3i** was isolated via column chromatography (10% ethyl acetate in pentane) colorless sticky solid (90.9 mg, 0.16 mmol, 79%); **R*f*** 0.63 (20% ethyl acetate in pentane); **^{1}H NMR (400 MHz, CDCl$_3$)** δ 8.75 (d, *J* = 2.4 Hz, 1H), 8.15 (dd, *J* = 8.8, 2.5 Hz, 1H), 7.40 – 7.33 (m, 2H), 7.32 – 7.27 (m, 2H), 7.18 – 7.12 (m, 2H), 7.08 – 7.04 (m, 2H), 7.01 – 6.96 (m, 2H), 4.38 – 4.26 (m 4H), 1.28 (t, *J* = 7.1 Hz, 6H).; **^{13}C NMR (101 MHz, CDCl$_3$)** δ 168.0 (2C), 157.8, 156.4, 149.9, 146.6, 135.9, 131.0, 130.7 (2C), 130.7, 130.0 (2C), 124.1, 122.7, 119.7 (2C), 118.4 (2C), 100.5, 71.9, 63.1 (2C), 13.9 (2C); **HRMS** [M+Na]$^+$ calcd. for $C_{25}H_{22}INNaO_7^+$ 598.0333, found 598.0335.

Diethyl 2-(4-acetamidophenyl)-2-(2-iodo-4-nitrophenyl)malonate (3j):

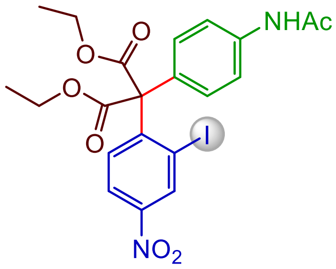

Following the general procedure **1A1** iodonium salt **1j** (110.0 mg, 0.20 mmol, 1.0 equiv)), NaH (11.5 mg, 0.48 mmol, 2.4 equiv) and diethyl malonate (41.6 mg or 39 µL, 0.26 mmol, 1.3 equiv) were reacted in DMA (3.0 mL) for 16 h. Product **3j** was isolated via column chromatography (50% ethyl acetate in petroleum ether) as yellow solid (100.5 mg, 0.19 mmol, 93%); **m.p.** 84.9 °C; **R*f*** 0.60 (pure ethyl acetate); **^{1}H NMR (400 MHz, $CDCl_3$)** δ 8.73 (d, *J* = 2.4 Hz, 1H), 8.13 (dd, *J* = 8.7, 2.5 Hz, 1H), 7.56 – 7.50 (m, 2H), 7.38 (s, 1H), 7.32 – 7.27 (m, 2H), 7.12 (d, *J* = 8.7 Hz, 1H), 4.31 (app qd, *J* = 7.1, 2.6 Hz, 4H), 2.18 (s, 3H), 1.26 (t, *J* = 7.1 Hz, 6H); **^{13}C NMR (101 MHz, $CDCl_3$)** δ 168.5, 167.9 (2C), 149.8, 146.6, 138.3, 135.9, 131.9, 131.1, 129.9 (2C), 122.7, 119.8 (2C), 100.5, 72.0, 63.1 (2C), 24.8, 13.9 (2C); **HRMS** $[M+Na]^+$ calcd. for $C_{21}H_{21}IN_2NaO_7^+$ 563.0286, found 563.0292.

Diethyl 2-([1,1'-biphenyl]-4-yl)-2-(2-iodo-4-nitrophenyl)malonate (3k):

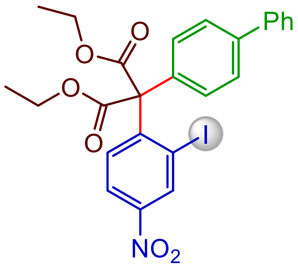

Following the general procedure **1A1** iodonium salt **1k** (101.4 mg, 0.20 mmol, 1.0 equiv)), NaH (11.5 mg, 0.48 mmol, 2.4 equiv) and diethyl malonate (41.6 mg or 39 µL, 0.26 mmol, 1.3 equiv) were reacted in DMA (3.0 mL) for 16 h. Product **3k** was isolated via column chromatography (10% diethyl ether in petroleum ether) as off-white solid (101.6 mg, 0.18 mmol, 91%); **m.p.** 106.5-107.4 °C; **R*f*** 0.66 (20% ethyl acetate in pentane); **^{1}H NMR (400 MHz, $CDCl_3$)** δ 8.77 (d, *J* = 2.4 Hz, 1H), 8.15 (dd, *J* = 8.7, 2.4 Hz, 1H), 7.65 – 7.59 (m, 4H), 7.49 – 7.35 (m, 5H), 7.16 (d, *J* = 8.8 Hz, 1H), 4.35 (app qd, *J* = 7.1, 4.5 Hz, 4H), 1.30 (t, *J* = 7.2 Hz, 6H); **^{13}C NMR (101 MHz, $CDCl_3$)** δ 167.9 (2C), 149.9, 146.7, 141.5, 140.1, 135.9, 135.4, 131.1, 129.5 (2C), 129.0 (2C), 127.9, 127.6 (2C), 127.2 (2C), 122.7, 100.7, 72.4, 63.1 (2C), 14.0 (2C); **HRMS** $[M+Na]^+$ calcd. for $C_{25}H_{22}INNaO_6^+$ 582.0384, found 582.0382.

Diethyl 2-(2-iodo-4-nitrophenyl)-2-(4-(trifluoromethyl)phenyl)malonate (3l):

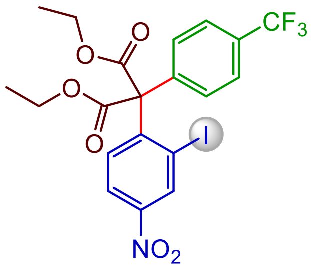

Following the general procedure **1A1** iodonium salt **1l** (112.2 mg, 0.20 mmol, 1.0 equiv) , NaH (11.5 mg, 0.48 mmol, 2.4 equiv) and diethyl malonate (41.6 mg or 39 µL, 0.13 mmol, 1.3 equiv) were reacted in DMA (3.0 mL) for 16 h. Product **3l** was isolated via column chromatography (10% ethyl acetate in pentane) as colorless oil which gradually turned into solid (100.3 mg, 0.18 mmol, 91%); **m.p.** 109.1-110.3 °C; **R*f*** 0.32 (10% ethyl acetate in pentane); **^{1}H NMR (400 MHz, $CDCl_3$)** δ 8.77 (d, *J* = 2.4 Hz, 1H), 8.22 (dd, *J* = 8.7, 2.5 Hz, 1H), 7.65 – 7.59 (m, 2H), 7.59 – 7.53 (m, 2H), 7.28 (d, *J* = 8.7 Hz, 1H), 4.33 (app qd, *J* = 7.2, 1.2 Hz, 4H), 1.27 (t, *J* = 7.2 Hz, 6H); **^{13}C NMR (101 MHz, $CDCl_3$)** δ 167.7 (2C), 148.4, 146.8, 140.2, 136.4, 130.9, 130.6 (d, *J* = 32.7 Hz), 130.1 (2C), 125.5 (q, *J* = 3.8 Hz, 2C), 124.0 (app d, *J* = 272.2 Hz), 122.9, 100.3, 72.1, 63.4 (2C), 13.9 (2C); **^{19}F NMR (377 MHz, $CDCl_3$)** δ -62.8; **HRMS** $[M+Na]^+$ calcd. for $C_{20}H_{17}F_3INNaO_6^+$ 573.9945, found 573.9946.
Reported yield following general procedure 1A2: (104.7 mg, 0.19 mmol, 95%).

Diethyl 2-(2-iodo-4-nitrophenyl)-2-(4-(trifluoromethoxy)phenyl)malonate (3m):

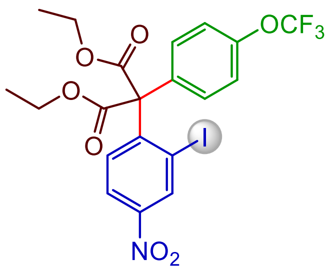

Following the general procedure **1A1** iodonium salt **1m** (115.4 mg, 0.20 mmol, 1.0 equiv), NaH (11.5 mg, 0.48 mmol, 2.4 equiv) and diethyl malonate (41.6 mg or 39 µL, 0.13 mmol, 1.3 equiv) were reacted in DMA (3.0 mL) for 16 h. Product **3m** was isolated via column chromatography (10% ethyl acetate in pentane) as colorless oil (104.4 mg, 0.18 mmol, 92%); **m.p.** 138.4-139.0 °C; **R_f** 0.36 (10% ethyl acetate in pentane); **^{1}H NMR (400 MHz, $CDCl_3$)** δ 8.75 (d, *J* = 2.4 Hz, 1H), 8.20 (dd, *J* = 8.7, 2.5 Hz, 1H), 7.47 – 7.43 (m, 2H), 7.25 (d, *J* = 8.7 Hz, 1H), 7.20 (dt, *J* = 7.9, 1.1 Hz, 2H), 4.32 (q, *J* = 7.1 Hz, 4H), 1.27 (t, *J* = 7.1 Hz, 6H); **^{13}C NMR (101 MHz, $CDCl_3$)** δ 167.8 (2C), 149.2 (q, *J* = 1.9 Hz), 148.8, 146.8, 136.3, 134.9, 131.1 (2C), 130.9, 122.8, 120.7 (2C), 120.5 (q, *J* = 257.9 Hz), 100.3, 71.8, 63.3 (2C), 13.9 (2C); **^{19}F NMR (377 MHz, $CDCl_3$)** δ -57.8. **HRMS** $[M+Na]^+$ calcd. for $C_{20}H_{17}F_3INNaO_7^+$ 589.9894, found 589.9893.

Diethyl 2-(4-acetylphenyl)-2-(2-iodo-4-nitrophenyl)malonate (3n):

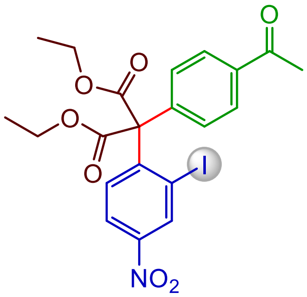

Following the general procedure **1A1** iodonium salt **1n** (94.6 mg, 0.20 mmol, 1.0 equiv)), NaH (11.5 mg, 0.48 mmol, 2.4 equiv) and diethyl malonate (41.6 mg or 39 µL, 0.26 mmol, 1.3 equiv) were reacted in DMA (3.0 mL) for 16 h. Product **3n** was isolated via column chromatography (20% ethyl acetate in petroleum ether) as a yellow solid (86.2 mg, 0.16 mmol, 82%); **m.p.** 114.7-115.7 °C; **R_f** 0.70 (40% ethyl acetate in pentane); **^{1}H NMR (400 MHz, $CDCl_3$)** δ 8.76 (d, *J* = 2.4 Hz, 1H), 8.20 (dd, *J* = 8.7, 2.5 Hz, 1H), 7.96 – 7.91 (m, 2H), 7.55 – 7.50 (m, 2H), 7.25 (d, *J* = 8.8 Hz, 1H), 4.32 (q, *J* = 7.1 Hz, 4H), 2.61 (s, 3H), 1.27 (t, *J* = 7.1 Hz, 6H); **^{13}C NMR (101 MHz, $CDCl_3$)** δ 197.6, 167.7 (2C), 148.6, 146.8, 141.3, 136.8, 136.3, 130.9, 129.8 (2C), 128.5 (2C), 122.9, 100.4, 72.3, 63.4 (2C), 26.8, 13.9 (2C); **HRMS** $[M+Na]^+$ calcd. for $C_{21}H_{20}INNaO_7^+$ 548.0177, found 548.0176.

Diethyl 2-(4-cyanophenyl)-2-(2-iodo-4-nitrophenyl)malonate (3o):

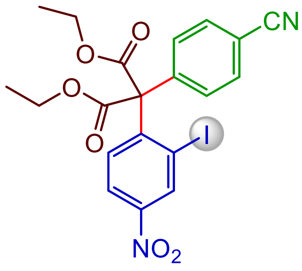

Following the general procedure **1A1** iodonium salt **1o** (103.6 mg, 0.20 mmol, 1.0 equiv), NaH (11.5 mg, 0.48 mmol, 2.4 equiv) and diethyl malonate (41.6 mg or 39 µL, 0.13 mmol, 1.3 equiv) were reacted in DMA (3.0 mL) for 16 h. Product **3o** was isolated through column chromatography (10% ethyl acetate in petroleum ether) as colorless oil which gradually turned into solid (83.1 mg, 0.16 mmol, 82%); **m.p**. 106.6-107.3 °C; **R_f** 0.10 (10% ethyl acetate in petroleum ether); **^{1}H NMR (400 MHz, $CDCl_3$)** δ 8.76 (d, *J* = 2.4 Hz, 1H), 8.26 (dd, *J* = 8.7, 2.4 Hz, 1H), 7.66 – 7.58 (m, 4H), 7.37 (d, *J* = 8.7 Hz, 1H), 4.39 – 4.24 (m, 4H), 1.26 (t, *J* = 7.1 Hz, 6H); **^{13}C NMR (101 MHz, $CDCl_3$)** δ 167.5 (2C), 147.7, 147.0, 141.4, 136.6, 132.1 (2C), 130.9, 130.6 (2C), 123.1, 118.5, 112.4, 100.1, 72.1, 63.6 (2C), 13.9 (2C); **HRMS** $[M+Na]^+$ $C_{20}H_{17}IN_2O_6$: 531.0024; found: 531.0020.

Diethyl 2-(2-iodo-4-nitrophenyl)-2-(3-nitrophenyl)malonate (3p):

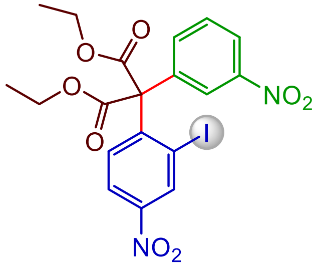

Following the general procedure **1A1** iodonium salt **1p** (107.6 mg, 0.20 mmol, 1.0 equiv), NaH (11.5 mg, 0.48 mmol, 2.4 equiv) and diethyl malonate (41.6 mg or 39 µL, 0.13 mmol, 1.3 equiv) were reacted in DMA (3.0 mL) for 16 h. Product **3p** was isolated via column chromatography (20% ethyl acetate in petroleum ether) as yellow solid (84.5 mg, 0.16 mmol, 80%); **m.p.** 127.8-128.8 °C; $\mathbf{R_f}$ 0.67 (40% ethyl acetate in pentane); **^{1}H NMR (400 MHz, $CDCl_3$)** δ 8.77 (d, *J* = 2.4 Hz, 1H), 8.53 (t, *J* = 2.1 Hz, 1H), 8.29 (dd, *J* = 8.7, 2.5 Hz, 1H), 8.22 (ddd, *J* = 8.2, 2.2, 1.0 Hz, 1H), 7.70 (ddd, *J* = 8.1, 2.0, 1.1 Hz, 1H), 7.51 (t, *J* = 8.1 Hz, 1H), 7.42 (d, *J* = 8.7 Hz, 1H), 4.42 – 4.27 (m, 4H), 1.28 (t, *J* = 7.1 Hz, 6H).; **^{13}C NMR (101 MHz, $CDCl_3$)** δ 167.6 (2C), 148.2, 147.5, 147.0, 138.3, 136.8, 135.9, 130.9, 129.2, 125.2, 123.3, 123.2, 100.0, 71.7, 63.7 (2C), 13.9 (2C); **HRMS** $[M+Na]^+$ calcd. for $C_{19}H_{17}IN_2NaO_8^+$ 550.9922, found 550.9927.

Reported yield following general procedure 1A2: (99.7 mg, 0.19 mmol, 94%).

Diethyl 2-(2-iodo-4-nitrophenyl)-2-(4-methoxy-3-(methoxycarbonyl)phenyl)malonate (3q):

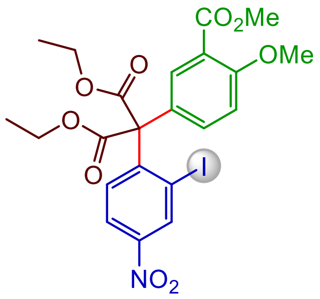

Following the general procedure **1A1** iodonium salt **1q** (116.4 mg, 0.20 mmol, 1.0 equiv), NaH (11.5 mg, 0.48 mmol, 2.4 equiv) and diethyl malonate (41.6 mg or 39 µL, 0.13 mmol, 1.3 equiv) were reacted in DMA (3.0 mL) for 16 h. Product **3q** was isolated via column chromatography (30% ethyl acetate in petroleum ether) as colorless oil which gradually turned into solid (78.6 mg, 0.14 mmol, 69%); **m.p.** 65 °C; $\mathbf{R_f}$ 0.23 (40% ethyl acetate in pentane); **^{1}H NMR (400 MHz, $CDCl_3$)** δ 8.75 (d, *J* = 2.4 Hz, 1H), 8.18 (dd, *J* = 8.7, 2.5 Hz, 1H), 7.93 (d, *J* = 2.7 Hz, 1H), 7.41 (dd, *J* = 8.9, 2.7 Hz, 1H), 7.22 (d, *J* = 8.8 Hz, 1H), 6.96 (d, *J* = 9.0 Hz, 1H), 4.32 (app qd, *J* = 7.2, 1.1 Hz, 4H), 3.92 (s, 3H), 3.85 (s, 3H), 1.27 (t, *J* = 7.1 Hz, 6H); **^{13}C NMR (101 MHz, $CDCl_3$)** δ 167.9 (2C), 166.2, 159.1, 149.1, 146.7, 136.2, 134.5, 132.9, 130.9, 128.0, 122.8, 120.0, 112.2, 100.3, 71.4, 63.2 (2C), 56.2, 52.3, 13.9 (2C); **HRMS:** $[M+Na]^+$ calcd. for $C_{22}H_{22}INNaO_9^+$ 594.0231, found 594.0232.

Reported yield following general procedure 1A2: (87.9 mg, 0.15 mmol, 77%).

Diethyl 2-(2-iodo-4-nitrophenyl)-2-(*o*-tolyl)malonate (3r):

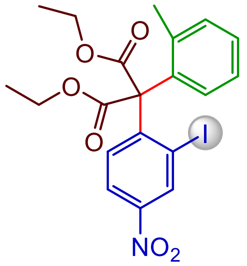

Following the general procedure **1A1** iodonium salt **1r** (37.0 mg, 73 µmol, 1.0 equiv), NaH (4.2 mg, 0.175 mmol, 2.4 equiv) and diethyl malonate (15.2 mg or 14 µL, 95 µmol, 1.3 equiv) were reacted in DMA (1.0 mL) for 16 h. Product **3r** was isolated via column chromatography (10% ethyl acetate in petroleum ether) as colorless oil which gradually turned into solid (26.8 mg, 0.15 mmol, 74%); **m.p.** 118.4.4-119.5 °C; $\mathbf{R_f}$ 0.15 (5% ethyl acetate in petroleum ether); **^{1}H NMR (400 MHz, $CDCl_3$)** δ 8.84 (d, *J* = 2.5 Hz, 1H), 8.04 (dd, *J* = 8.8, 2.5 Hz, 1H), 7.36 – 7.15 (m, 4H), 7.01 (d, *J* = 8.8 Hz, 1H), 4.44 – 4.24 (m, 4H), 2.02 (s, 3H), 1.29 (t, *J* = 7.1 Hz, 6H); **^{13}C NMR (101 MHz, $CDCl_3$)** δ 167.7 (2C), 147.7, 146.5, 138.2, 136.6, 135.5, 132.8, 131.5, 129.3, 128.9, 127.0, 122.2, 101.3, 72.7, 63.1 (2C), 21.5, 13.9 (2C); **HRMS** $[M+Na]^+$ $C_{20}H_{20}INNaO_6^+$ 520.0228; found: 520.0239.

Diethyl 2-(2-iodo-4-nitrophenyl)-2-(2-(methoxycarbonyl)phenyl)malonate (3s):

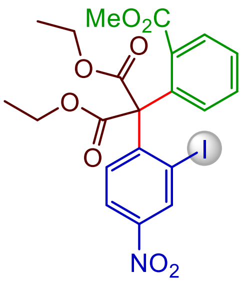

Following the general procedure **1A1** iodonium salt **1s** (97.8 mg, 0.20 mmol, 1.0 equiv), NaH (11.5 mg, 0.48 mmol, 2.4 equiv) and diethyl malonate (41.6 mg or 39 µL, 0.13 mmol, 1.3 equiv) were reacted in DMA (3.0 mL) for 16 h. Product **3s** was isolated through column chromatography (10% ethyl acetate in petroleum ether) as colorless oil which gradually turned into an off-white solid (68.4 mg, 0.13 mmol, 63%); **m.p.** 155.3-157.0 °C; $\mathbf{R_f}$ 0.15 (10% ethyl acetate in petroleum ether); ^{1}**H NMR (400 MHz, $CDCl_3$)** δ 8.80 (d, *J* = 2.5 Hz, 1H), 8.28 (dd, *J* = 8.8, 2.5 Hz, 1H), 7.92 (dd, *J* = 7.8, 1.5 Hz, 1H), 7.47 – 7.37 (m, 2H), 7.31 (td, *J* = 7.7, 1.6 Hz, 1H), 6.69 (d, *J* = 8.1 Hz, 1H), 4.40 – 4.20 (m, 4H), 3.84 (s, 3H), 1.24 (t, *J* = 7.1 Hz, 6H); ^{13}**C NMR (101 MHz, $CDCl_3$)** δ 169.4, 168.3 (2C), 148.4, 146.7, 136.7, 134.4, 133.3, 131.4, 130.9 (2C), 130.8, 128.2, 123.2, 100.8, 72.3, 63.3 (2C), 52.2, 13.9 (2C) (one peak missing due to overlap); **HRMS** $[M+Na]^+$ $C_{21}H_{20}INNaO_8^+$: 564.0126; found: 564.0124.

Diethyl 2-(2-iodo-4-nitro-5-(trifluoromethyl)phenyl)-2-phenylmalonate (3t):

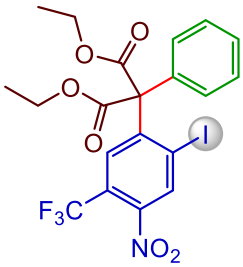

Following the general procedure **1A1** iodonium salt **1t** (112.2 mg, 0.20 mmol, 1.0 equiv)), NaH (11.5 mg, 0.48 mmol, 2.4 equiv) and diethyl malonate (41.6 mg or 39 µL, 0.26 mmol, 1.3 equiv) were reacted in DMA (3.0 mL) for 16 h. Product **3t** was isolated via column chromatography (2% diethyl ether in petroleum ether) as a yellow solid (96.1 mg, 0.17 mmol, 87%); $\mathbf{R_f}$ 0.26 (10% ethyl acetate in pentane); **m.p.** 103.3-104.1 °C; ^{1}**H NMR (400 MHz, $CDCl_3$)** δ 8.42 (s, 1H), 7.45 – 7.40 (m, 3H), 7.34 – 7.30 (m, 2H), 7.30 – 7.28 (m, 1H), 4.41 – 4.28 (m, 4H), 1.30 (t, *J* = 7.1 Hz, 6H); ^{13}**C NMR (101 MHz, $CDCl_3$)** δ 167.3 (2C), 149.1, 145.9, 137.3, 135.7, 129.3 (2C), 129.1, 128.9 (2C), 121.9 (q, *J* = 273.7 Hz) 122.9 (q, *J* = 34.5 Hz), 120.5, 105.5, 72.4, 63.3 (2C), 14.0 (2C); ^{19}**F NMR (377 MHz, $CDCl_3$)** δ -60.3; **HRMS** $[M+Na]^+$ calcd. for $C_{20}H_{17}F_3INNaO_6^+$ 573.9945, found 573.9948.

Diethyl 2-(2-iodo-6-nitro-4-(trifluoromethyl)phenyl)-2-phenylmalonate (3u):

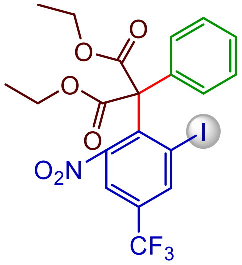

Following the general procedure **1A1** iodonium salt **1u** (112.2 mg, 0.20 mmol, 1.0 equiv), NaH (11.5 mg, 0.48 mmol, 2.4 equiv) and diethyl malonate (41.6 mg or 39 µL, 0.13 mmol, 1.3 equiv) were reacted in DMA (3.0 mL) for 16 h. Product **3u** was isolated via column chromatography (10% ethyl acetate in pentane) as colorless oil which gradually turned into solid (94.8 mg, 0.17 mmol, 86%); **m.p.** 130.5-131.1 °C; $\mathbf{R_f}$ 0.43 (10% ethyl acetate in pentane); ^{1}**H NMR (400 MHz, $CDCl_3$)** δ 8.35 (dt, *J* = 2.0, 0.8 Hz, 1H), 8.17 (dd, *J* = 2.1, 0.8 Hz, 1H), 7.60 – 7.55 (m, 2H), 7.35 – 7.27 (m, 3H), 4.21 (app qd, *J* = 7.1, 2.4 Hz, 4H), 1.19 (t, *J* = 7.1 Hz, 6H); ^{13}**C NMR (101 MHz, $CDCl_3$)** δ 166.3 (2C), 152.2, 142.4 (q, *J* = 3.6 Hz), 141.2, 136.1, 131.1 (q, *J* = 34.9 Hz), 129.9 (2C), 128.4, 128.4 (2C), 122.2 (q, *J* = 3.7 Hz), 121.7 (q, *J* = 273.4 Hz), 103.6, 70.8, 63.2 (2C), 13.7 (2C); ^{19}**F NMR (377 MHz, $CDCl_3$)** δ -62.9; **HRMS** $[M+Na]^+$ calcd. for $C_{20}H_{17}F_3INNaO_6^+$ 573.9945, found 573.9943.

Diethyl 2-(2-fluoro-6-iodo-4-nitrophenyl)-2-phenylmalonate (3v):

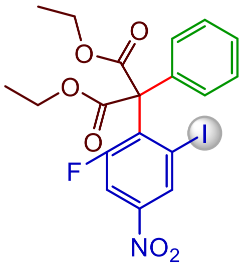

Following the general procedure **1A1** iodonium salt **1v** (102.2 mg, 0.20 mmol, 1.0 equiv), NaH (11.5 mg, 0.48 mmol, 2.4 equiv) and diethyl malonate (41.6 mg or 39 µL, 0.13 mmol, 1.3 equiv) were reacted in DMA (3.0 mL) for 16 h. Product **3v** was isolated via column chromatography (10% ethyl acetate in pentane) as colorless oil (91.2 mg, 0.18 mmol, 91%); **m.p.** 176.8 °C; **R*f*** 0.46 (10% ethyl acetate in pentane); **^{1}H NMR (400 MHz, CDCl$_3$)** δ 8.58 (dd, *J* = 2.4, 1.4 Hz, 1H), 8.02 (dd, *J* = 11.4, 2.4 Hz, 1H), 7.60 – 7.54 (m, 2H), 7.34 – 7.29 (m, 3H), 4.37 – 4.18 (m, 4H), 1.24 (t, *J* = 7.1 Hz, 6H); **^{13}C NMR (101 MHz, CDCl$_3$)** δ 167.6 (2C), 159.9 (d, *J* = 256.4 Hz), 147.3 (d, *J* = 10.7 Hz), 137.5 (d, *J* = 12.2 Hz), 135.9, 132.6 (d, *J* = 3.4 Hz), 130.0 (2C), 128.1 (3C), 112.4 (d, *J* = 32.2 Hz), 101.2 (d, *J* = 2.3 Hz), 68.7 (d, *J* = 1.5 Hz), 63.2 (2C), 13.8 (2C); **^{19}F NMR (377 MHz, CDCl$_3$)** δ -98.1; **HRMS** $[M+Na]^+$ calcd. for $C_{19}H_{17}FINNaO_6^+$ 523.9977, found 523.9973.

Diethyl 2-(3-fluoro-2-iodo-6-nitrophenyl)-2-phenylmalonate (3w):

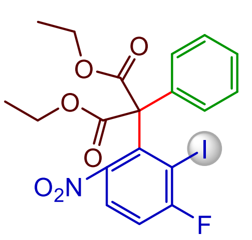

Following the general procedure **1A1** iodonium salt **1w** (102.2 mg, 0.20 mmol, 1.0 equiv), NaH (11.5 mg, 0.48 mmol, 2.4 equiv) and diethyl malonate (41.6 mg or 39 µL, 0.26 mmol, 1.3 equiv) were reacted in DMA (3.0 mL) for 16 h. Product **3w** was isolated via column chromatography (10% ethyl acetate in petroleum ether) as a yellow solid (65.6 mg, 0.13 mmol, 65%); **R*f*** 0.71 (20% ethyl acetate in pentane); **^{1}H NMR (400 MHz, CDCl$_3$)** δ 8.01 (dd, *J* = 9.0, 5.4 Hz, 1H), 7.67 – 7.61 (m, 2H), 7.34 – 7.27 (m, 3H), 7.22 (dd, *J* = 9.0, 6.1 Hz, 1H), 4.29 – 4.15 (m, 4H), 1.20 (t, *J* = 7.1 Hz, 6H); **^{13}C NMR (101 MHz, CDCl$_3$)** δ 166.7 (2C), 163.4 (d, *J* = 249.9 Hz), 148.9 (d, *J* = 2.9 Hz), 140.3 (d, *J* = 1.8 Hz), 136.7, 130.0 (2C), 128.1 (3C), 127.1 (d, *J* = 9.7 Hz), 114.2 (d, *J* = 28.5 Hz), 93.5 (d, *J* = 28.5 Hz), 71.1 (d, *J* = 1.8 Hz), 63.1 (2C), 13.7 (2C); **^{19}F NMR (377 MHz, CDCl$_3$)** δ -75.3; **HRMS** $[M+Na]^+$ calcd. for $C_{19}H_{17}INNaO_6^+$ 523.9977, found 523.9978.

Diethyl 2-(2-fluoro-5-nitrophenyl)-2-(2-iodo-4-nitrophenyl)malonate (3x):

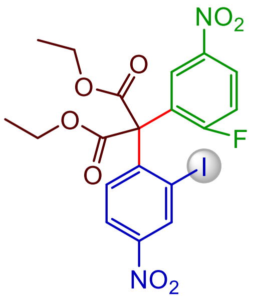

Following the general procedure **1A1** iodonium salt **1x** (111.2 mg, 0.20 mmol, 1.0 equiv)), NaH (11.5 mg, 0.48 mmol, 2.4 equiv) and diethyl malonate (41.6 mg or 39 µL, 0.26 mmol, 1.3 equiv) were reacted in DMA (3.0 mL) for 16 h. Product **3x** was isolated via column chromatography (10% ethyl acetate in petroleum ether) as a yellow sticky material (76.7 mg, 0.14 mmol, 70%) which did not solidified after several days; **R*f*** 0.67 (10% ethyl acetate in pentane); **^{1}H NMR (400 MHz, CDCl$_3$)** δ 8.81 (d, *J* = 2.5 Hz, 1H), 8.52 (dd, *J* = 6.5, 2.8 Hz, 1H), 8.30 (ddd, *J* = 8.9, 4.1, 2.7 Hz, 1H), 8.25 (dd, *J* = 8.8, 2.5 Hz, 1H), 7.42 (dd, *J* = 8.8, 1.3 Hz, 1H), 7.23 (dd, *J* = 10.3, 9.0 Hz, 1H), 4.46 – 4.31 (m, 4H), 1.31 (t, *J* = 7.1 Hz, 6H); **^{13}C NMR (101 MHz, CDCl$_3$)** δ 166.3 (2C), 164.7 (d, *J* = 261.5 Hz), 147.0, 145.5, 143.7 (d, *J* = 2.6 Hz), 137.0, 131.0 (d, *J* = 2.7 Hz), 128.2 (d, *J* = 4.7 Hz), 126.6 (d, *J* = 11.2 Hz), 125.4 (d, *J* = 12.9 Hz), 123.2, 117.5 (d, *J* = 26.3 Hz), 99.4, 69.9 (d, *J* = 2.7 Hz), 64.0 (2C), 13.9 (2C); **^{19}F NMR (377 MHz, CDCl$_3$)** δ -93.7; **HRMS** $[M+Na]^+$ calcd. for $C_{19}H_{16}FIN_2NaO_8^+$ 568.9828, found 568.9821.

Diethyl 2-(2-iodo-4-((trifluoromethyl)sulfonyl)phenyl)-2-phenylmalonate (3y):

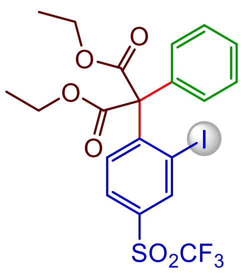

Following the general procedure **1A1** iodonium salt **1y** (116.9 mg, 0.20 mmol, 1.0 equiv)), NaH (11.5 mg, 0.48 mmol, 2.4 equiv) and diethyl malonate (41.6 mg or 39 μL, 0.26 mmol, 1.3 equiv) were reacted in DMA (3.0 mL) for 16 h. Product **3y** was isolated via column chromatography (10% ethyl acetate in petroleum ether) as yellow solid (101.9 mg, 0.18 mmol, 89%); **m.p.** 126.8-127.6 °C; **R*f*** 0.26 (10% ethyl acetate in pentane); **^{1}H NMR (400 MHz, CDCl$_3$)** δ 8.50 (d, *J* = 2.1 Hz, 1H), 7.91 (dd, *J* = 8.4, 2.0 Hz, 1H), 7.44 – 7.39 (m, 3H), 7.35 – 7.30 (m, 2H), 7.14 (d, *J* = 8.4 Hz, 1H), 4.42 – 4.25 (m, 4H), 1.27 (t, *J* = 7.1 Hz, 6H); **^{13}C NMR (101 MHz, CDCl$_3$)** δ 167.5 (2C), 152.4, 142.1, 136.1, 131.8, 131.1 (d, *J* = 1.6 Hz), 129.9, 129.2 (2C), 129.0 (2C), 128.9, 119.7 (q, *J* = 325.7 Hz), 102.0, 72.9, 63.2 (2C), 13.9 (2C); **^{19}F NMR (377 MHz, CDCl$_3$)** δ -78.0. **HRMS** $[M+Na]^+$ calcd. for $C_{20}H_{18}F_3INaO_6^+$ 592.9713, found 592.9711.

Diethyl 2-(4-cyano-2-iodophenyl)-2-phenylmalonate (3z):

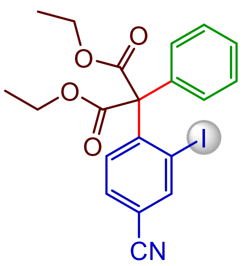

Following the general procedure **1A1** iodonium salt **1z** (116.0 mg, 0.20 mmol, 1.0 equiv)), NaH (11.5 mg, 0.48 mmol, 2.4 equiv) and diethyl malonate (64.1 mg or 61 μL, 0.40 mmol, 2.0 equiv) were reacted in DMA (3.0 mL) for 16 h. Product **3z** was isolated via column chromatography (10% ethyl acetate in petroleum ether) as colorless sticky solid (58.4 mg, 0.13 mmol, 63%); **m.p.** 102.1-102.5 °C; **R*f*** 0.20 (10% ethyl acetate in pentane); **^{1}H NMR (400 MHz, CDCl$_3$)** δ 8.18 (d, *J* = 1.8 Hz, 1H), 7.56 (dd, *J* = 8.2, 1.8 Hz, 1H), 7.40 – 7.36 (m, 3H), 7.35 – 7.31 (m, 2H), 7.00 (d, *J* = 8.3 Hz, 1H), 4.38 – 4.25 (m, 4H), 1.27 (t, *J* = 7.1 Hz, 6H); **^{13}C NMR (101 MHz, CDCl$_3$)** δ 167.9 (2C), 148.3, 143.9, 136.4, 131.4, 131.1, 129.1 (2C), 129.0 (2C), 128.6, 116.9, 112.7, 101.3, 72.7, 63.0 (2C), 13.9 (2C); **HRMS** $[M+Na]^+$ calcd. for $C_{20}H_{18}INNaO_4^+$ 486.0173 found 486.0177.

Diethyl 2-(2-iodo-4-nitrophenyl)-2-(2-oxo-2H-chromen-6-yl)malonate (3aa):

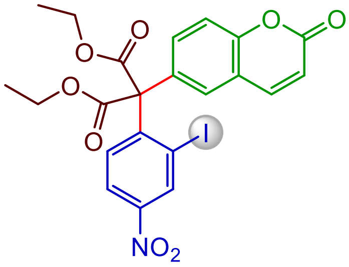

Following the general procedure **1A1** iodonium salt **1aa** (112.2 mg, 0.20 mmol, 1.0 equiv), NaH (11.5 mg, 0.48 mmol, 2.4 equiv) and diethyl malonate (41.6 mg or 39 μL, 0.26 mmol, 1.3 equiv) were reacted in DMA (3.0 mL) for 16 h. Product **3aa** was isolated via column chromatography (20% ethyl acetate in petroleum ether) as off-white solid (78.9 mg, 0.14 mmol, 72%); **m.p.** 199.5-200.9 °C; **R*f*** 0.47 (50% ethyl acetate in pentane); **^{1}H NMR (400 MHz, CDCl$_3$)** δ 8.76 (d, *J* = 2.4 Hz, 1H), 8.25 (dd, *J* = 8.7, 2.5 Hz, 1H), 7.74 (d, *J* = 2.4 Hz, 1H), 7.65 (dd, *J* = 9.7, 0.6 Hz, 1H), 7.49 (dd, *J* = 8.9, 2.4 Hz, 1H), 7.39 (d, *J* = 8.7 Hz, 1H), 7.29 (d, *J* = 8.9 Hz, 1H), 6.43 (d, *J* = 9.6 Hz, 1H), 4.39 – 4.25 (m, 4H), 1.27 (t, *J* = 7.1 Hz, 6H); **^{13}C NMR (101 MHz, CDCl$_3$)** δ 168.0 (2C), 160.5, 153.7, 148.2, 146.9, 143.6, 136.6, 133.2, 132.7, 130.9, 129.3, 123.0, 118.7, 117.2, 117.0, 100.1, 71.4, 63.5 (2C), 13.9 (2C); **HRMS:** $[M+Na]^+$ calcd. for $C_{22}H_{18}INNaO_8^+$ 573.9969, found 573.9968.

Diethyl 2-(2,6-dimethoxypyridin-3-yl)-2-(2-iodo-4-nitrophenyl)malonate (3ab):

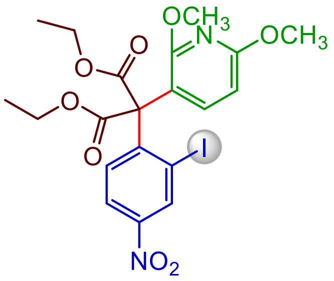

Following the general procedure **1A1** iodonium salt **1ab-OTs** (115.3 mg, 0.20 mmol, 1.0 equiv)), NaH (11.5 mg, 0.48 mmol, 2.4 equiv) and diethyl malonate (64.1 mg or 61 µL, 0.40 mmol, 2.0 equiv) were reacted in DMA (3.0 mL) for 16 h. Product **3ab** was isolated via column chromatography (10% ethyl acetate in petroleum ether) as colorless oil (65.8 mg, 0.12 mmol, 60%); **R*f*** 0.47 (20% ethyl acetate in pentane); **^{1}H NMR (400 MHz, $CDCl_3$)** δ 8.78 (d, *J* = 2.4 Hz, 1H), 8.07 (dd, *J* = 8.8, 2.5 Hz, 1H), 7.44 (d, *J* = 8.4 Hz, 1H), 7.03 (d, *J* = 8.8 Hz, 1H), 6.36 (d, *J* = 8.4 Hz, 1H), 4.37 – 4.24 (m, 4H), 3.96 (s, 3H), 3.82 (s, 3H), 1.28 (t, *J* = 7.1 Hz, 6H); **^{13}C NMR (101 MHz, $CDCl_3$)** δ 167.4 (2C), 163.4, 160.1, 147.6, 146.5, 141.1, 136.3, 131.2, 122.2, 111.2, 101.5, 100.6, 69.2, 63.0 (2C), 53.9, 53.6, 14.0 (2C); **HRMS** $[M+Na]^+$ calcd. for $C_{20}H_{21}IN_2NaO_8^+$ 567.0235, found 567.0237.

Diethyl 2-(4-(1,3-dioxoisoindolin-2-yl)phenyl)-2-(2-iodo-4-nitrophenyl)malonate (3ac):

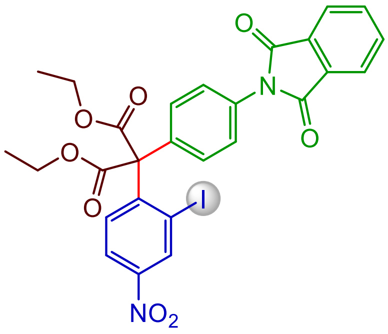

Following the general procedure **1A1** iodonium salt **1ac** (127.7 mg, 0.20 mmol, 1.0 equiv), NaH (11.5 mg, 0.48 mmol, 2.4 equiv) and diethyl malonate (41.6 mg or 39 µL, 0.26 mmol, 1.3 equiv) were reacted in DMA (3.0 mL) for 16 h. Product **3ac** was isolated via column chromatography (20% ethyl acetate in petroleum ether) as a yellow solid (69.9 mg, 0.11 mmol, 56%); **m.p.** 180.5-182.3 °C; **R*f*** 0.30 (40% ethyl acetate in pentane); **^{1}H NMR (400 MHz, $CDCl_3$)** δ 8.76 (d, *J* = 2.4 Hz, 1H), 8.17 (dd, *J* = 8.8, 2.5 Hz, 1H), 7.97 (dd, *J* = 5.5, 3.0 Hz, 2H), 7.81 (dd, *J* = 5.5, 3.1 Hz, 2H), 7.57 – 7.48 (m, 4H), 7.19 (d, *J* = 8.8 Hz, 1H), 4.34 (app qd, *J* = 7.2, 4.5 Hz, 4H), 1.29 (t, *J* = 7.1 Hz, 6H); **^{13}C NMR (101 MHz, $CDCl_3$)** δ 167.7 (2C), 167.1 (2C), 149.5, 146.7, 136.0, 135.8, 134.7 (2C), 132.2, 131.8 (2C), 131.2, 130.0 (2C), 126.4 (2C), 124.0 (2C), 122.8, 100.6, 72.2, 63.2 (2C), 14.0 (2C); **HRMS** $[M+Na]^+$ calcd. for $C_{27}H_{21}IN_2NaO_8^+$ 651.0235, found 651.0239.

Diethyl 2-(5-chloro-2-((1-ethoxy-2-methyl-1-oxopropan-2-yl)oxy)phenyl)-2-(2-iodo-4-nitrophenyl)-malonate (3ad):

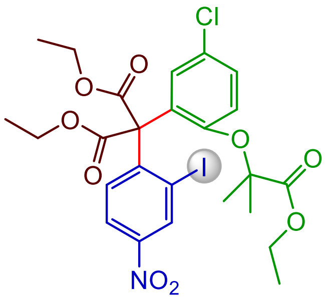

Following the general procedure **1A1** iodonium salt **1ad** (131.8 mg, 0.20 mmol, 1.0 equiv) , NaH (11.5 mg, 0.48 mmol, 2.4 equiv) and diethyl malonate (41.6 mg or 39 µL, 0.26 mmol, 1.3 equiv) were reacted in DMA (3.0 mL) for 16 h. Product **3ad** was isolated via column chromatography (20% ethyl acetate in pentane) as a yellow solid (115.0 mg, 0.18 mmol, 89%); **m.p.** charred at 157.8-158.5 °C; **R*f*** 0.77 (40% ethyl acetate in pentane); **^{1}H NMR (400 MHz, $CDCl_3$)** δ 8.81 (d, *J* = 2.4 Hz, 1H), 8.02 (dd, *J* = 8.8, 2.5 Hz, 1H), 7.28 (dd, *J* = 8.9, 2.6 Hz, 1H), 7.19 (d, *J* = 2.5 Hz, 1H), 7.06 (d, *J* = 8.8 Hz, 1H), 6.51 (d, *J* = 8.8 Hz, 1H), 4.44 (app dq, *J* = 10.9, 7.1 Hz, 2H), 4.27 – 4.16 (m, 4H), 1.40 (s, 6H), 1.32 (t, *J* = 7.1 Hz, 6H), 1.25 (t, *J* = 7.1 Hz, 3H); **^{13}C NMR (101 MHz, $CDCl_3$)** δ 173.2, 167.0 (2C), 151.9, 146.7, 146.5, 136.4, 132.1, 130.3, 129.4, 127.9, 126.2, 121.6, 114.8, 100.7, 79.5, 70.2, 63.1

(2C), 62.0, 24.7, 14.3 (2C), 14.0 (2C); **HRMS** [M+Na]$^+$ calcd. for $C_{25}H_{27}ClINNaO_9^+$ 670.0311 found 670.0315.

Diethyl 2-(2-iodo-4-nitrophenyl)-2-(4-((5-methoxy-4,4-dimethyl-5-oxopentyl)oxy)-2,5-dimethyl-phenyl)malonate (3ae):

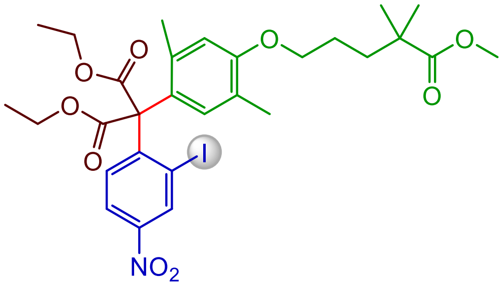

Following the general procedure **1A1** iodonium salt **1ae** (135.9 mg, 0.20 mmol, 1.0 equiv), NaH (11.5 mg, 0.48 mmol, 2.4 equiv) and diethyl malonate (41.6 mg or 39 µL, 0.26 mmol, 1.3 equiv) were reacted in DMA (3.0 mL) for 16 h. Product **3ae** was isolated via column chromatography (10% ethyl acetate in petroleum ether) as a yellow solid (72.7 mg, 0.11 mmol, 54%); **m.p.** 94.8-95.5 °C; **R*f*** 0.77 (40% ethyl acetate in pentane); **^{1}H NMR (400 MHz, $CDCl_3$)** δ 8.82 (d, *J* = 2.4 Hz, 1H), 8.03 (dd, *J* = 8.8, 2.5 Hz, 1H), 7.02 (d, *J* = 8.8 Hz, 1H), 6.89 (s, 1H), 6.65 (s, 1H), 4.46 – 4.22 (m, 4H), 3.96 (t, *J* = 5.6 Hz, 2H), 3.68 (s, 3H), 2.16 (s, 3H), 1.95 (s, 3H), 1.79 – 1.70 (m, 4H), 1.30 (t, *J* = 7.1 Hz, 6H), 1.23 (s, 6H); **^{13}C NMR (101 MHz, $CDCl_3$)** δ 178.4, 167.9 (2C), 157.0, 148.3, 146.5, 136.6, 136.4, 131.6, 131.5, 126.9, 125.0, 122.2, 115.1, 101.4, 72.1, 68.2, 63.0 (2C), 51.9, 42.3, 37.2, 25.3 (2C), 25.3, 21.5, 16.2, 14.0 (2C); **HRMS** [M+Na]$^+$ calcd. for $C_{29}H_{36}INNaO_9^+$ 692.1327, found 692.1326.

Dimethyl 2-(2-iodo-4-nitrophenyl)-2-phenylmalonate (4a):

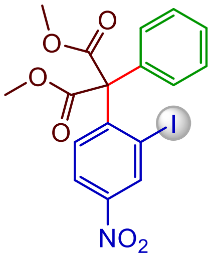

Following the general procedure **1A1** iodonium salt **1a** (98.6 mg, 0.20 mmol, 1.0 equiv), NaH (11.5 mg, 0.48 mmol, 2.4 equiv) and dimethyl malonate (34.3 mg or 30 µL, 0.28 mmol, 1.3 equiv) were reacted in DMA (3.0 mL) for 16 h. Product **4a** was isolated via column chromatography (10% ethyl acetate in petroleum ether) as colorless oil which gradually turned into solid (75.6 mg, 0.166 mmol, 83%); m.p. 153.3-154.4 °C; **R*f*** 0.17 (10% ethyl acetate in pentane); **^{1}H NMR (400 MHz, $CDCl_3$)** δ 8.74 (d, *J* = 2.4 Hz, 1H), 8.11 (dd, *J* = 8.7, 2.4 Hz, 1H), 7.45 – 7.39 (m, 3H), 7.32 – 7.27 (m, 2H), 6.96 (d, *J* = 8.7 Hz, 1H), 3.85 (s, 6H); **^{13}C NMR (101 MHz, $CDCl_3$)** δ 168.3 (2C), 150.0, 146.7, 136.3, 135.7, 131.1, 129.3 (2C), 129.0, 128.9 (2C), 122.7, 100.9, 72.9, 53.9 (2C); **HRMS** [M+Na]$^+$ $C_{17}H_{14}INNaO_6^+$ 477.9758, found 477.9755.

Diisopropyl 2-(2-iodo-4-nitrophenyl)-2-phenylmalonate (4b):

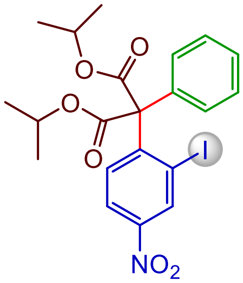

Following the general procedure **1A1** iodonium salt **1a** (98.6 mg, 0.20 mmol, 1.0 equiv), NaH (11.5 mg, 0.48 mmol, 2.4 equiv) and diisopropyl malonate (48.9 mg or 49 µL, 0.26 mmol, 1.3 equiv) were reacted in DMA (3.0 mL) for 16 h. Product **4b** was isolated via column chromatography (5% ethyl acetate in petroleum ether) as light yellow oily solid (95.9 mg, 0.19 mmol, 94%); **R*f*** 0.44 (10% ethyl acetate in pentane); **^{1}H NMR (400 MHz, $CDCl_3$)** δ 8.74 (d, *J* = 2.4 Hz, 1H), 8.14 (dd, *J* = 8.8, 2.5 Hz, 1H), 7.41 – 7.33 (m, 5H), 7.19 (d, *J* = 8.8 Hz, 1H), 5.18 (hept, *J* = 6.3 Hz, 2H), 1.27 (d, *J* = 6.3 Hz, 6H), 1.24 (d, *J* = 6.3 Hz, 6H); **^{13}C NMR (101 MHz, $CDCl_3$)** δ 167.4 (2C), 150.0, 146.5, 136.6, 135.9, 131.1, 129.2 (2C), 128.8 (2C), 128.4, 122.6, 100.5,

72.3, 71.0 (2C), 21.6 (2C), 21.4 (2C); **HRMS** $[M+Na]^+$ calcd. for $C_{21}H_{22}INNaO_6^+$ 534.0348, found 534.0340.

Dibenzyl 2-(2-iodo-4-nitrophenyl)-2-phenylmalonate (4c):

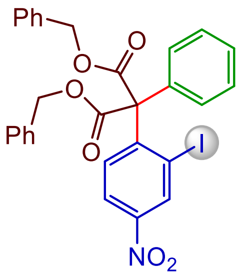

Following the general procedure **1A1** iodonium salt **1a** (98.6 mg, 0.20 mmol, 1.0 equiv), NaH (11.5 mg, 0.48 mmol, 2.4 equiv) and dibenzyl malonate (77.8 mg or 68 µL, 0.26 mmol, 1.3 equiv) were reacted in DMA (3.0 mL) for 16 h. Product **4c** was isolated via column chromatography (10% ethyl acetate in petroleum ether) as colorless sticky solid (113.6 mg, 0.19 mmol, 94%); **R*f*** 0.29 (10% ethyl acetate in pentane); **^{1}H NMR (400 MHz, $CDCl_3$)** δ 8.73 (d, *J* = 2.4 Hz, 1H), 8.05 (dd, *J* = 8.7, 2.4 Hz, 1H), 7.41 – 7.34 (m, 2H), 7.34 – 7.30 (m, 7H), 7.28 – 7.21 (m, 6H), 6.97 (d, *J* = 8.7 Hz, 1H), 5.37 – 5.21 (m, 4H); **^{13}C NMR (101 MHz, $CDCl_3$)** δ 167.6 (2C), 149.7, 146.6, 136.1, 135.7, 134.7, 131.2, 129.0 (4C), 128.7, 128.6 (6C), 128.6 (5C), 122.6, 100.7, 72.7, 68.6 (2C); **HRMS** $[M+Na]^+$ calcd. for $C_{29}H_{22}INNaO_6^+$ 630.0384, found 630.0380.

1-Ethyl 3-methyl 2-(2-iodo-4-nitrophenyl)-2-phenylmalonate (4d):

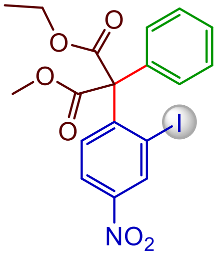

Following the general procedure **1A1** iodonium salt **1a** (98.6 mg, 0.20 mmol, 1.0 equiv), NaH (11.5 mg, 0.48 mmol, 2.4 equiv) and ethyl methyl malonate (38.0 mg, 0.26 mmol, 1.3 equiv) were reacted in DMA (3.0 mL) for 16 h. Product **4d** was isolated via column chromatography (10% ethyl acetate in petroleum ether) as colorless solid (81.6 mg, 0.17 mmol, 87%); **m.p.** 96.6-97.8 °C; **R*f*** 0.53 (20% ethyl acetate in pentane); **^{1}H NMR (400 MHz, $CDCl_3$)** δ 8.75 (d, *J* = 2.4 Hz, 1H), 8.11 (dd, *J* = 8.8, 2.4 Hz, 1H), 7.43 – 7.38 (m, 3H), 7.35 – 7.28 (m, 2H), 7.01 (d, *J* = 8.8 Hz, 1H), 4.42 – 4.25 (m, 2H), 3.84 (s, 3H), 1.27 (t, *J* = 7.1 Hz, 3H); **^{13}C NMR (101 MHz, $CDCl_3$)** δ 168.4, 167.8, 150.0, 146.7, 136.4, 135.8, 131.1, 129.2 (2C), 129.0 (2C), 128.8, 122.7, 100.8, 72.7, 63.2, 53.8, 13.9; **HRMS** $[M+Na]^+$ calcd. for $C_{18}H_{16}INNaO_6^+$ 491.9915, found 491.9912.

1-(*tert*-Butyl) 3-ethyl 2-(2-iodo-4-nitrophenyl)-2-phenylmalonate (4e):

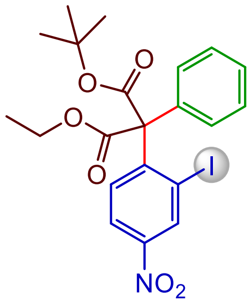

Following the general procedure **1A1** iodonium salt **1a** (98.6 mg, 0.20 mmol, 1.0 equiv), NaH (11.5 mg, 0.48 mmol, 2.4 equiv) and tert-butyl ethyl malonate (48.9 mg, 0.26 mmol, 1.3 equiv) were reacted in DMA (3.0 mL) for 16 h. Product **4e** was isolated via column chromatography (10% diethyl ether in petroleum ether) as oily solid (82.8 mg, 0.16 mmol, 81%); **R*f*** 0.81 (20% ethyl acetate in pentane); **^{1}H NMR (400 MHz, $CDCl_3$)** δ 8.75 (d, *J* = 2.4 Hz, 1H), 8.14 (dd, *J* = 8.8, 2.5 Hz, 1H), 7.41 – 7.35 (m, 5H), 7.18 (d, *J* = 8.7 Hz, 1H), 4.37 – 4.23 (m, 2H), 1.48 (s, 9H), 1.27 (t, *J* = 7.1 Hz, 3H); **^{13}C NMR (101 MHz, $CDCl_3$)** δ 168.2, 166.6, 150.2, 146.5, 136.8, 136.0, 131.1, 129.3 (2C), 128.8 (2C), 128.4, 122.5, 100.5, 84.2, 72.9, 62.8, 27.8 (3C), 14.0; **HRMS** $[M+Na]^+$ $C_{21}H_{22}INNaO_6^+$ 534.0384, found 534.0381.

Methyl 2-cyano-2-(2-iodo-4-nitrophenyl)-2-phenylacetate (4f):

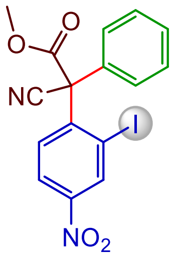

Following the general procedure **1A1** iodonium salt **1a** (101.4 mg, 0.20 mmol, 1.0 equiv), NaH (11.5 mg, 0.48 mmol, 2.4 equiv) and methyl 2-cyanoacetate (25.8 mg or 23 µL, 0.26 mmol, 1.3 equiv) were reacted in DMA (3.0 mL) for 16 h. Product **3w** was isolated via column chromatography (10% ethyl acetate in petroleum ether) as a yellow solid (73.7 mg, 0.18 mmol, 87%); **m.p.** 76.1 °C; $\mathbf{R}_{\boldsymbol{f}}$ 0.20 (10% ethyl acetate in pentane); **^{1}H NMR (400 MHz, $CDCl_3$)** δ 8.85 (d, *J* = 2.4 Hz, 1H), 8.07 (dd, *J* = 8.8, 2.4 Hz, 1H), 7.65 – 7.59 (m, 2H), 7.58 – 7.52 (m, 3H), 6.79 (d, *J* = 8.8 Hz, 1H), 3.93 (s, 3H); **^{13}C NMR (101 MHz, $CDCl_3$)** δ 166.1, 147.7, 145.6, 136.7, 132.0, 131.5, 130.4, 130.1 (2C), 128.3 (2C), 123.1, 116.0, 99.0, 62.3, 54.9; **HRMS** $[M+Na]^+$ calcd. for $C_{16}H_{11}IN_2NaO_4^+$ 444.9656, found 444.9657.

Ethyl 2-cyano-2-(2-iodo-4-nitrophenyl)-2-phenylacetate (4g):

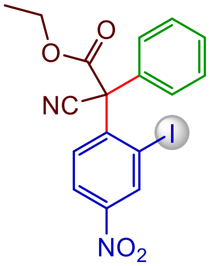

Following the general procedure **1A1** iodonium salt **1a** (98.6 mg, 0.20 mmol, 1.0 equiv), NaH (11.5 mg, 0.48 mmol, 2.4 equiv) and ethyl cyanoacetate (29.4 mg or 28 µL, 0.26 mmol, 1.3 equiv) were reacted in DMA (3.0 mL) for 16 h. Product **4g** was isolated via column chromatography (10% ethyl acetate in petroleum ether) as colorless solid (82.3 mg, 0.19 mmol, 94%); **m.p.** 108.4-109.9 °C; $\mathbf{R}_{\boldsymbol{f}}$ 0.63 (20% ethyl acetate in pentane); **^{1}H NMR (400 MHz, $CDCl_3$)** δ 8.85 (d, *J* = 2.4 Hz, 1H), 8.07 (dd, *J* = 8.8, 2.4 Hz, 1H), 7.64 – 7.59 (m, 2H), 7.57 – 7.52 (m, 3H), 6.78 (d, *J* = 8.8 Hz, 1H), 4.48 – 4.30 (m, 2H), 1.35 (t, *J* = 7.2 Hz, 3H); **^{13}C NMR (101 MHz, $CDCl_3$)** δ 165.5, 147.6, 145.7, 136.7, 132.3, 131.6, 130.3, 130.0 (2C), 128.3 (2C), 123.0, 116.1, 99.0, 64.5, 62.4, 13.9; **HRMS** $[M+Na]^+$ calcd. for $C_{17}H_{13}IN_2NaO_4^+$ 458.9812, found 458.9813.

Diethyl (cyano(2-iodo-4-nitrophenyl)(phenyl)methyl)phosphonate (4h):

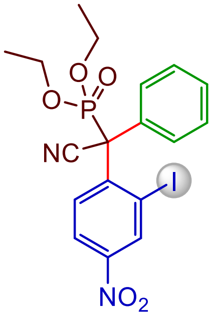

Following the general procedure **1A1** iodonium salt **1a** (98.6 mg, 0.20 mmol, 1.0 equiv), NaH (11.5 mg, 0.48 mmol, 2.4 equiv) and diethyl cyanomethylphosphonate (46.1 mg or 42 µL, 0.26 mmol, 1.3 equiv) were reacted in DMA (3.0 mL) for 16 h. Product **4h** was isolated via column chromatography (20% ethyl acetate in petroleum ether) as white solid (50.8 mg, 0.10 mmol, 51%); **m.p.** 175.8-176.4 °C; $\mathbf{R}_{\boldsymbol{f}}$ 0.23 (50% ethyl acetate in pentane); **^{1}H NMR (400 MHz, $CDCl_3$)** δ 8.77 (dd, *J* = 2.5, 1.2 Hz, 1H), 8.58 (dd, *J* = 8.9, 1.1 Hz, 1H), 8.33 (dd, *J* = 8.9, 2.5 Hz, 1H), 7.39 (d, *J* = 1.1 Hz, 5H), 4.35 (app dq, *J* = 8.4, 7.0 Hz, 2H), 3.81 (app dp, *J* = 10.2, 7.1 Hz, 1H), 3.55 (app tq, *J* = 10.1, 7.1 Hz, 1H), 1.41 (td, *J* = 7.1, 0.7 Hz, 3H), 1.18 (td, *J* = 7.1, 0.9 Hz, 3H); **^{13}C NMR (101 MHz, $CDCl_3$)** δ 147.4, 142.4, 138.1, 132.6 (d, *J* = 5.2 Hz), 131.3 (d, *J* = 8.8 Hz), 130.3 (d, *J* = 4.4 Hz, 2C), 129.1 (d, *J* = 3.2 Hz), 128.8 (d, *J* = 2.9 Hz, 2C), 122.8, 115.8 (d, *J* = 12.2 Hz), 98.9 (d, *J* = 13.5 Hz), 66.6 (d, *J* = 7.6 Hz), 65.8 (d, *J* = 8.7 Hz), 56.4 (d, *J* = 135.4 Hz), 16.4 (d, *J* = 2.3 Hz), 16.4 (d, *J* = 3.2 Hz); **^{31}P NMR (162 MHz, $CDCl_3$)** δ 14.0; **HRMS** $[M+Na]^+$ calcd. for $C_{18}H_{18}IN_2NaO_5P^+$ 522.9890 found 522.9887.

2-Iodo-4-nitro-1-(1-nitro-1-phenylethyl)benzene (4i):

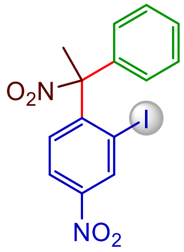

Following the general procedure **1A1** iodonium salt **1a** (107.1 mg, 0.20 mmol, 1.0 equiv), NaH (11.5 mg, 0.48 mmol, 2.4 equiv) and nitroethane (20.8 mg or 20 µL, 0.26 mmol, 1.3 equiv) were reacted in DMA (3.0 mL) for 16 h. Product **4i** was isolated via column chromatography (8% ethyl acetate in petroleum ether) as yellowish solid (33.6 mg, 0.084 mmol, 42%); **m.p.** 98.6-99.4 °C; **R*f*** 0.47 (10% ethyl acetate in pentane); **^{1}H NMR (400 MHz, CDCl$_3$)** δ 8.84 (d, *J* = 2.4 Hz, 1H), 8.12 (dd, *J* = 8.8, 2.4 Hz, 1H), 7.52 – 7.44 (m, 5H), 7.04 (d, *J* = 8.8 Hz, 1H), 2.53 (s, 3H); **^{13}C NMR (101 MHz, CDCl$_3$)** δ 149.2, 147.1, 137.8, 137.0, 130.4, 130.3, 129.3 (2C), 128.3 (2C), 122.9, 98.6, 95.8, 24.5; **HRMS** [M+Na]$^+$ calcd. for $C_{14}H_{11}IN_2NaO_4^+$ 420.9656, found 420.9658.

2-Iodo-4-nitro-1-(1-nitro-1-phenylpropyl)benzene (4j):

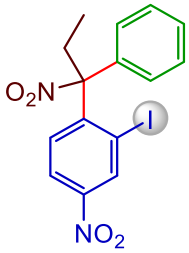

Following the general procedure **1A1** iodonium salt **1a** (98.6 mg, 0.20 mmol, 1.0 equiv), NaH (11.5 mg, 0.48 mmol, 2.4 equiv) and nitropropane (23.2 mg or 23 µL, 0.26 mmol, 1.3 equiv) were reacted in DMA (3.0 mL) for 16 h. Product **4j** was isolated via column chromatography (10% ethyl acetate in petroleum ether) as yellow sticky solid (34.8 mg, 0.084 mmol, 42%); **R*f*** 0.81 (20% ethyl acetate in pentane); **^{1}H NMR (400 MHz, CDCl$_3$)** δ 8.85 (d, *J* = 2.4 Hz, 1H), 8.19 (dd, *J* = 8.8, 2.4 Hz, 1H), 7.53 – 7.48 (m, 2H), 7.46 – 7.39 (m, 3H), 7.20 (d, *J* = 8.8 Hz, 1H), 3.14 (app dp, *J* = 25.2, 7.5 Hz, 2H), 0.82 (t, *J* = 7.4 Hz, 3H); **^{13}C NMR (101 MHz, CDCl$_3$)** δ 147.0, 146.8, 137.8, 136.9, 132.1, 130.1, 129.1 (2C), 129.0 (2C), 122.3, 102.4, 96.3, 28.0, 10.3; **HRMS:** [M+Na]$^+$ calcd. for $C_{15}H_{13}IN_2NaO_4^+$ 434.9812, found 434.9813.

Diethyl 2-(2-hydroxy-5-nitrophenyl)-2-(2-iodo-4-nitrophenyl)malonate (6a):

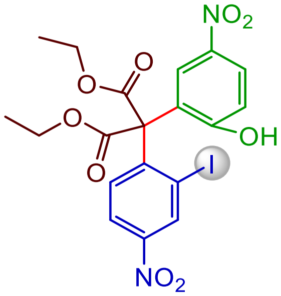

Following the general procedure **1B** iodonium ylide **5a** (84.7 mg, 0.20 mmol, 1.0 equiv, 95% purity), NaH (11.5 mg, 0.48 mmol, 2.4 equiv) and diethyl malonate (41.6 mg or 39 µL, 0.26 mmol, 1.3 equiv) were reacted in DMA (3.0 mL) for 16 h. Product **6a** was isolated via column chromatography (20% ethyl acetate in petroleum ether) as light yellow oil (77.6 mg, 0.14 mmol, 71%); **R*f*** 0.87 (50% ethyl acetate in pentane); **^{1}H NMR (400 MHz, CDCl$_3$)** δ 8.79 (d, *J* = 2.6 Hz, 1H), 8.22 (dd, *J* = 9.0, 2.7 Hz, 1H), 8.13 (dd, *J* = 8.6, 2.2 Hz, 1H), 7.80 (d, *J* = 8.6 Hz, 1H), 7.69 (d, *J* = 2.2 Hz, 1H), 6.95 (d, *J* = 9.0 Hz, 1H), 5.11 (s, 1H), 4.33 – 4.15 (m, 4H), 1.26 (t, *J* = 7.1 Hz, 6H); **^{13}C NMR (101 MHz, CDCl$_3$)** δ 166.8 (2C), 160.5, 153.8, 148.8, 144.7, 136.0, 132.3, 132.0, 125.8, 120.1, 117.8, 113.7, 87.4, 62.7 (2C), 51.8, 14.2 (2C); **HRMS** [M+Na]$^+$ calcd. for $C_{19}H_{17}IN_2NaO_9^+$ 566.9871, found 566.9873.

Dimethyl 2-(2-hydroxy-5-nitrophenyl)-2-(2-iodo-4-nitrophenyl)malonate (6b):

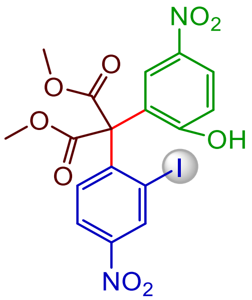

Following the general procedure **1B** iodonium ylide **5a** (84.7 mg, 0.20 mmol, 1.0 equiv, 95% purity), NaH (11.5 mg, 0.48 mmol, 2.4 equiv) and dimethyl malonate (34.3 mg or 30 µL, 0.26 mmol, 1.3 equiv) were reacted in DMA (3.0 mL) for 16 h. Product **6b** was isolated via column chromatography (20% ethyl acetate in petroleum ether) as light yellow oil (92.2 mg, 0.18 mmol, 89%); **R*f*** 0.67 (50% ethyl acetate in pentane); **^{1}H NMR (400 MHz, $CDCl_3$)** δ 8.79 (d, *J* = 2.7 Hz, 1H), 8.23 (dd, *J* = 9.0, 2.7 Hz, 1H), 8.12 (dd, *J* = 8.6, 2.3 Hz, 1H), 7.78 (d, *J* = 8.6 Hz, 1H), 7.67 (d, *J* = 2.3 Hz, 1H), 6.97 (d, *J* = 9.0 Hz, 1H), 5.14 (s, 1H), 3.79 (s, 6H); **^{13}C NMR (101 MHz, $CDCl_3$)** δ 167.2 (2C), 160.3, 153.9, 148.9, 144.8, 136.0, 132.3, 131.6, 125.8, 120.0, 118.0, 113.6, 87.6, 53.6 (2C), 51.5; **HRMS** $[M+Na]^+$ calcd. for $C_{17}H_{13}IN_2NaO_9^+$ 538.9558, found 538.9554.

1-Ethyl 3-methyl 2-(2-hydroxy-5-nitrophenyl)-2-(2-iodo-4-nitrophenyl)malonate (6c):

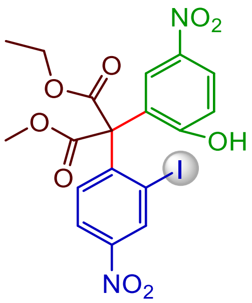

Following the general procedure **1B** iodonium ylide **5a** (84.7 mg, 0.20 mmol, 1.0 equiv, 95% purity), NaH (11.5 mg, 0.48 mmol, 2.4 equiv) and ethyl methyl malonate (38.0 mg, 0.26 mmol, 1.3 equiv) were reacted in DMA (3.0 mL) for 16 h. Product **6c** was isolated via column chromatography (20% ethyl acetate in petroleum ether) as light yellow oil (88.5 mg, 0.17 mmol, 84%); **R*f*** 0.77 (50% ethyl acetate in pentane);**^{1}H NMR (400 MHz, $CDCl_3$)** δ 8.79 (d, *J* = 2.7 Hz, 1H), 8.22 (dd, *J* = 9.0, 2.7 Hz, 1H), 8.12 (dd, *J* = 8.6, 2.3 Hz, 1H), 7.79 (d, *J* = 8.6 Hz, 1H), 7.68 (d, *J* = 2.2 Hz, 1H), 6.96 (d, *J* = 9.0 Hz, 1H), 5.12 (s, 1H), 4.33 – 4.15 (m, 2H), 3.79 (s, 3H), 1.26 (t, *J* = 7.1 Hz, 3H); **^{13}C NMR (101 MHz, $CDCl_3$)** δ 167.3, 166.7, 160.4, 153.9, 148.8, 144.7, 136.0, 132.3, 131.8, 125.8, 120.1, 117.9, 113.6, 87.5, 62.8, 53.5, 51.7, 14.2; **HRMS** $[M+Na]^+$ calcd. for $C_{18}H_{15}IN_2NaO_9^+$ 552.9714, found 552.9712.

3.2 Large scale synthesis

Synthesis of diethyl 2-(2-iodo-4-nitrophenyl)-2-phenylmalonate (3a):

Diethyl malonate (888 µL, 5.85 mmol, 1.3 equiv) was added into a solution of NaH (259 mg, 10.8 mmol, 2.4 equiv) in dry DMA (7 mL) at 0 °C over 10 min under nitrogen atmosphere. After additional 10 min at 0 °C the solution was stirred for 15 min at room temperature. Under nitrogen atmosphere the solution of deprotonated malonate was added to a solution of iodonium salt (2.22 g, 4.5 mmol, 1.0 equiv) in DMA (15 mL) at 0 °C over 30 min via syringe. The vessel of deprotonated malonate was rinsed with additional DMA (7 mL) and the solution was added at once to the iodonium salt solution. The reaction was allowed to reach room temperature and stirred for 16 h. EtOAc (50 mL) was added to prevent the product from crashing out and water (10 mL) was added. The organic phase was washed with water (2×100 mL). The combined aqueous phases were extracted with EtOAc (50 mL). The combined organic phases were dried over Na_2SO_4, filtered and adsorbed onto silica gel. The product was purified by column chromatography (pentane: EtOAc, 10:1 to 5:1) to give **diarylated product 3a** (1.945 g, 4.025 mmol, 90%) as a light-yellow solid. *(Caution! NaH should be handled carefully during weighing as well as quenching.)*

Synthesis of methyl 2-cyano-2-(2-iodo-4-nitrophenyl)-2-phenylacetate (4f):

Methyl cyanoacetate (170 µL, 1.93 mmol, 1.3 equiv) was added dropwise into a solution of NaH (81.8 mg, 3.4 mmol, 2.3 equiv) in dry DMA (5 mL) at 0 °C under nitrogen atmosphere. After additional 30 min, the solution of deprotonated malonate was added to a solution of iodonium salt (2.22 g, 4.5 mmol, 1.0 equiv) in DMA (7 mL) at 0 °C over 30 min under N_2 atmosphere. The vessel of deprotonated cyanoacetate was rinsed with additional DMA (2 mL) and the solution was added at once to the iodonium salt solution. The reaction was allowed to reach room temperature and stirred for 16 h. EtOAc (50 mL) was added to prevent the product from crashing out and water (10 mL) was added. The organic phase was washed with water (2×100 mL). The combined aqueous phases were extracted with EtOAc (50 mL). The combined organic phases were dried over Na_2SO_4, filtered and adsorbed onto silica gel. The product was purified by column chromatography (pentane:EtOAc, 10:1 to 5:1) to give **diarylated product 4f** (567.2 mg, 1.34 mmol, 91%) as a light yellow solid. *(Caution! NaH should be handled carefully during weighing as well as quenching.)*

4 Mechanistic investigations

4.1 Mechanistic hypothesis

Literature precedent of established reactivity of fluorinated diaryliodonium salts shows S_NAr as the first step, followed by an intramolecular aryl transfer.[2-4] If these precedents were applied to the reaction of salt **1a** with malonate **2a**, then initial enolate formation should be followed by S_NAr, which would yield intermediate **II** (Scheme S5, Pathway 1). In the presence of NaH, **II** should be easily deprotonated to form intermediate **III**, which likely has the enolate oxygen bound to iodine via the hypervalent iodine bond. The T-shaped structure of intermediate **III** is supported by the recently published crystal structure of VBX[6] benziodoxolone with a six-membered side arm. In this structure, a distorted 6-membered ring is formed in favour of the T-shape iodine(III) moiety (Figure S2).[5]

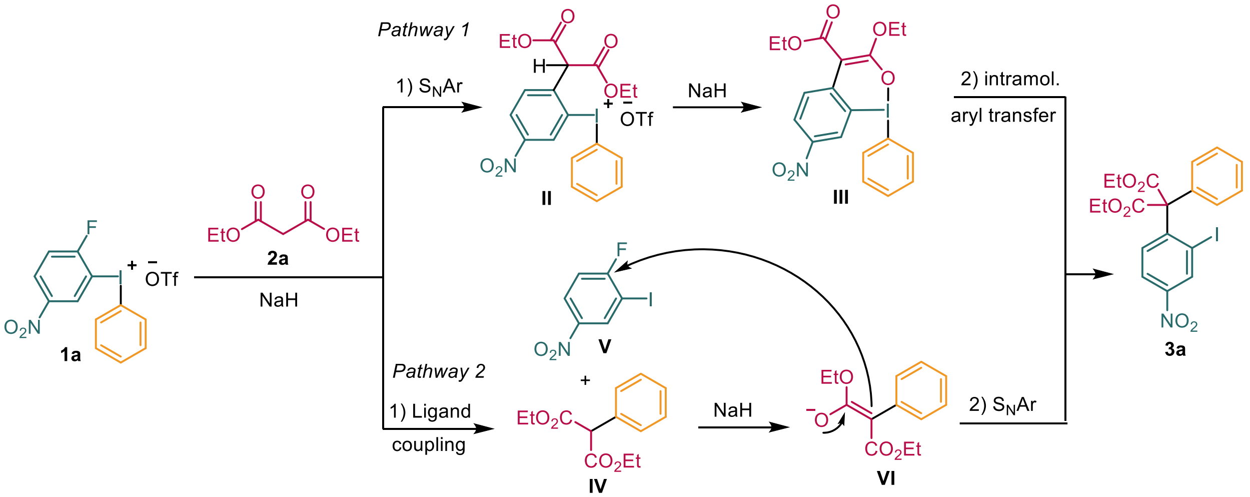

Scheme S5: Possible mechanistic pathways for the diarylation of malonates.

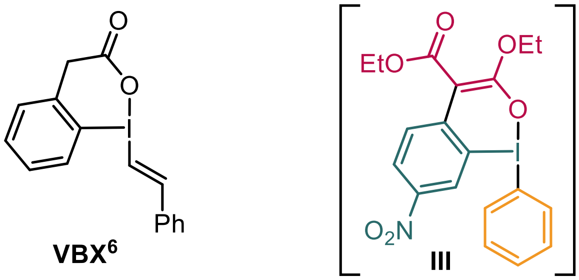

Figure S2: Structural comparison of the 6-membered VBX and S_NAr intermediate III.

Alternatively, the reaction could proceed through initial ligand coupling, followed by an intermolecular S_NAr (Pathway 2). In this case, the formed enolate would undergo ligand exchange to a T-shaped intermediate, followed by ligand coupling with the more electron rich aryl group to give phenyl malonate **IV** and fluoroarene **V**. This chemoselectivity would be expected to follow the observed *anti-ortho effect* previously reported for α-arylation of malonates.[6] The second arylation would then proceed through deprotonation of **IV** to yield enolate **VI**, followed by S_NAr with **V** to give product **3a**.

4.2 Investigation of the S_NAr in pathway 2

The feasibility of the suggested S_NAr reaction between enolate **VI** and fluoroarene **V** was evaluated under our reaction conditions (Scheme S6). Hence commercially available diethyl phenymalonate (**IV**) was deprotonated with sodium hydride to give enolate **VI**, before addition of fluoroarene **V**. The reaction was quenched after 16 h and TMB was added as internal standard. Only starting material was observed in the crude mixture. The formation of **3a** was not observed at 20 °C, which disproves pathway 2.

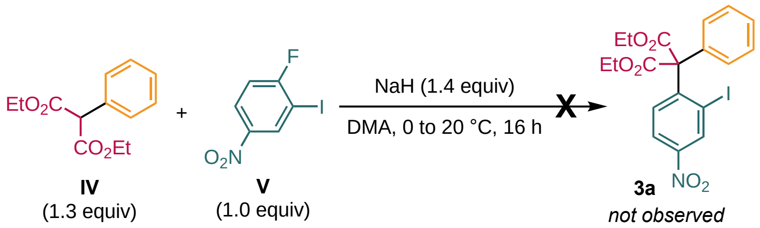

Scheme S6: S_NAr reaction to evaluate the mechanism via pathway 2.

To observe any short-lived intermediates in the reaction, we monitored the reaction via NMR-spectroscopy at low temperatures. The diarylation of **2a** with **1a** was set up under modified conditions to allow *in situ* ^{1}H-NMR measurements in DMA (Scheme S7). **Vial 1** was charged with pentane-washed, vacuum-dried NaH (5.3 mg, 0.22 mmol, 2.2 equiv), then non-deuterated DMA (0.3 mL) was added, followed by addition of **2a** (27 µL, 0.26 mmol, 2.6 equiv). Then the resulting solution was stirred for 30 min at room temperature for complete enolate formation. **Vial 2** was charged with salt **1a** (49.3 mg, 0.10 mmol, 1.0 equiv) dissolved in DMA (0.3 mL). To ensure that the reaction would not take place upon mixing, the two solutions were frozen in alternating layers of 0.1 mL aliquots in an NMR tube (that was precooled to 223 K), making sure that each layer was frozen before addition of the next aliquot. In the NMR spectrometer, the NMR sample was allowed to thaw at 256 K, just above the melting point of DMA (253 K).

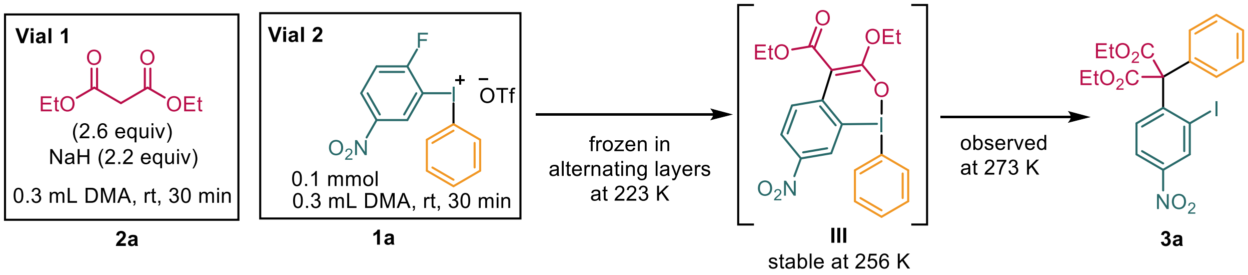

Scheme S7: Reaction setup and intermediate III.

Figure S3 shows the comparison of the respective ^{1}H-NMR spectra all measured at 256 K in DMA: **i)** iodonium salt **1a**, **ii)** the reaction solution, and **iii)** diarylated malonate **3a**. The signals of **1a** at 8.7 ppm (dd) and 9.8 ppm (ddd) have a distinctive multiplicity because of the fluorine substitution of the aryl ring. Interestingly, spectrum ii) contains a set of signals that does not correlate to spectrum i) or spectrum iii). This indicates that an unknown intermediate **III**, only stable at this lower temperature, has been formed. Since the multiplicities of **III**'s signals lack indications of fluorine coupling, it can be presumed that the fluoride substitution has already taken place. This is supported by comparing the ^{19}F-NMR spectra **iv)** of **1a** and the reaction mixture **v)** at 256 K, where the ArF signal is not observed anymore and only the OTf signal remains (Figure S4).

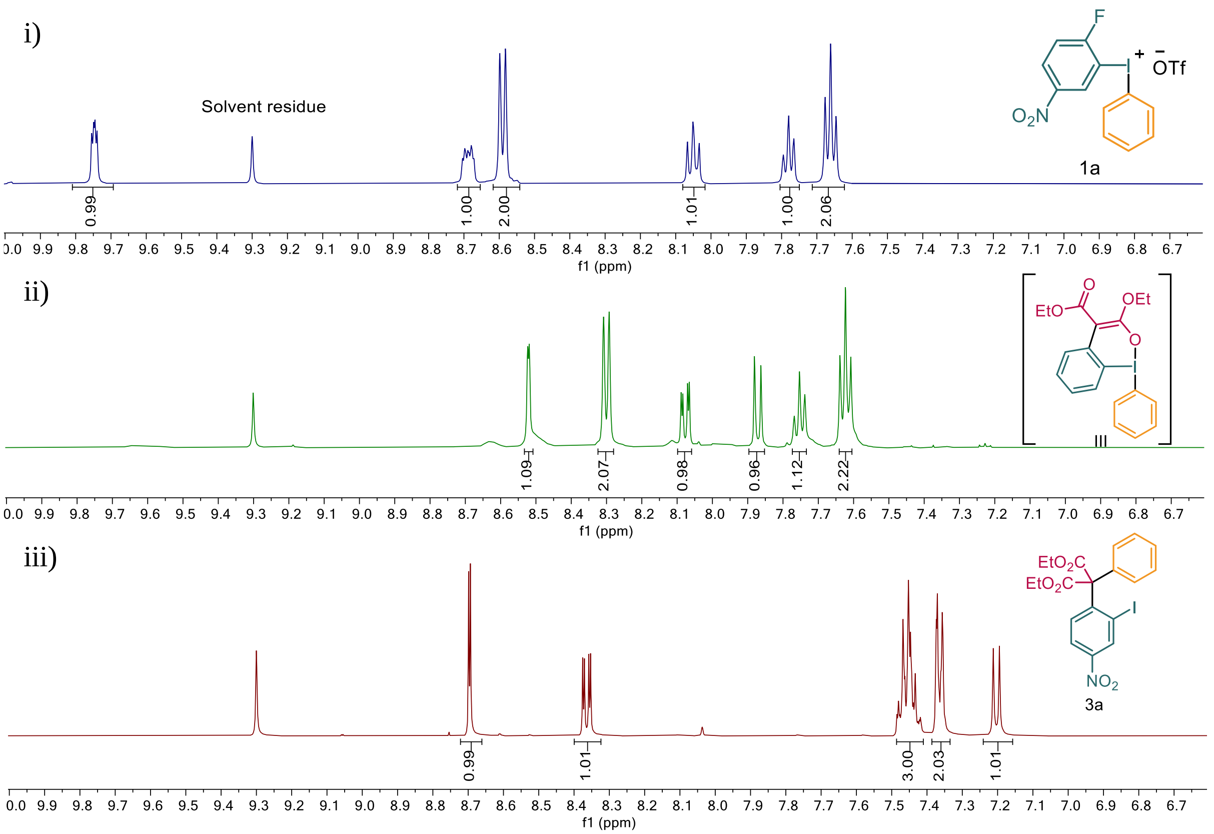

Figure S3: Excerpt of the ¹H-NMR spectra of: i) 1a, ii) the reaction solution and iii) 3a all measured at 256 K. The singlet at 9.30 ppm originates from DMA.

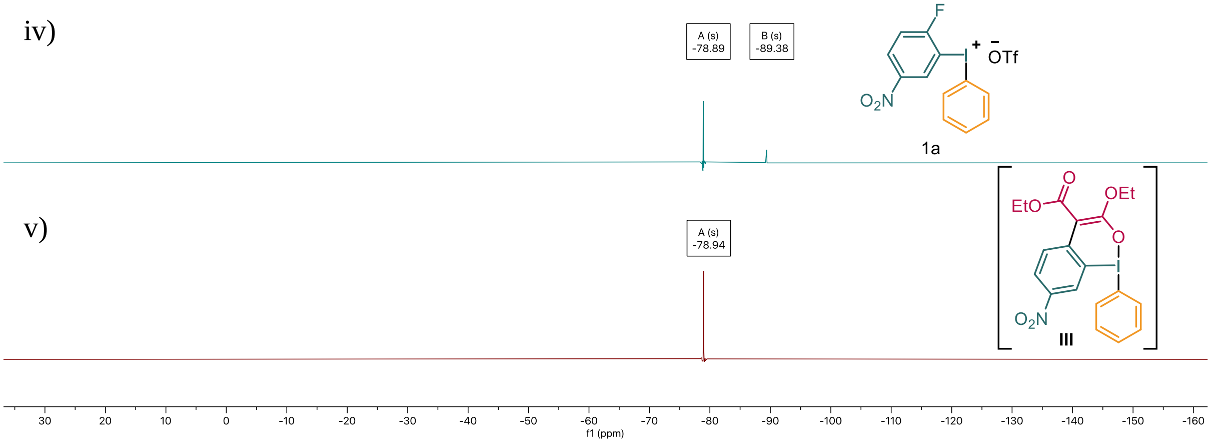

Figure S4: ¹⁹F-NMR spectra of iv) 1a and v) the reaction solution at 256 K.

Figure S5 indicates that intermediate **III** is stable at 256 K for at least 30 min.

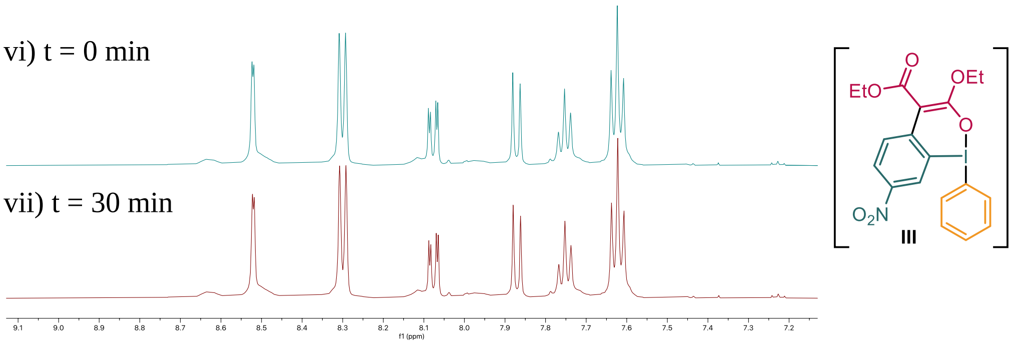

Figure S5: Excerpt of the ¹H-NMR-spectra of vi) reaction solution at 256 K and vii) after 30 min.

Upon raising the temperature to 273 K, the transformation of intermediate **III** to product **3a** occurred. The temperature was kept at 273 K for 60 min, during which the **III**'s signals decreased and **3a**'s increased (Figure S6). When raising the temperature to 298 K, the signals of the **III** almost completely disappeared (Figure S7).

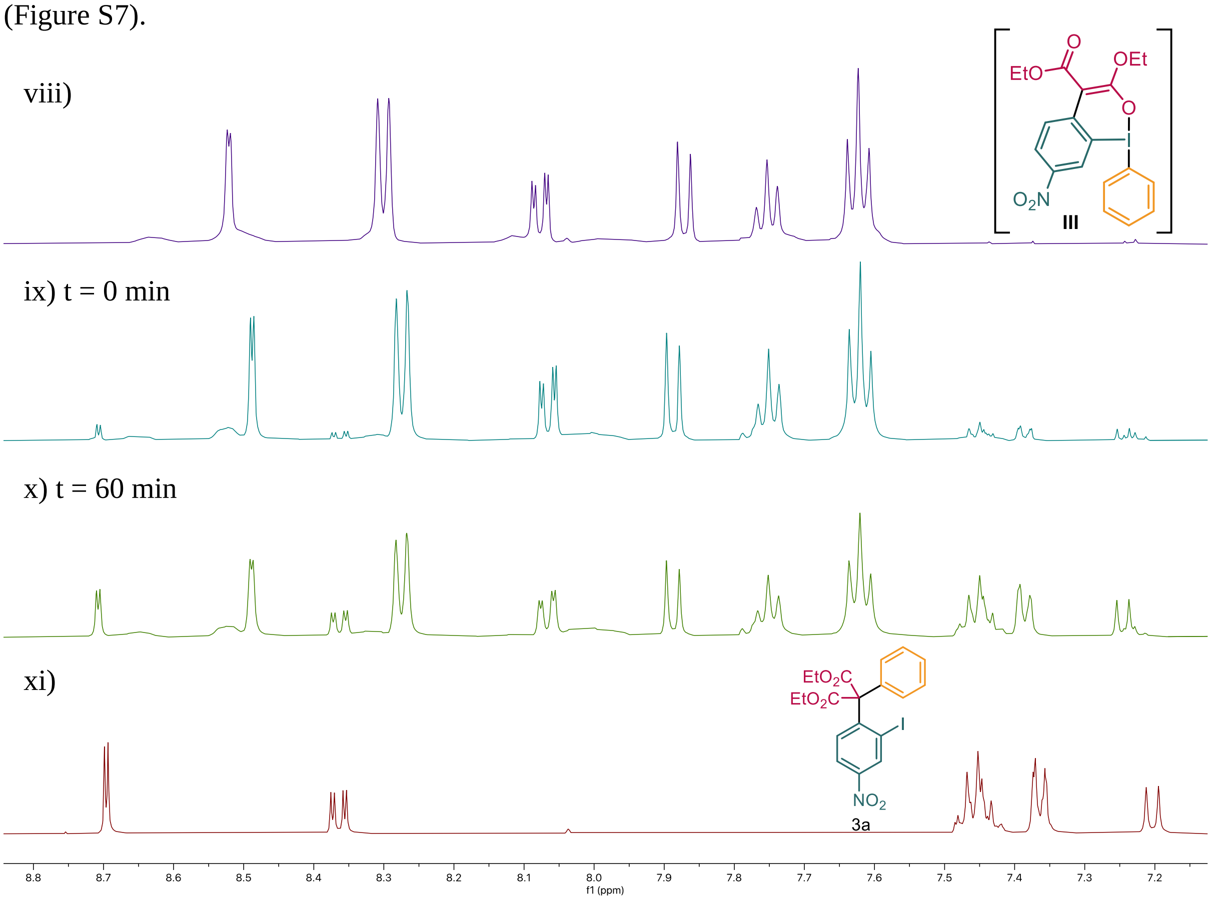

Figure S6: Excerpt of the ¹H-NMR-spectra viii) of intermediate III at 256 K for comparison and ix) the reaction solution measured when reaching 273 K and x) keeping it at 273 K for 60 min. Excerpt of the ¹H-NMR-spectrum xi) 3a at 256 K for comparison.

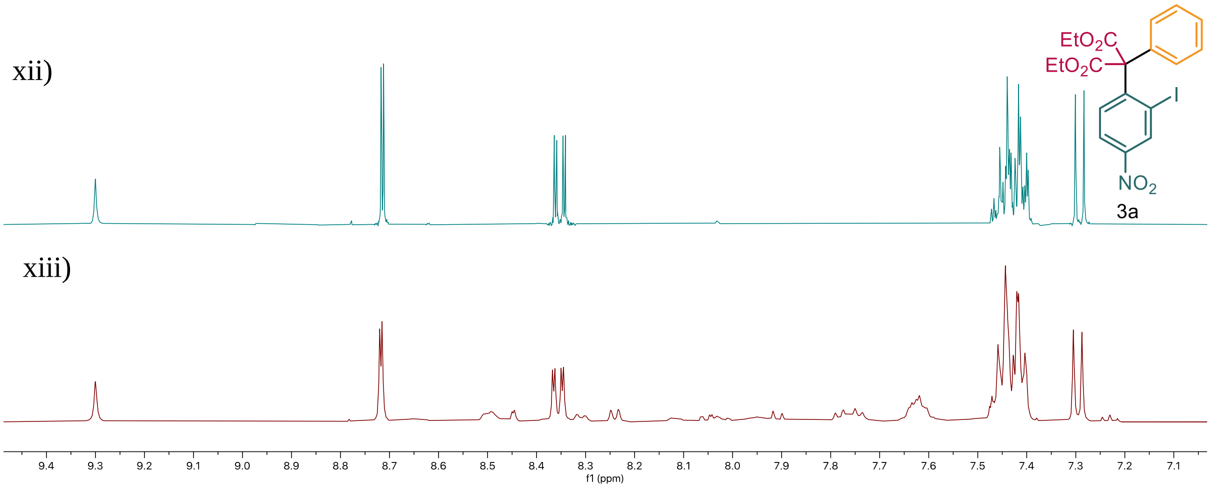

Figure S7: Comparison of the excerpt of ^{1}H-NMR spectra xii) 3a and xiii) reaction solution at 298 K in DMA. The singlet at 9.30 ppm originates from DMA.

4.3 Characterization and trapping of intermediates

Below ^{1}H-NMR data of salt **1a**, product **3a** and intermediate **III** in DMA shown in Figure *S3*-*S7* at different temperatures are listed, providing strong support to mechanistic pathway 1.

Iodonium salt 1a

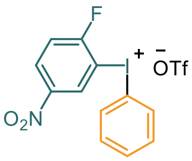

1a

^{1}H NMR (500 MHz, DMA, 298 K) δ 9.65 (dd, *J* = 4.9, 2.8 Hz, 1H), 8.68 (ddd, *J* = 9.1, 4.5, 2.8 Hz, 1H), 8.54 (d, *J* = 7.9 Hz, 2H), 8.01 (dd, *J* = 9.2, 7.6 Hz, 1H), 7.78 (t, *J* = 7.5 Hz, 1H), 7.66 (t, *J* = 7.9 Hz, 2H).

^{1}H NMR (500 MHz, DMA, 256 K) δ 9.75 (dd, *J* = 4.9, 2.8 Hz, 1H), 8.69 (dt, *J* = 8.9, 3.2 Hz, 1H), 8.59 (d, *J* = 7.9 Hz, 2H), 8.05 (dd, *J* = 9.1, 7.5 Hz, 1H), 7.78 (t, *J* = 7.4 Hz, 1H), 7.66 (t, *J* = 7.8 Hz, 2H). **^{19}F NMR (471 MHz, DMA, 256 K)** δ –78.89, –89.38.

Product 3a

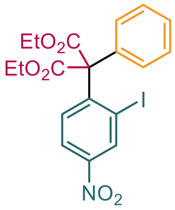

3a

^{1}H NMR (500 MHz, DMA, 298 K) δ 8.71 (d, *J* = 2.5 Hz, 1H), 8.35 (dd, *J* = 8.7, 2.5 Hz, 1H), 7.48 – 7.39 (m, 5H), 7.29 (d, *J* = 8.8 Hz, 1H), 4.32 (app qd, *J* = 7.1, 1.3 Hz, 4H), 1.24 (t, *J* = 7.1 Hz, 6H).

^{1}H NMR (500 MHz, DMA, 256 K) δ 8.70 (d, *J* = 2.5 Hz, 1H), 8.36 (dd, *J* = 8.7, 2.5 Hz, 1H), 7.49 – 7.42 (m, 3H), 7.39 – 7.35 (m, 2H), 7.20 (d, *J* = 8.7 Hz, 1H), 4.29 (app qd, *J* = 7.1, 1.8 Hz, 4H), 1.21 (t, *J* = 7.1 Hz, 6H).

Intermediate III

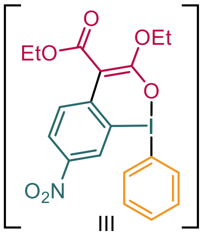

^{1}H NMR (500 MHz, DMA, 256 K) δ 8.52 (d, *J* = 2.5 Hz, 1H), 8.30 (d, *J* = 7.5 Hz, 2H), 8.08 (dd, *J* = 9.1, 2.5 Hz, 1H), 7.87 (d, *J* = 9.1 Hz, 1H), 7.75 (t, *J* = 7.5 Hz, 1H), 7.62 (t, *J* = 7.6 Hz, 2H), 4.07 (app t, *J* = 7.1 Hz, 4H), 1.25 – 1.21 (m, 6H); ethyl groups of **III** overlap with signals

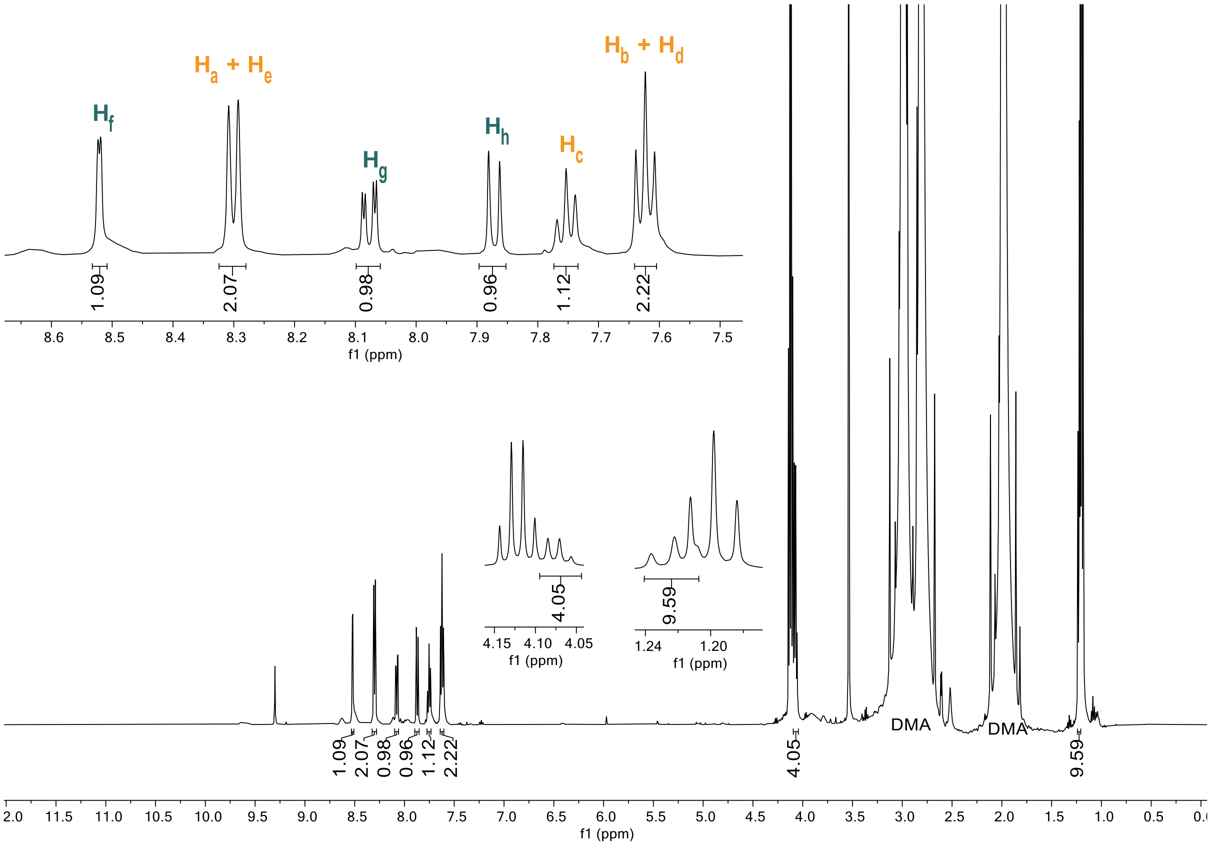

of ethyl groups of diethyl malonate (excess starting material) (

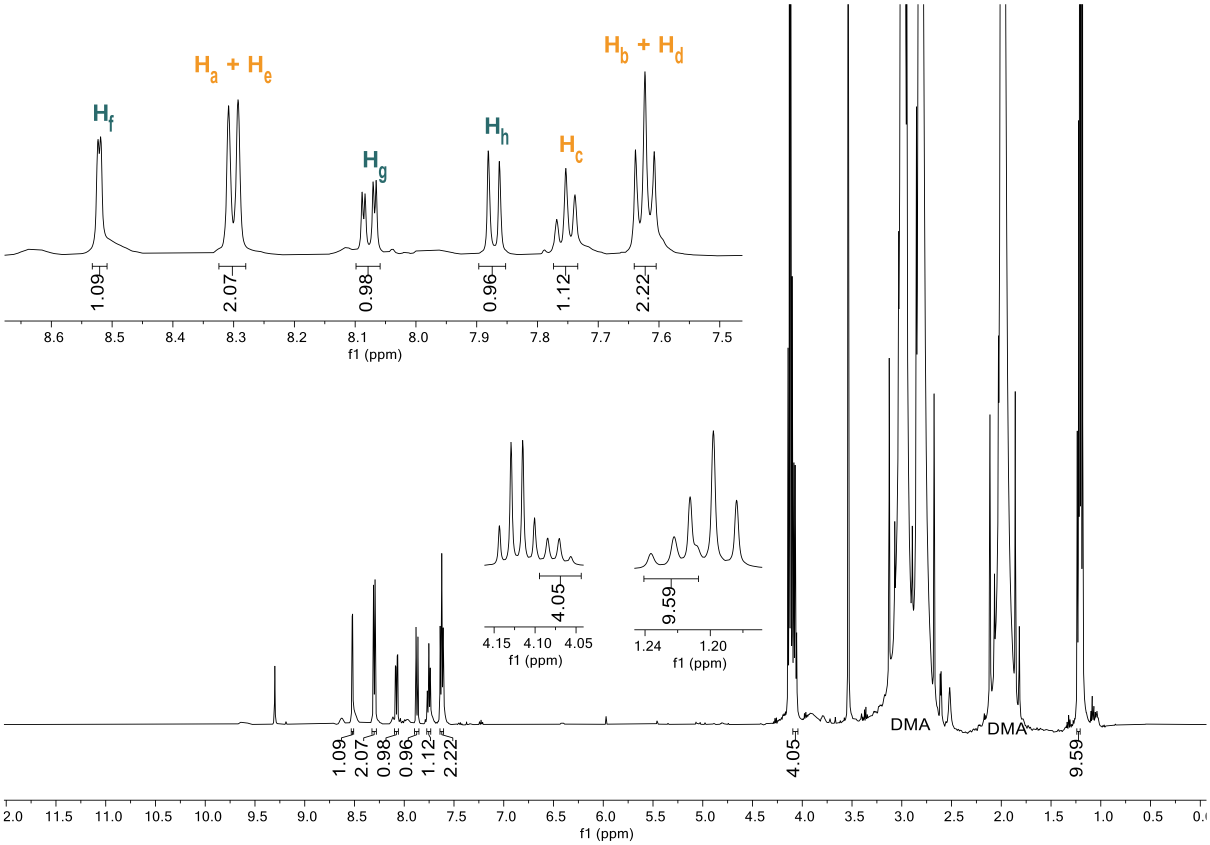

Figure S8).

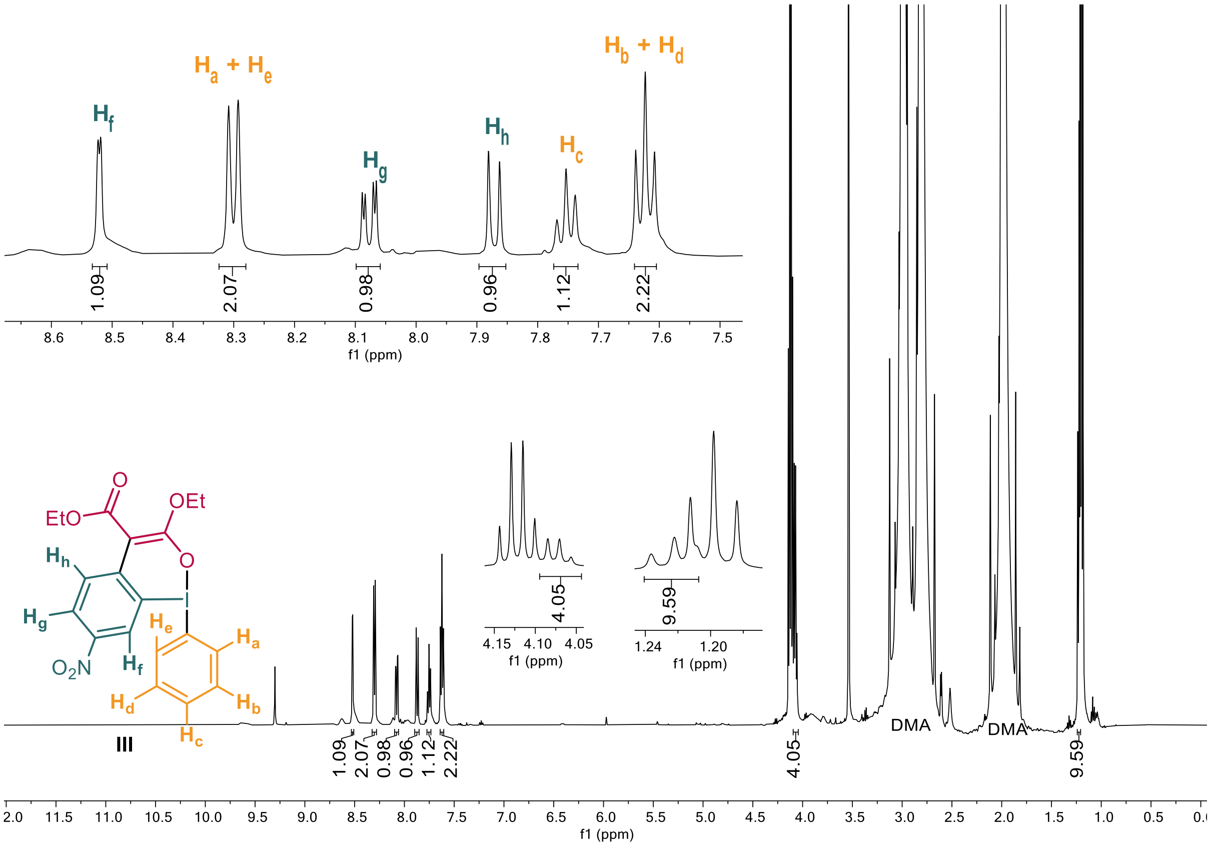

Figure S8: Labelled and tabulated ^{1}H-NMR of intermediate III.

Trapping of intermediate II

To enable trapping of the reaction intermediates, the protocol was adjusted to halt the reaction after formation of the S_NAr intermediate **III**, which upon HCl quench would yield malonate-substituted iodonium salt **II**. Hence, the reaction procedure applied in the NMR experiments was utilized for a reaction in a vail (Scheme S8). Vial 1 was charged with pentane-washed, dried sodium hydride (10.6 mg, 0.44 mmol, 2.2 equiv) and DMA (1.0 mL), followed by **2a** (39 µL, 0.26 mmol, 1.3 equiv). The resulting solution was stirred for 30 min for complete enolate formation at room temperature. To prevent the immediate reaction, the enolate solution was frozen in liquid nitrogen, before iodonium salt **1a** solution was then added.

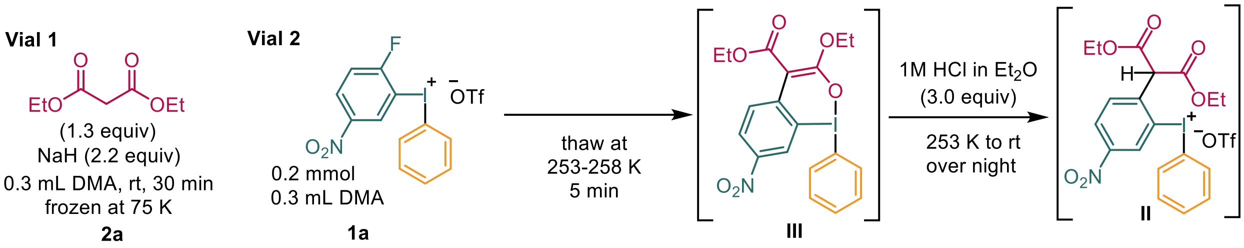

Scheme S8: Reaction setup to trap intermediate II.

In vial 2, iodonium salt **1a** (98.6 mg, 0.20 mmol, 1.0 equiv) was dissolved in DMA (1.0 mL) and subsequently layered on top of the frozen enolate solution and cooled to 75 K.

The reaction mixture was thawed between 253-258 K and allowed to stir for 5 min, before 1 M HCl in diethyl ether (0.3 mL, 0.3 mmol, 1.5 equiv) was added. The resulting mixture was stirred at 253-258 K and then allowed to reach rt overnight. The solvent was removed under reduced pressure to give intermediate **II**. A crude ^{1}H-NMR confirmed the formation of **II** mixed with remaining starting material **1a**. Unfortunately, the attempt to obtain a pure sample of **II** was unsuccessful due to complete transformation of **II** to diarylated product **3a** on a silica gel column.

Nevertheless, attempts at acquiring HRMS data of intermediate **II** ($[M_{iodonium}–OTf]^+$) were successful. The ESI(+) spectrum **xiv)** of crude mixture **II** and **xv) 3a** show different species. While HRMS spectrum **xiv)** displays the signal for $[M_{iodonium}–OTf]^+$, which is characteristic for diaryliodonium salts, HRMS spectrum **xv)** does not display the same characteristic mass signal, which would correlate to protonated species $[M_{diarylation}+H]^+$, but only displays the $[M_{diarylation}+Na]^+$ signal (Figure S9).

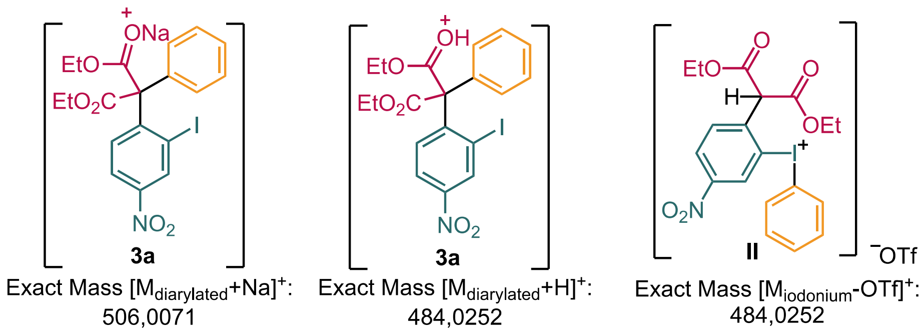

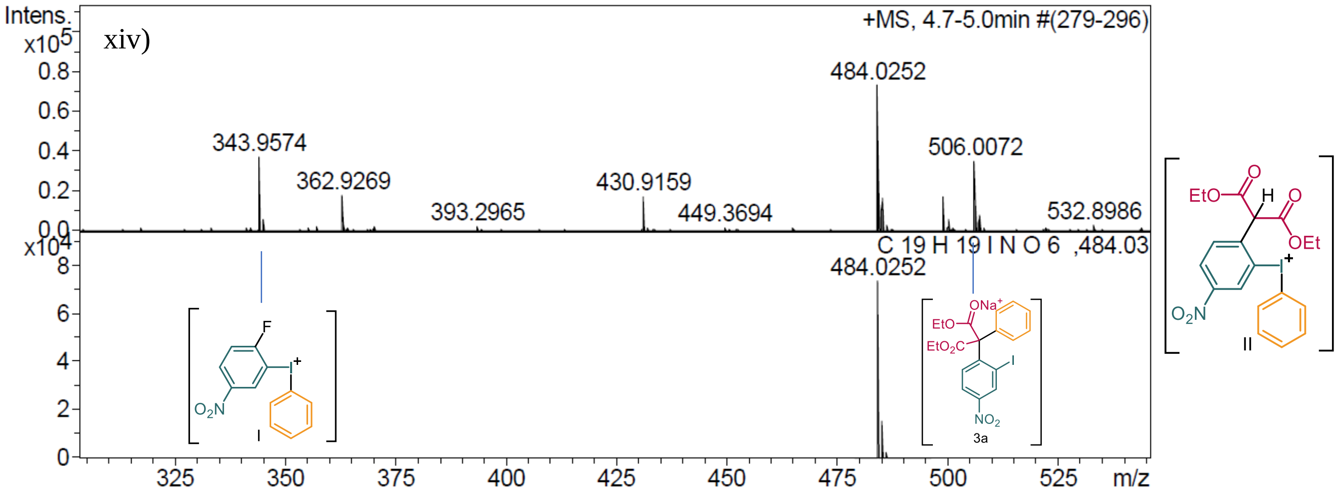

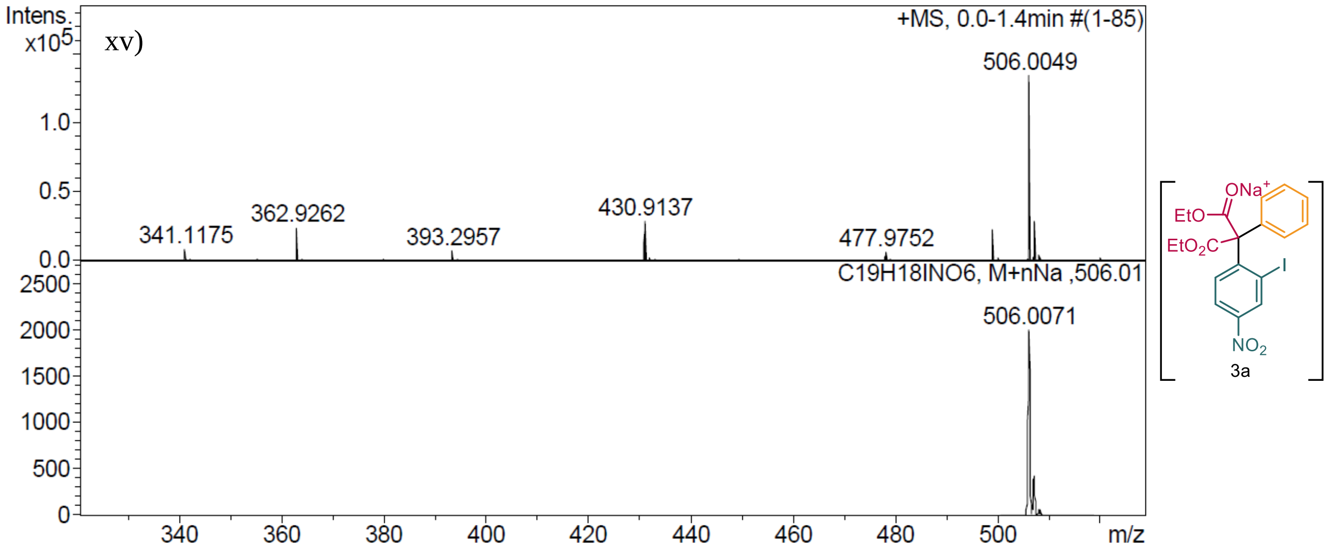

Figure S9: Comparison of the ESI(+)-HRMS spectra of xiv) crude, quenched reaction mixture containing intermediate II, and xv) 3a.

HRMS [$M_{iodonium}$-OTf]$^+$ calcd. for $C_{19}H_{19}INO_6^+$ 484.0252, found 484.0252.

4.4 Intramolecular aryl transfer

Generally, metal-free arylations with diaryliodonium salts proceed by coordination of the nucleophile to the iodine(III) center, forming a three-coordinated T-shaped intermediate (Scheme S9A). The reaction continues with a ligand coupling, whereby the equatorial aryl group is transferred to the nucleophile, and the other aryl group is eliminated as iodoarene, which is the driving force of the reaction.

We have previously studied the ligand coupling mechanism with enolates and malonates with DFT, and found that the favored reaction pathway goes through an O-I intermediate, which undergoes an "extended" ligand coupling through a [2,3]-rearrangement (Scheme S9B).[6-7]

In the diarylation reactions, the second arylation is intramolecular, which imposes steric restrictions that might alter the preferred pathway. In our previously reported S-diarylation, DFT-calculations showed a preferred pathway through a five-membered TS where the nucleophile is not bound to the iodine, and instead attacks the ipso-carbon of the second aryl group, with simultaneous breaking of the C-I bond (Scheme S9C).

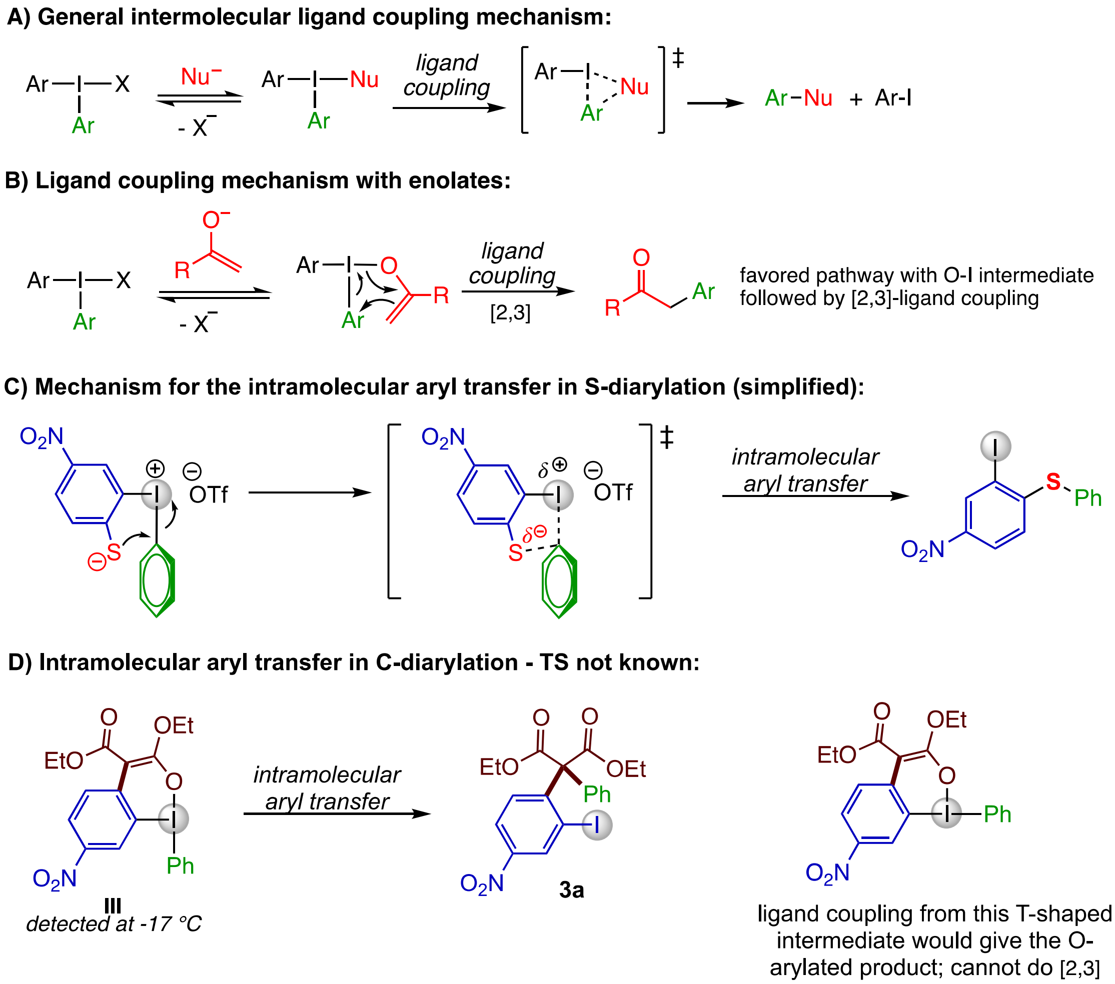

Scheme S9: The intramolecular aryl transfer.

We have not studied the C-diarylation with DFT, and hence refrain from suggesting an exact pathway from intermediate **III** to **3a** (Scheme S9D). The O-I bond in **III** could break to allow for rotation and arylation

through a TS similar to the S-diarylation. Note that isomerization[8-9] of **III** to a T-shaped I-O intermediate with the two aryl groups in the hypervalent bond would give the O-arylated product through ligand coupling (not observed). The [2,3]-pathway shown in Scheme S9B would not work in this case, as the enolate carbon cannot come close enough to the phenyl group.

5 Post-Synthetic transformations

5.1 Reductions and cyclizations

Reduction of the nitro group to give diethyl 2-(4-amino-2-iodophenyl)-2-phenylmalonate (7a):

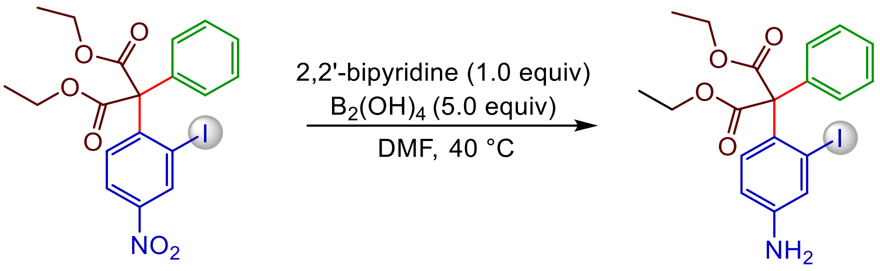

Following a literature procedure,[10] diarylmalonate **3a** (48.3 mg, 0.10 mmol, 1.0 equiv) and 2,2'-bipyridine (15.6 mg, 0.10 mmol, 1.0 equiv) were added to an vial followed by addition of DMF (1.0 mL) and $B_2(OH)_4$ (44.8 mg, 0.50 mmol, 1.0 equiv) and the reaction mixture was allowed to stir for 4 h at 40 °C. After that, the reaction mixture was diluted with water (10 mL) and EtOAc (20 mL), the organic phase was separated and amine **8a** was isolated via column chromatography (20% ethyl acetate in petroleum ether) as colorless viscous oil (43.8 mg, 0.10 mmol, 97%); $\mathbf{R_f}$ 0.47 (50% ethyl acetate in pentane); **m.p.** 142.8-143.7 °C; **^{1}H NMR (400 MHz, $CDCl_3$)** δ 7.45 – 7.39 (m, 2H), 7.34 – 7.29 (m, 4H), 6.77 (d, *J* = 8.5 Hz, 1H), 6.58 (dd, *J* = 8.5, 2.5 Hz, 1H), 4.29 (q, *J* = 7.1 Hz, 4H), 3.68 (s, 2H), 1.26 (t, *J* = 7.1 Hz, 6H); **^{13}C NMR (101 MHz, $CDCl_3$)** δ 169.6 (2C), 146.3, 138.0, 131.7, 131.0, 129.7 (2C), 128.2 (2C), 128.0, 127.7, 114.0, 101.8, 71.8, 62.5 (2C), 14.0 (2C); **HRMS** $[M+Na]^+$ calcd. for $C_{19}H_{20}INNaO_4^+$ 476.0329, found 476.0332.

Decarboxylation of the ester group to give ethyl 2-(2-iodo-4-nitrophenyl)-2-phenylacetate (7b):

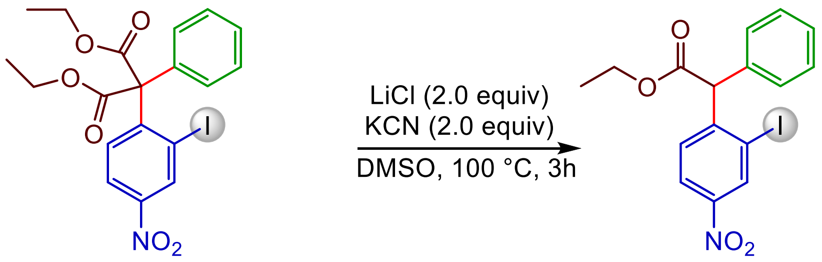

Compound **8e** was prepared following a modified procedure from Krapcho and co-workers.[11] Diarylated malonate (48.3 mg, 0.1 mmol, 1.0 equiv) were dissolved in DMSO (0.3 mL). LiCl (8.5 mg, 0.2 mmol, 2.0 equiv) and water (2 µL, 0.1 mmol), KCN (13 mg, 0.2 mmol, 2.0 equiv) were added and the reaction was heated to 100 °C for 3 h. The color was changed to violet immediately by addition of KCN. Extraction of the reaction mixture with EtOAc and brine. The reaction was quenched by addition of water (2 mL). Brine (10 mL) was added and the aqueous phase was extracted with EtOAc (3x10 mL) before the combined organic layers were dried over Na_2SO_4. After filtration and evaporation of solvent, the residue was purified by column chromatography (5% ethyl acetate in petroleum ether) to isolate ethyl 2-(2-iodo-4-nitrophenyl)-2-phenylacetate (37.2 mg, 0.09 mmol, 90%) as yellow oil. $\mathbf{R_f}$ = 0.32 (5% ethyl acetate in petroleum ether); **^{1}H NMR (400 MHz, $CDCl_3$)** δ 8.71 (d, *J* = 2.4 Hz, 1H), 8.13 (dd, *J* = 8.7, 2.4 Hz, 1H), 7.42 – 7.31 (m, 4H), 7.30 – 7.24 (m, 2H), 5.43 (s, 1H), 4.32 – 4.19 (m, 2H), 1.28 (t, *J* = 7.1 Hz, 3H); **^{13}C NMR (101 MHz,**

CDCl$_3$) δ 170.9, 148.8, 147.0, 136.7, 134.6, 130.5, 129.3 (2C), 128.9 (2C), 128.2, 123.2, 100.7, 62.1, 61.1, 14.3; **HRMS** $[M+Na]^+$ $C_{16}H_{14}INNaO_4^+$: 433.9860; found: 433.9843.

Pd-catalyzed cyclization to give diethyl 3-nitro-9H-fluorene-9,9-dicarboxylate (7c):

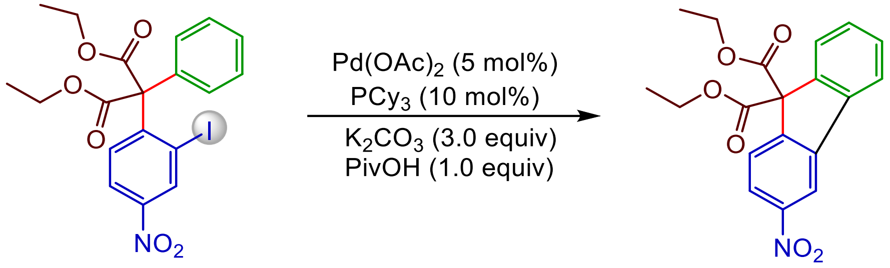

3a **7c**

Following a modified procedure,[12] PCy_3 (5.6 mg, 0.02 mmol, 10 mol%) was added to an oven dried microwave vial containing $Pd(OAc)_2$ (2.2 mg, 0.01 mmol, 5 mol%), K_2CO_3 (82.9 mg, 0.60 mmol, 3.0 equiv), pivalic acid (PivOH) (20.4 mg, 0.20 mmol, 1.0 equiv) under nitrogen atmosphere. Then malonate derivative (96.7 mg, 0.20 mmol, 1.0 equiv) and THF (2.0 mL) was injected via syringe. The reaction mixture was allowed to stir at room temperature for 5 minutes, then stirring was continued at 120 °C for 12 hours. The reaction mixture was quenched with saturated NH_4Cl solution (10 mL). Then the organic compounds were extracted with ethyl acetate (3×10 mL). All the organic layers were combined, dried over Na_2SO_4, volatiles were removed under reduced pressure and desired compound was isolated via column chromatography (10% ethyl acetate in petroleum ether) as off white solid (62.7 mg, 0.18 mmol, 88%); **m.p.** 184.6-188.2 °C; **R$_f$** 0.73 (20% ethyl acetate in pentane); **^{1}H NMR (400 MHz, CDCl$_3$)** δ 8.52 (dd, J = 2.1, 0.5 Hz, 1H), 8.24 (dd, J = 8.4, 2.2 Hz, 1H), 7.97 (dd, J = 8.4, 0.5 Hz, 1H), 7.86 (ddd, J = 7.5, 1.3, 0.7 Hz, 1H), 7.81 (ddd, J = 7.6, 1.4, 0.8 Hz, 1H)., 7.53 (td, J = 7.5, 1.3 Hz, 1H), 7.47 (td, J = 7.5, 1.3 Hz, 1H), 4.26 (q, J = 7.1 Hz, 4H), 1.27 (t, J = 7.1 Hz, 6H).; **^{13}C NMR (101 MHz, CDCl$_3$)** δ 167.3 (2C), 149.3, 146.4, 143.2, 140.6, 139.2, 129.8, 129.4, 127.8, 127.3, 122.9, 120.9, 115.2, 68.6, 63.0 (2C), 14.1 (2C); **HRMS** $[M+Na]^+$ calcd. for $C_{19}H_{17}N_2NaO_6^+$ 378.0948, found 378.0921.

Pd-catalyzed Sonogashira coupling to give diethyl 2-(4-nitro-2-(phenylethynyl)phenyl)-2-phenylmalonate (7d):

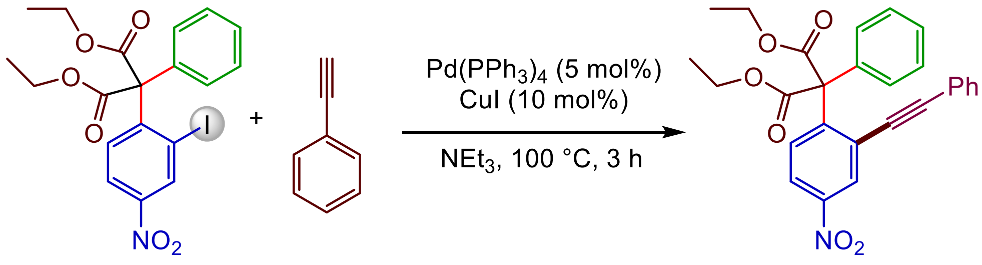

3a **7d**

Following a modified procedure from Jana,[13] diarylated malonate (48.3 mg, 0.10 mmol, 1.0 equiv), $Pd(PPh_3)_4$ (5.8 mg, 5 μmol, 0.05 equiv), CuI (1.9 mg, 10 μmol, 0.1 equiv) were added to an oven-dried microwave vial. The sealed vial was evacuated under vacuum and filled with argon. NEt_3 (1 mL) was added, followed by phenylacetylene (16 μL, 0.15 mmol, 1.5 equiv) The mixture was stirred at 100 °C for 3 h. The reaction was quenched by addition of water (5 mL). and aqueous phase was extracted with EtOAc (3×10 mL) before the combined organic layers were dried with Na_2SO_4. After filtration and evaporation of solvent,

the residue was purified by column chromatography (10% ethyl acetate in petroleum ether) to isolate diethyl 2-(4-nitro-2-(phenylethynyl)phenyl)-2-phenylmalonate (40.0 mg, 0.092 mmol, 92%) as light yellow solid; **m.p.** 110.4-113-2 °C; **R*f*** 0.26 (10% ethyl acetate in petroleum ether); **^{1}H NMR (400 MHz, $CDCl_3$)** δ 8.41 (d, *J* = 2.5 Hz, 1H), 8.08 (dd, *J* = 8.8, 2.5 Hz, 1H), 7.41 – 7.29 (m, 10H), 7.22 (d, *J* = 8.8 Hz, 1H), 4.26 (app qd, *J* = 7.1, 4.3 Hz, 4H), 1.19 (t, *J* = 7.1 Hz, 6H); **^{13}C NMR (101 MHz, $CDCl_3$)** δ 168.4 (2C), 148.3, 147.0, 136.6, 131.6 (2C), 129.9, 129.3, 128.9 (2C), 128.8 (2C), 128.5 (2C), 128.4, 127.3, 126.4, 122.5, 122.0, 98.4, 86.4, 69.3, 62.7 (2C), 13.9 (2C); **HRMS** $[M+Na]^+$ $C_{27}H_{23}NNaO_6^+$: 480.1418, found 480.1414.

5.2 Synthesis and application of cyclic salts

Synthesis of cyclic salts:

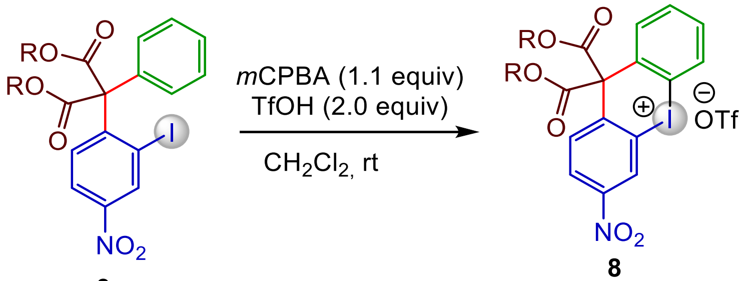

3a **8**

General procedure 1C: Following a literature procedure,[14] diethyl 2-(2-iodo-4-nitrophenyl)-2-phenylmalonate **3a** (1.00 equiv) and *m*CPBA (1.10 equiv) were dissolved in CH_2Cl_2 (10 mL). TfOH (2.00 equiv) was added dropwise at 0 °C and the reaction was stirred at room temperature for 2 h. Then solvent was removed under reduced pressure, Et_2O (40 mL) was added and the mixture was stirred vigorously for 10 h at room temperature, to precipitate the cyclic salt. The salt was filtered over a sintered funnel and washed with Et_2O (40 mL). The solid on the sintered funnel was eluted with MeOH (20 mL). The solvent was removed and re-precipitation of the product was induced by stirring in diethyl ether (20 mL). After removal of the solvent, the cyclic salt was isolated.

10,10-Bis(methoxycarbonyl)-3-nitro-10*H*-dibenzo[*b*,*e*]iodinin-5-ium triflate (8a):

Following general procedure **1C** dimethyl 2-(2-iodo-4-nitrophenyl)-2-phenylmalonate **4a** (227 mg, 0.50 mmol, 1.00 equiv), *m*CPBA (105 mg, 0.55 mmol. 1.10 equiv, 90% active oxidant), TfOH (88 µL, 1.00 mmol, 2.00 equiv) were reacted in CH_2Cl_2 (2.5 mL). for 2 h. The cyclic salt **7a** (246 mg, 0.41 mmol, 82%) was isolated as white solid.

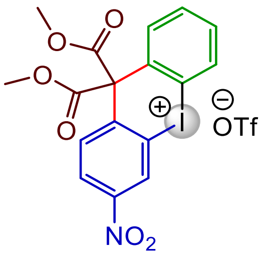

m.p. 258.7-260.2 °C; **^{1}H NMR (400 MHz, DMSO-*d*$_6$)** δ 9.00 (d, *J* = 2.4 Hz, 1H), 8.50 (dd, *J* = 8.8, 2.5 Hz, 1H), 8.22 (dd, *J* = 8.2, 0.7 Hz, 1H), 8.04 (d, *J* = 8.8 Hz, 1H), 7.78 – 7.71 (m, 2H), 7.64 (ddd, *J* = 8.6, 6.3, 2.7 Hz, 1H), 3.93 (s, 6H). **^{13}C NMR (101 MHz, DMSO-*d*$_6$)** δ 167.0 (2C), 147.0, 142.6, 135.3, 134.6, 132.3, 132.2, 131.6, 131.1, 129.3, 126.3, 120.7 (q, *J* = 322.4 Hz), 114.3, 113.8, 73.5, 54.8 (2C); **^{19}F NMR (377 MHz, DMSO-*d*$_6$)** δ -73.0; **HRMS** $[M-OTf]^+$ calcd. for $C_{17}H_{13}INO_6^+$ 453.9782, found 453.9785.

10,10-Bis(ethoxycarbonyl)-3-nitro-10*H*-dibenzo[*b*,*e*]iodinin-5-ium triflate (8b):

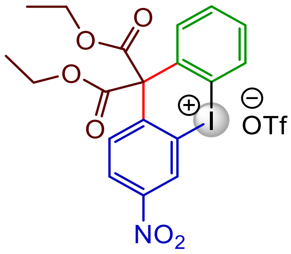

Following the above-mentioned procedure diethyl 2-(2-iodo-4-nitrophenyl)-2-phenylmalonate **3a** (891 mg, 1.84 mmol, 1.00 equiv), *m*CPBA (105 mg, 0.55 mmol. 1.10 equiv, 90% active oxidant), TfOH (88 µL, 1.00 mmol, 2.00 equiv) were reacted in CH_2Cl_2 (2.5 mL). for 2 h. The cyclic salt **7b** (1.12 g, 1.77 mmol, 96%) was isolated as white solid. **m.p.** 185.5-187.0 °C; **^{1}H NMR (400 MHz, DMSO-*d*$_6$)** δ 8.98 (d, *J* = 2.5 Hz, 1H), 8.51 (dd, *J* = 8.8, 2.5 Hz, 1H), 8.23 – 8.17 (m, 1H), 8.02 (d, *J* = 8.9 Hz, 1H), 7.78 – 7.71 (m, 2H), 7.62 (ddd, *J* = 8.6, 6.2, 2.8 Hz, 1H), 4.41 (q, *J* = 7.1 Hz, 4H), 1.22 (t, *J* = 7.1 Hz, 6H). **^{13}C NMR (101 MHz, DMSO-*d*$_6$)** δ 166.4 (2C), 147.0, 142.7, 135.5, 134.5, 132.2, 132.1, 131.5, 131.0, 129.3, 126.3, 120.7 (q, *J* = 322.3 Hz), 114.3, 113.8, 73.4, 64.0 (2C), 13.5 (2C); **^{19}F NMR (377 MHz, DMSO-*d*$_6$)** δ -77.7 **HRMS** $[M\text{-}OTf]^+$ calcd. for $C_{19}H_{17}INNaO_6^+$ 482.0095, found 482.0094.

10,10-bis(Ethoxycarbonyl)-3-nitro-10*H*-dibenzo[*b*,*e*]iodinin-5-ium tosylate (8c):

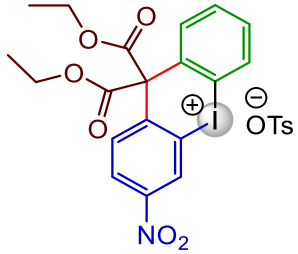

Diethyl 2-(2-iodo-4-nitrophenyl)-2-phenylmalonate **3a** (96.7 mg, 0.20 mmol, 1.00 equiv), TsOH·H_2O (41.8 mg, 1.00 mmol, 1.10 equiv) and *m*CPBA (82.2 mg, 0.3 mmol. 1.50 equiv, 63% active oxidant) were dissolved in CH_2Cl_2 (2 mL). The reaction was stirred at 60 °C for 20 h. The solvent was removed, Et_2O (5 mL) was added and the mixture was stirred vigorously for 20 h at room temperature, to precipitate the cyclic salt. The salt was filtered through a sintered funnel and washed with Et_2O (5 mL). The solid on the sintered funnel was eluted with MeOH (5 mL). After removal of the solvent, the cyclic salt (74 mg, 0.11 mmol, 56%) was isolated as light-yellow solid. **m.p.** 114.7 °C; **^{1}H NMR (400 MHz,** MeOD-***d*$_4$)** δ 9.01 (d, *J* = 2.4 Hz, 1H), 8.57 (dd, *J* = 8.8, 2.4 Hz, 1H), 8.20 (dd, *J* = 8.0, 1.2 Hz, 1H), 8.09 (d, *J* = 8.9 Hz, 1H), 7.82 – 7.74 (m, 2H), 7.69 (d, *J* = 8.1 Hz, 2H), 7.60 (ddd, *J* = 8.7, 7.0, 1.9 Hz, 1H), 7.21 (d, *J* = 7.9 Hz, 2H), 4.46 (q, *J* = 7.1 Hz, 4H), 2.36 (s, 3H), 1.31 (t, *J* = 7.1 Hz, 6H); **^{13}C NMR (101 MHz,** MeOD-***d*$_4$)** δ 166.4 (2C), 147.6, 143.1, 141.9, 140.4, 135.9, 134.1, 132.2, 132.0, 131.6, 130.9, 129.0, 128.5 (2C), 126.0, 125.5 (2C) (2 overlying signals in total 3 C), 112.6, 112.6, 74.0, 64.0 (2C), 19.9, 12.8 (2C); **HRMS** $[M\text{-}OTs]^+$ cald for $C_{19}H_{17}INNaO_6^+$ 482.0095, found 482.0086.

10-(Ethoxycarbonyl)-3-nitro-10*H*-dibenzo[*b*,*e*]iodinin-5-ium trifluoromethanesulfonate (8d):

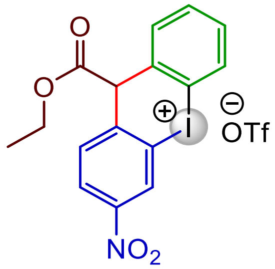

Ethyl 2-(2-iodo-4-nitrophenyl)-2-phenylacetate **7b** (114 mg, 0.28 mmol, 1.00 equiv) and *m*CPBA (59 mg, 0.30 mmol. 1.10 equiv, 87% active oxidant) were dissolved in CH_2Cl_2 (1.3 mL). TfOH (49 µL, 0.55 mmol, 2.00 equiv) was added dropwise at 0 °C and the reaction was stirred at room temperature for 2 h. The solvent was removed, Et_2O (10 mL) was added and the mixture was stirred vigorously for 1 h at room temperature, to precipitate the cyclic salt. The salt was filtered through a sintered funnel and washed with Et_2O (25 mL). The solid on the sintered funnel was eluted with MeOH (20 mL). After removal of the solvent, the cyclic salt (127 mg, 0.227 mmol, 82%) was isolated as white solid. **m.p.** 230.3 °C (decomposition); **^{1}H NMR (400 MHz, DMSO-*d*$_6$)** δ 8.94 (d, *J* = 2.3 Hz, 1H), 8.55 (dd, *J* = 8.4, 2.4 Hz, 1H), 8.17 – 8.11 (m, 2H), 7.89 (dd, *J* = 7.7, 1.6 Hz, 1H), 7.72 (td, *J* = 7.5, 1.2 Hz, 1H), 7.55 (td, *J* = 7.8, 1.6 Hz, 1H), 6.38 (s,

1H), 4.20 – 4.08 (m, 2H), 1.10 (t, J = 7.1 Hz, 3H), **^{13}C NMR (101 MHz, DMSO-d_6)** δ 167.7, 147.0, 143.7, 136.0, 134.1, 133.1, 132.9, 132.3, 130.4, 129.1, 126.7, 119.1 (q, J = 325.1 Hz), 115.3, 115.0, 62.5, 58.6, 13.9; **^{19}F NMR (377 MHz, DMSO-d_6)** δ -77.7; **HRMS** [M-OTf]$^+$ calcd. for $C_{16}H_{13}INNaO_4^+$ 409.9884, found 409.9883.

Application of cyclic salts

Diethyl 3-nitro-9*H*-thioxanthene-9,9-dicarboxylate (7e):

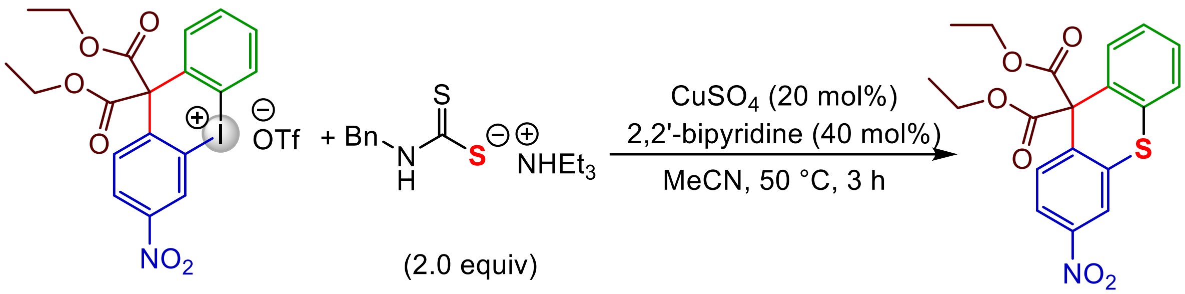

8b **7e**

In a microwave vail $CuSO_4$ (10 mg, 0.04 mmol, 0.2 equiv), cyclic diaryliodonium triflate **8b** (126.3 mg, 0.20 mmol, 1.0 equiv), triethylammonium benzylcarbamodithioate (113.8 mg, 0.40 mmol, 2.0 equiv), and 2,2'-bipyridine (12.5 mg, 0.08 mmol, 0.4 equiv) were added. The atmosphere was exchanged to nitrogen and anhydrous MeCN (2 mL) was added. The reaction mixture was stirred at room temperature for 3 hours at 50 °C. Afterwards, H_2O (5 mL) was added to the reaction mixture and the product was extracted with EtOAc (3×10 mL). The combined organic layers were washed with brine, dried over Na_2SO_4 and concentrated in vacuo. The crude product was purified by column chromatography (20:1 to 5:1 petroleum ether to ethyl acetate) to isolate diethyl 3-nitro-9*H*-thioxanthene-9,9-dicarboxylate (15.7 mg, 0.041 mmol, 21%) as yellow oil. R_f = 0.30 (in pentane:EtOAc 10:1); **^{1}H NMR (400 MHz, CDCl$_3$)** δ 8.32 (d, J = 2.3 Hz, 1H), 8.12 (dd, J = 8.7, 2.4 Hz, 1H), 7.52 (d, J = 8.7 Hz, 1H), 7.50 – 7.47 (m, 1H), 7.37 – 7.31 (m, 3H), 4.32 (q, J = 7.1 Hz, 4H), 1.25 (t, J = 7.1 Hz, 6H); **^{13}C NMR (101 MHz, CDCl$_3$)** δ 168.5 (2C), 147.1, 140.4, 135.0, 132.8, 130.9, 130.2, 129.2, 128.5, 127.4, 126.9, 121.9, 121.2, 67.7, 63.1 (2C), 14.0 (2C); **HRMS** [M+Na]$^+$ $C_{19}H_{17}NNaO_6S^+$: 410.0669, found: 410.0669.

Diethyl 2-(2-azido-4-nitrophenyl)-2-(2-iodophenyl)malonate (7f):

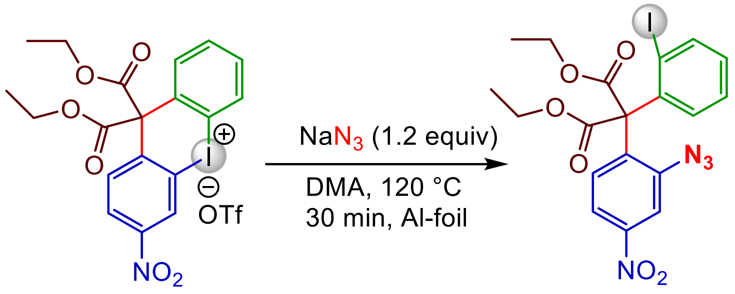

8b **7f**

Compound **7f** was prepared following a modified procedure from Jiang and co-workers.[15] In a microwave vial under nitrogen atmosphere, cyclic diaryliodonium salt **8b** (63.1 mg, 0.1 mmol, 1.0 equiv) and NaN_3 (7.8 mg, 0.12 mmol, 1.2 equiv) were dissolved in DMA (1 mL). The vessel was wrapped with aluminum

foil to protect the solution from exposure to light and the mixture was stirred for 30 min at 120 °C. After completion of the reaction, water (5 mL) was added. The solution was extracted with EtOAc (3·5 mL) and the combined organic layers were dried over Na_2SO_4. After filtration and evaporation of solvent, the residue was purified by column chromatography (5% ethyl acetate in petroleum ether) to isolate 7d (25.0 mg, 0.048 mmol, 48%) as yellow solid. **R_f** = 0.18 (10% ethyl acetate in petroleum ether); **m.p.** 123.6-125.1 °C; **^{1}H NMR (400 MHz, $CDCl_3$)** δ 8.09 (d, *J* = 2.3 Hz, 1H), 8.05 (dd, *J* = 7.9, 1.4 Hz, 1H), 7.90 (dd, *J* = 8.8, 2.3 Hz, 1H), 7.40 (ddd, *J* = 8.0, 7.3, 1.5 Hz, 1H), 7.28 (d, *J* = 8.8 Hz, 1H), 7.20 (dd, *J* = 8.0, 1.6 Hz, 1H), 7.07 (td, *J* = 7.6, 1.6 Hz, 1H), 4.42 – 4.27 (m, 4H), 1.29 (t, *J* = 7.1 Hz, 6H). **^{13}C NMR (101 MHz, $CDCl_3$)** δ 167.3 (2C), 148.3, 142.8, 142.0, 138.7, 134.5, 133.0, 130.9, 129.8, 128.7, 118.9, 114.6, 101.1, 71.3, 63.3 (2C), 13.9 (2C); **HRMS** $[M+Na]^+$ $C_{19}H_{17}IN_4NaO_6^+$: 547.0085; found: 547.0083.

6 Synthesis of starting materials

6.1 Synthesis of diaryliodonium salts 1

General procedures for the synthesis of diaryliodonium salts **1** are given below, using slight modifications of one-pot procedures from the Olofsson group[4, 14, 16-19] and other literature.[20-24]

General procedure 2A: synthesis of Ar_2IOTf

Following a standard literature procedure by Olofsson and coworkers.[14, 16] Iodoarene (1.0 equiv) was added to a stirring solution of *m*CPBA (1.2 equiv, 79%) in CH_2Cl_2, followed by dropwise addition of TfOH (2.0 equiv) at 0 °C. After stirring for 30 min at room temperature the corresponding arene (1.2 equiv) was added and the reaction was allowed to stir for 15 h at room temperature. Then the solvent was removed under reduced pressure and the residue was triturated with diethyl ether to afford the iodonium salt after filtration.

General procedure 2A2: synthesis of electron rich Ar_2IOTf

Following a standard literature procedure by Olofsson and coworkers.[14, 16] Iodoarene (1.0 equiv) was added to a stirring solution of *m*CPBA (1.2 equiv, 79%) in CH_2Cl_2, followed by dropwise addition of TfOH (2.0 equiv) at 0 °C. After stirring for 30 min at room temperature, 2.0 equiv of water was added to quench excess triflic acid. Then the corresponding electron-rich arene (1.2 equiv) was added and the reaction was allowed to stir for 15 h at room temperature. Then the solvent was removed under reduced pressure and the residue was triturated with diethyl ether to afford the iodonium salt after filtration.

General procedure 2B: synthesis of Ar_2IBF_4

Following a literature procedure by Olofsson and coworkers.[17] Iodoarene (1.0 equiv) was added to a stirring solution of *m*CPBA (1.2 equiv, 79%) in CH_2Cl_2, followed by dropwise addition of $BF_3{\cdot}OEt_2$ (2.5 equiv) at 0 °C. After stirring for 30-90 min at room temperature the corresponding arylboronic acid (1.2 equiv) was added at 0 °C, and the reaction was allowed to stir for 2 h. Then the reaction mixture was passed through a short silica plug with (CH_2Cl_2/MeOH 20:1) eluent. The solvent was removed under reduced pressure and the residue was triturated with diethyl ether to afford the salt after filtration.

General procedure 2B2: synthesis of Ar_2IOTf through anion exchange

For the synthesis of the triflate salts via the boronic acid route, general procedure **2B** was followed until the reaction was finished. Then 1.2 equiv of TfOH was added to the reaction solution at 0 °C, and stirred for additional 30 min, then the reaction mixture was passed through a short silica plug with (CH_2Cl_2/MeOH 20:1) eluent.[19] The solvent was removed under reduced pressure and the residue was triturated with diethyl ether to afford the salt after filtration.

General procedure 2C: synthesis of Ar_2IOTs

Following a modified procedure from Kita and coworkers,[20] tosylate salts were synthesized from Koser's reagent (synthesized following General Procedure **2F**) (1.0 equiv) and an electron-rich arene (1.2-2.0 equiv) in DCM/TFE or TFE/HFIP/TFA (0.2 M). The resulting solution was stirred overnight at room temperature. Then solvent was removed under reduced pressure. The residue was triturated by the addition of diethyl ether with stirring. The precipitate was collected by filtration and dried *in vacuo* to afford the corresponding tosylate salt.

General procedure 2D: synthesis of Ar_2IBF_4 from $ArI(OAc)_2$

Following a modified literature procedure by Widdowson and coworkers.[21] To a solution of (diacetoxyiodo)arene (synthesized following General Procedure **2E**) in CH_2Cl_2 at -78 °C, $BF_3{\cdot}OEt_2$ (2.5 equiv) was added dropwise and the reaction mixture was allowed to stir for 15 min. Then the corresponding arylboronic acid (1.2 equiv) was added in one portion, the reaction was left to reach room temperature and stirred for another hour. Then the reaction mixture was filtered over a short silica column eluted with 20:1 CH_2Cl_2/MeOH mixture, the solvent was evaporated to dryness and the residue was triturated with diethyl ether to afford the product.

General procedure 2D2: synthesis of Ar_2IOTf from $ArI(OAc)_2$

Following a modified literature procedure.[22-23] To the solution containing Ar_2IBF_4 salt (obtained following procedure **2D**) in MeCN was dropwise added TfOH (1.2 equiv) at 0 °C, and stirring was continued for an hour at room temperature. The solvent was removed under reduced pressure and the residue was triturated with diethyl ether to isolate the product as powder after filtration.

Table S7: Synthesis of salts 1

Salt 1	number	General procedures	Analytical data
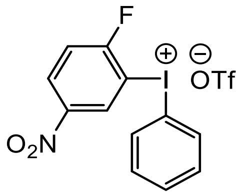	**1a**	**2A**	Reference[4, 16]
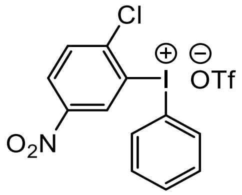	**1a-Cl**	**2A**	Reference[4, 16]
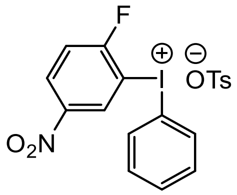	**1a-OTs**	**2C**	Reference[4, 20]
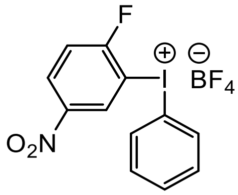	**1a-BF4**	**2B**	Reference[4, 17]
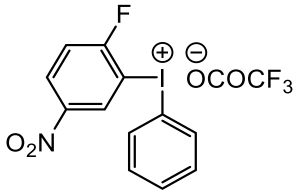	**1a-TFA**	**anion exchange**	Reference[2, 4]

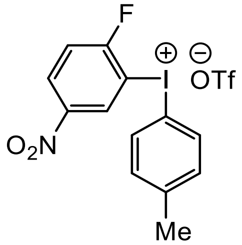	**1b**	**2A**	Reference[2, 16]
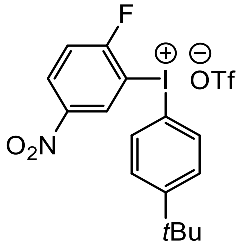	**1c**	**2A**	Reference[4, 16]
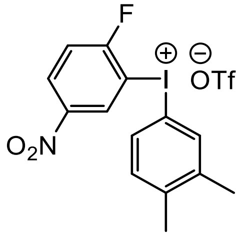	**1d**	**2A**	Reference[2, 16]
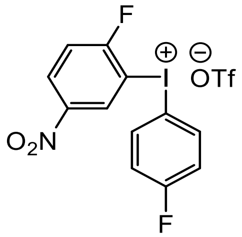	**1e**	**2A**	Reference[4, 16]
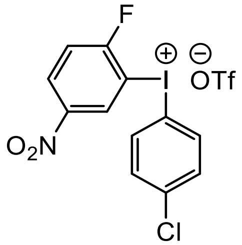	**1f**	**2A**	Reference[4, 16]
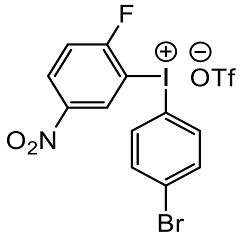	**1g**	**2A**	Reference[4, 16]

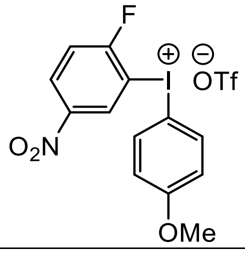	**1h**	**2A2**	Reference[4, 16]
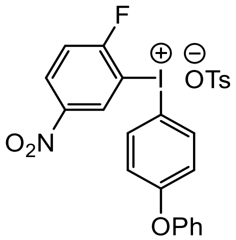	**1i**	**2A**	Reference[4, 20]
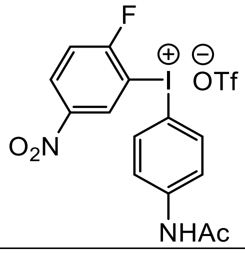	**1j**	**2A2**	Reference[4, 16]
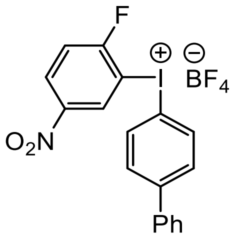	**1k**	**2C**	Reference[2, 17]
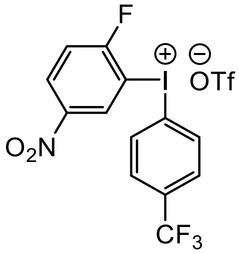	**1l**	**2B2**	Reference[4, 17]
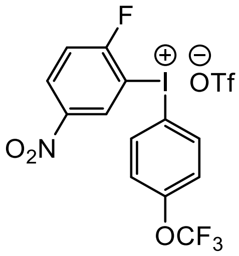	**1m**	**2A**	Reference[2, 16]

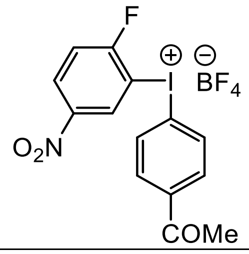	**1n**	**2D**	Reference[2, 17]
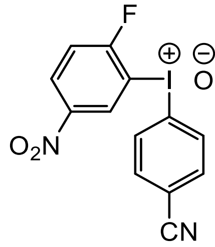	**1o**	**2D2**	Reference[2, 21]
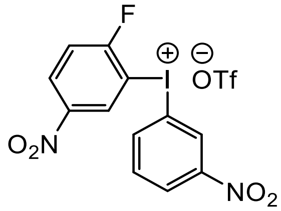	**1p**	**2B2**	Reference[4, 17]
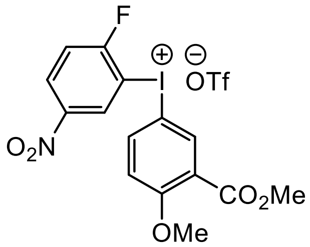	**1q**	**2A**	Reference[4, 16]
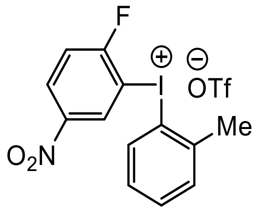	**1r**	**2B2**	Reference[2, 17]
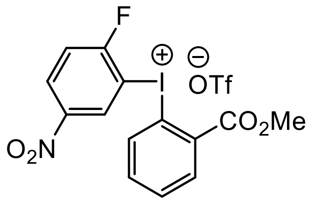	**1s**	**2D2**	Reference[2, 17]
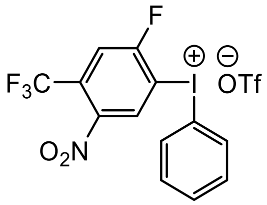	**1t**	**2A**	Reference[4, 16]

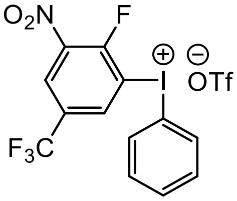	**1u**	**2A**	Reference[4, 16]
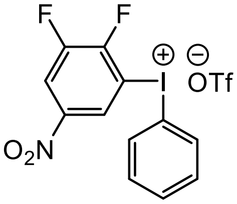	**1v**	**2A**	Reference[2, 16]
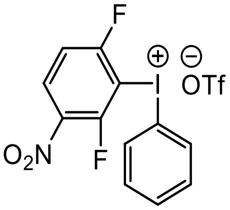	**1w**	**2A**	Reference[2, 16]
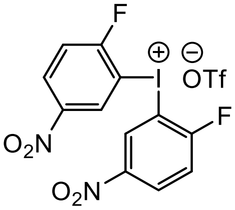	**1x**	**2D2**	Reference[2, 21]
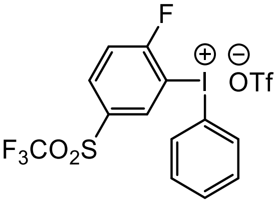	**1y**	**2A**	Reference[4, 16]
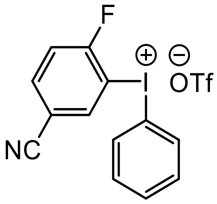	**1z**	**2A**	Reference[4, 16]
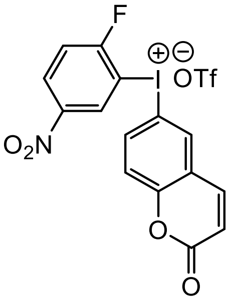	**1aa**	**2A**	Reference[2, 25]

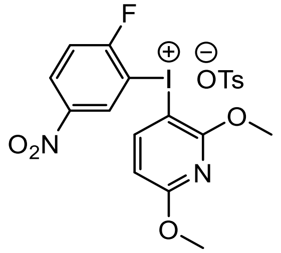	**1ab**	**2C**	Reference[2, 20]
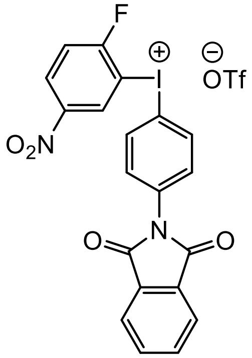	**1ac**	**2A**	Reference[2, 25]
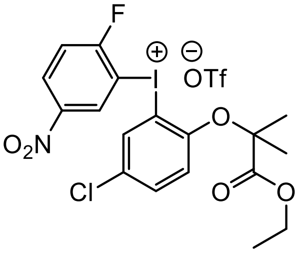	**1ad**	**2A**	Reference[2, 25]
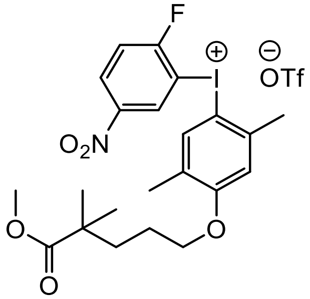	**1ae**	**2A**	Reference[25]

[1,1'-biphenyl]-4-yl(2-fluoro-5-nitrophenyl) iodonium tetrafluoroborate, **1k**:

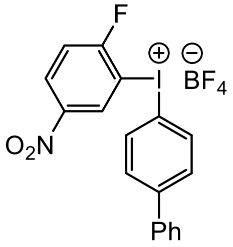

This salt was synthesized following a literature procedure.[17, 19] Yield 45%. **^{1}H NMR (400 MHz, DMSO-*d*$_6$)** δ 9.44 (dd, *J* = 4.9, 2.8 Hz, 1H), 8.57 (ddd, *J* = 9.1, 4.5, 2.8 Hz, 1H), 8.44 – 8.35 (m, 2H), 7.86 (dd, *J* = 9.1, 7.4 Hz, 3H), 7.75 – 7.66 (m, 2H), 7.52 – 7.39 (m, 3H); **^{13}C NMR (101 MHz, DMSO-*d*$_6$)** δ 162.9 (d, *J* = 258.2 Hz), 144.9 (d, *J* = 2.9 Hz), 144.1, 137.9, 136.0 (2C), 132.7 (d, *J* = 3.2 Hz), 130.9 (d, *J* = 10.5 Hz), 130.1 (2C), 129.2, 128.8 (2C), 127.1 (2C), 117.9 (d, *J* = 25.4 Hz), 115.7, 104.7 (d, *J* = 27.1 Hz); **^{19}F NMR (377 MHz, DMSO-*d*$_6$)** δ -88.66 (dt, *J* = 8.7, 4.8 Hz), -148.21.;m.p **HRMS** [M-BF$_4$]$^+$ calcd. for $C_{18}H_{12}FINO_2^+$ 419.9891; found 419.9889.

6.2 Synthesis of zwitterionic iodonium reagent 5a:

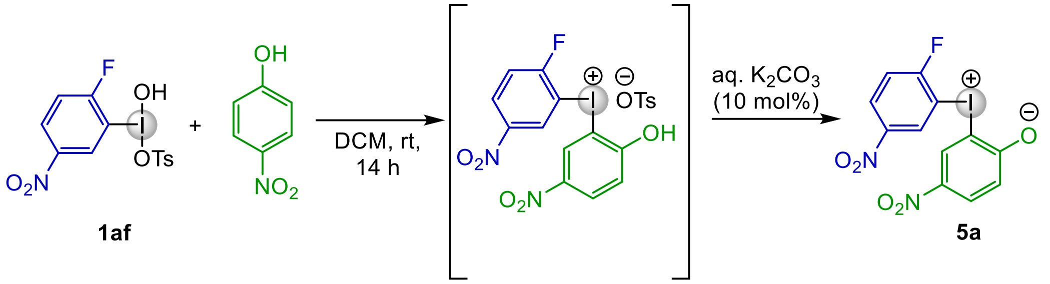

Synthesized according to a modified literature procedure.[24] After a 25 mL round bottom was charged with *p*-nitrophenol (306.0 mg, 2.2 mmol, 1.0 equiv), and dissolved in dichloromethane (8 mL), Koser's reagent (910.4 mg, 2.0 mmol, 1.1 equiv) was then added at once. The solution turned clear after some time and reaction was continued to stir for 14 h. Then solvent was removed under reduced pressure. The solid residue was triturated with diethyl ether and the solid was collected via filtration. Then the solid was treated with 5 mL of 10% aqueous K_2CO_3 solution. After 30 min the precipitate was filtered off and washed with cold water. The fluffy solid was dried overnight (308.7 mg, 0.87 mmol, 38% yield, 95% purity); **^{1}H NMR (400 MHz, DMSO-*d*$_6$)** δ 8.72 (d, *J* = 2.7 Hz, 1H), 8.31 – 8.20 (m, 3H), 7.81 (dd, *J* = 10.0, 9.1 Hz, 1H), 7.17 (dd, *J* = 9.0, 0.6 Hz, 1H); **^{13}C NMR (101 MHz, DMSO-*d*$_6$)** δ 160.4, 157.1 (d, *J* = 258.1 Hz), 144.6 (d, *J* = 2.6 Hz), 144.0, 141.8 (d, *J* = 13.4 Hz), 134.9, 125.8, 122.8 (d, *J* = 9.3 Hz), 118.7 (d, *J* = 20.7 Hz), 118.2 (d, *J* = 2.2 Hz), 116.4, 87.6; **^{19}F NMR (377 MHz, DMSO-*d*$_6$)** δ -87.4; **HRMS** $[M+H]^+$ calcd. for $C_{12}H_7FIN_2O_5^+$ 404.9378, found 404.9373.

6.3 Synthesis of other iodine(III) reagents

General procedure 2E: synthesis of (diacetoxy)iodoarene substrate

Following a literature procedure,[26] iodoarene (1.0 equiv) was added to a vigorously stirring solution of $NaOCl{\cdot}5H_2O$ (1.2 equiv) in concentrated AcOH (0.6 M). Stirring was continued for 30 min followed by addition of CH_2Cl_2 and toluene (1:1), then undissolved solid was filtered off and solvent was evaporated under reduced pressure. The resulted in solid materials were collected, and washed with petroleum ether to afford corresponding (diacetoxy)iodo substrate, which was used for the next steps without further purification.

General procedure 2F: synthesis of Koser's reagent

Following a literature procedure by Olofsson and coworkers,[17] Iodoarene **1** (1.0 equiv), *m*-CPBA (1.1 equiv) and $TsOH{\cdot}H_2O$ (1.1 equiv) were dissolved in CH_2Cl_2 and the reaction flask transferred to a preheated oil bath of 60 °C and the reaction was stirred for 2 h. A white precipitate appeared after 10-20 min. The solvent was evaporated and product was isolated by trituration in diethyl ether.

7 References

(1) Greber, G., Vogel's textbook of practical organic chemistry (5th ed.), revised by Brian S. Furniss, Antony J. Hannaford, Peter W. G. Smith, and Austin R. Tatchell, John Wiley & Sons, New York, 1514 pp. price: $84.95. *J. Polym. Sci. A Polym.* **1991,** *29*, 1223-1223.

(2) Mondal, S.; Di Tommaso, E. M.; Olofsson, B., Transition-Metal-Free Difunctionalization of Sulfur Nucleophiles. *Angew. Chem. Int. Ed.* **2023,** *62*, e202216296.

(3) Linde, E.; Olofsson, B., Synthesis of Complex Diarylamines through a Ring-Opening Difunctionalization Strategy. *Angew. Chem. Int. Ed.* **2023,** *62*, e202310921.

(4) Linde, E.; Bulfield, D.; Kervefors, G.; Purkait, N.; Olofsson, B., Diarylation of N- and O-nucleophiles through a metal-free cascade reaction. *Chem* **2022,** *8*, 850-865.

(5) Doobary, S.; Di Tommaso, E. M.; Postole, A.; Inge, A. K.; Olofsson, B., Structure-reactivity analysis of novel hypervalent iodine reagents in S-vinylation of thiols. *Front. Chem.* **2024,** *12*, 1376948.

(6) Malmgren, J.; Santoro, S.; Jalalian, N.; Himo, F.; Olofsson, B., Arylation with Unsymmetrical Diaryliodonium Salts: A Chemoselectivity Study. *Chem. Eur. J.* **2013,** *19*, 10334-10342.

(7) Norrby, P.-O.; Petersen, T. B.; Bielawski, M.; Olofsson, B., alfa-Arylation by Rearrangement: On the Reaction of Enolates with Diaryliodonium Salts. *Chem. Eur. J.* **2010,** *16*, 8251-8254.

(8) Jobin-Des Lauriers, A.; Legault, Y. C., Metathetical Redox Reaction of (Diacetoxyiodo)arenes and Iodoarenes. *Molecules* **2015,** *20*, 22635-22644.

(9) Izquierdo, S.; Essafi, S.; del Rosal, I.; Vidossich, P.; Pleixats, R.; Vallribera, A.; Ujaque, G.; Lledós, A.; Shafir, A., Acid Activation in Phenyliodine Dicarboxylates: Direct Observation, Structures, and Implications. *J. Am. Chem. Soc.* **2016,** *138*, 12747-12750.

(10) Jang, M.; Lim, T.; Park, B. Y.; Han, M. S., Metal-Free, Rapid, and Highly Chemoselective Reduction of Aromatic Nitro Compounds at Room Temperature. *J. Org. Chem.* **2022,** *87*, 910-919.

(11) Krapcho, A. P.; Weimaster, J.; Eldridge, J.; Jahngen Jr, E.; Lovey, A.; Stephens, W., Synthetic applications and mechanism studies of the decarbalkoxylations of geminal diesters and related systems effected in dimethyl sulfoxide by water and/or by water with added salts. *J. Org. Chem.* **1978,** *43*, 138-147.

(12) Song, J.; Li, Y.; Sun, W.; Yi, C.; Wu, H.; Wang, H.; Ding, K.; Xiao, K.; Liu, C., Efficient palladium-catalyzed C (sp 2)–H activation towards the synthesis of fluorenes. *New J. Chem.* **2016,** *40*, 9030-9033.

(13) Bera, K.; Jalal, S.; Sarkar, S.; Jana, U., FeCl 3-catalyzed synthesis of functionally diverse dibenzo [b, f] oxepines and benzo [b] oxepines via alkyne–aldehyde metathesis. *Org. Biomol. Chem.* **2014,** *12*, 57-61.

(14) Bielawski, M.; Zhu, M.; Olofsson, B., Efficient and general one-pot synthesis of diaryliodonium triflates: optimization, scope and limitations. *Adv. Synth. Catal.* **2007,** *349*, 2610-2618.

(15) Wang, M.; Fan, Q.; Jiang, X., Nitrogen–Iodine Exchange of Diaryliodonium Salts: Access to Acridine and Carbazole. *Org. Lett.* **2018,** *20*, 216-219.

(16) Bielawski, M.; Olofsson, B., High-yielding one-pot synthesis of diaryliodonium triflates from arenes and iodine or aryl iodides. *Chem. Commun.* **2007**, 2521-2523.

(17) Bielawski, M.; Aili, D.; Olofsson, B., Regiospecific One-Pot Synthesis of Diaryliodonium Tetrafluoroborates from Arylboronic Acids and Aryl Iodides. *J. Org. Chem.* **2008,** *73*, 4602-4607.

(18) Brown, M.; Delorme, M.; Malmedy, F.; Malmgren, J.; Olofsson, B.; Wirth, T., Synthesis of New Chiral Diaryliodonium Salts. *Synlett* **2015,** *26*, 1573-1577.

(19) Linde, E.; Mondal, S.; Olofsson, B., Advancements in the Synthesis of Diaryliodonium Salts: Updated Protocols. *Adv. Synth. Catal.* **2023,** *365*, 2751-2756.

(20) Dohi, T.; Ito, M.; Morimoto, K.; Minamitsuji, Y.; Takenaga, N.; Kita, Y., Versatile direct dehydrative approach for diaryliodonium(III) salts in fluoroalcohol media. *Chem. Commun.* **2007**, 4152-4154.

(21) Carroll, M. A.; Pike, V. W.; Widdowson, D. A., New synthesis of diaryliodonium sulfonates from arylboronic acids. *Tetrahedron Lett.* **2000,** *41*, 5393-5396.

(22) Kitamura, T.; Matsuyuki, J.-i.; Taniguchi, H., Improved Preparation of Diaryliodonium Triflates. *Synthesis* **1994,** *1994*, 147-148.

(23) Yoshimura, A.; Shea, M. T.; Guselnikova, O.; Postnikov, P. S.; Rohde, G. T.; Saito, A.; Yusubov, M. S.; Nemykin, V. N.; Zhdankin, V. V., Preparation and structure of phenolic aryliodonium salts. *Chem. Commun.* **2018,** *54*, 10363-10366.

(24) Prakash, O.; Kumar, M.; Kumar, R., A novel and convenient approach for tosyloxylation of aromatic ring of some ortho-substituted phenolic compounds using [hydroxy(tosyloxy)iodo]benzene. *Tetrahedron* **2010,** *66*, 5827-5832.

(25) Chen, Y.; Gu, Y.; Meng, H.; Shao, Q.; Xu, Z.; Bao, W.; Gu, Y.; Xue, X.-S.; Zhao, Y., Metal-Free C−H Functionalization via Diaryliodonium Salts with a Chemically Robust Dummy Ligand. *Angew. Chem. Int. Ed.* **2022,** *61*, e202201240.
(26) Watanabe, A.; Miyamoto, K.; Okada, T.; Asawa, T.; Uchiyama, M., Safer synthesis of (diacetoxyiodo) arenes using sodium hypochlorite pentahydrate. *J. Org. Chem.* **2018,** *83*, 14262-14268.

8 NMR spectra

^{1}H NMR (400 MHz, CDCl₃), 3a:

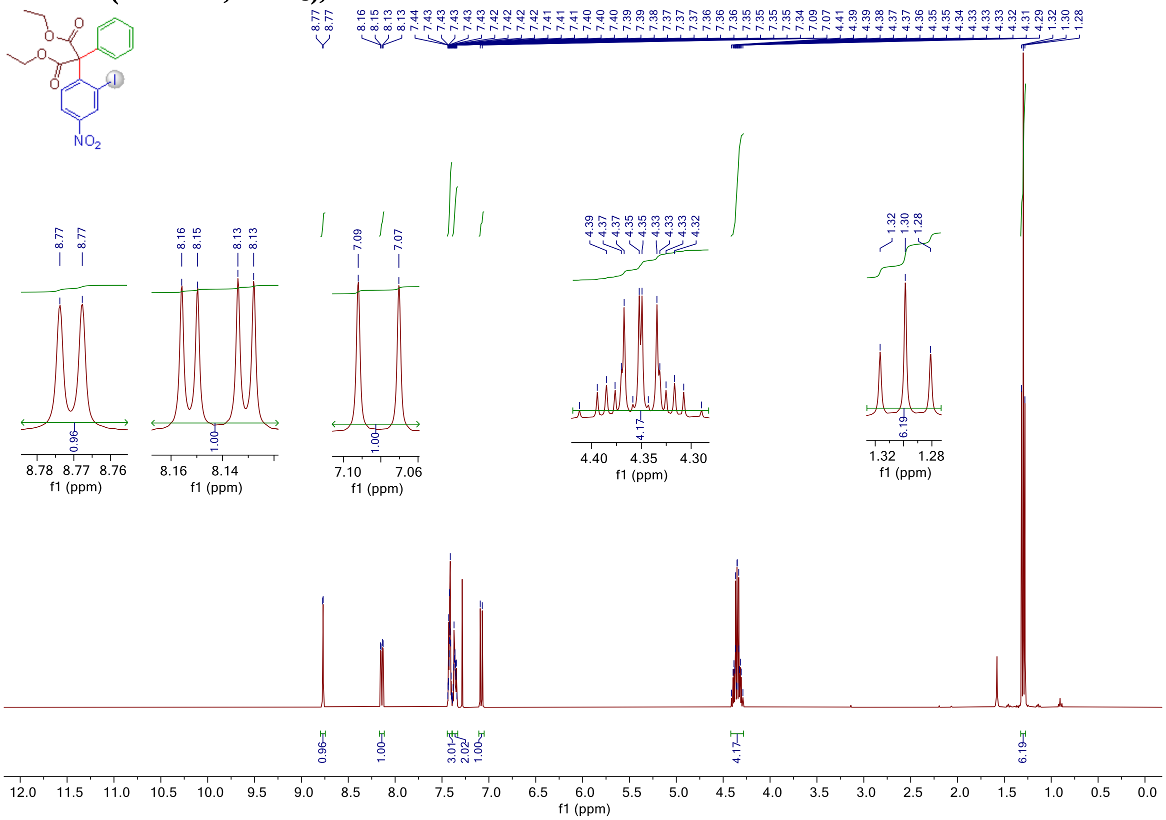

^{13}C NMR (101 MHz, CDCl₃), 3a:

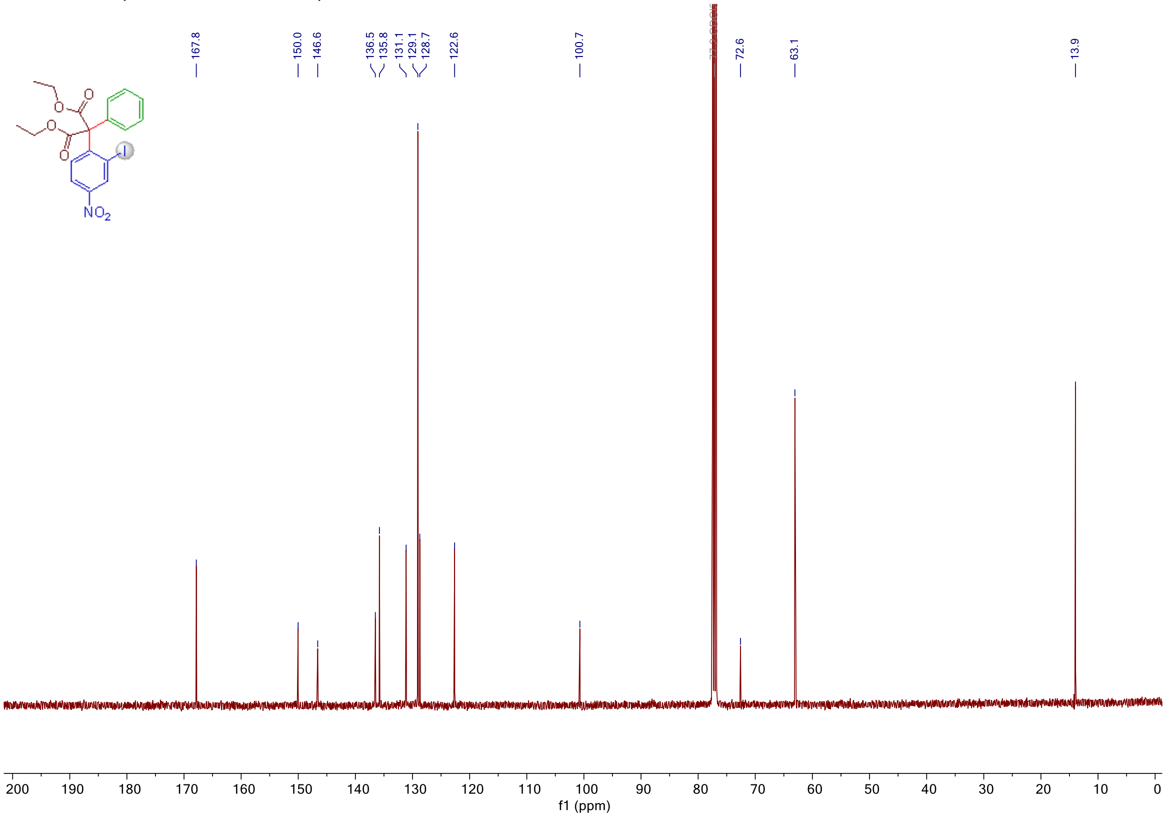

^{1}H NMR (400 MHz, CDCl3), 3b:

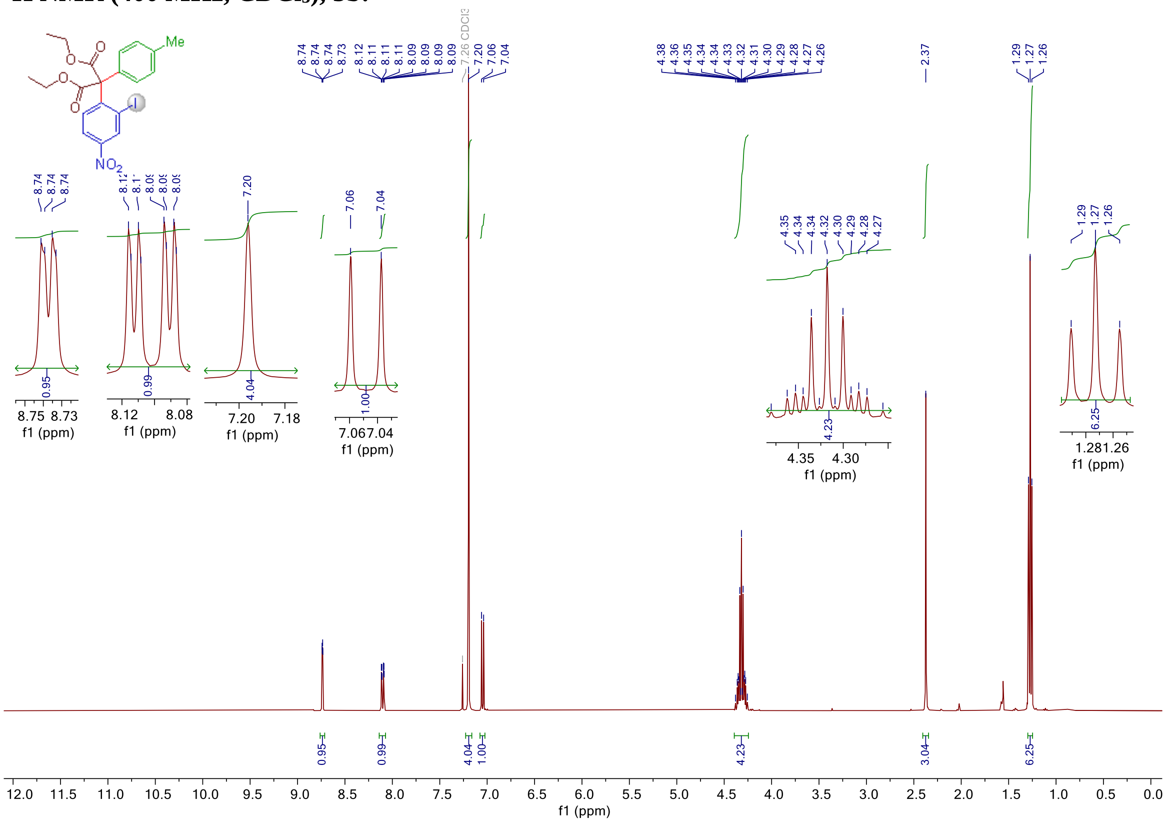

^{13}C NMR (101 MHz, CDCl3), 3b:

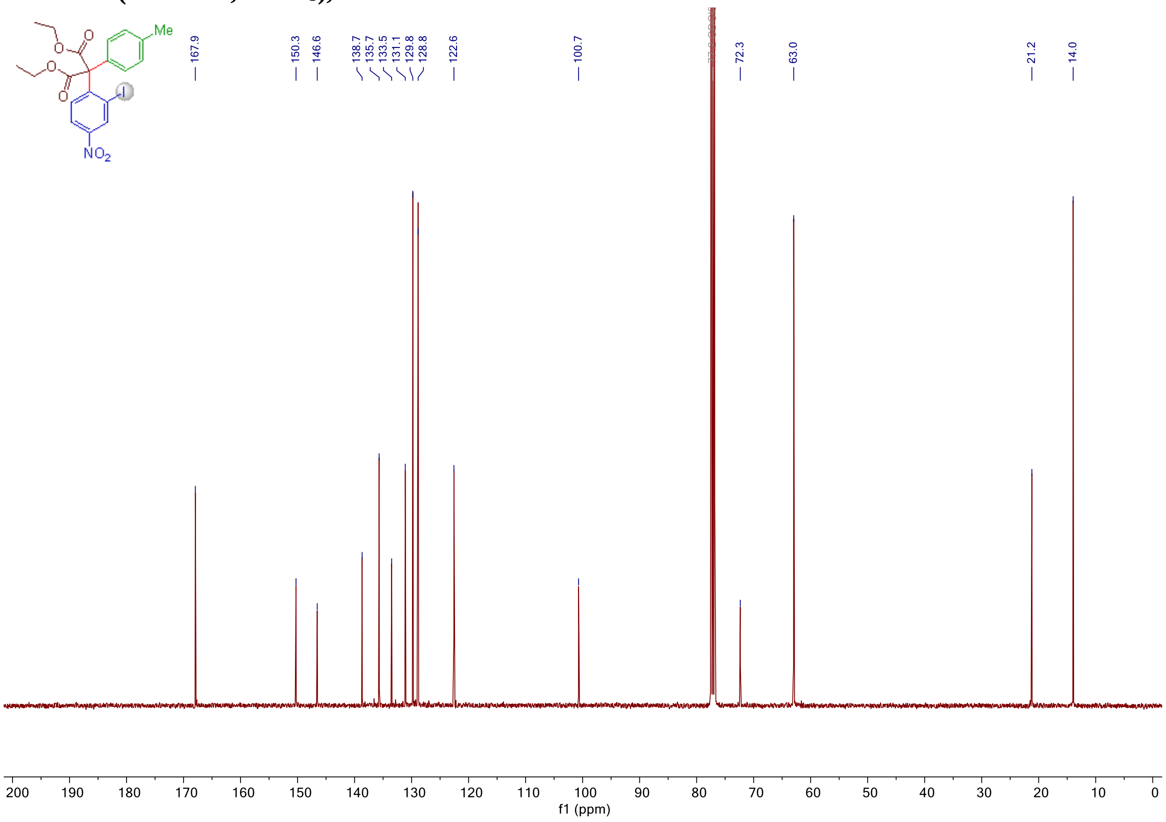

^{1}H NMR (400 MHz, CDCl$_3$), 3c:

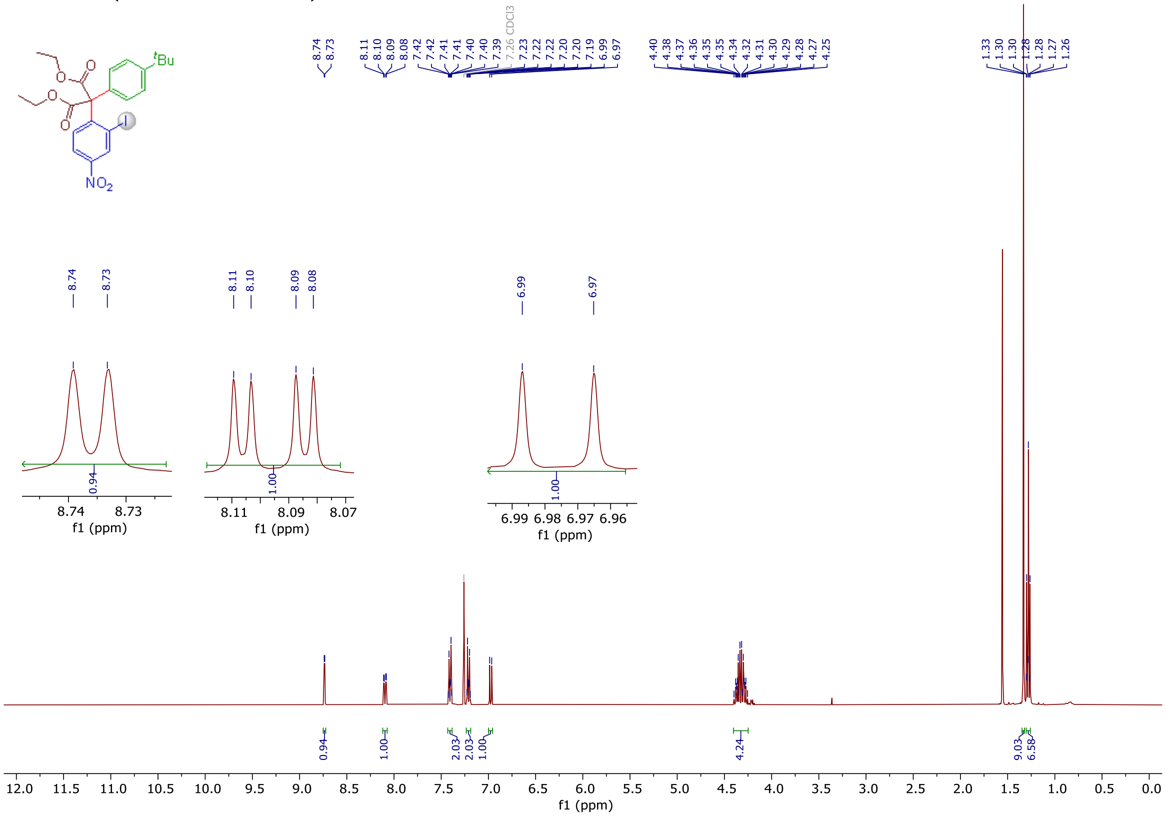

^{13}C NMR (101 MHz, CDCl$_3$), 3c:

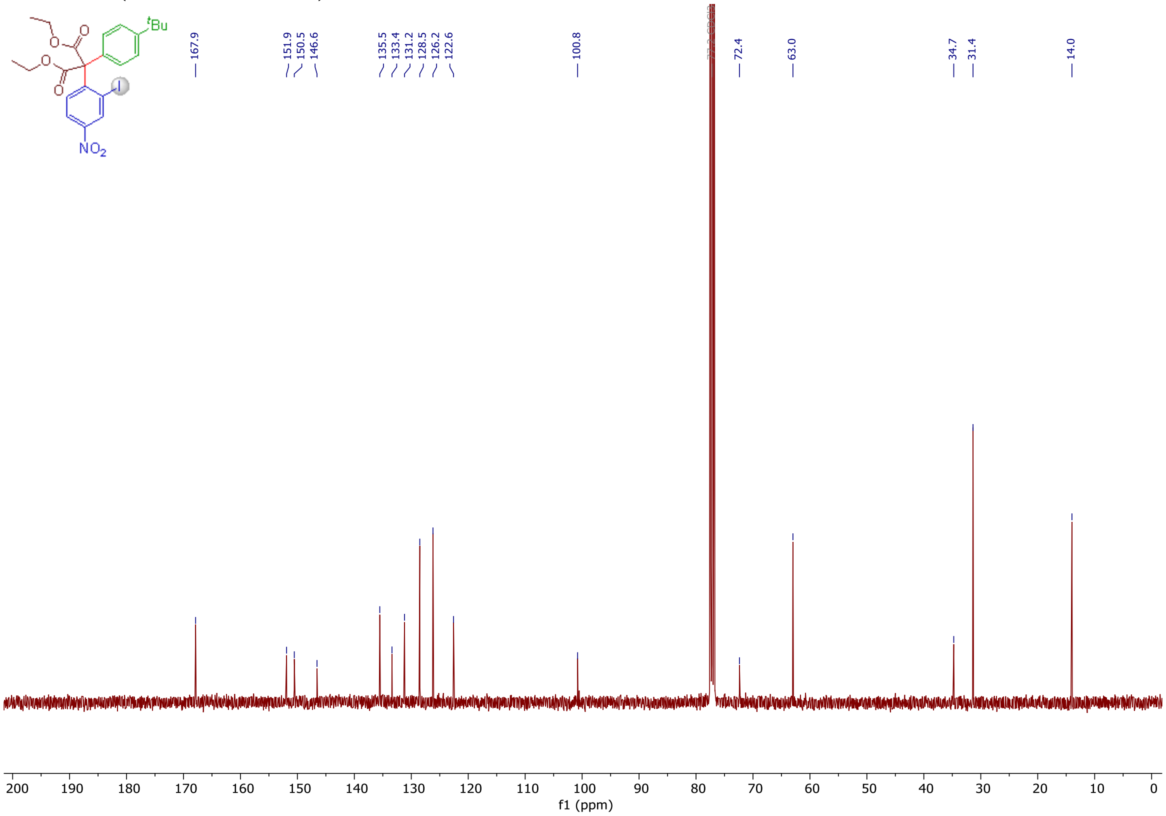

^{1}H NMR (400 MHz, $CDCl_3$), 3d:

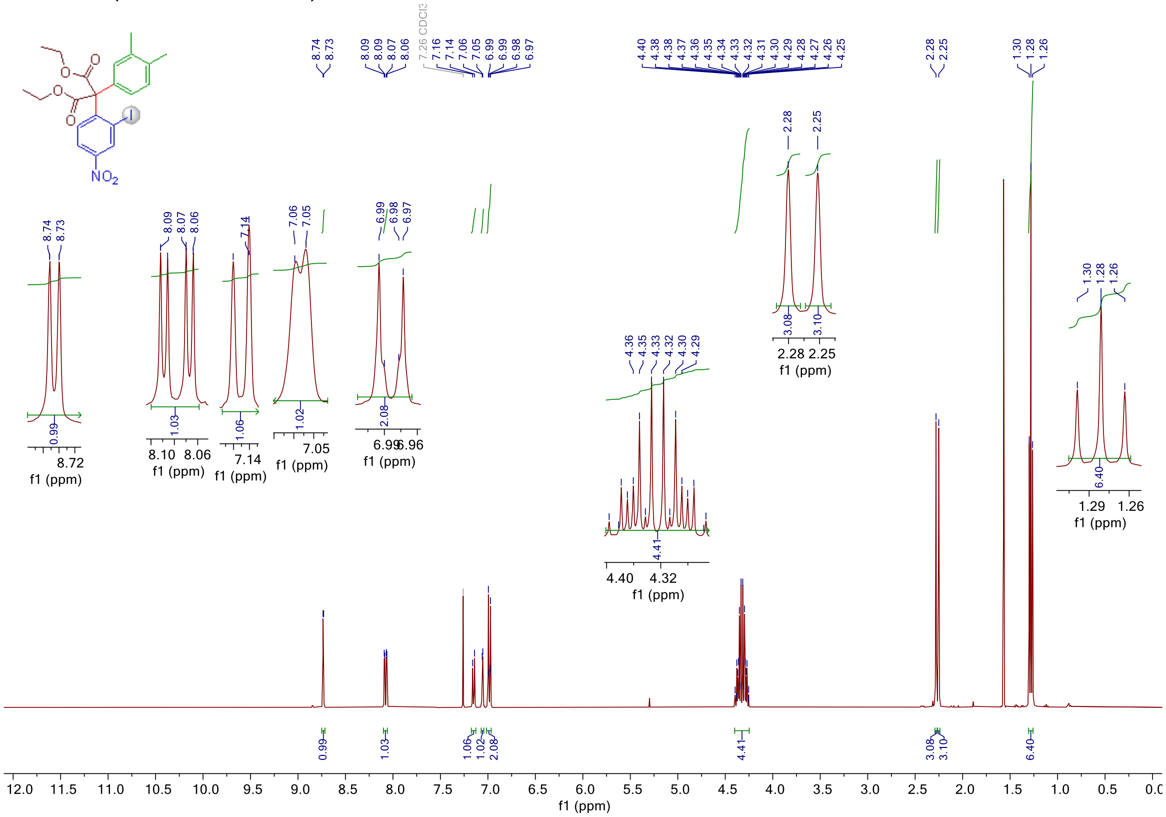

^{13}C NMR (101 MHz, $CDCl_3$), 3d:

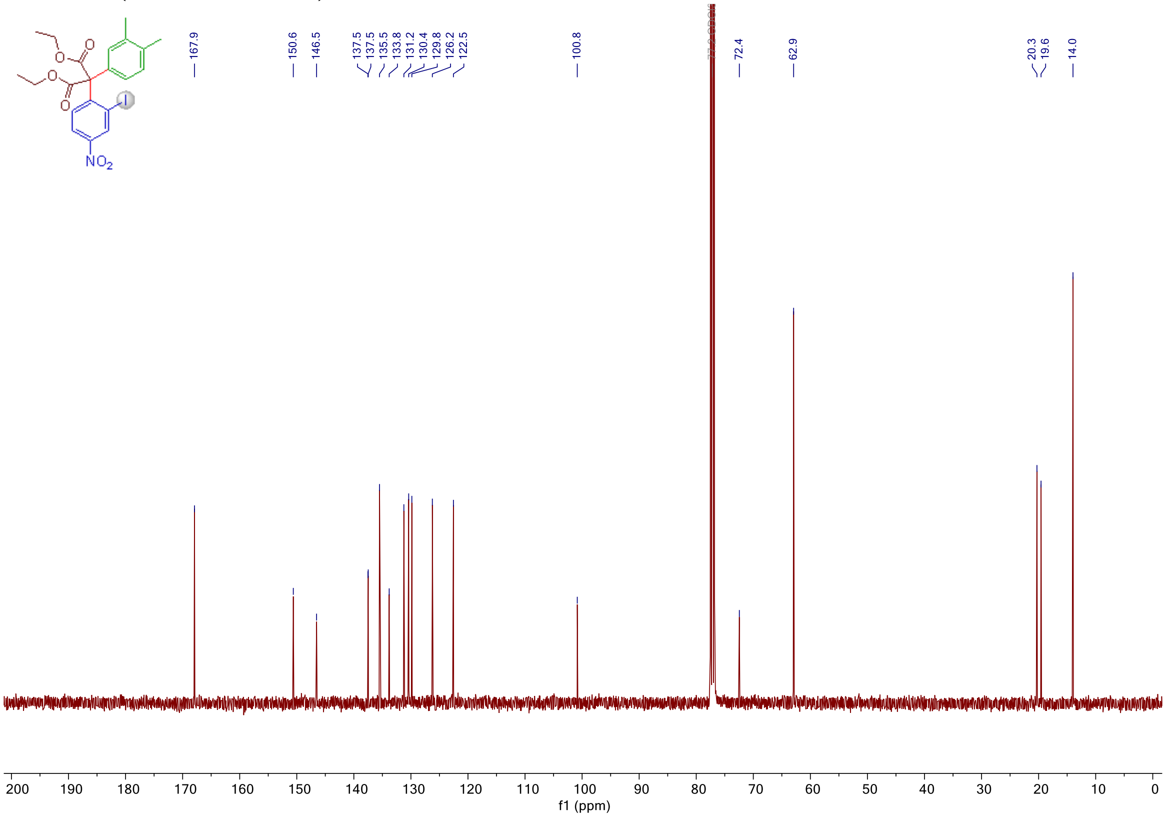

^{1}H NMR (400 MHz, $CDCl_3$), 3e:

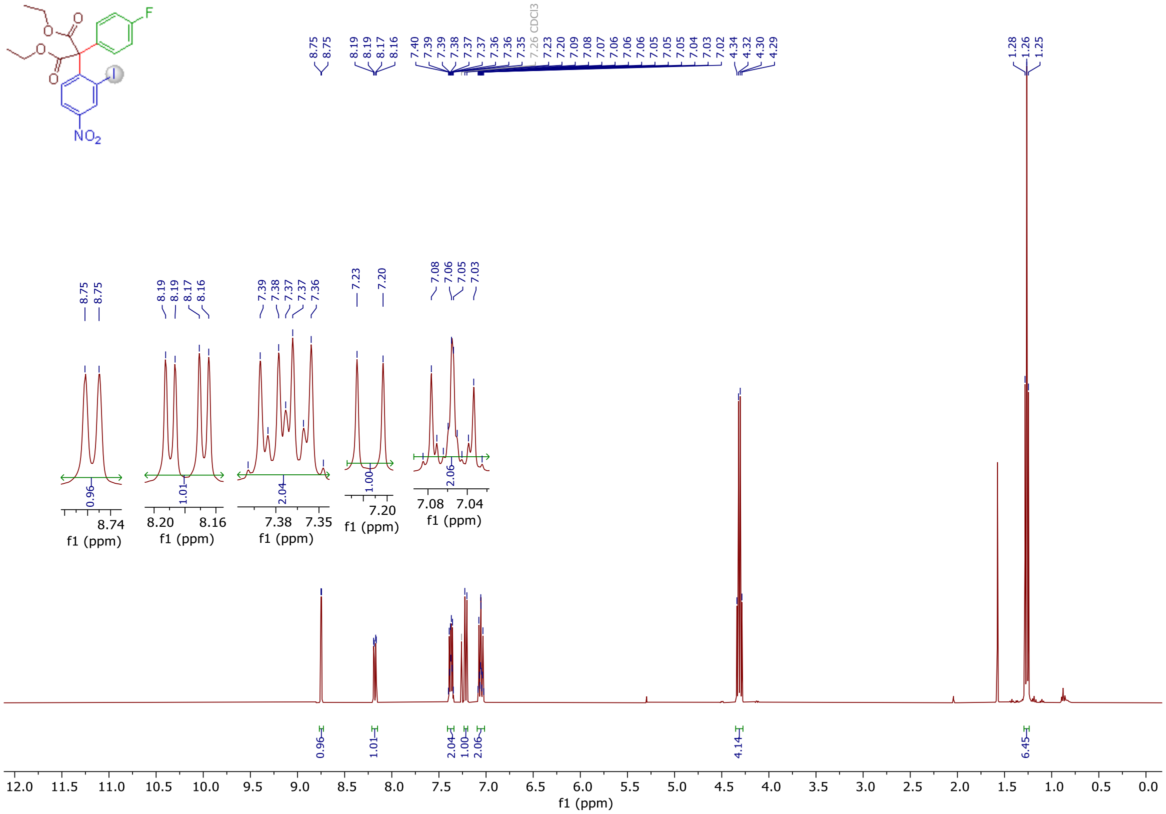

^{13}C NMR (101 MHz, $CDCl_3$), 3e:

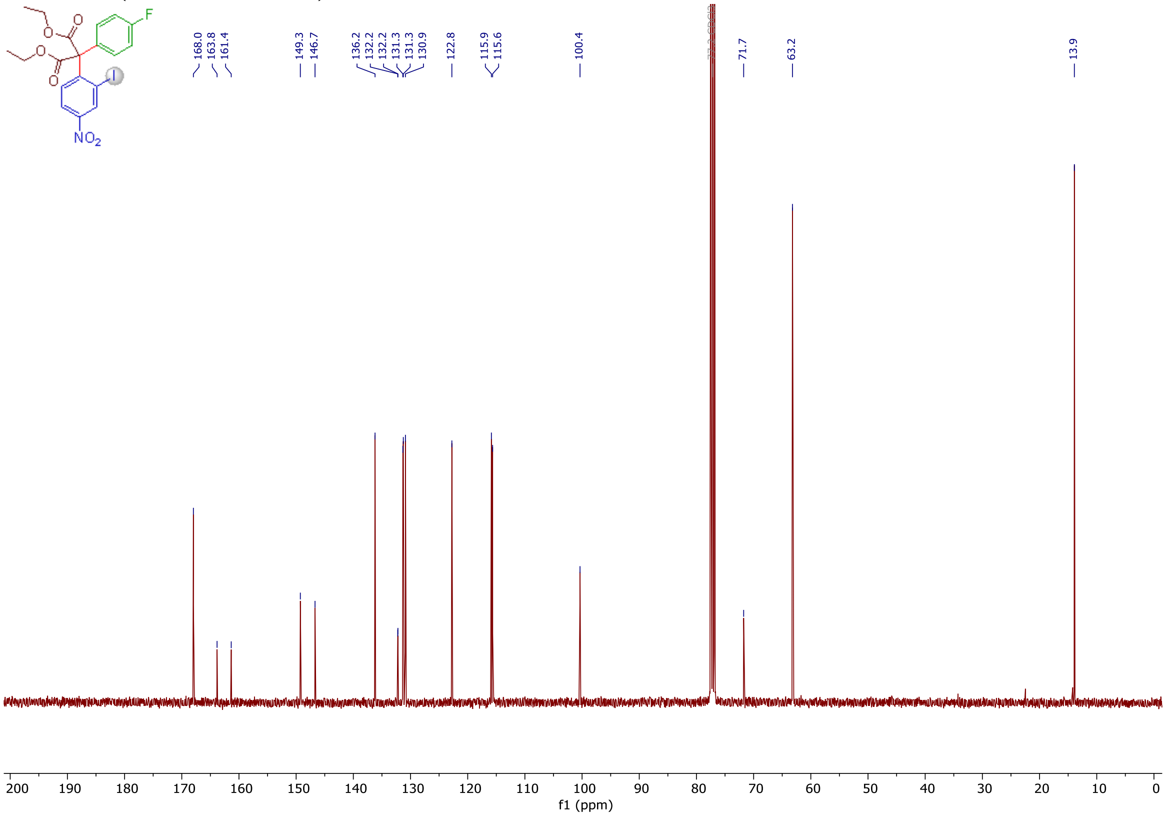

^{19}F NMR (377 MHz, CDCl$_3$), 3e:

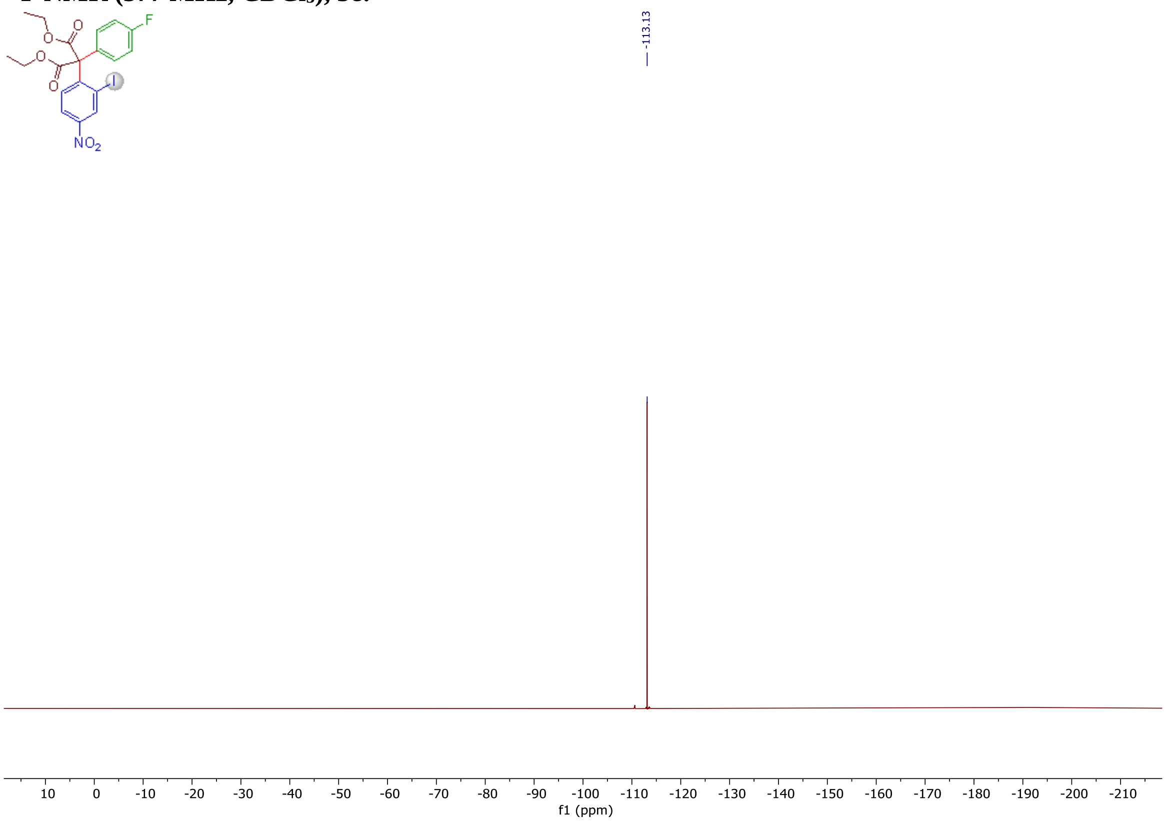

^{1}H NMR (400 MHz, CDCl$_3$), 3f:

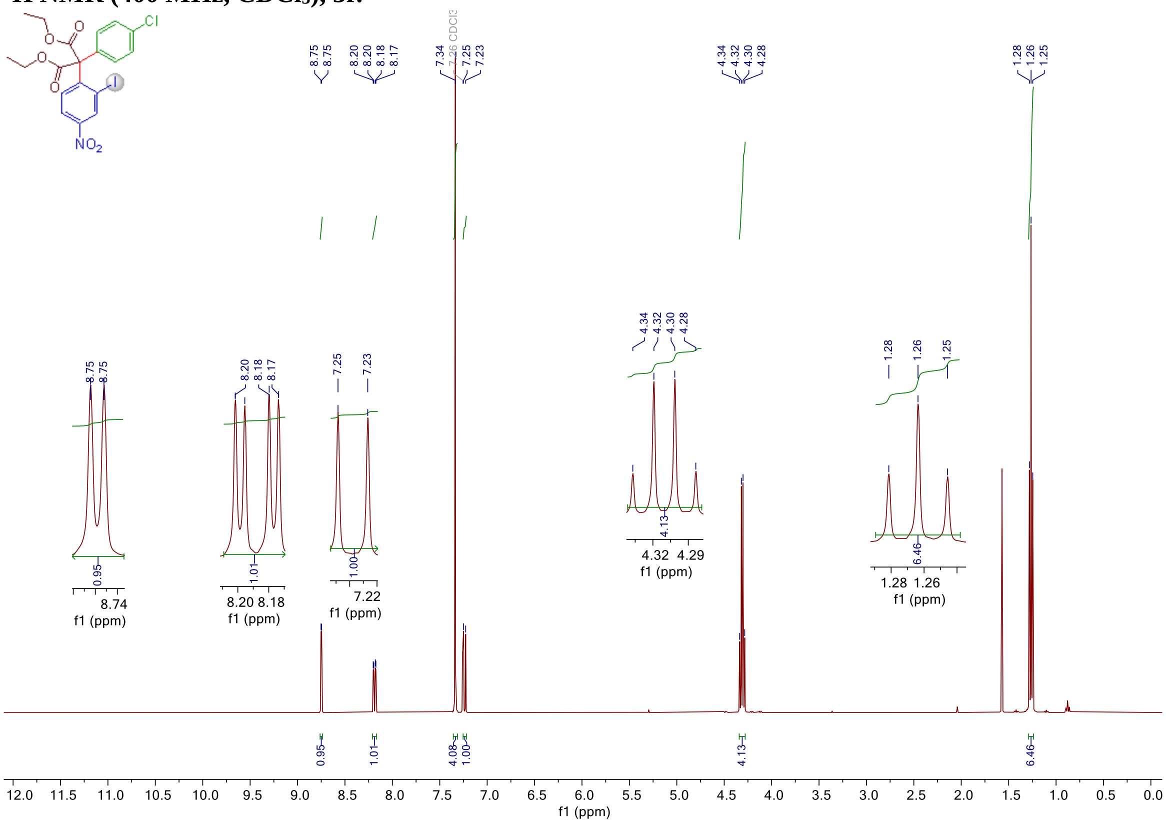

^{13}C NMR (101 MHz, $CDCl_3$), 3f:

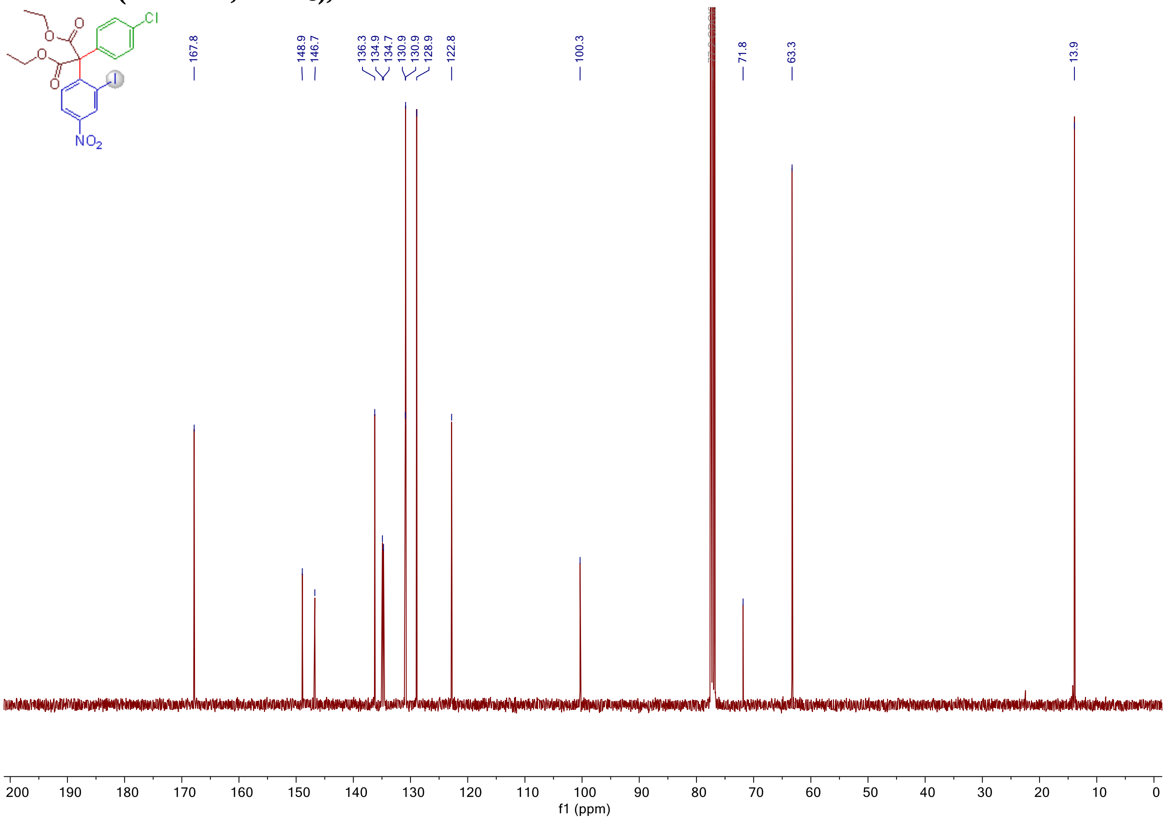

^{1}H NMR (400 MHz, $CDCl_3$), 3g:

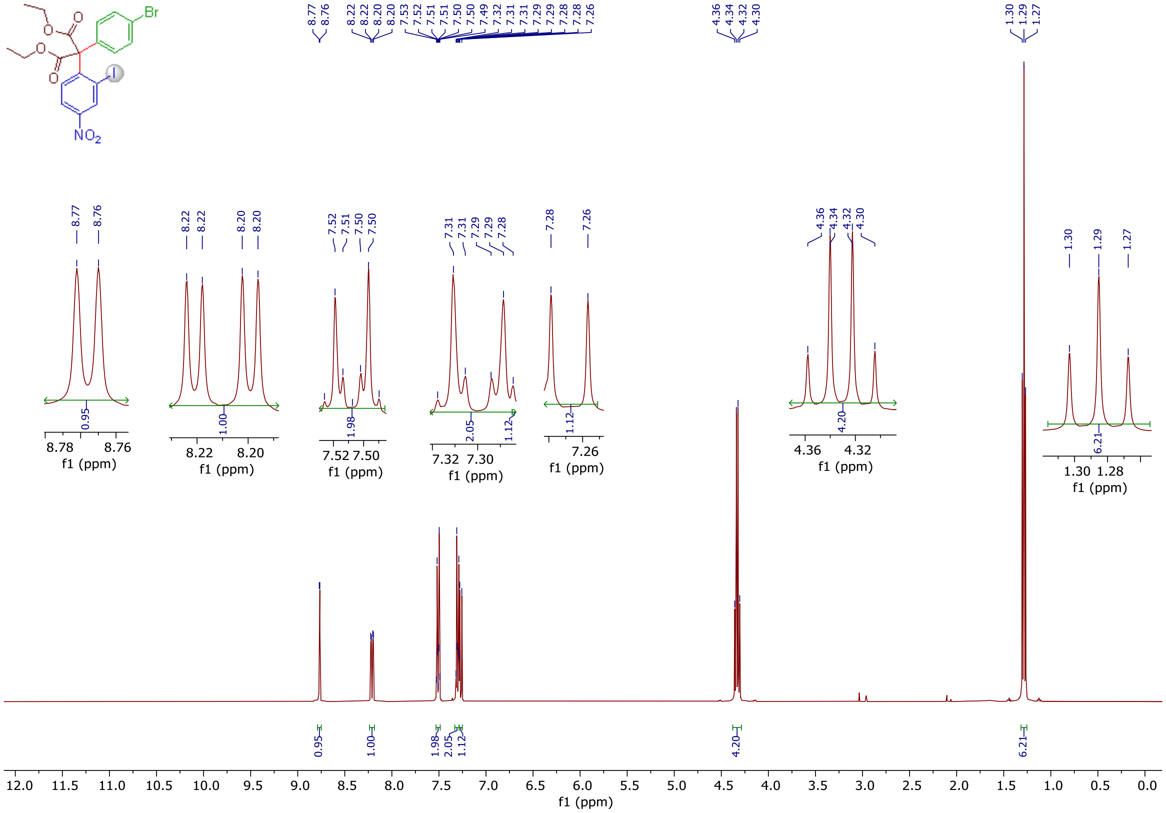

^{13}C NMR (101 MHz, CDCl3), 3g:

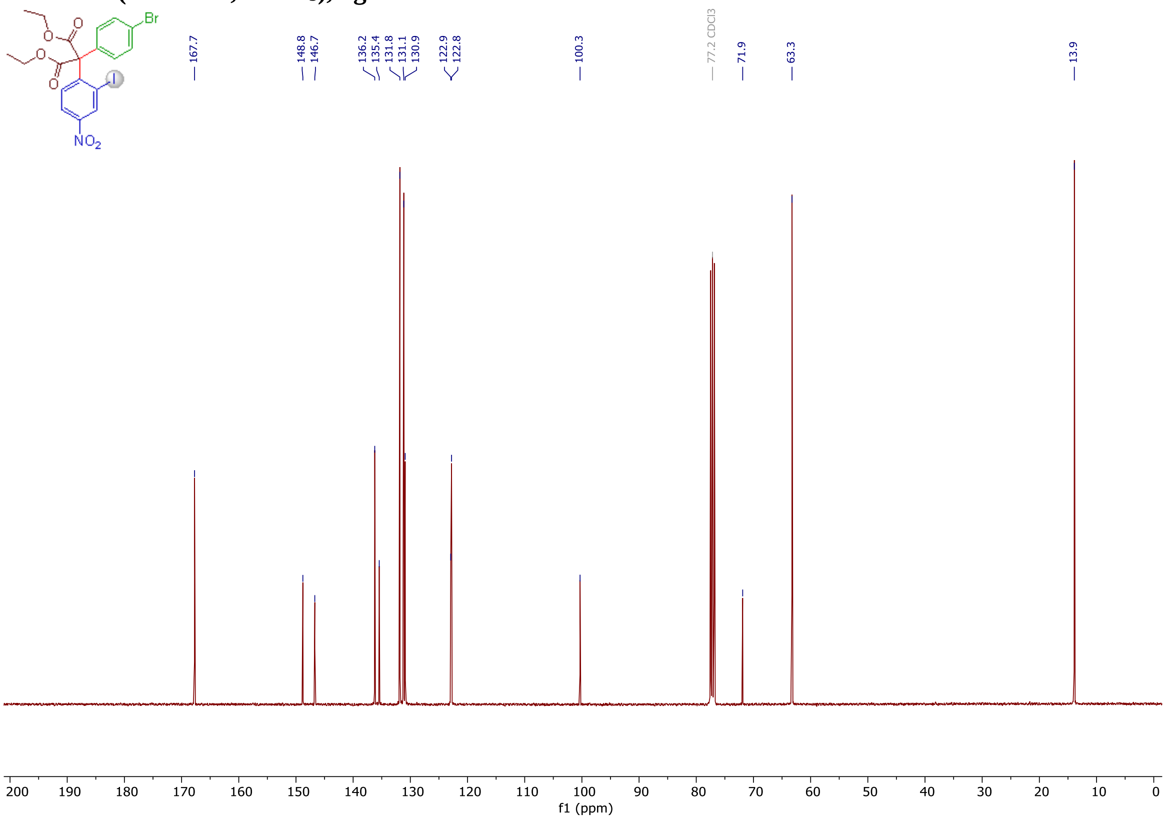

^{1}H NMR (400 MHz, CDCl3), 3h:

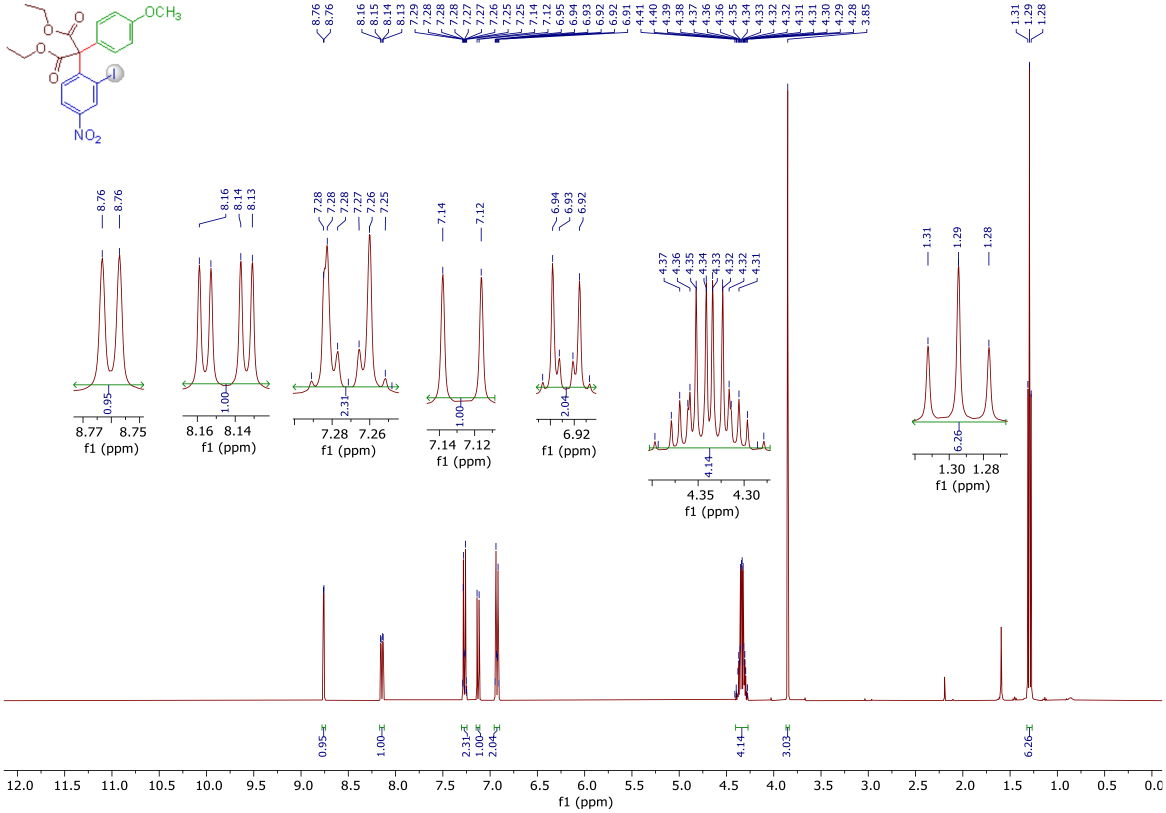

^{13}C NMR (101 MHz, CDCl$_3$), 3h:

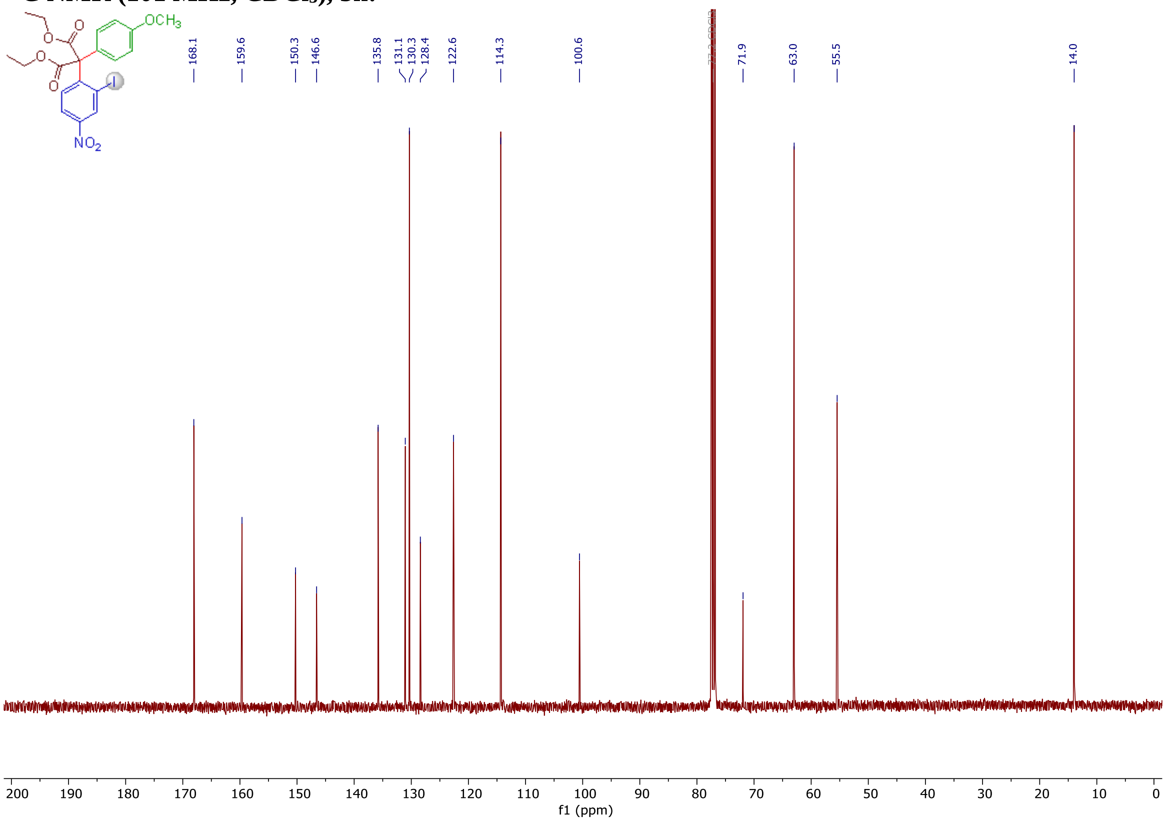

^{1}H NMR (400 MHz, CDCl$_3$), 3i:

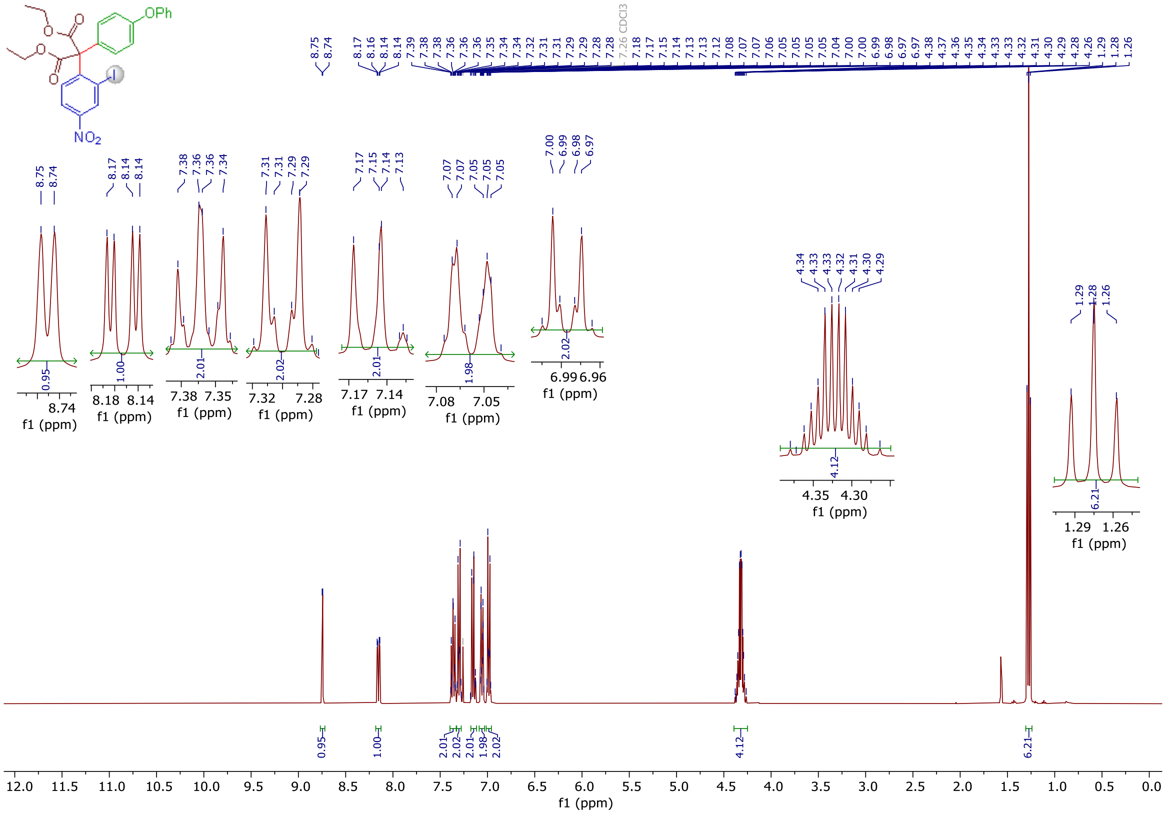

^{13}C NMR (101 MHz, $CDCl_3$), 3i:

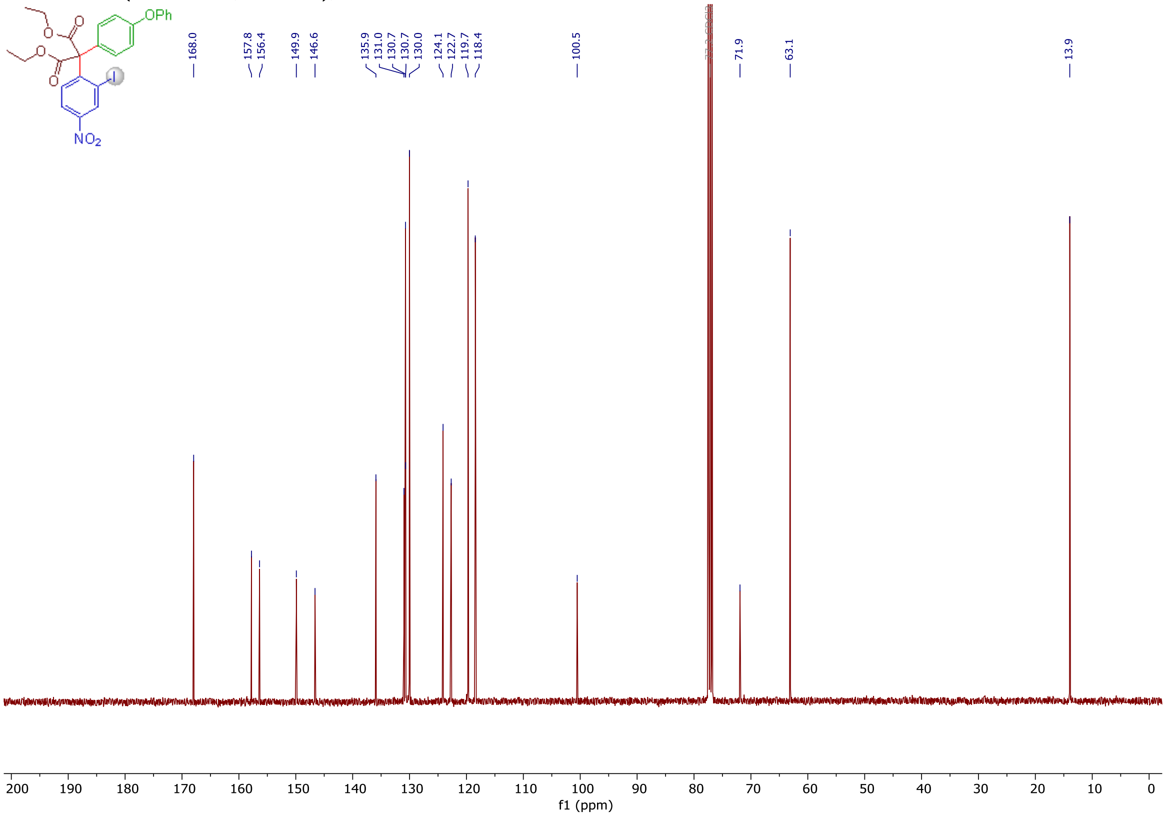

^{1}H NMR (400 MHz, $CDCl_3$), 3j:

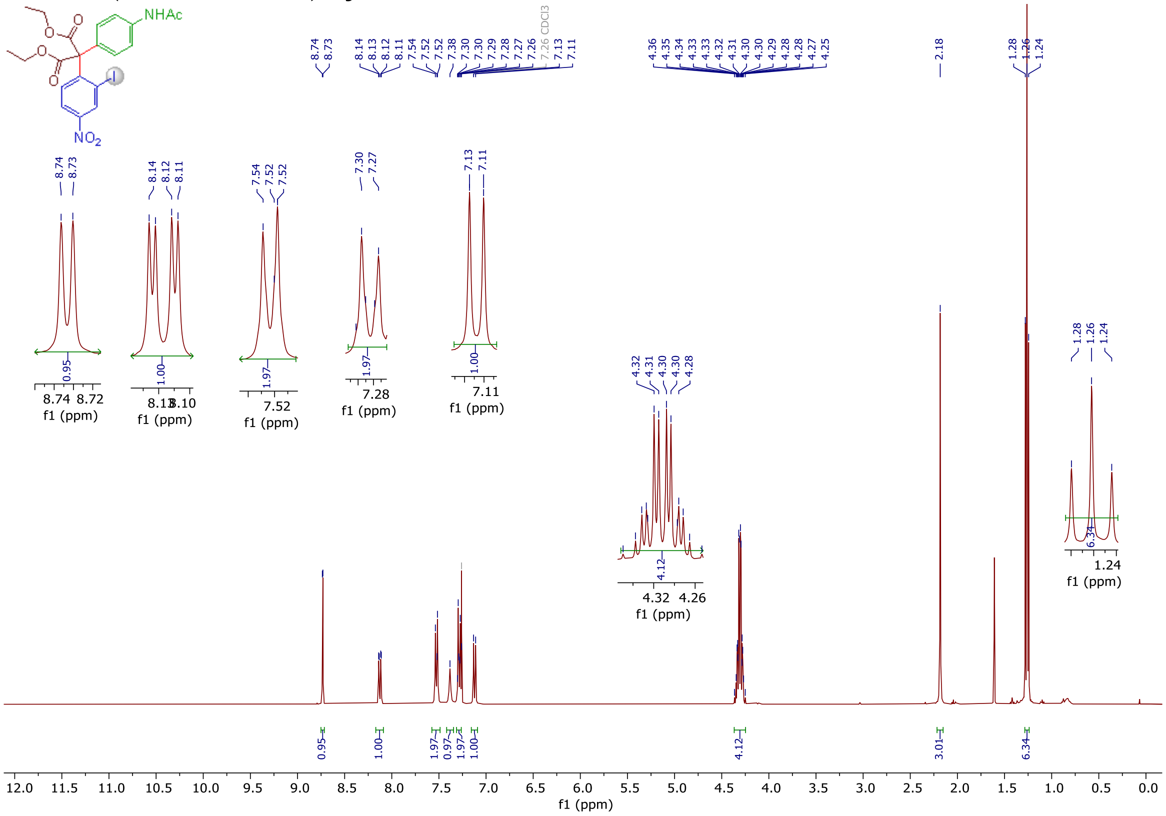

^{13}C NMR (101 MHz, CDCl$_3$), 3j:

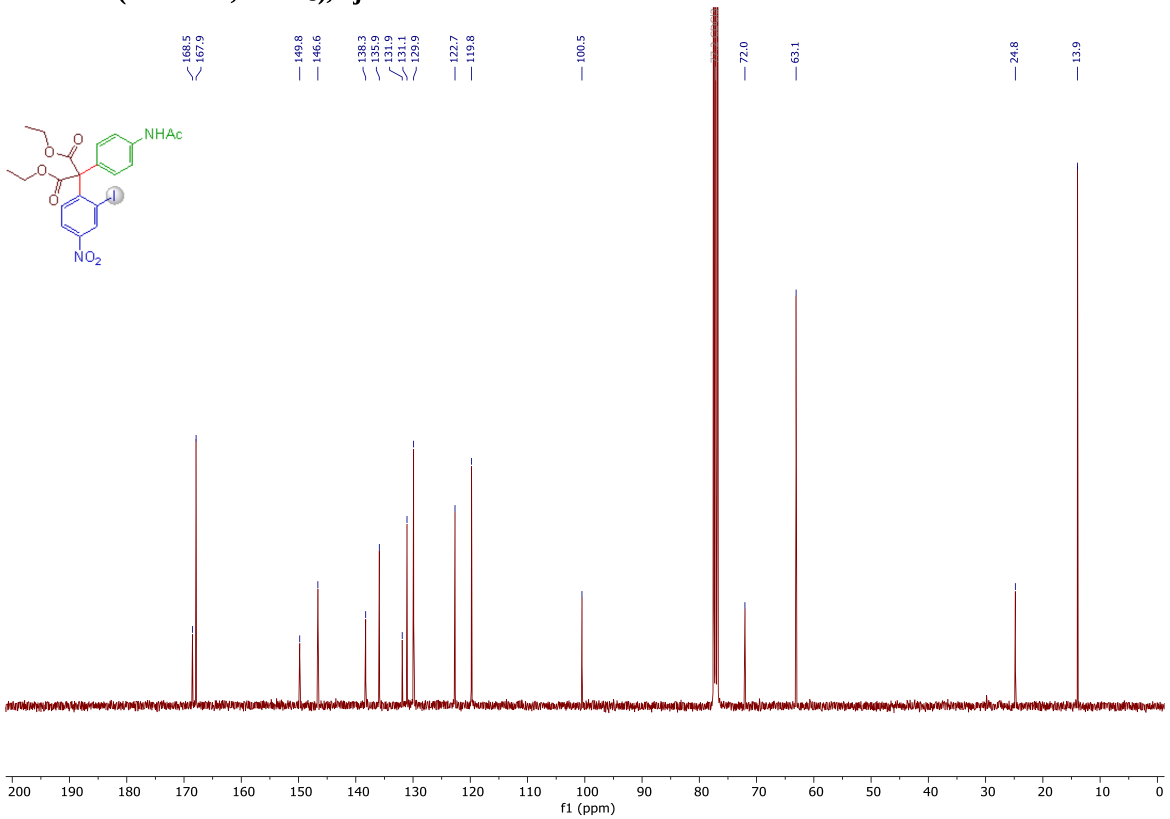

^{1}H NMR (400 MHz, CDCl$_3$), 3k:

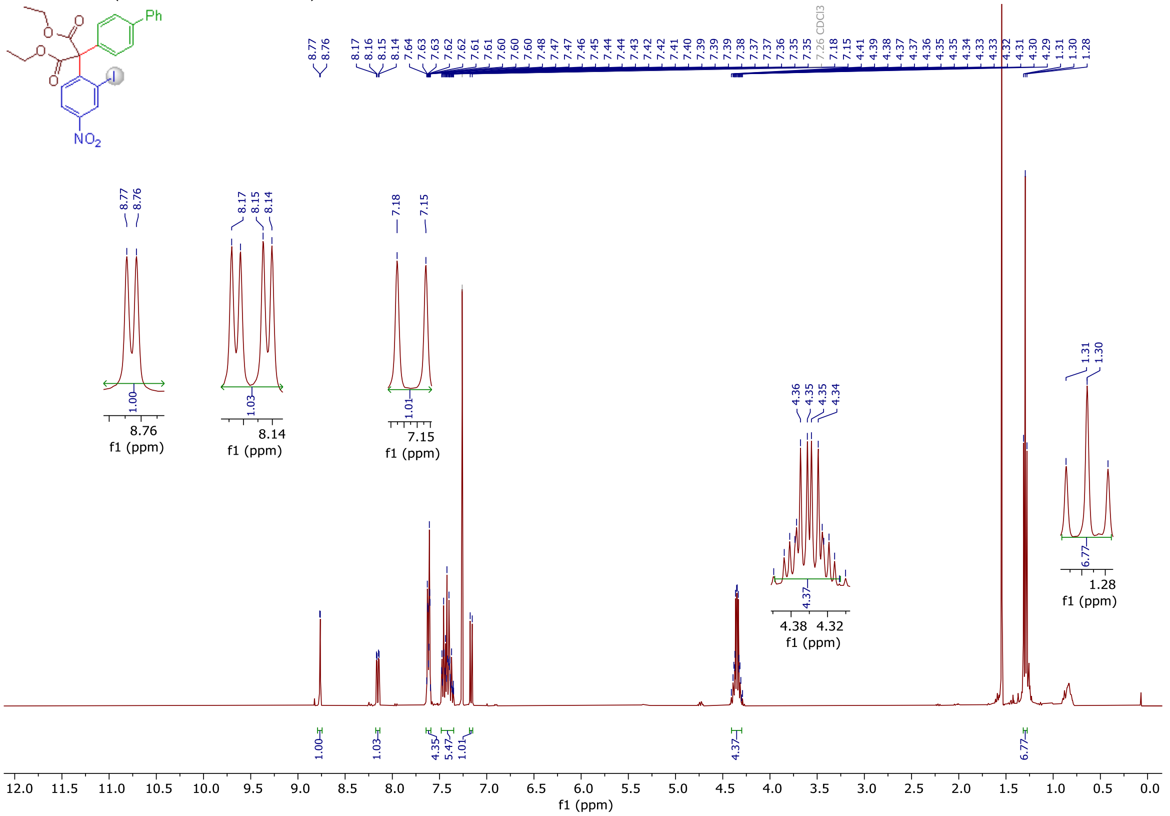

^{13}C NMR (101 MHz, CDCl3), 3k:

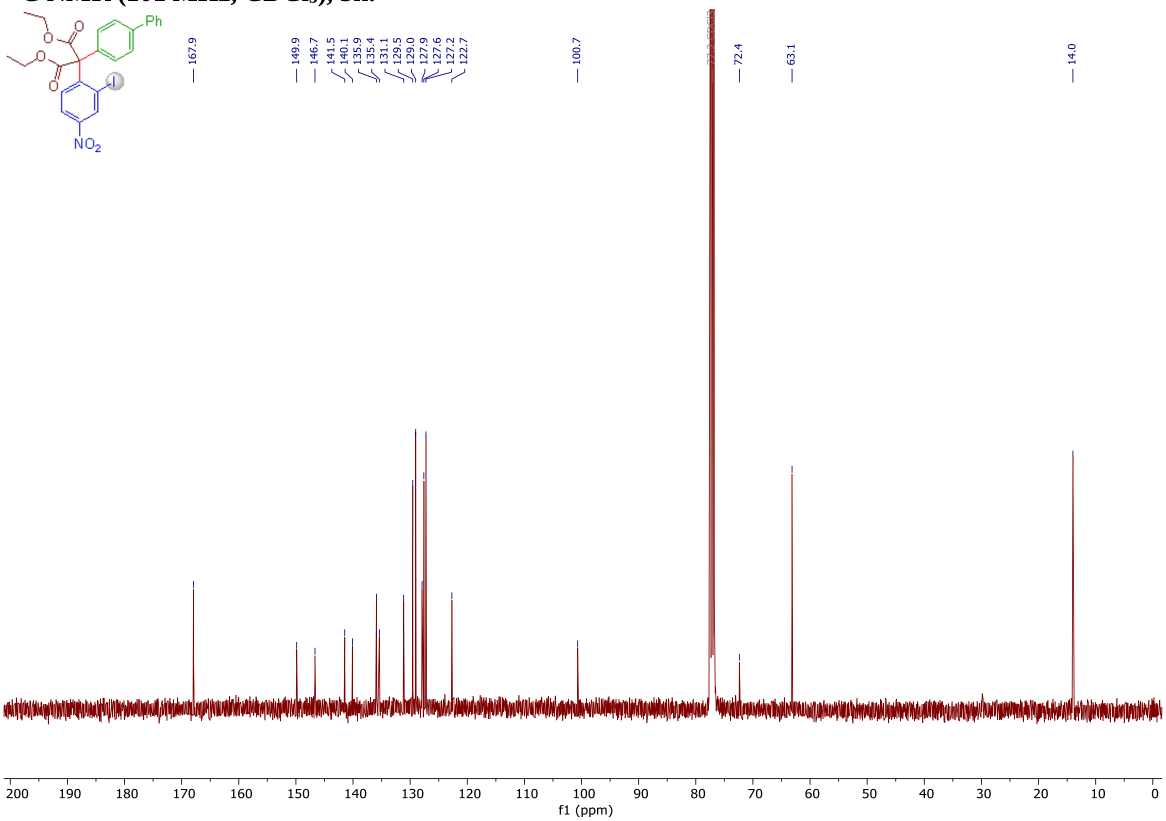

^{1}H NMR (400 MHz, CDCl3), 3l:

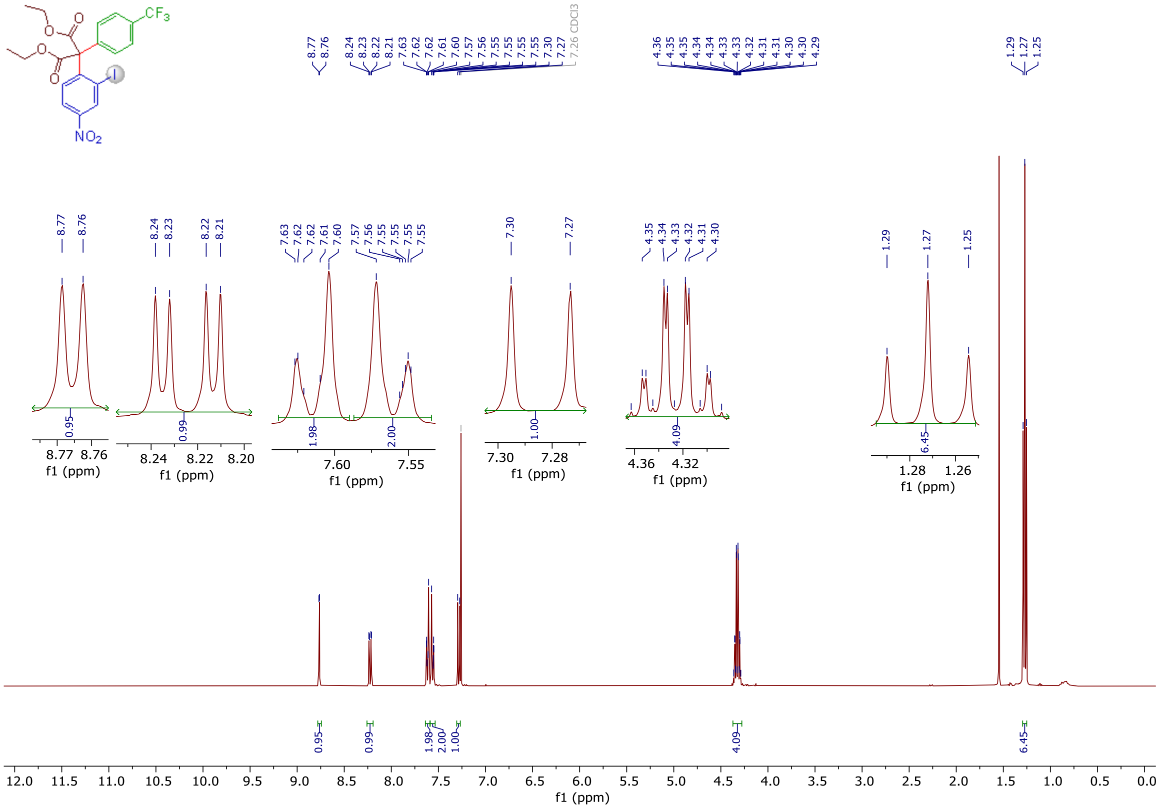

^{13}C NMR (101 MHz, $CDCl_3$), 3l:

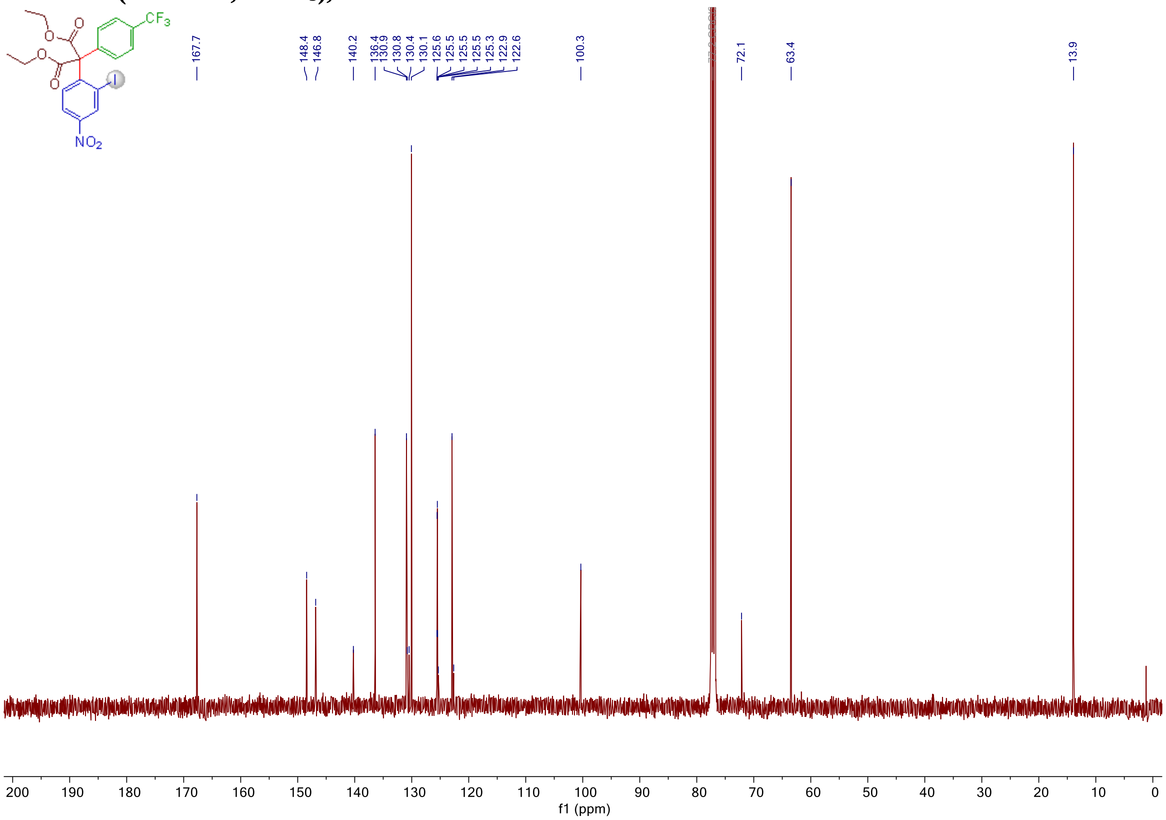

^{19}F NMR (377 MHz, $CDCl_3$), 3l:

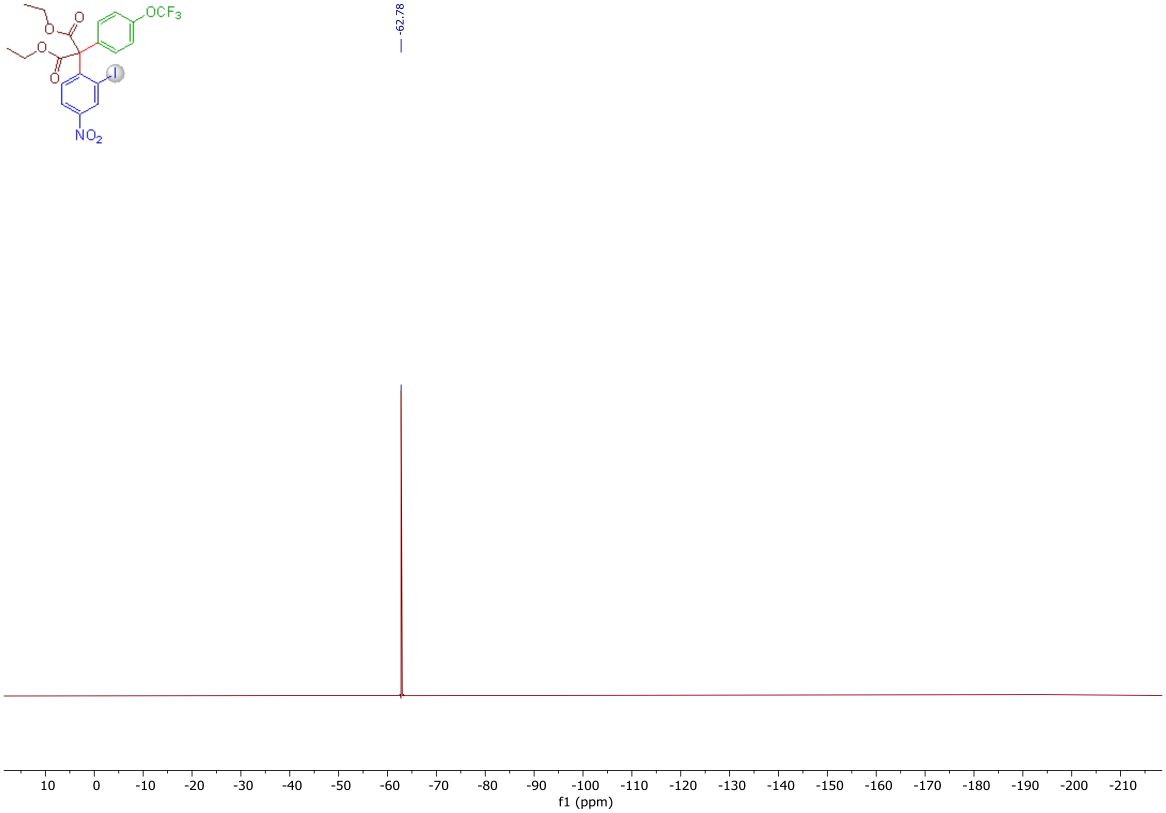

^{1}H NMR (400 MHz, $CDCl_3$), 3m:

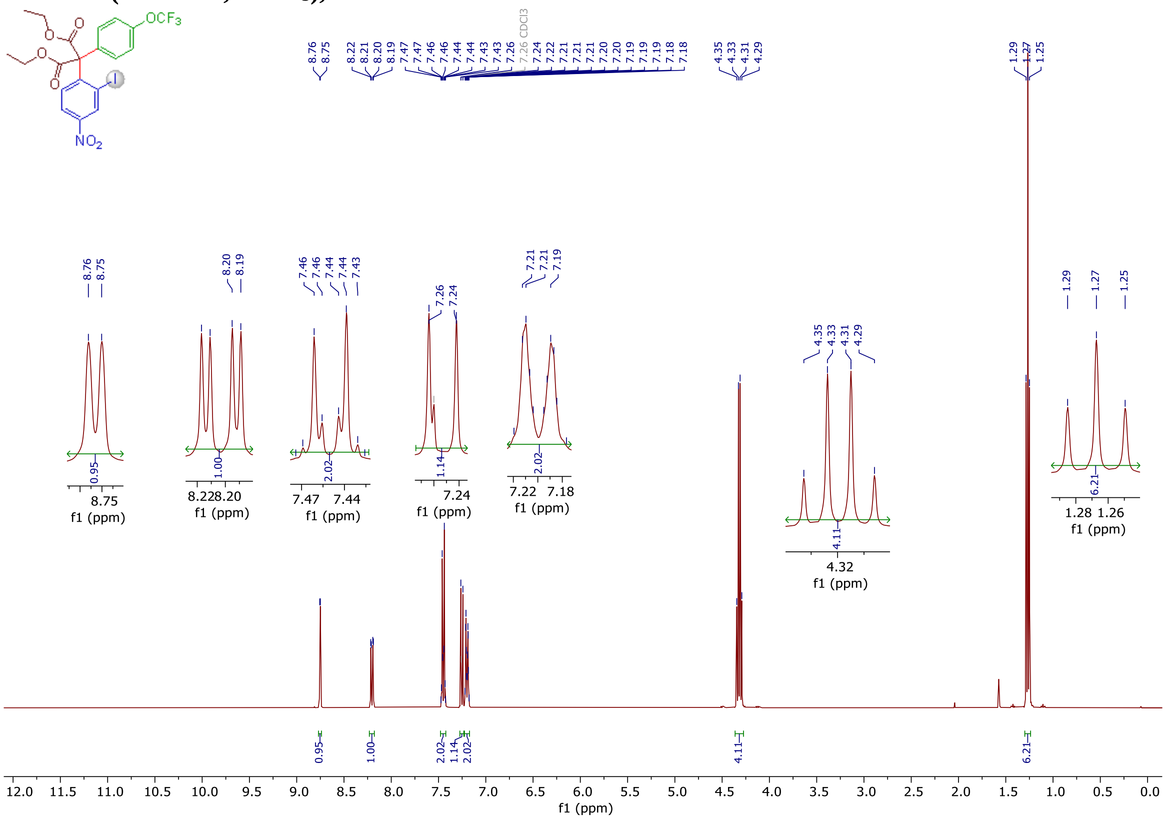

^{13}C NMR (101 MHz, $CDCl_3$), 3m:

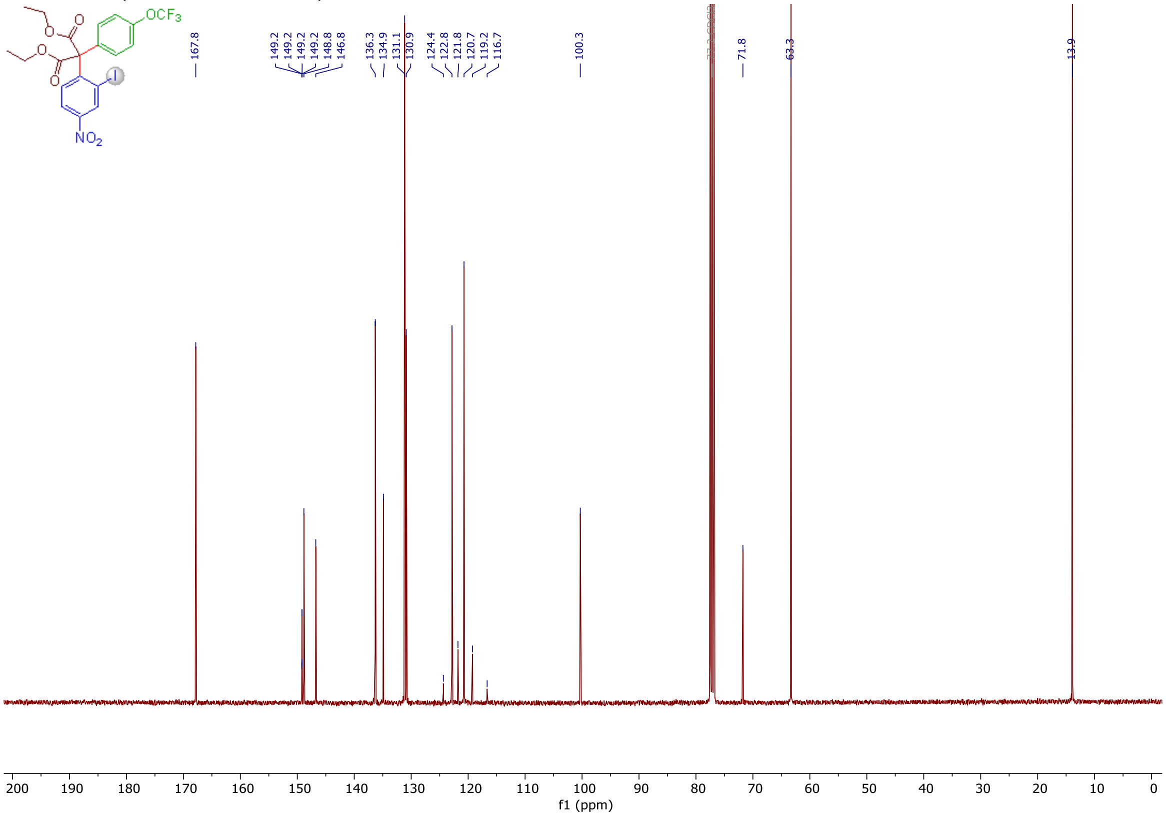

^{19}F NMR (377 MHz, $CDCl_3$), 3m:

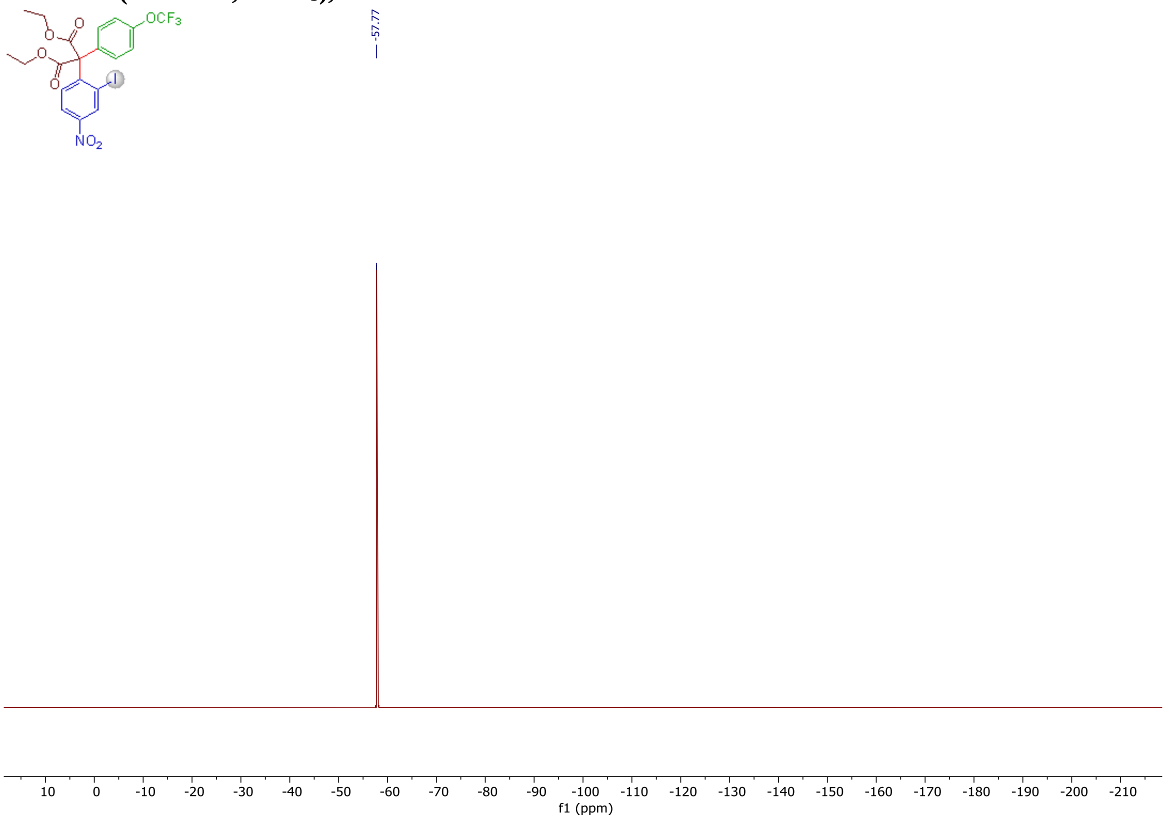

^{1}H NMR (400 MHz, $CDCl_3$), 3n:

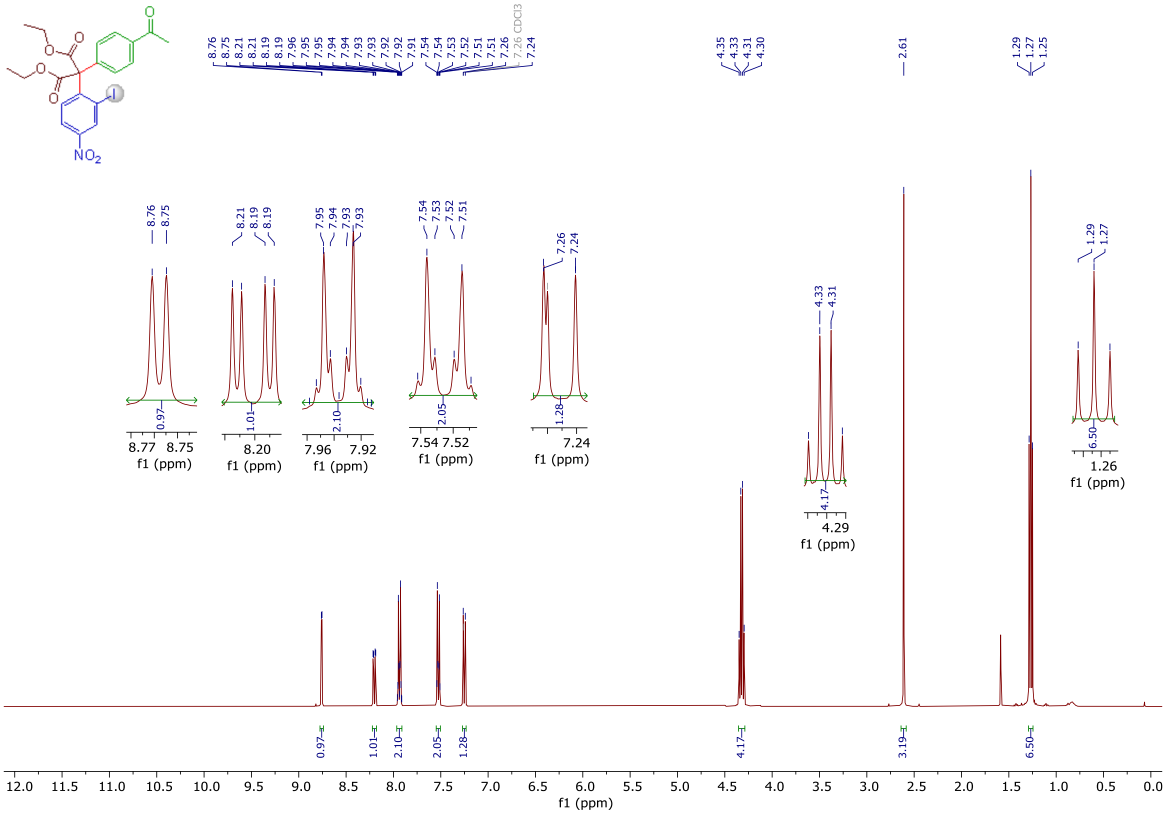

^{13}C NMR (101 MHz, CDCl$_3$), 3n:

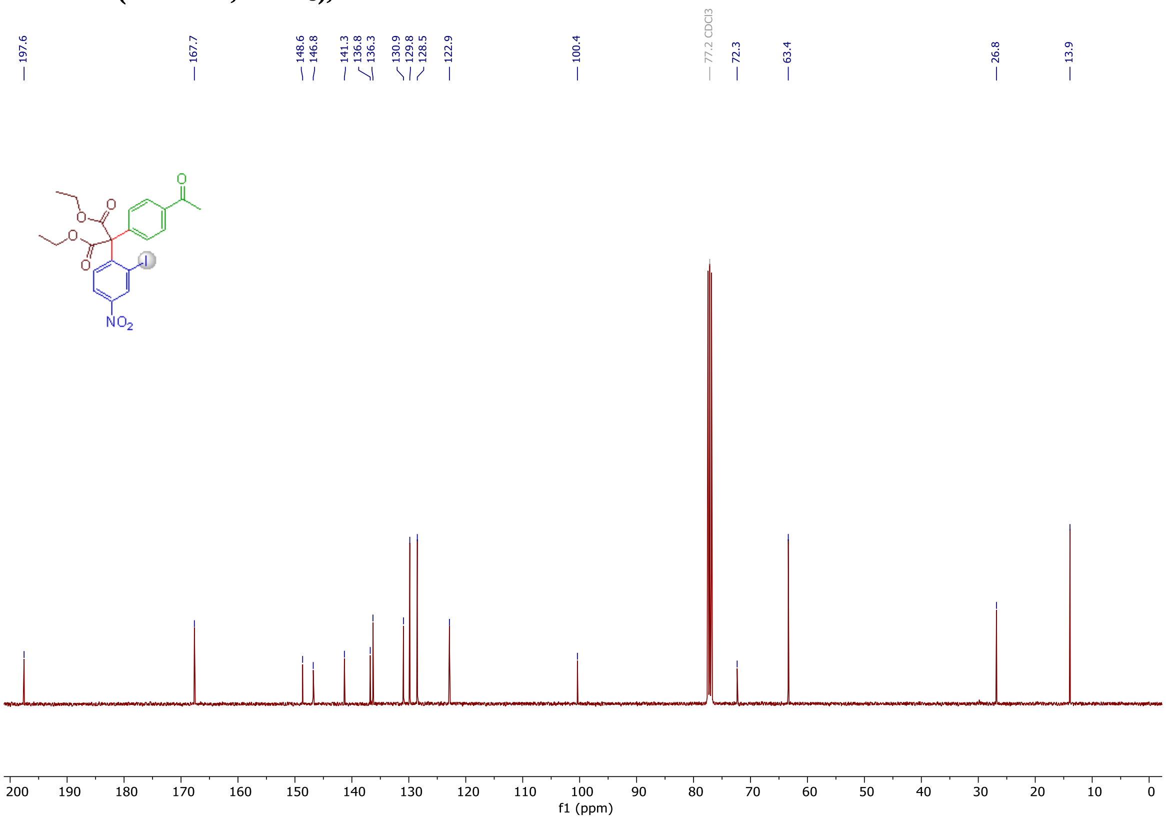

^{1}H NMR (400 MHz, CDCl$_3$), 3o:

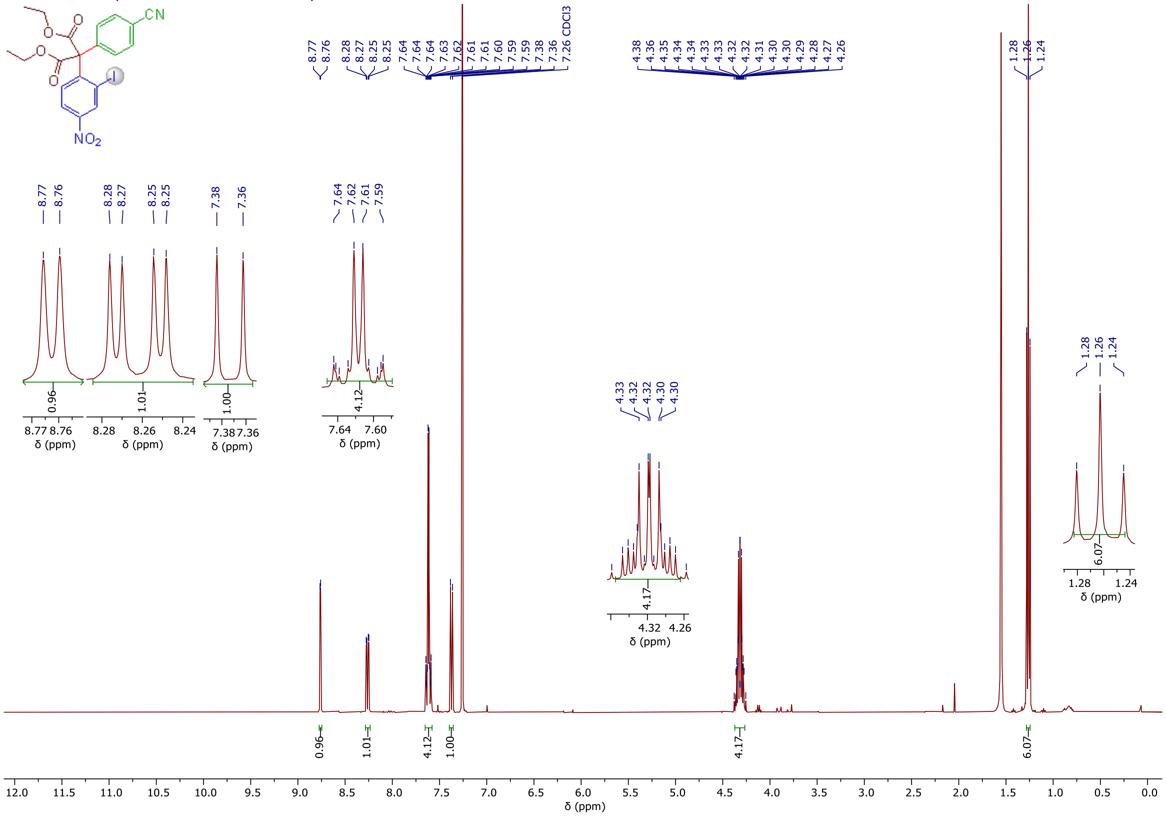

^{13}C NMR (101 MHz, CDCl$_3$), 3o:

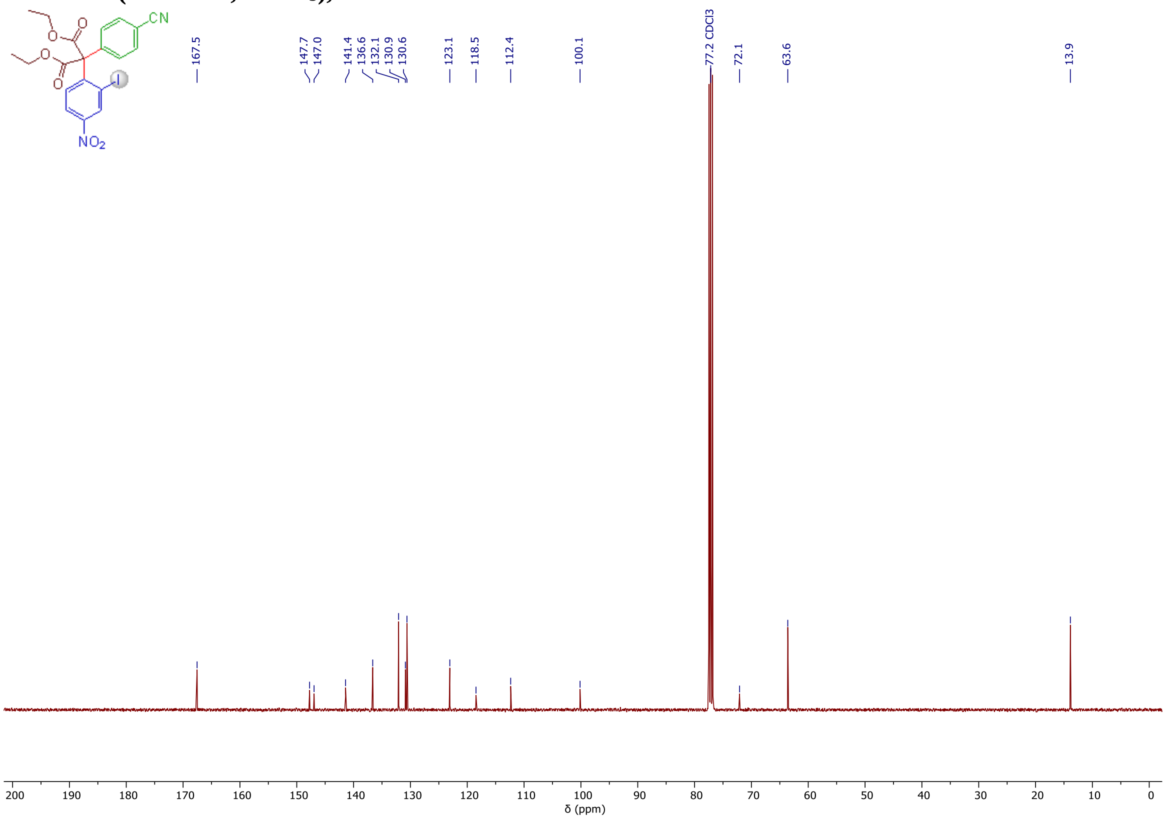

^{1}H NMR (400 MHz, CDCl$_3$), 3p:

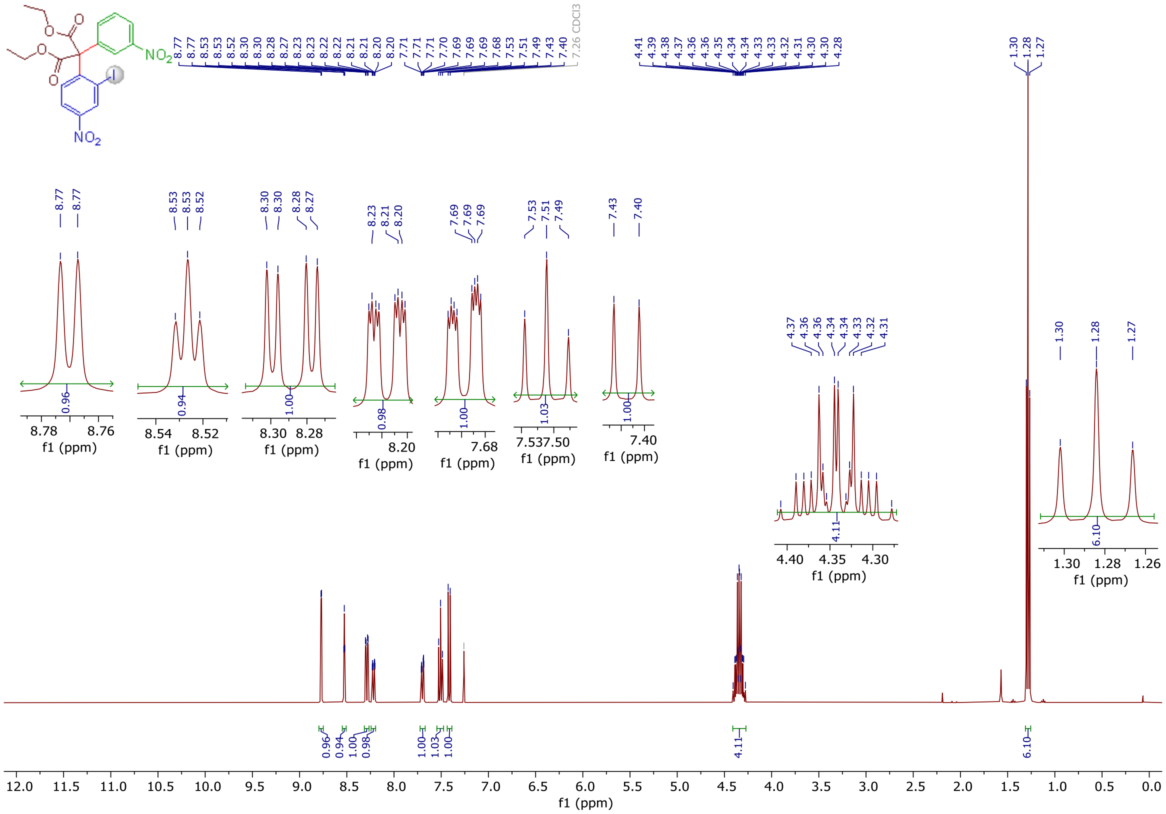

^{13}C NMR (101 MHz, $CDCl_3$), 3p:

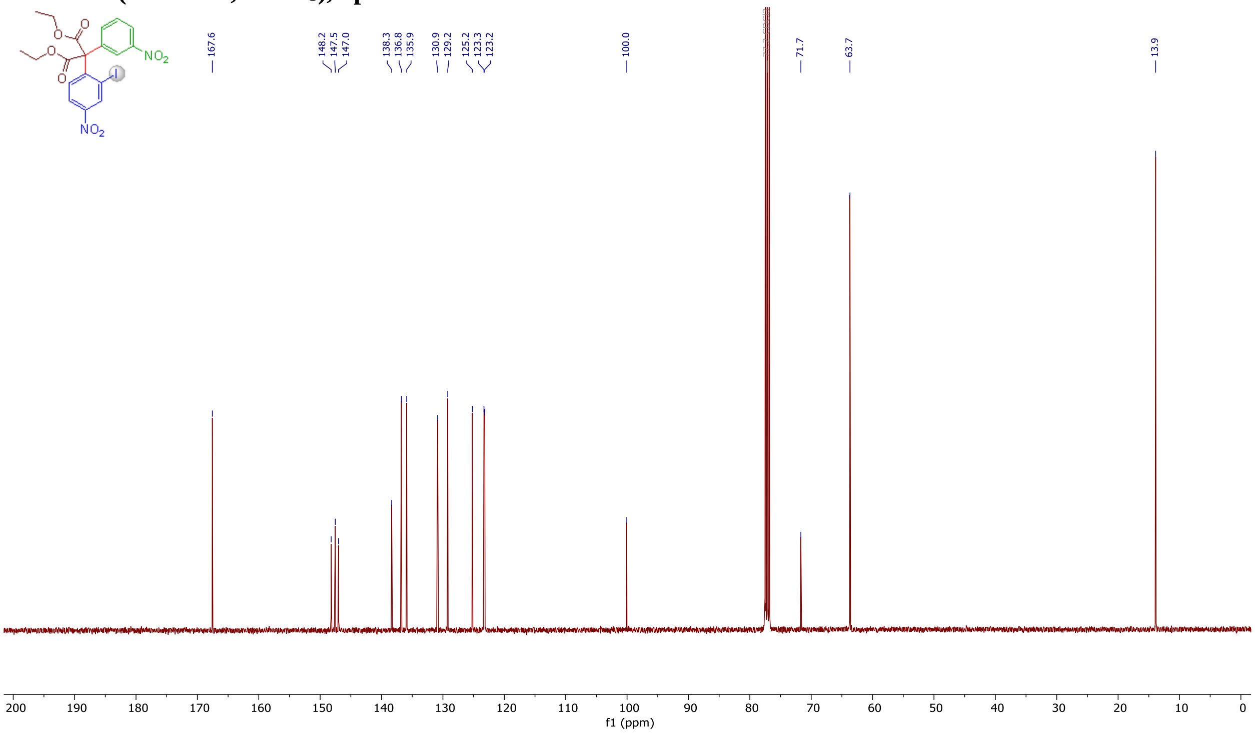

^{1}H NMR (400 MHz, $CDCl_3$), 3q:

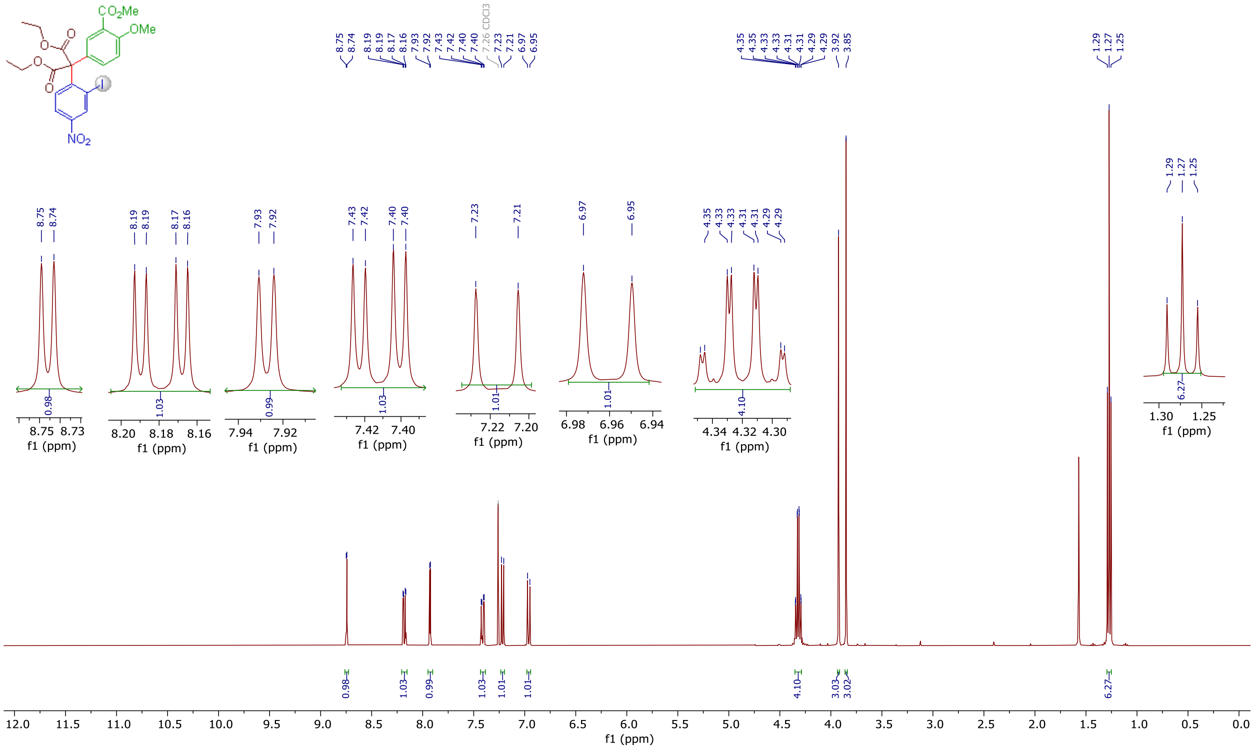

^{13}C NMR (101 MHz, $CDCl_3$), 3q:

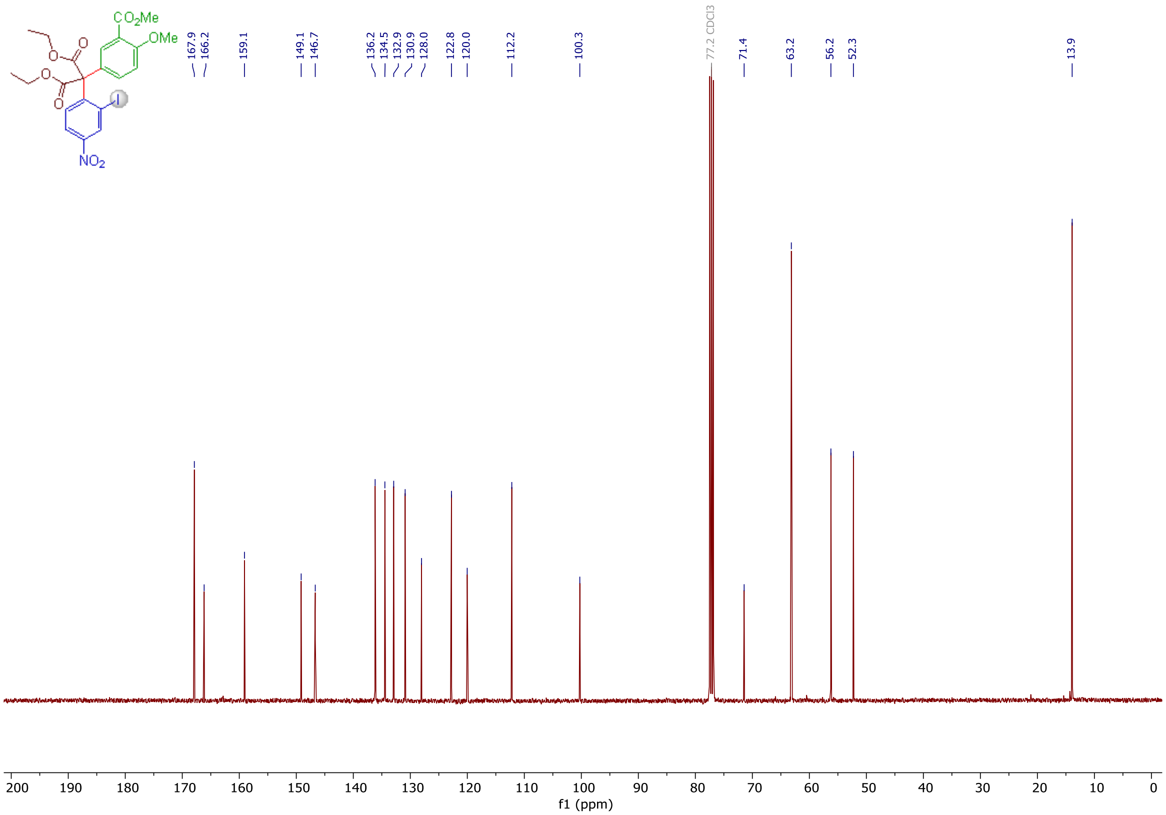

^{1}H NMR (400 MHz, $CDCl_3$), 3r:

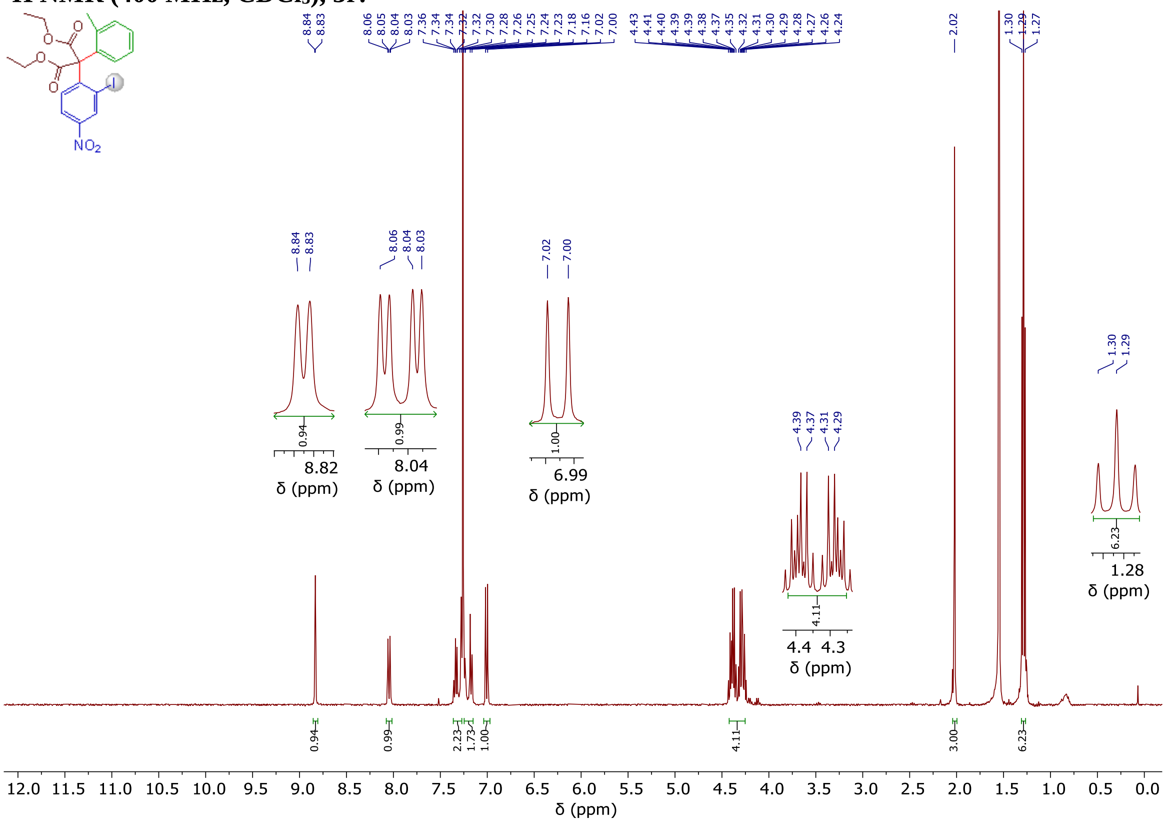

^{13}C NMR (101 MHz, CDCl$_3$), 3r:

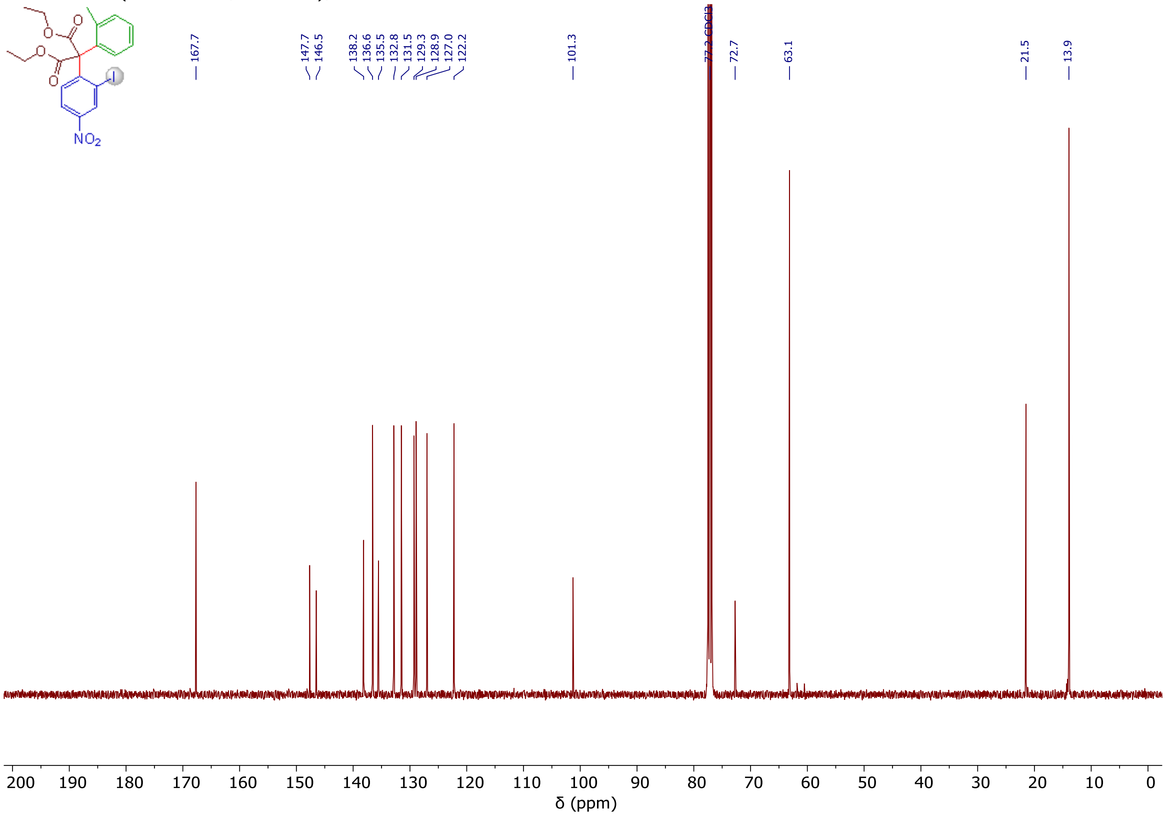

^{1}H NMR (400 MHz, CDCl$_3$), 3s:

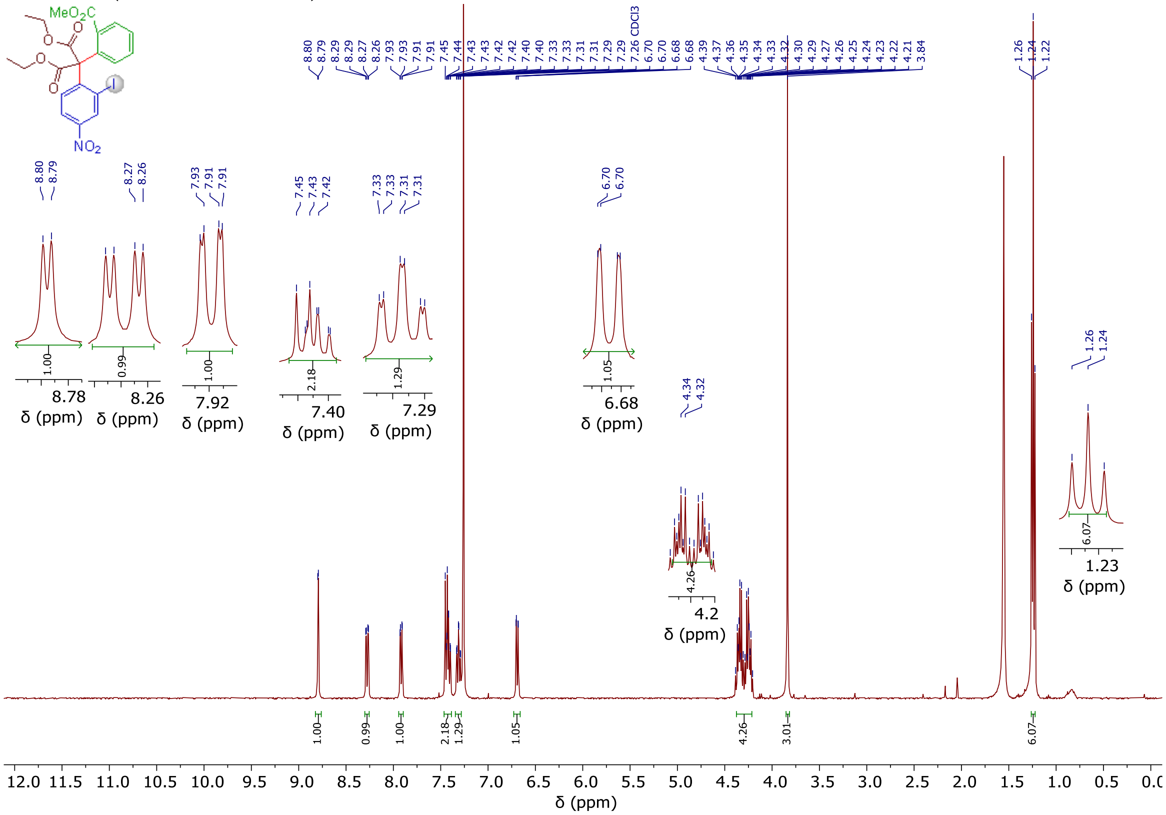

^{13}C NMR (101 MHz, CDCl3), 3s:

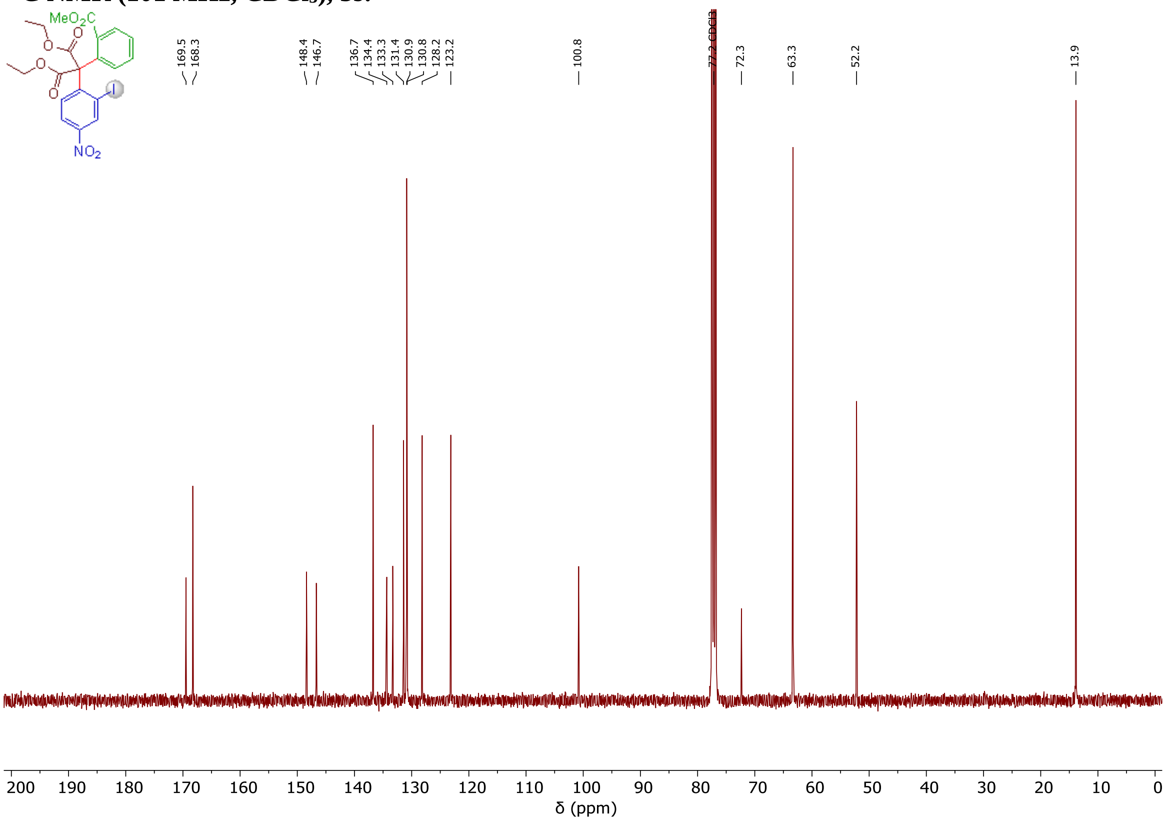

^{1}H NMR (400 MHz, CDCl3), 3t:

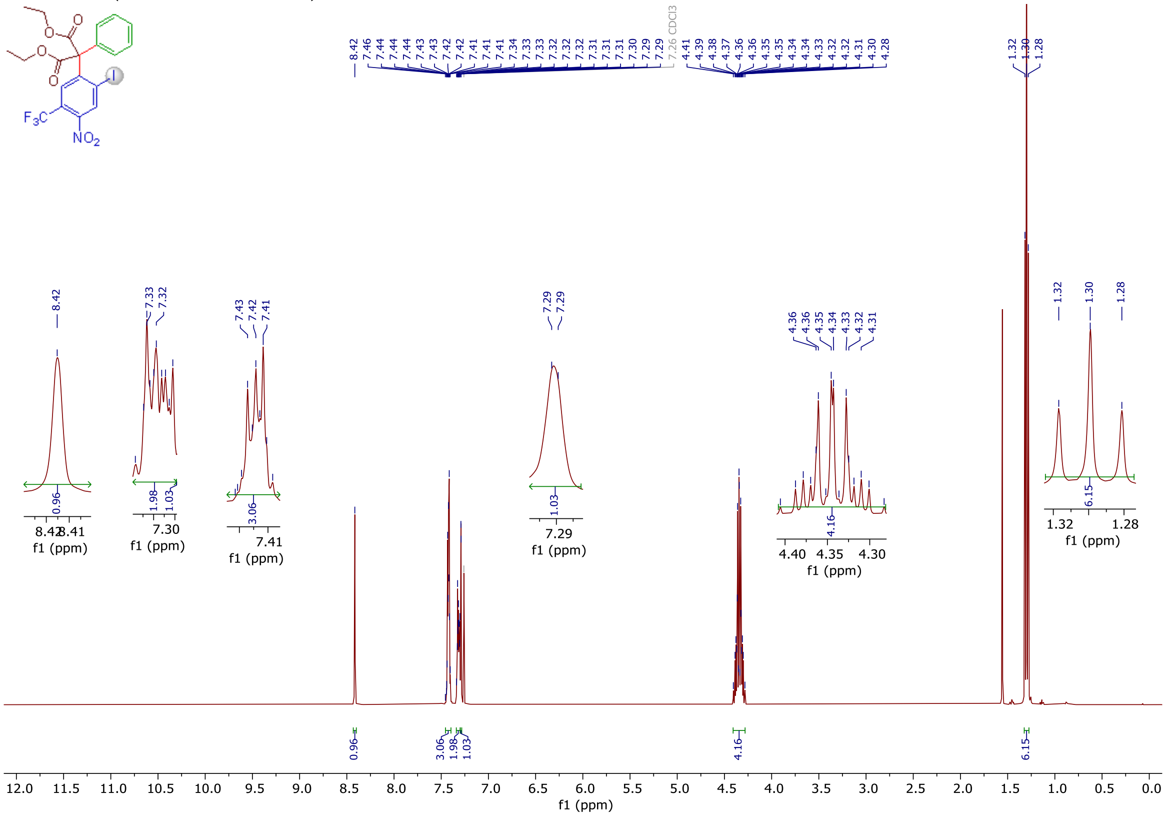

^{13}C NMR (101 MHz, CDCl3), 3t:

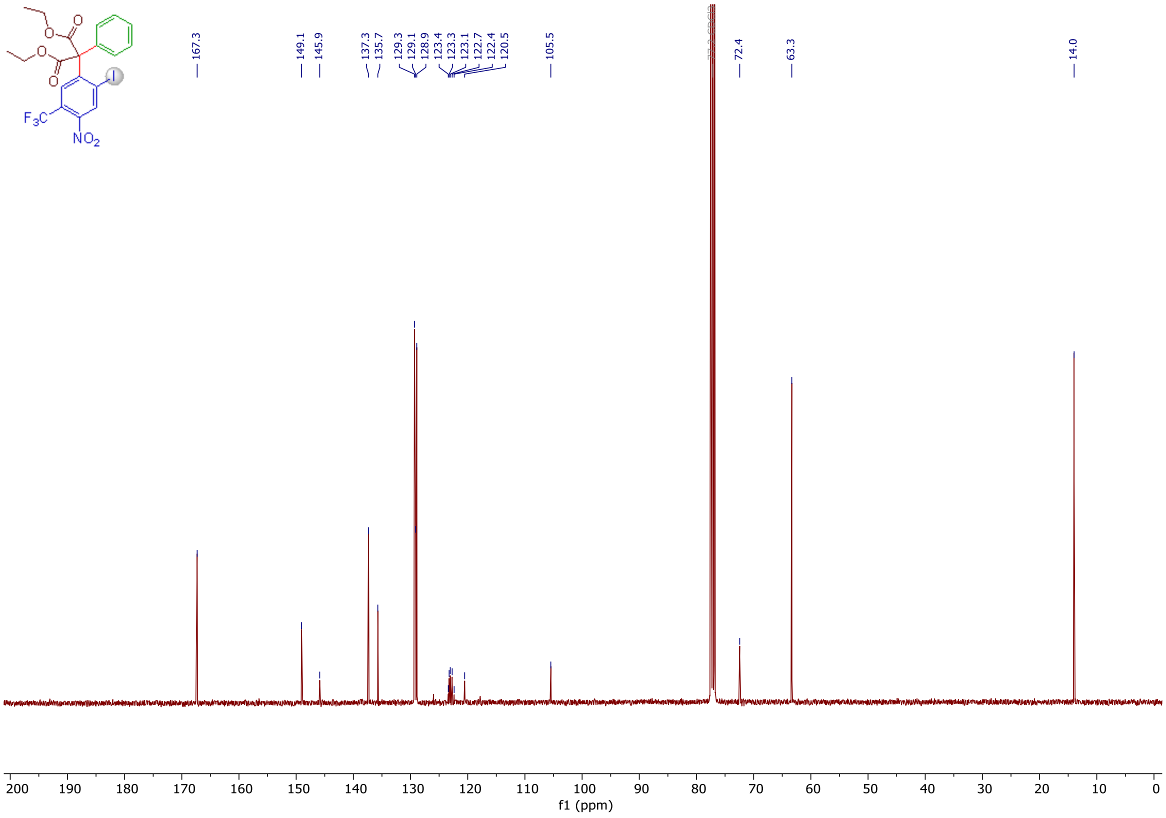

^{19}F NMR (101 MHz, $CDCl_3$), 3t:

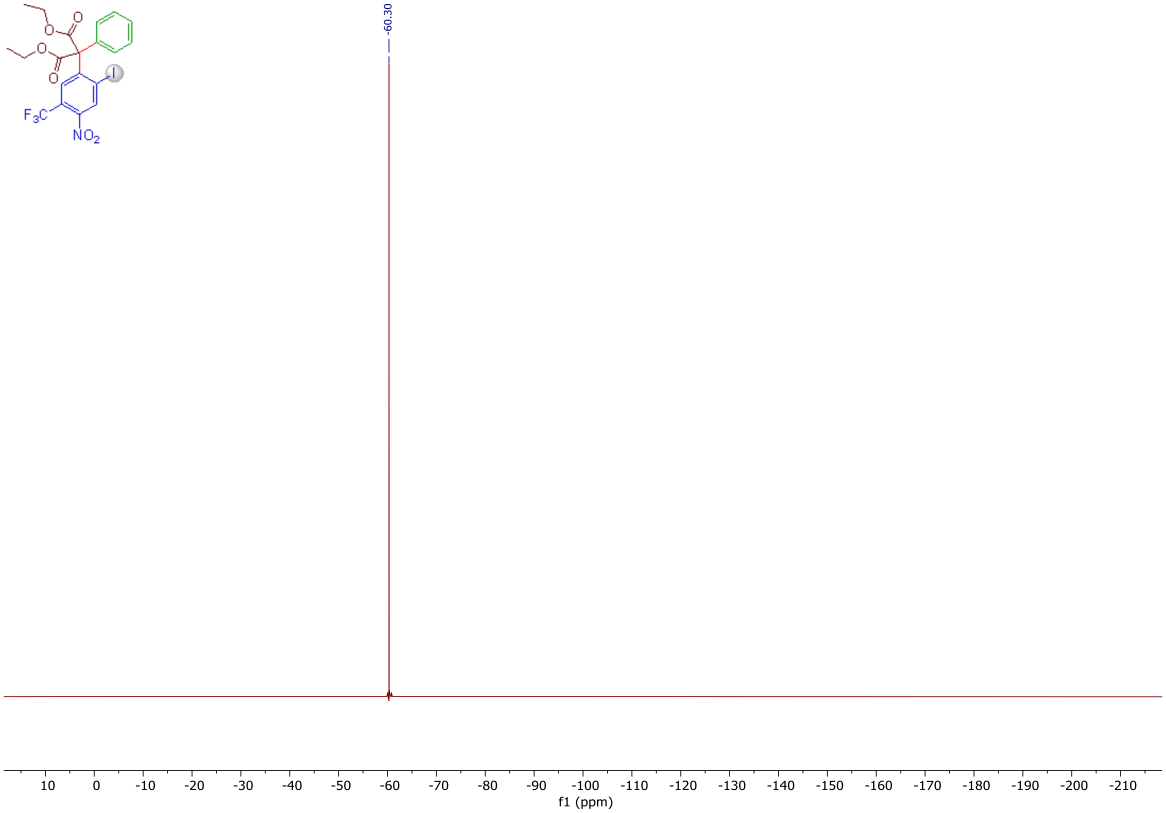

^{1}H NMR (400 MHz, $CDCl_3$), 3u:

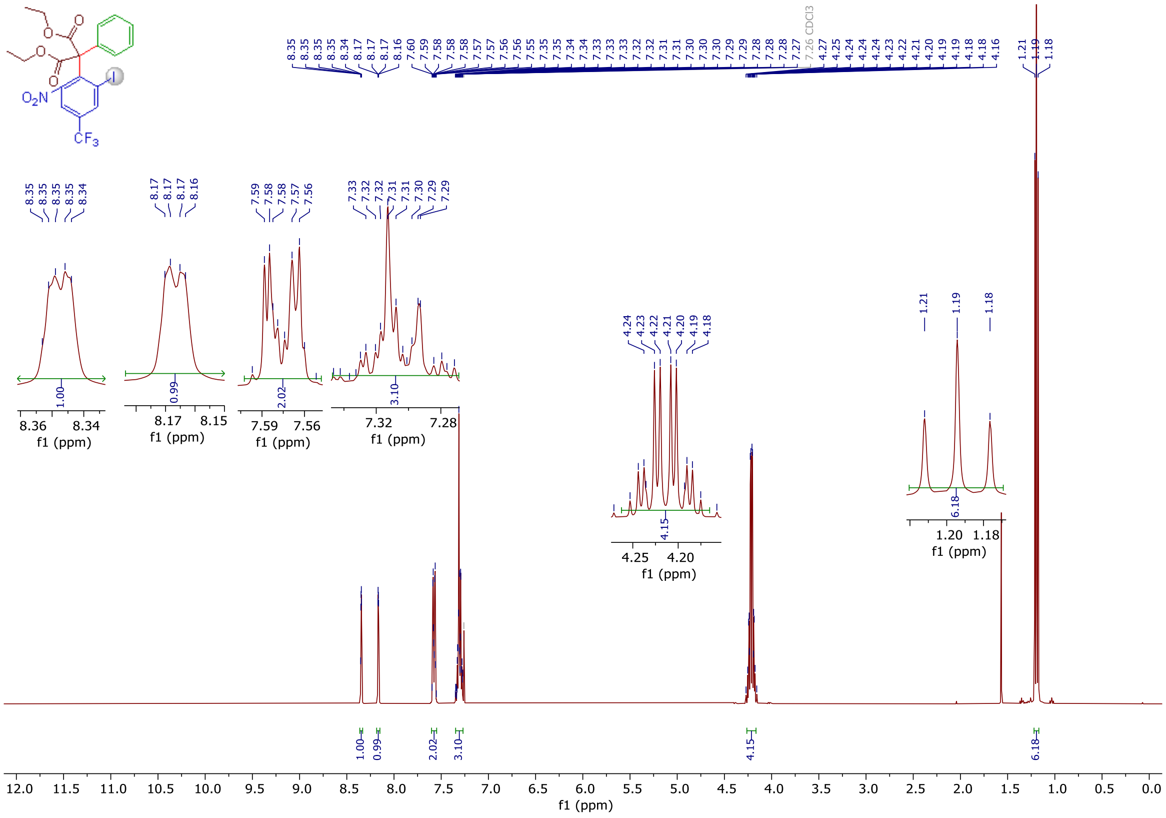

^{13}C NMR (101 MHz, CDCl3), 3u:

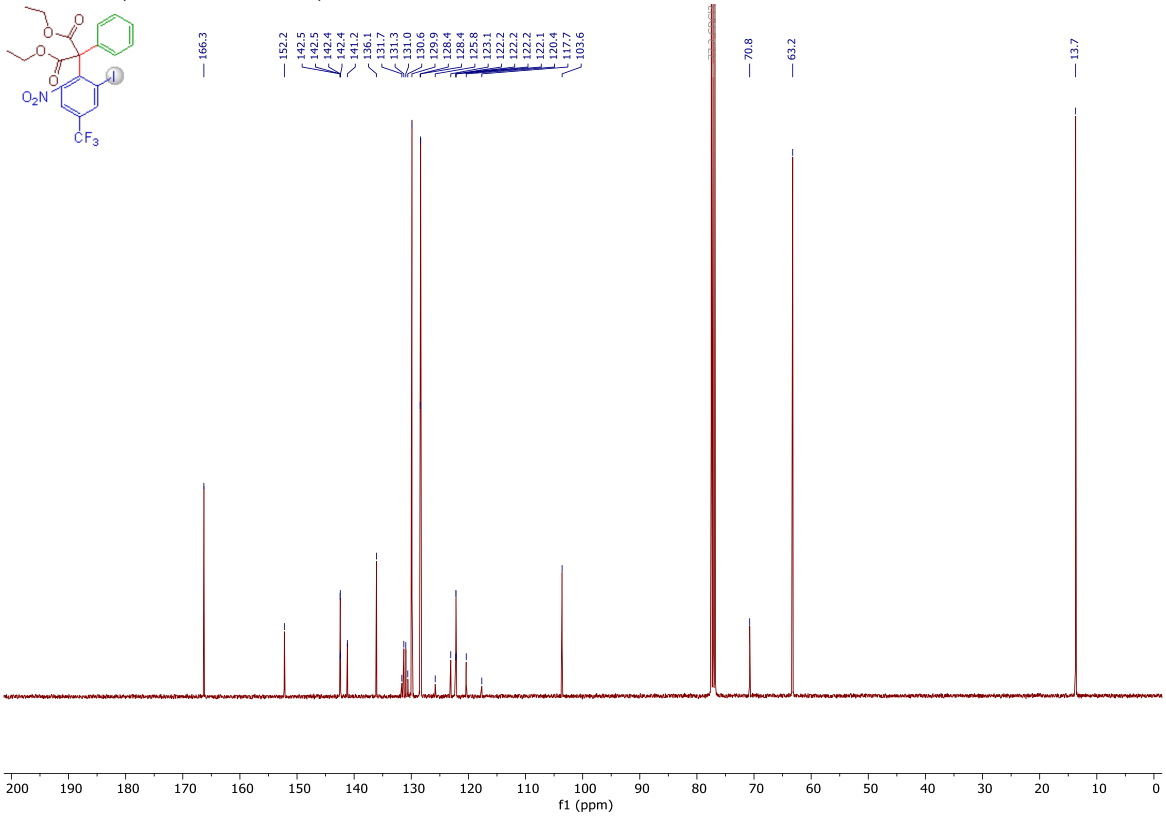

^{19}F NMR (377 MHz, CDCl3), 3u:

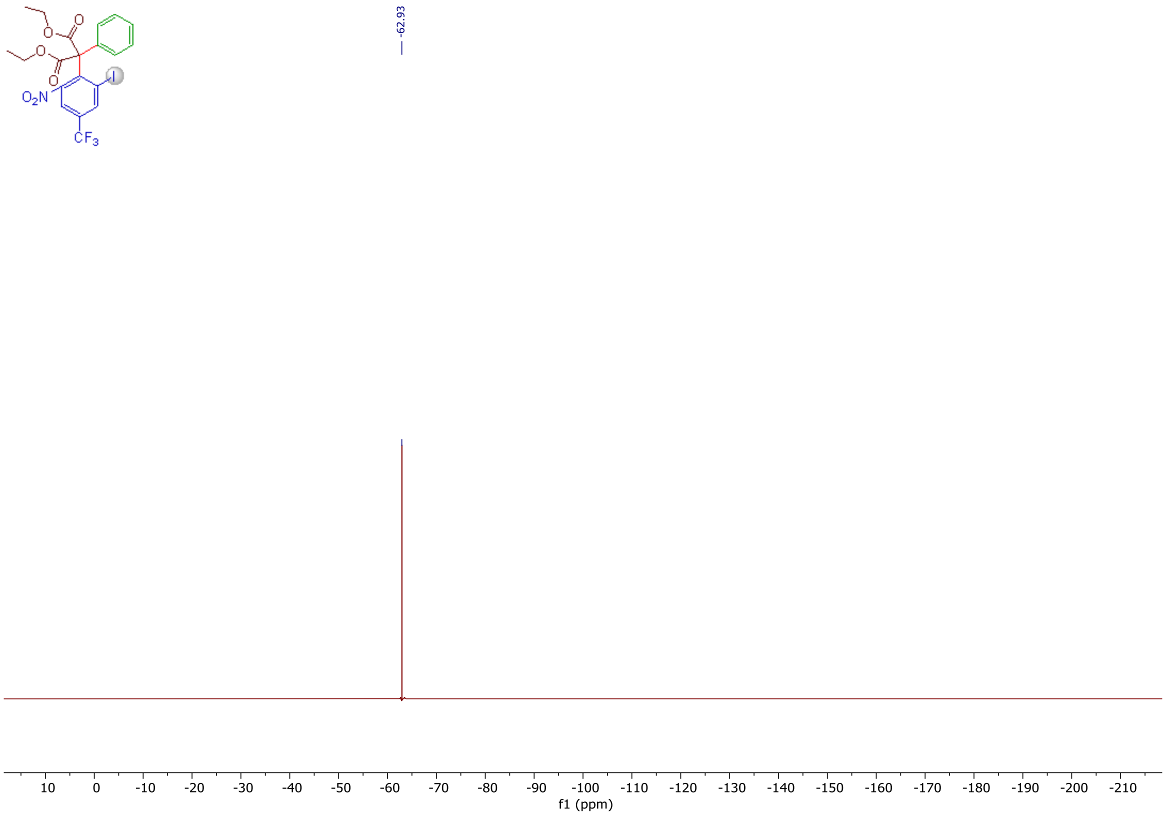

^{1}H NMR (400 MHz, CDCl₃), 3v:

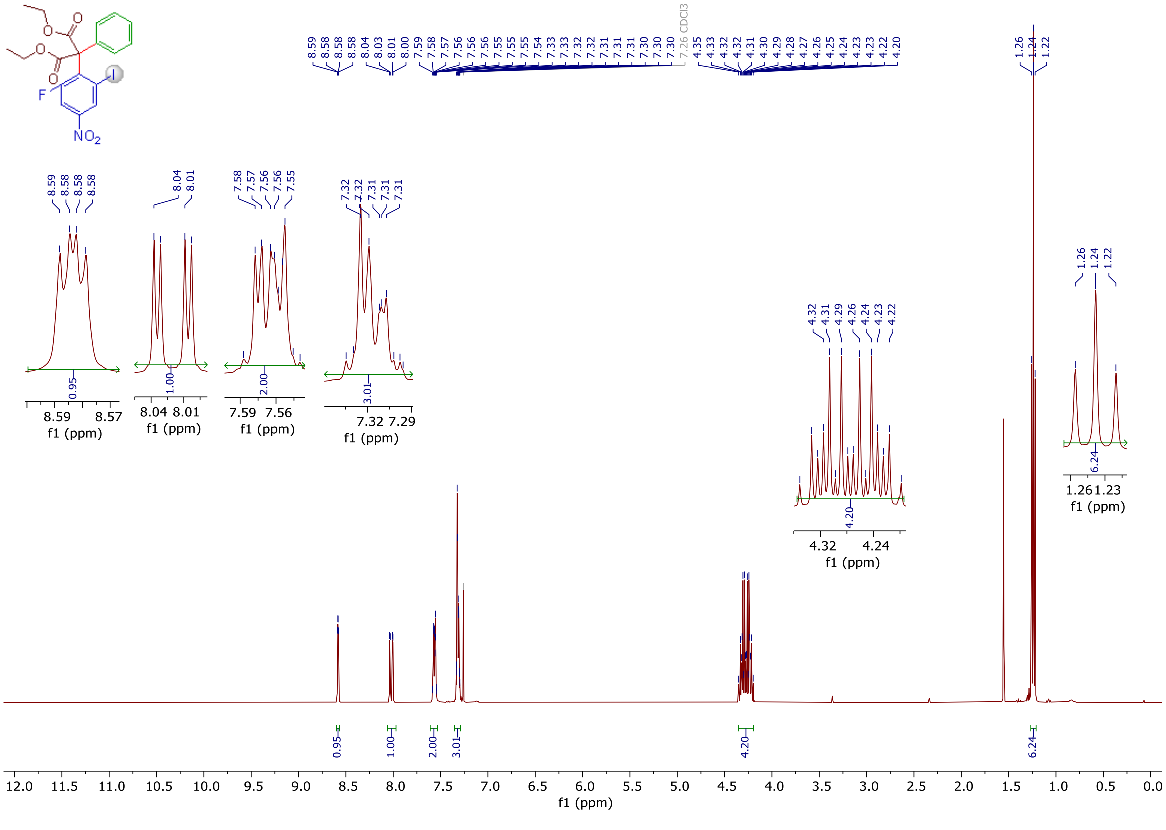

^{13}C NMR (101 MHz, CDCl3), 3v:

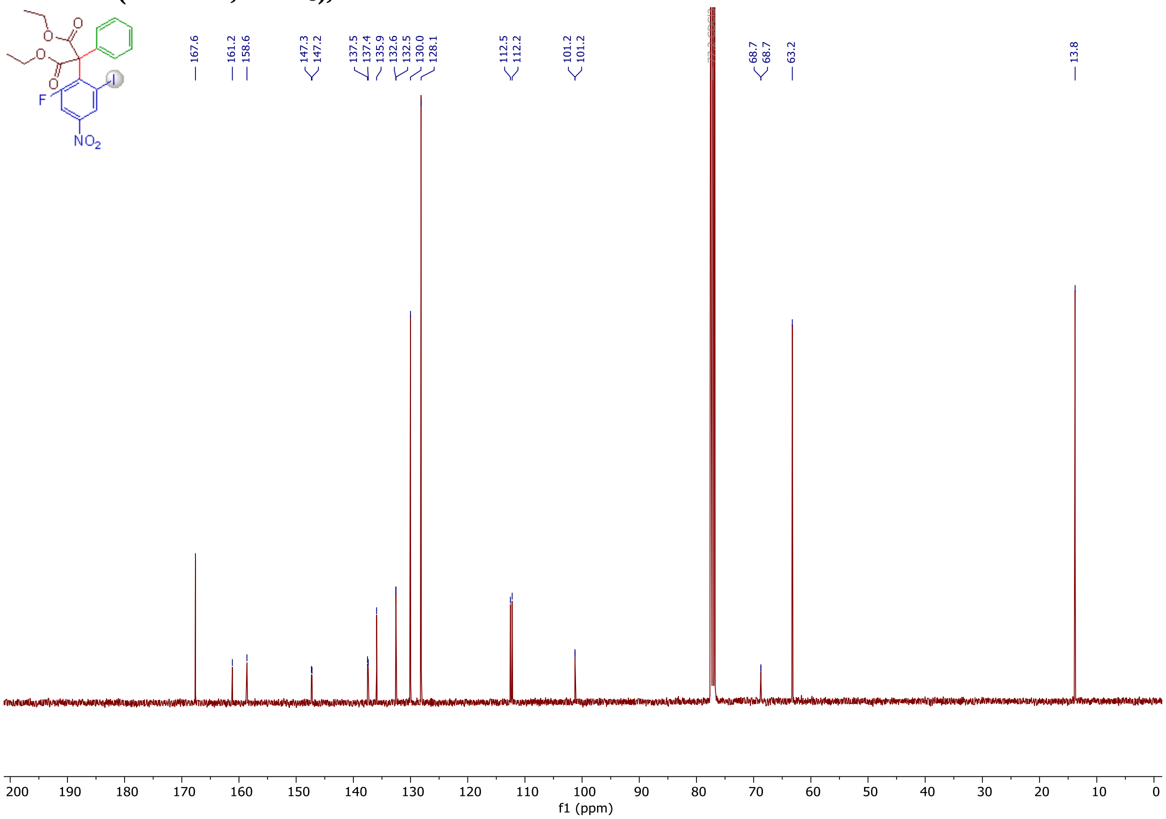

^{19}F NMR (377 MHz, CDCl3), 3v:

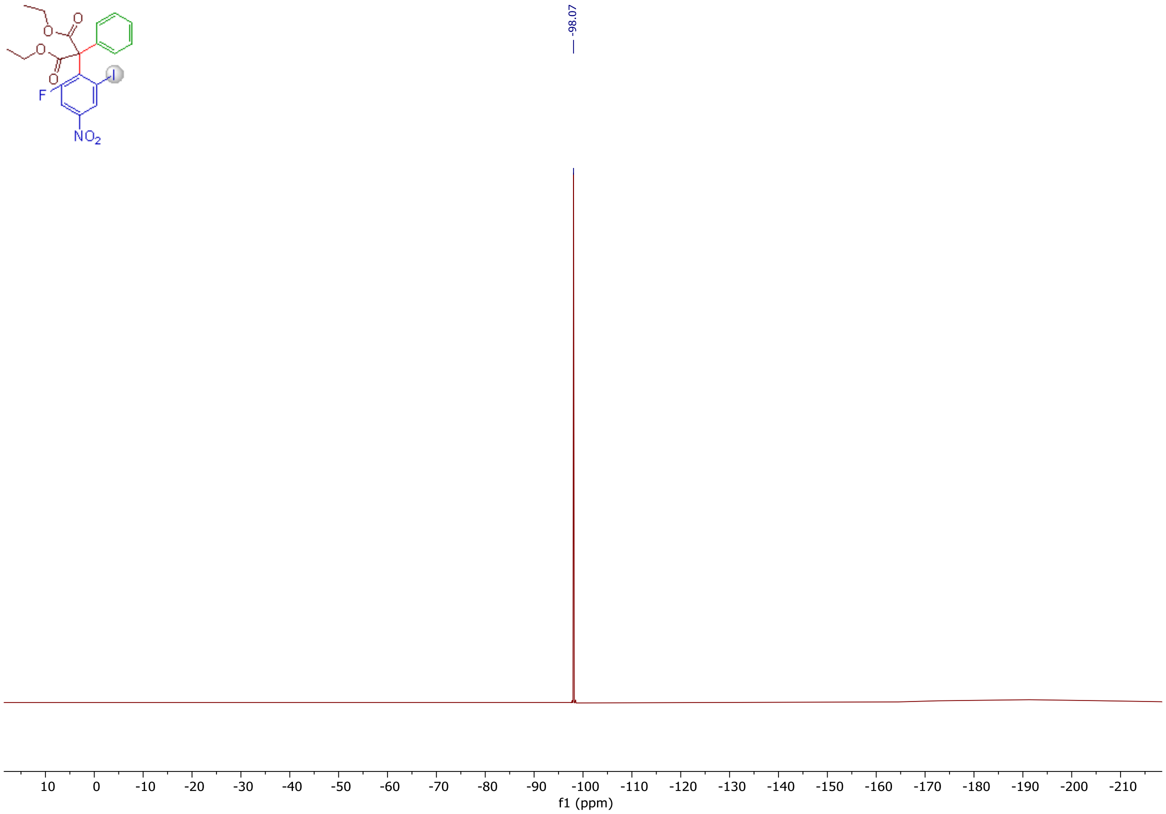

^{1}H NMR (400 MHz, CDCl3), 3w:

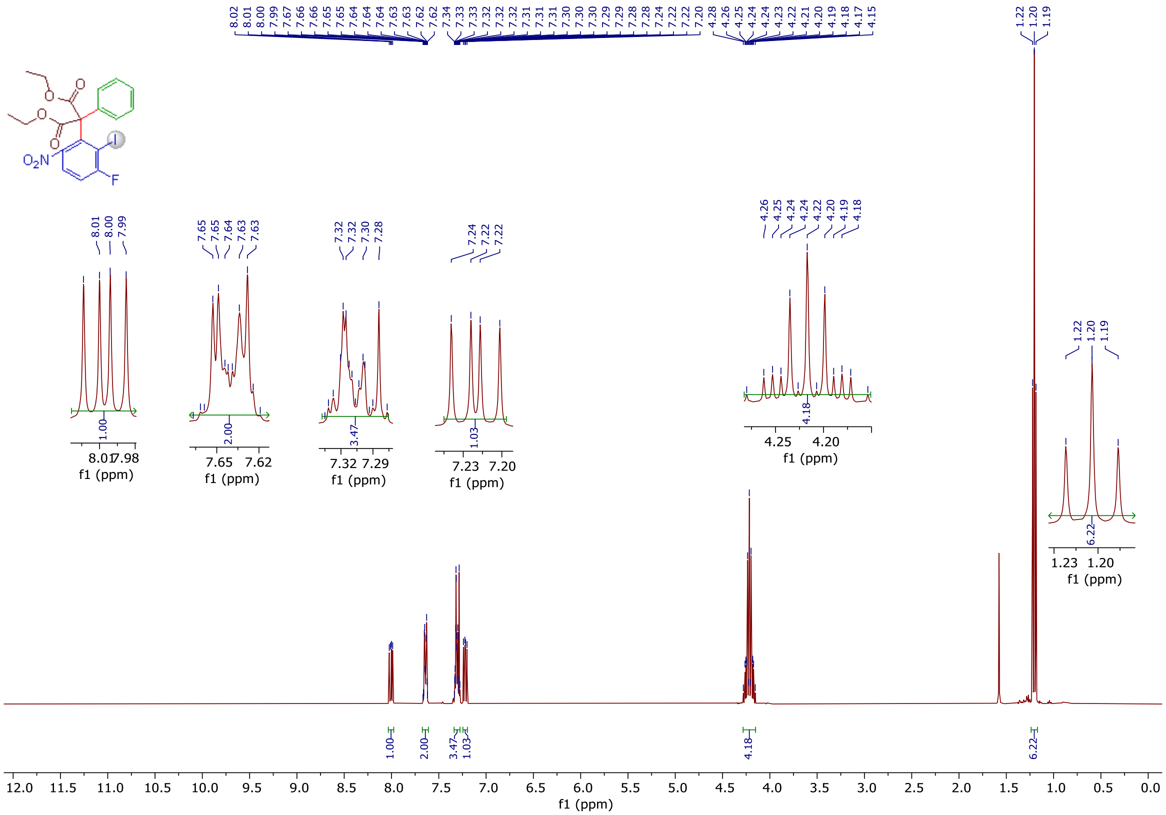

^{13}C NMR (101 MHz, $CDCl_3$), 3w:

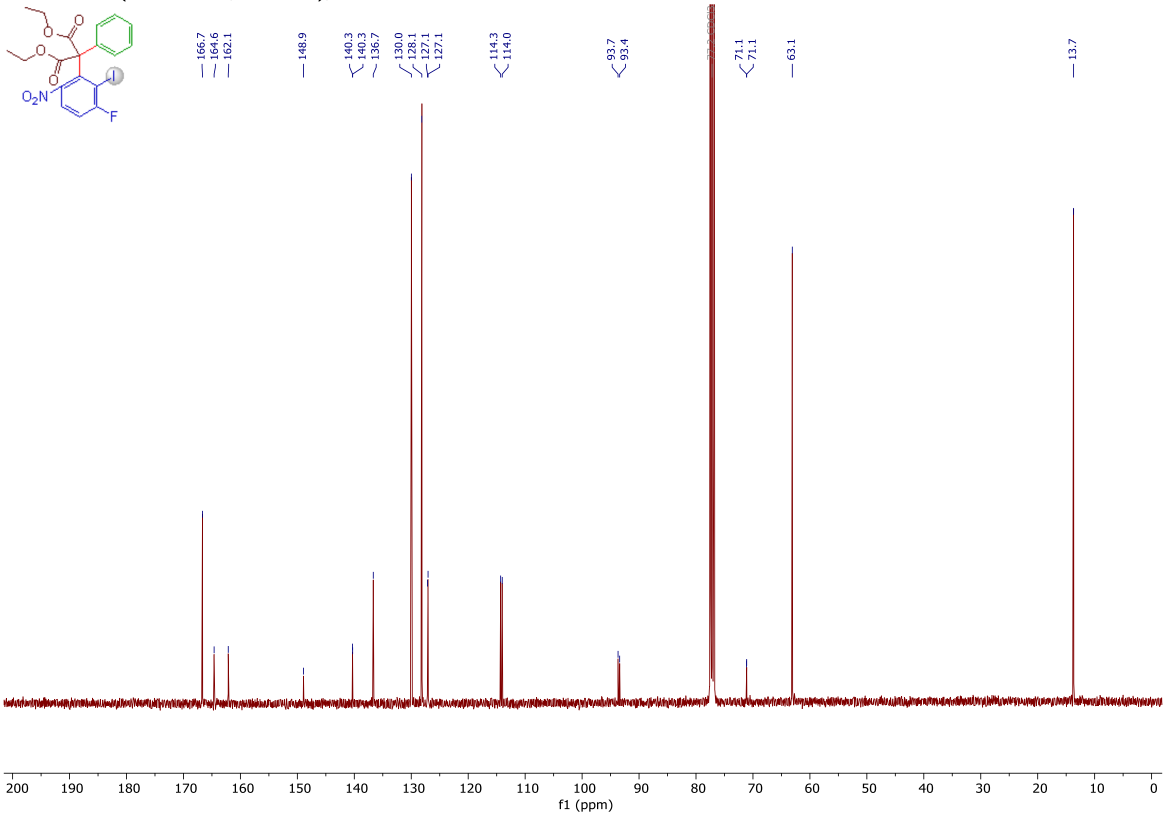

^{19}F NMR (101 MHz, $CDCl_3$), 3w:

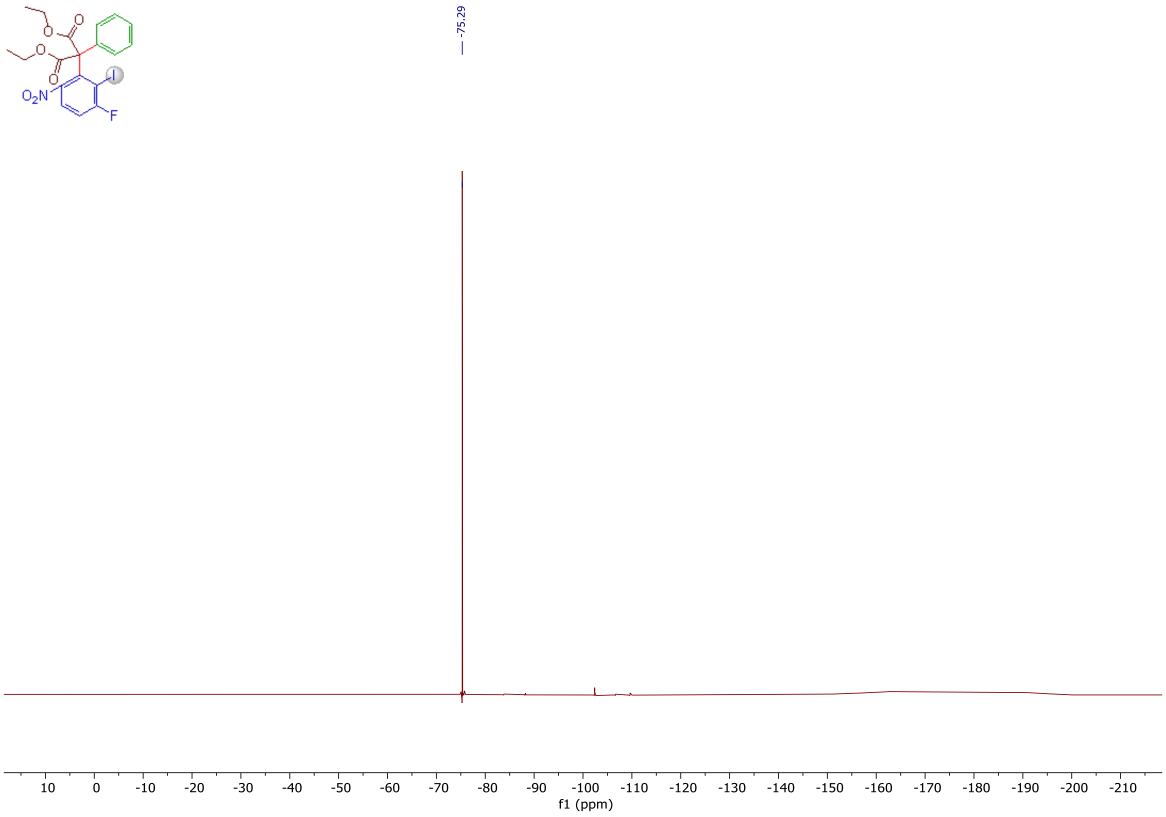

^{1}H NMR (400 MHz, CDCl$_3$), 3x:

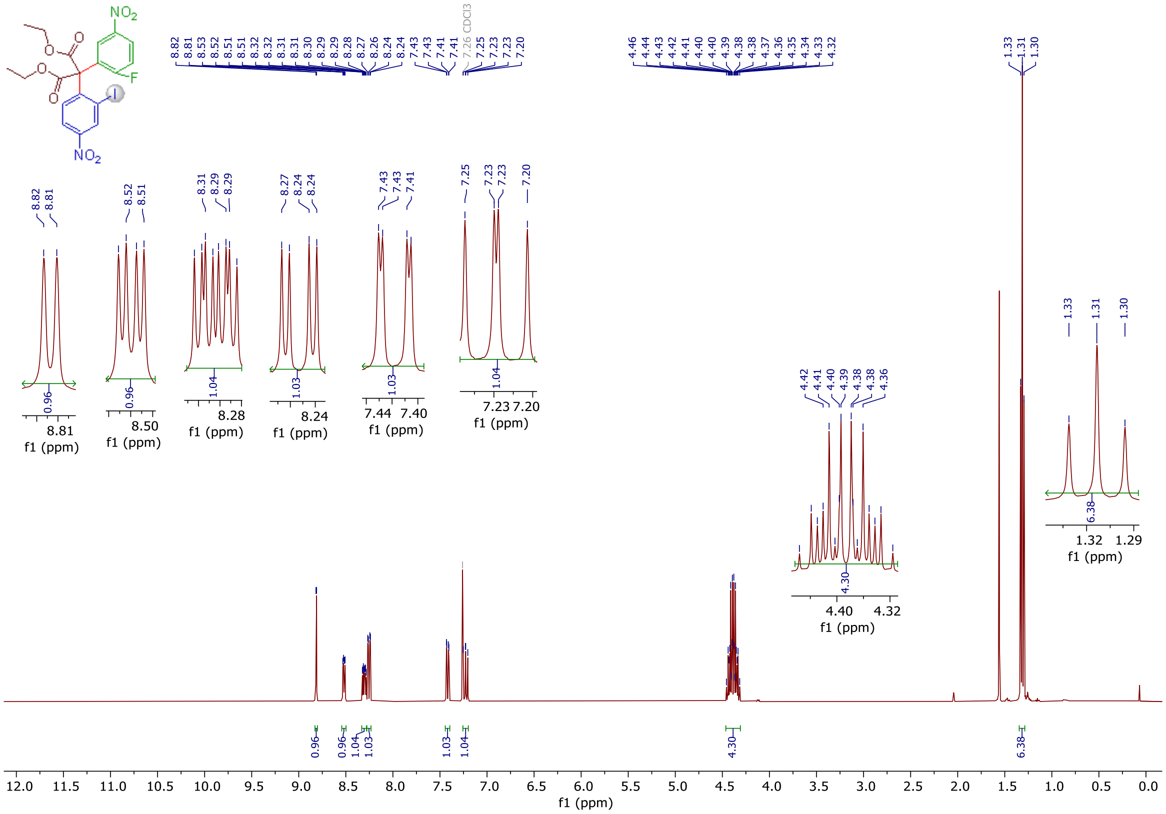

^{13}C NMR (101 MHz, CDCl$_3$), 3x:

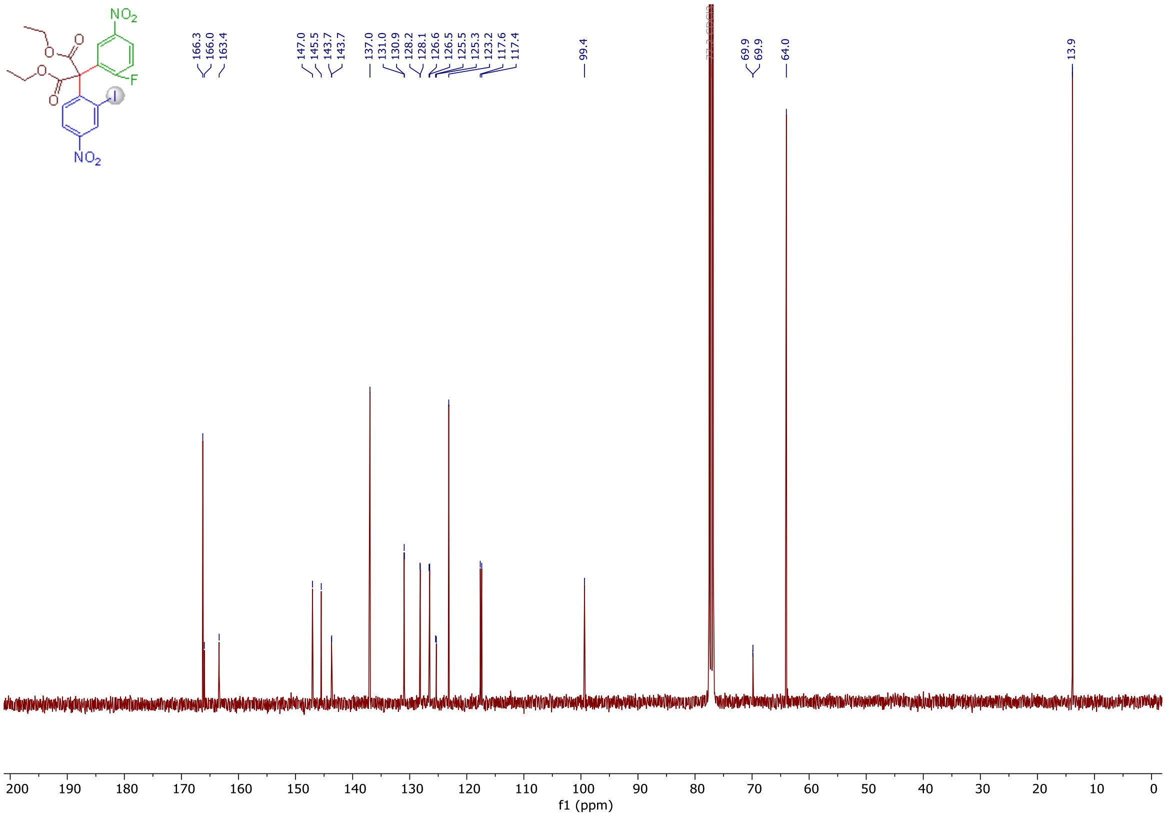

^{19}F NMR (101 MHz, CDCl$_3$), 3x:

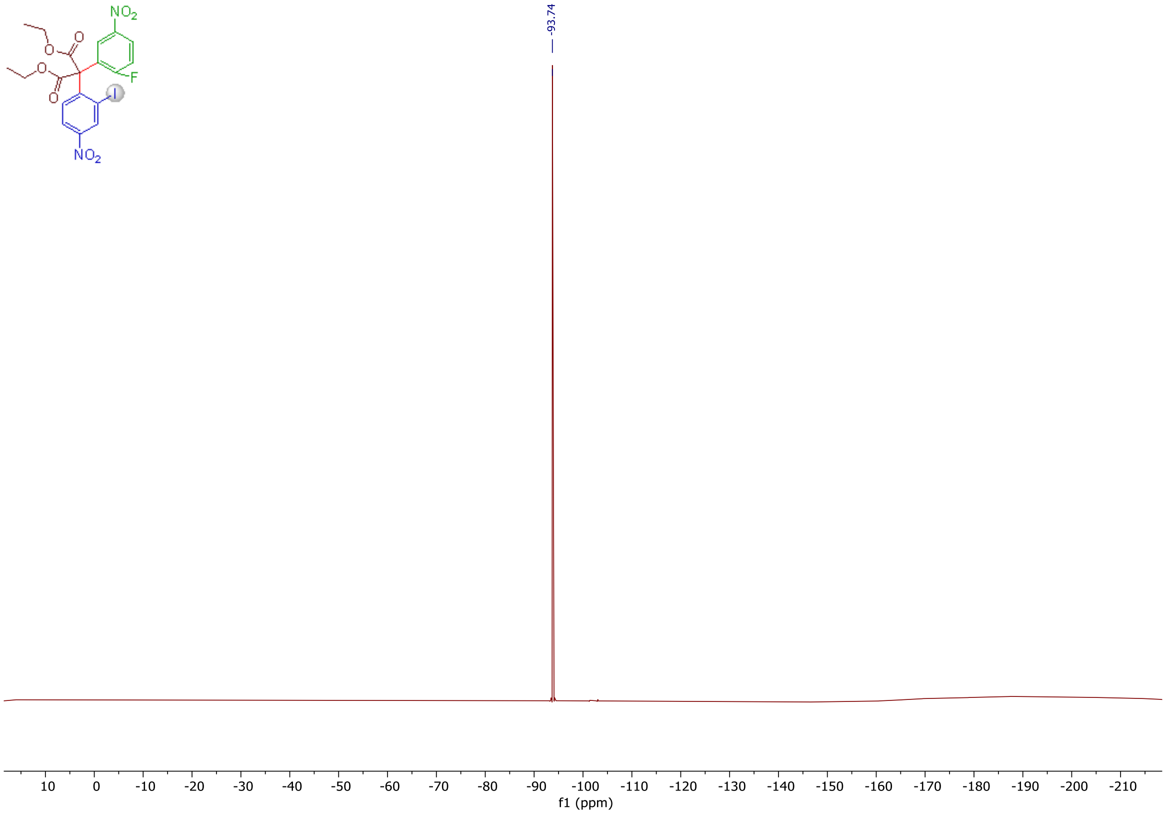

^{1}H NMR (400 MHz, CDCl$_3$), 3y:

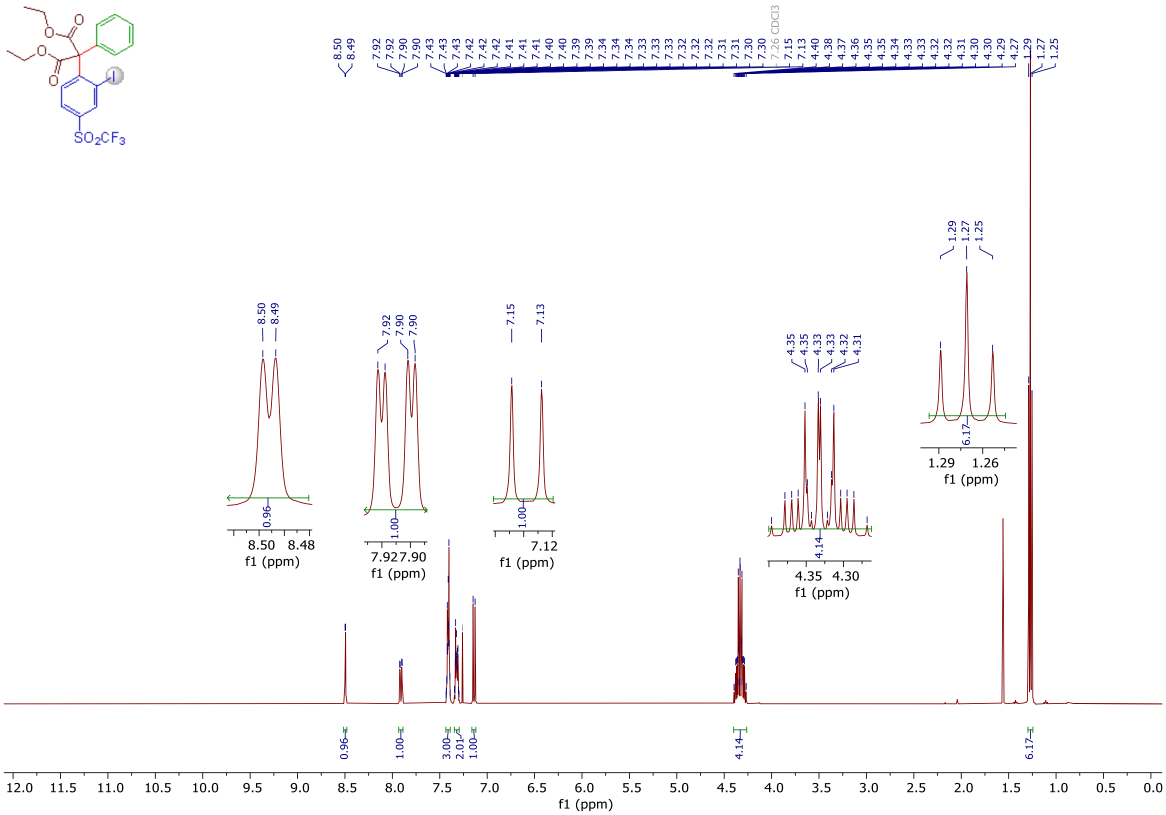

^{13}C NMR (101 MHz, $CDCl_3$), 3y:

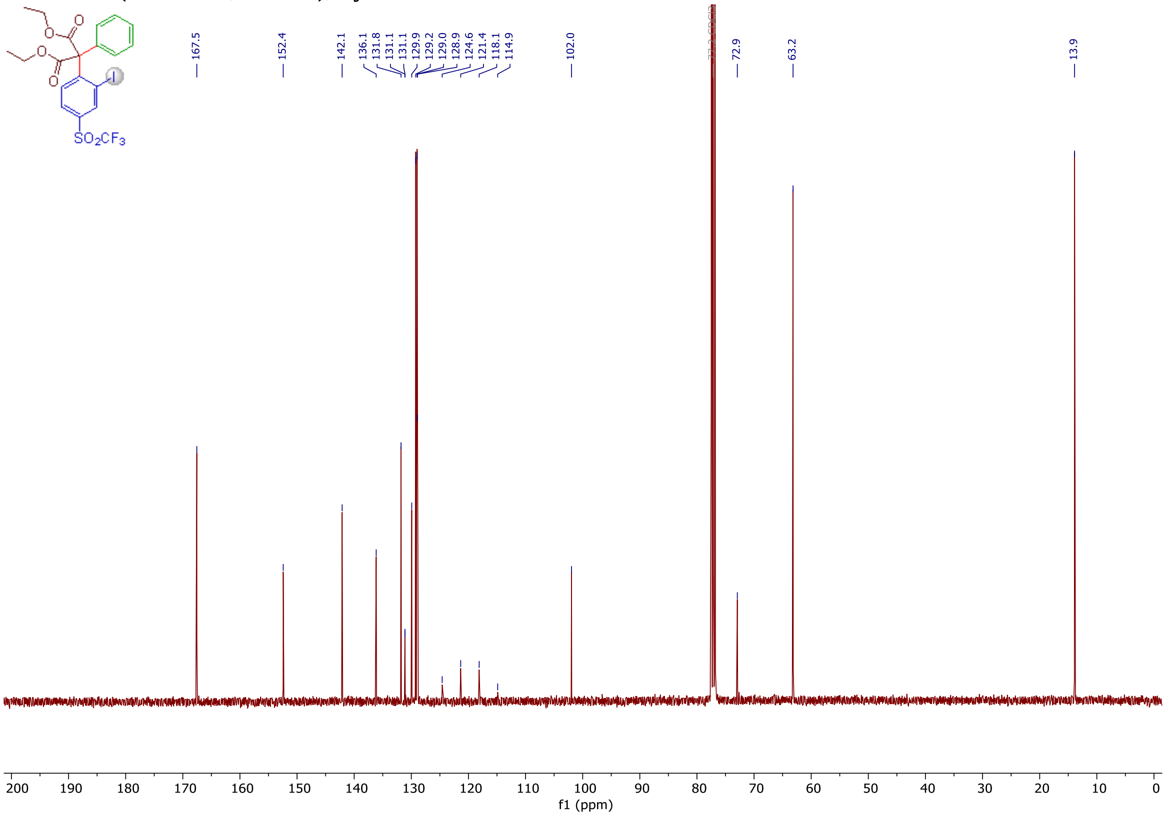

^{19}F NMR (377 MHz, $CDCl_3$), 3y:

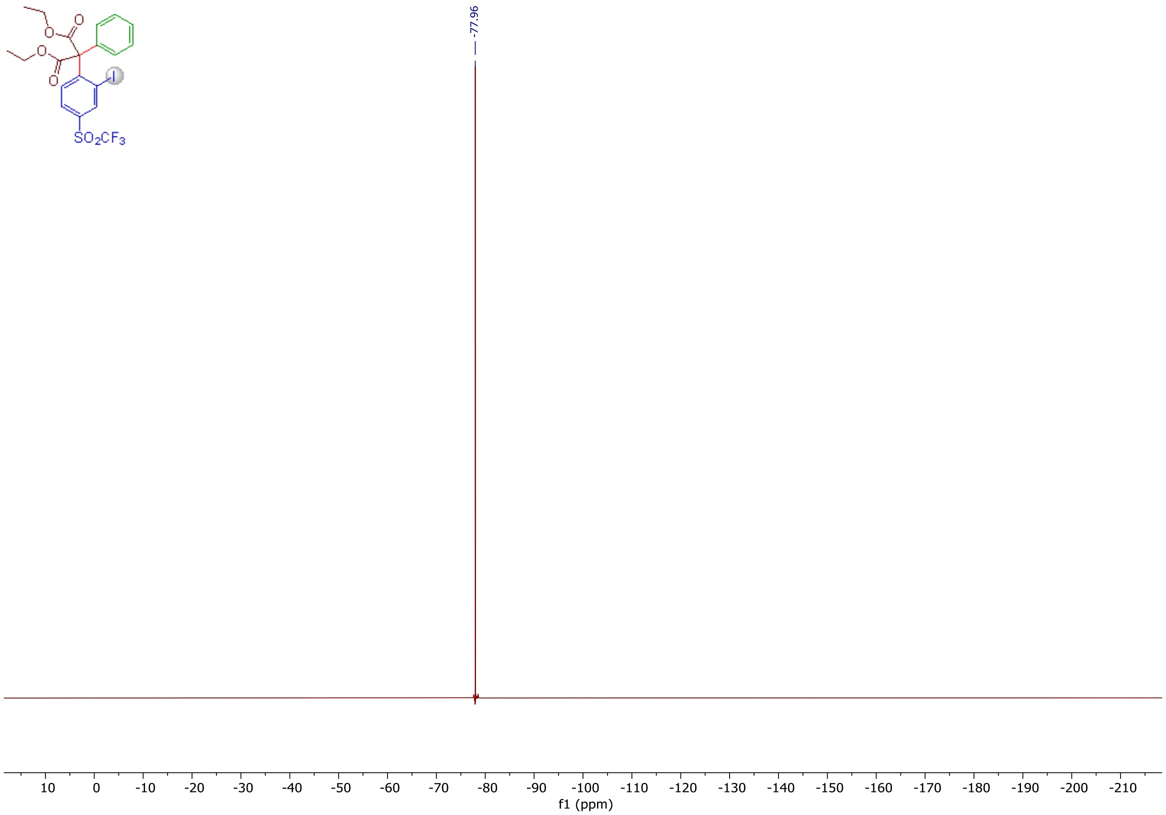

^{1}H NMR (400 MHz, $CDCl_3$), 3z:

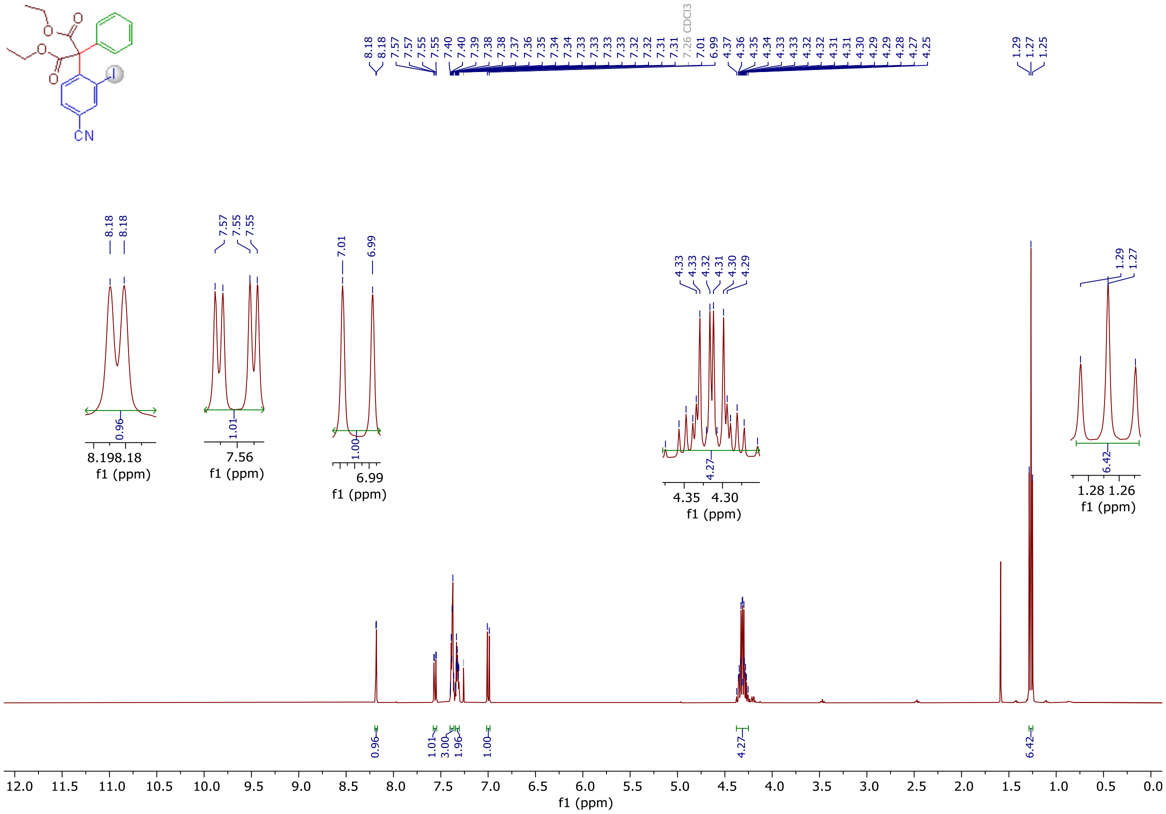

^{13}C NMR (101 MHz, $CDCl_3$), 3z:

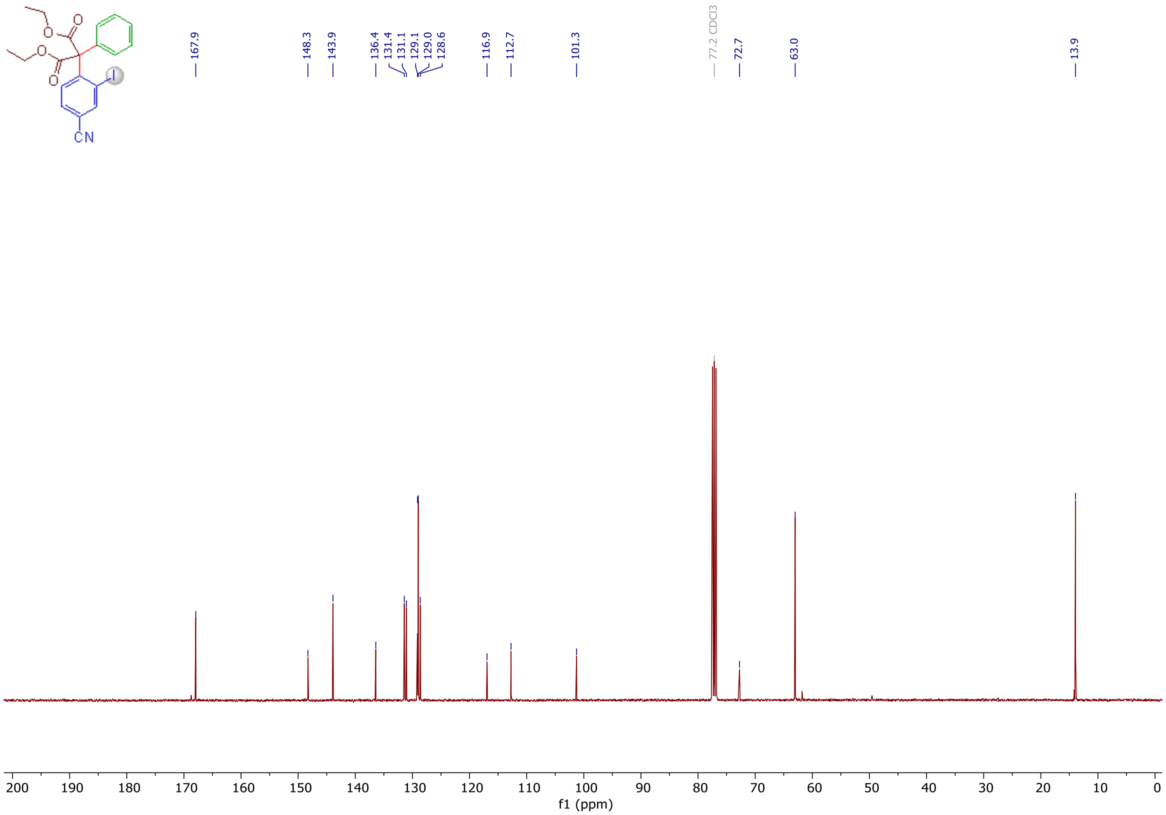

^{1}H NMR (400 MHz, $CDCl_3$), 3aa:

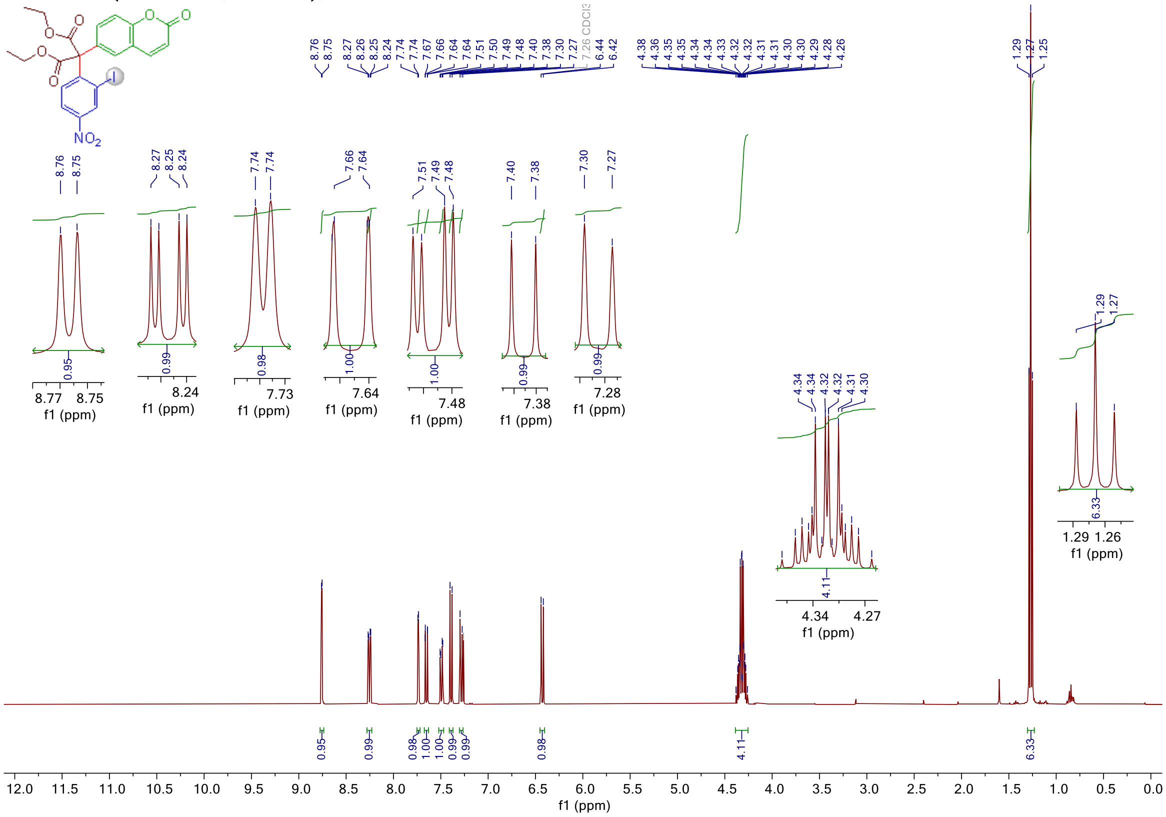

^{13}C NMR (101 MHz, $CDCl_3$), 3aa:

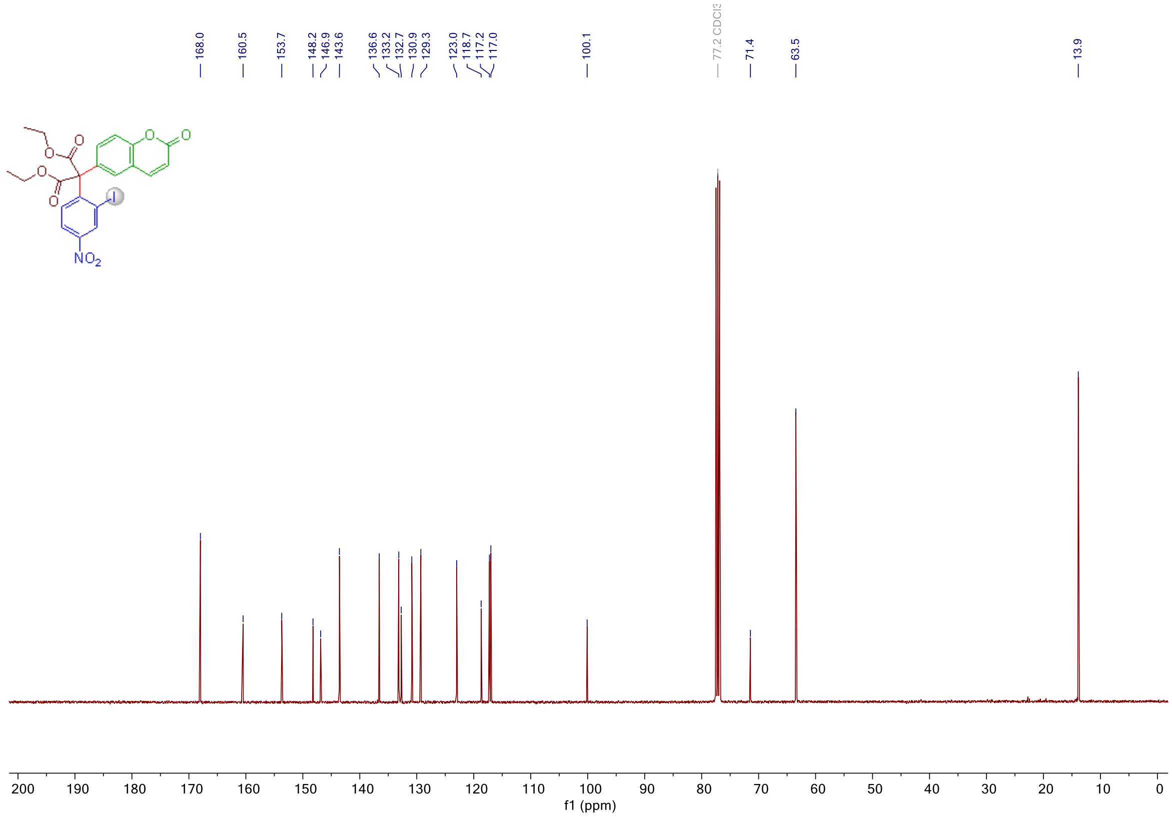

^{1}H NMR (400 MHz, CDCl$_3$), 3ab:

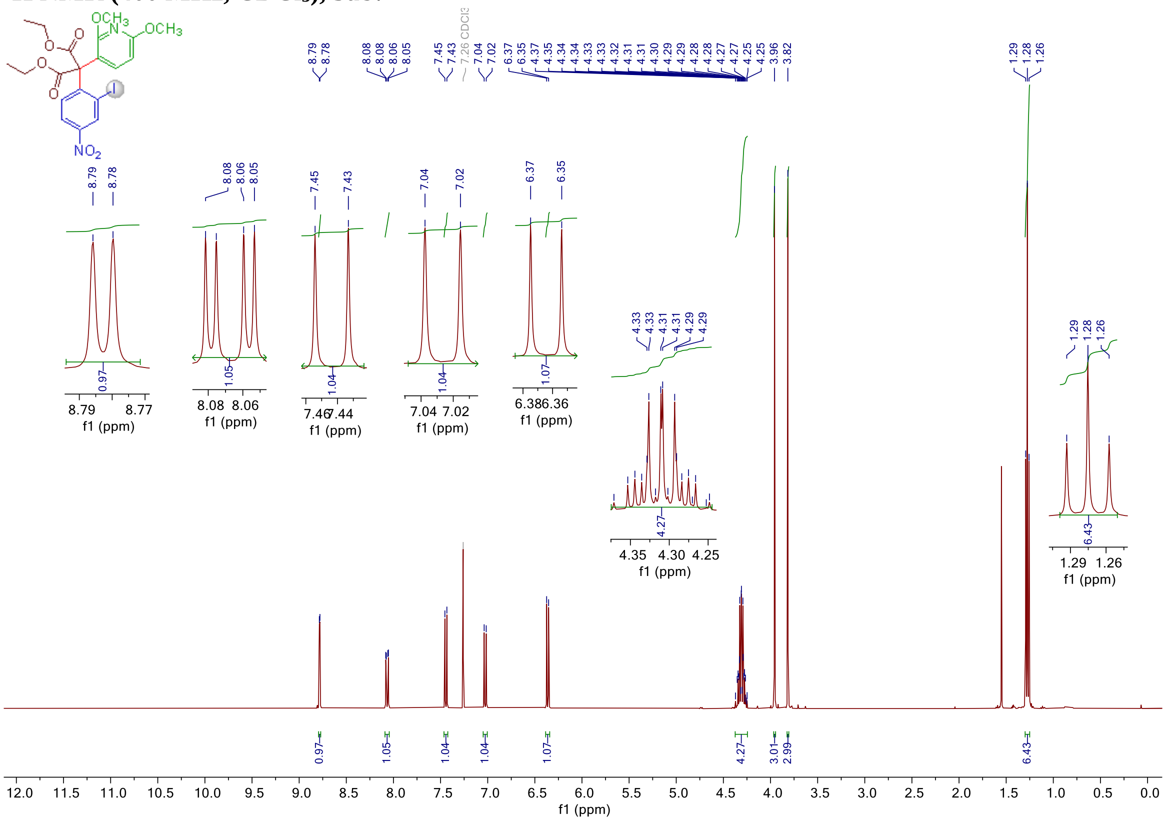

^{13}C NMR (101 MHz, CDCl$_3$), 3ab:

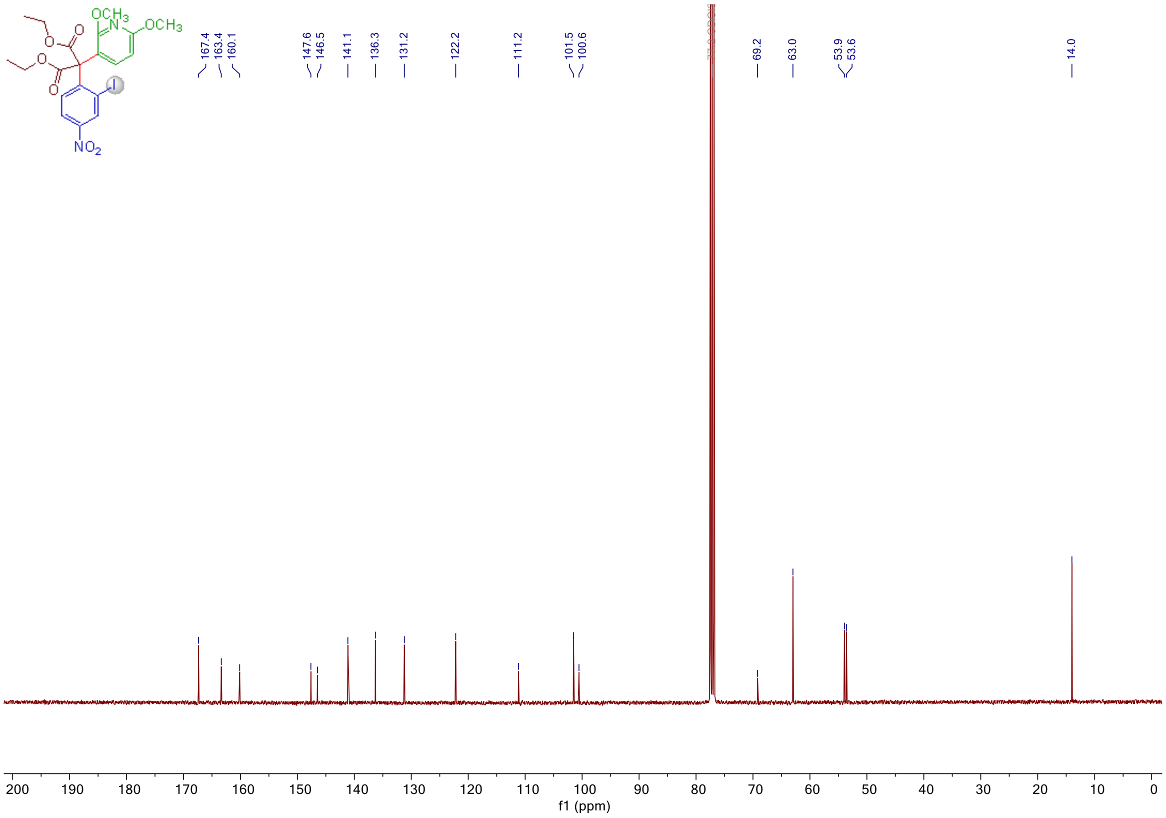

^{1}H NMR (400 MHz, CDCl$_3$), 3ac:

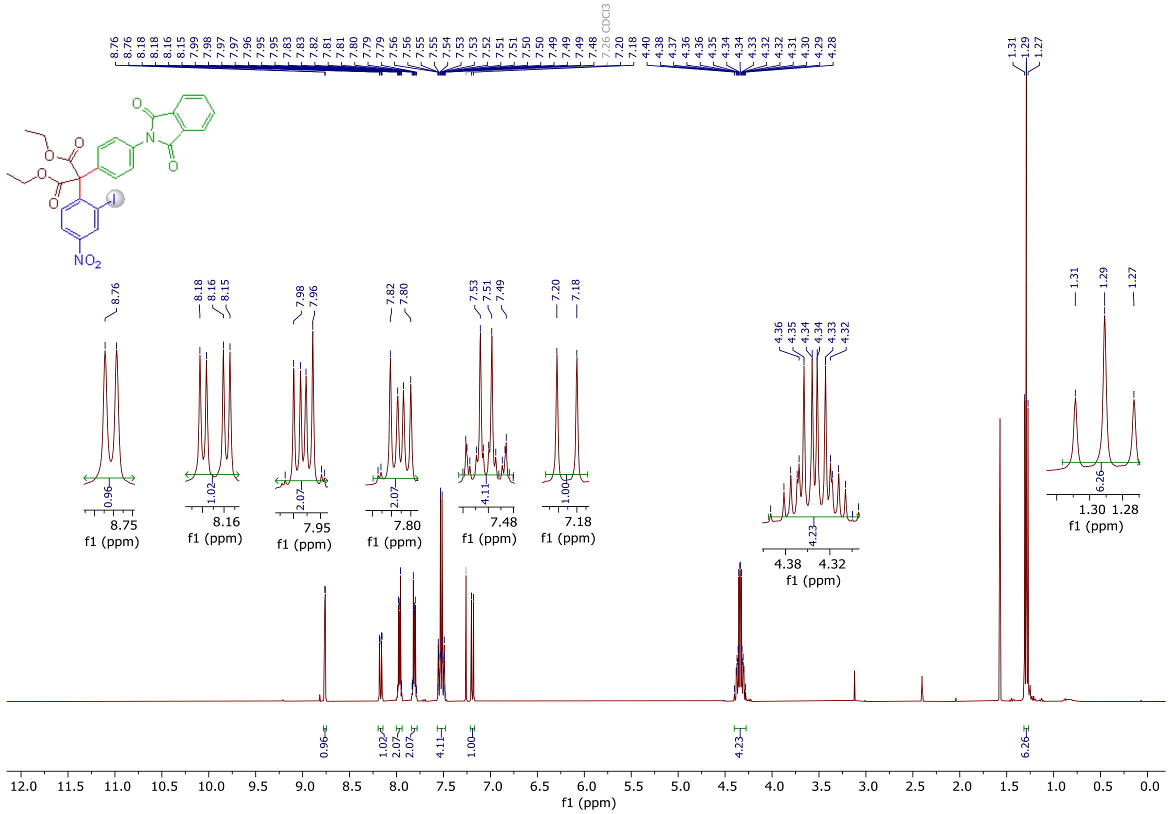

^{13}C NMR (101 MHz, CDCl$_3$), 3ac:

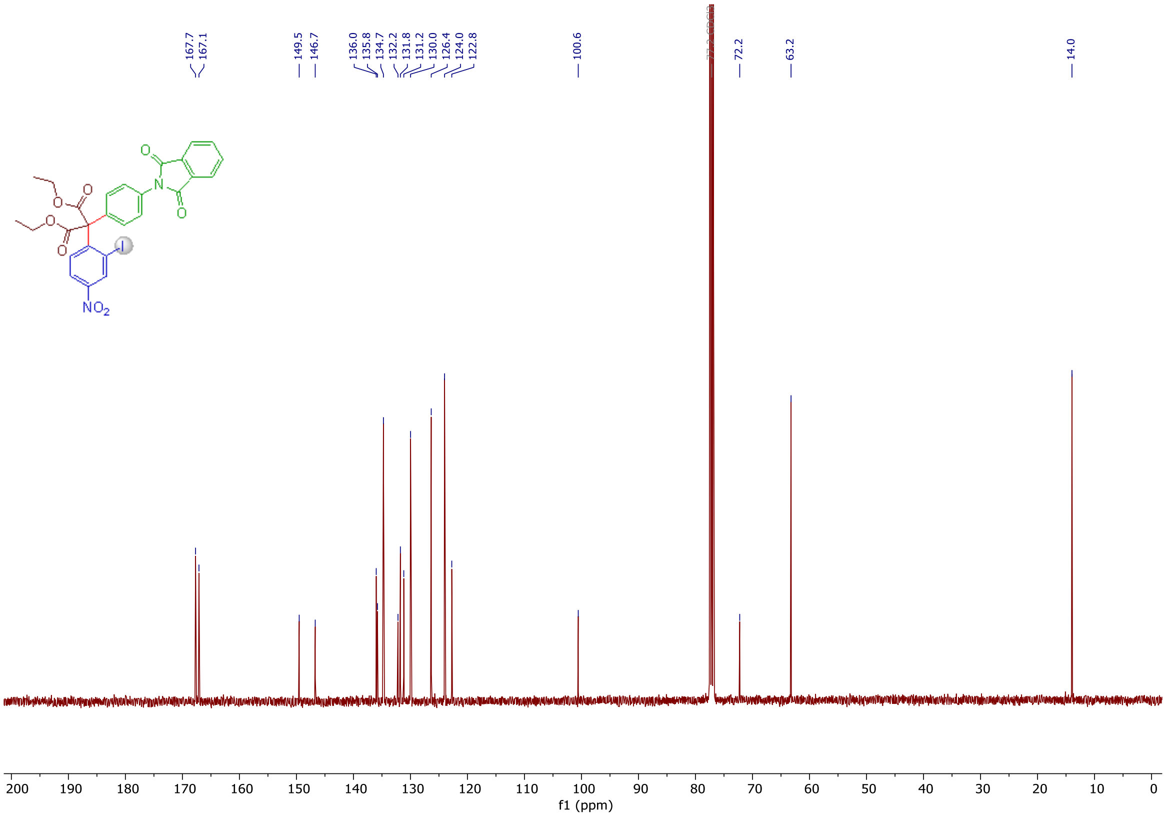

^{1}H NMR (400 MHz, CDCl₃), 3ad:

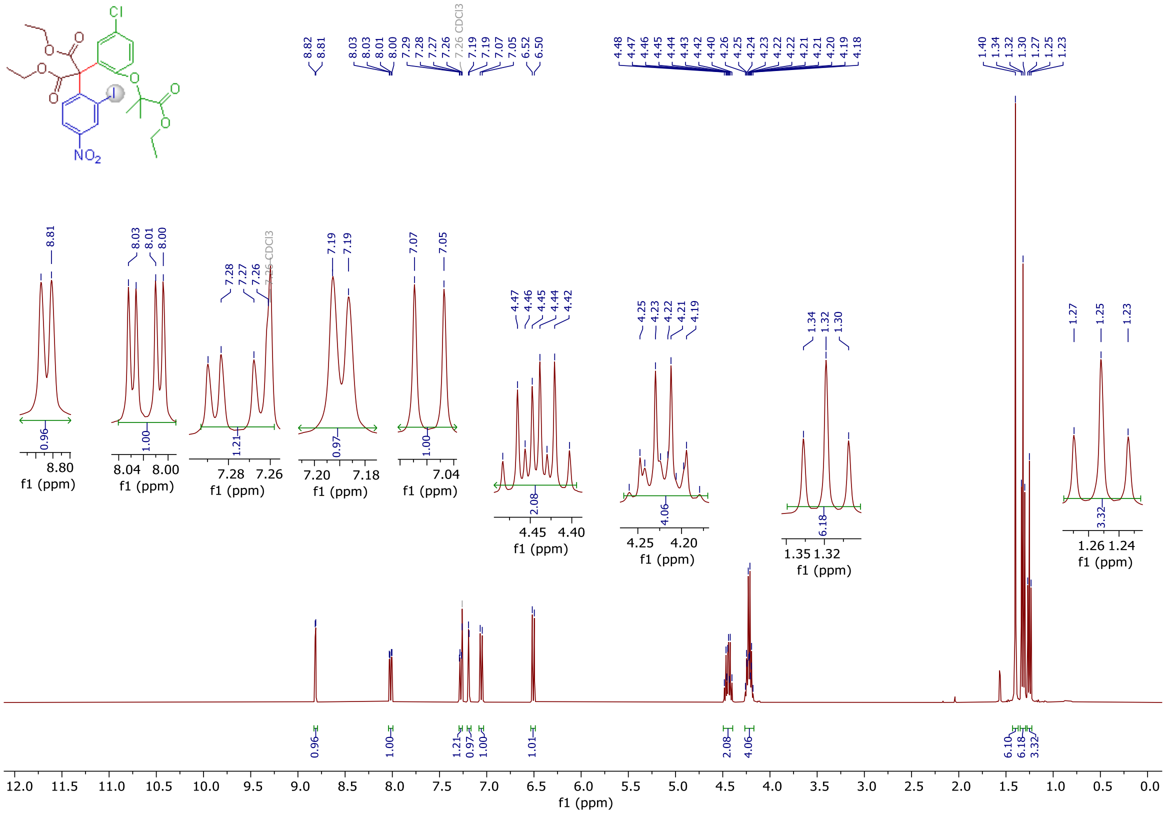

^{13}C NMR (101 MHz, CDCl₃), 3ad:

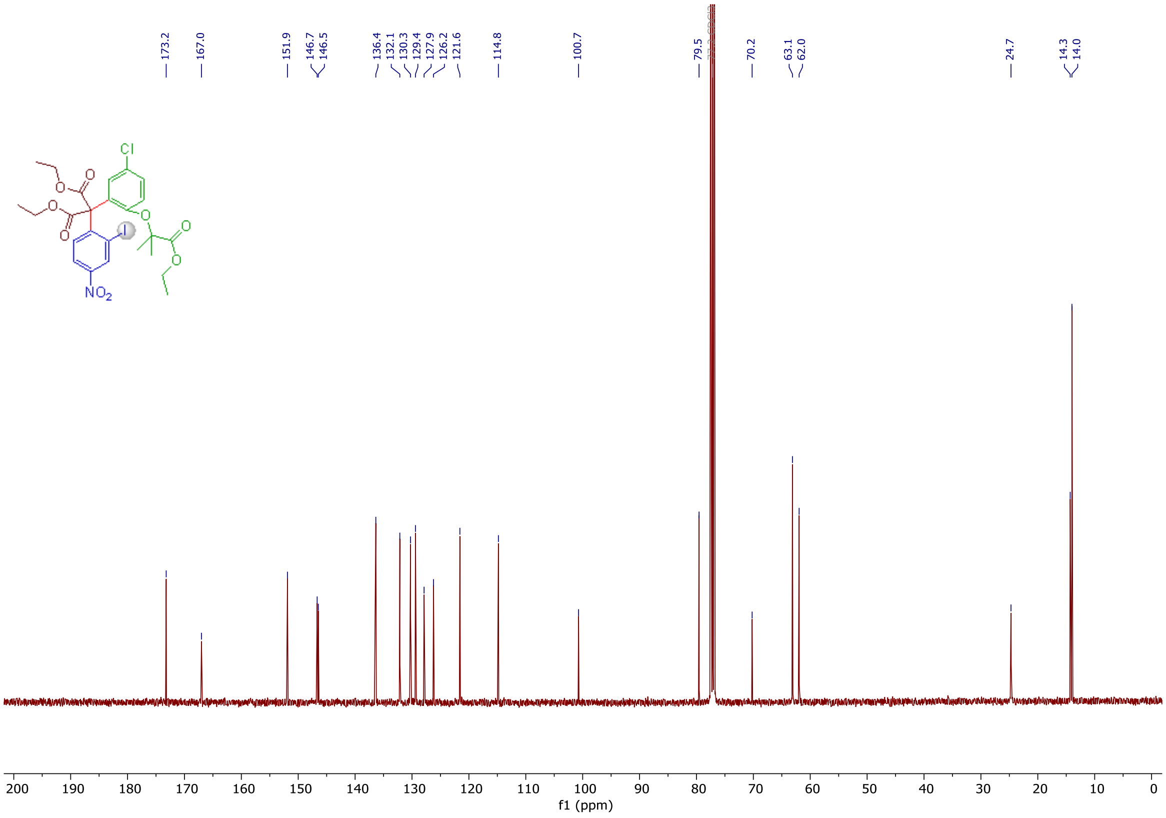

^{1}H NMR (400 MHz, $CDCl_3$), 3ae:

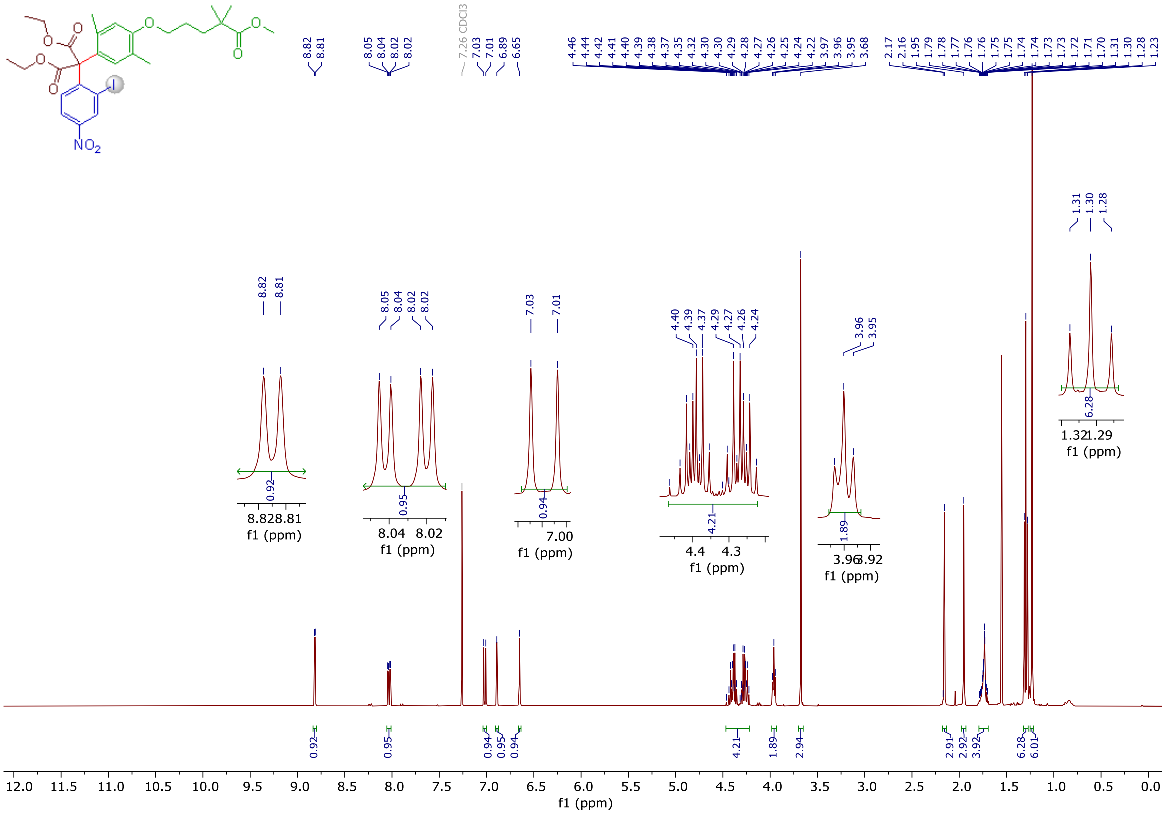

^{13}C NMR (101 MHz, $CDCl_3$), 3ae:

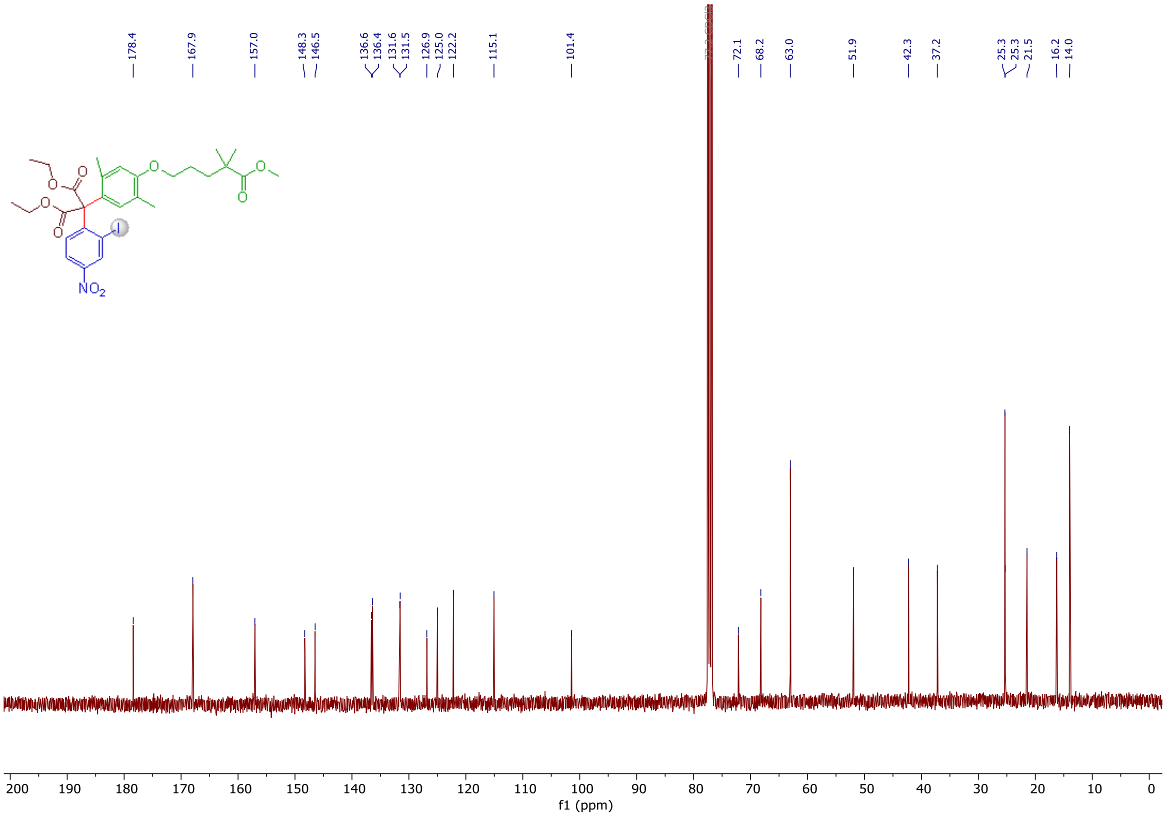

^{1}H NMR (400 MHz, $CDCl_3$), 4a:

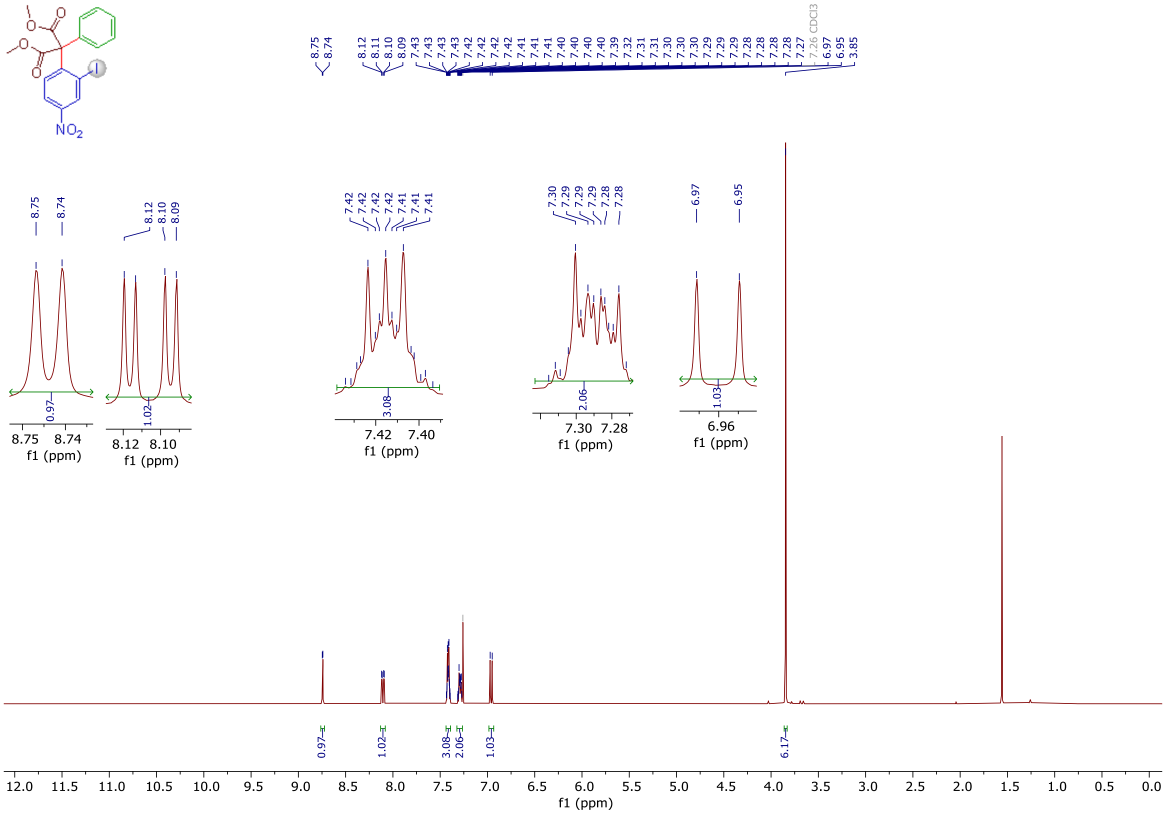

^{13}C NMR (101 MHz, $CDCl_3$), 4a:

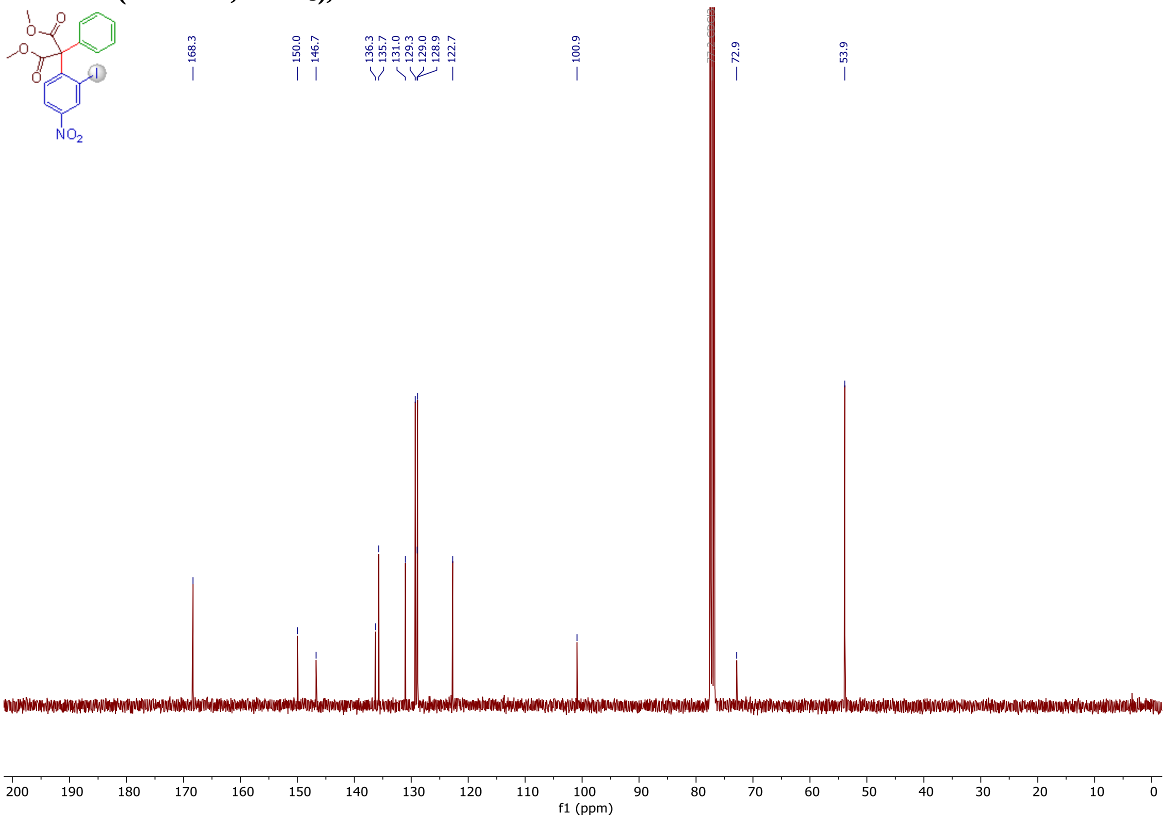

^{1}H NMR (400 MHz, $CDCl_3$), 4b:

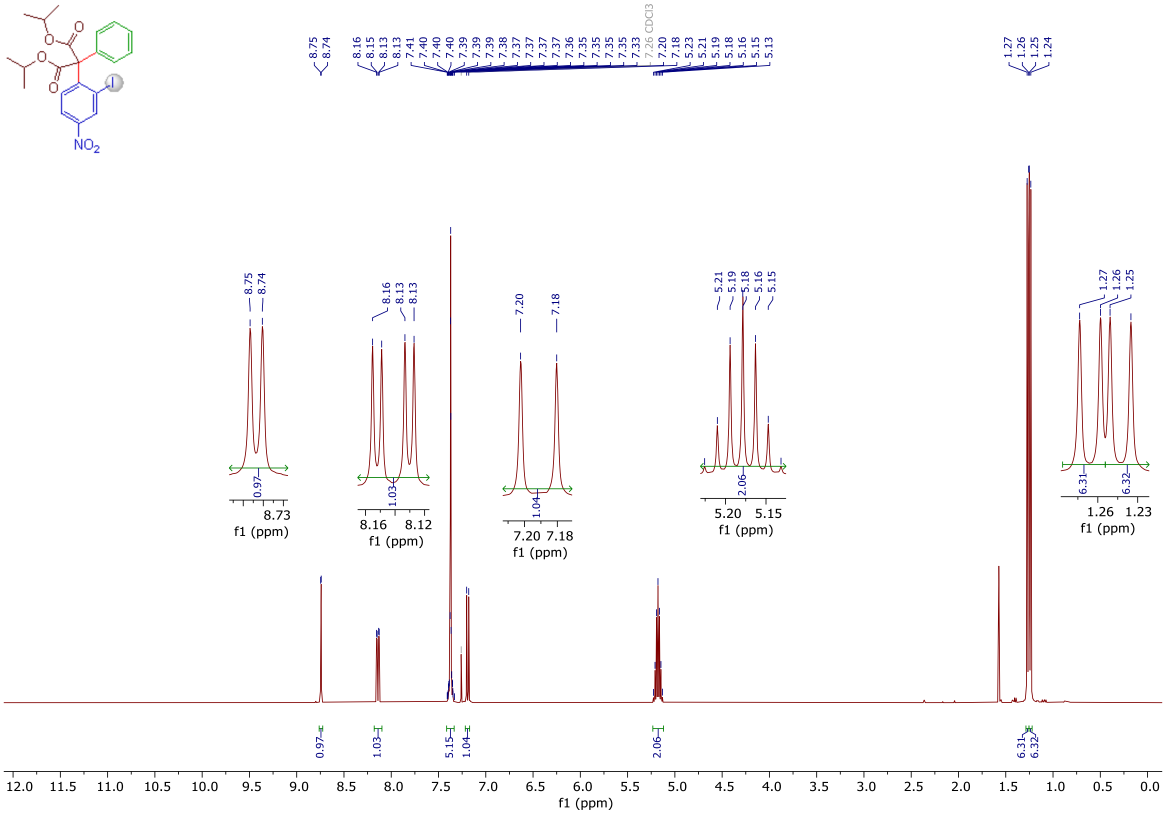

^{13}C NMR (101 MHz, $CDCl_3$), 4b:

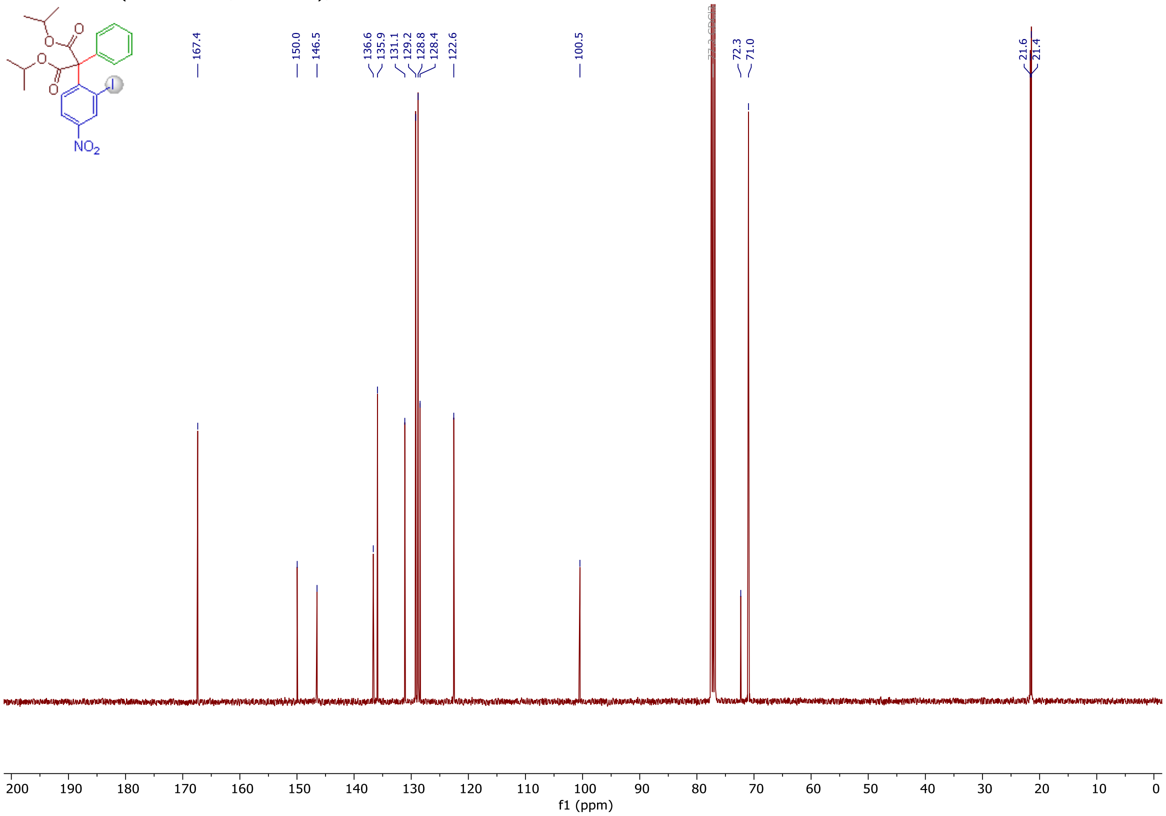

^{1}H NMR (400 MHz, $CDCl_3$), 4c:

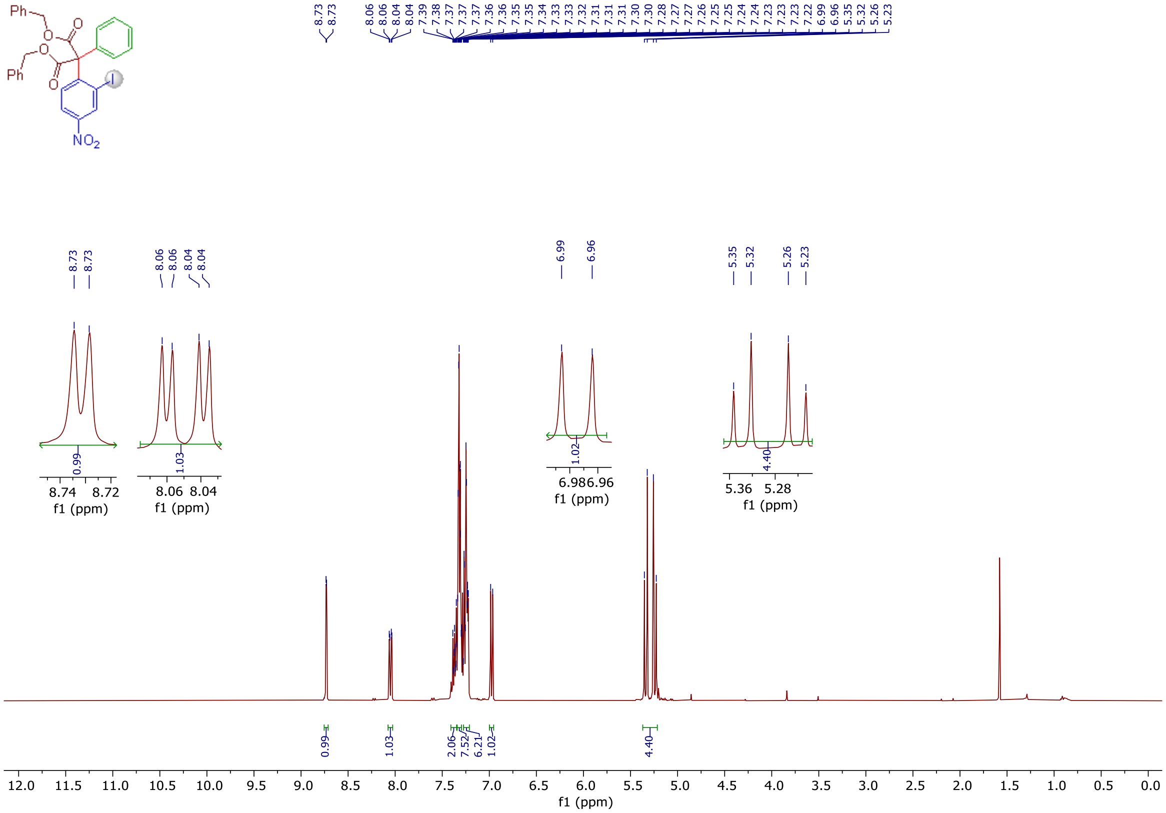

^{13}C NMR (101 MHz, CDCl3), 4c:

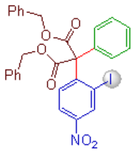

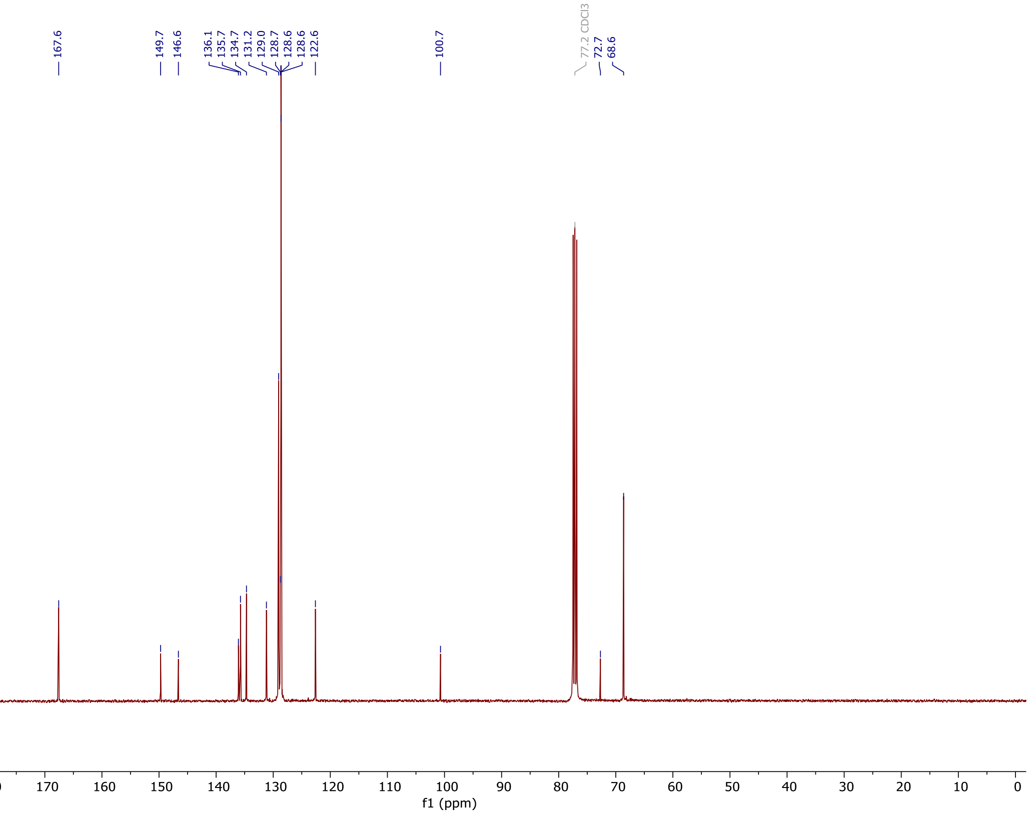

^{1}H NMR (400 MHz, CDCl3), 4d:

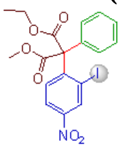

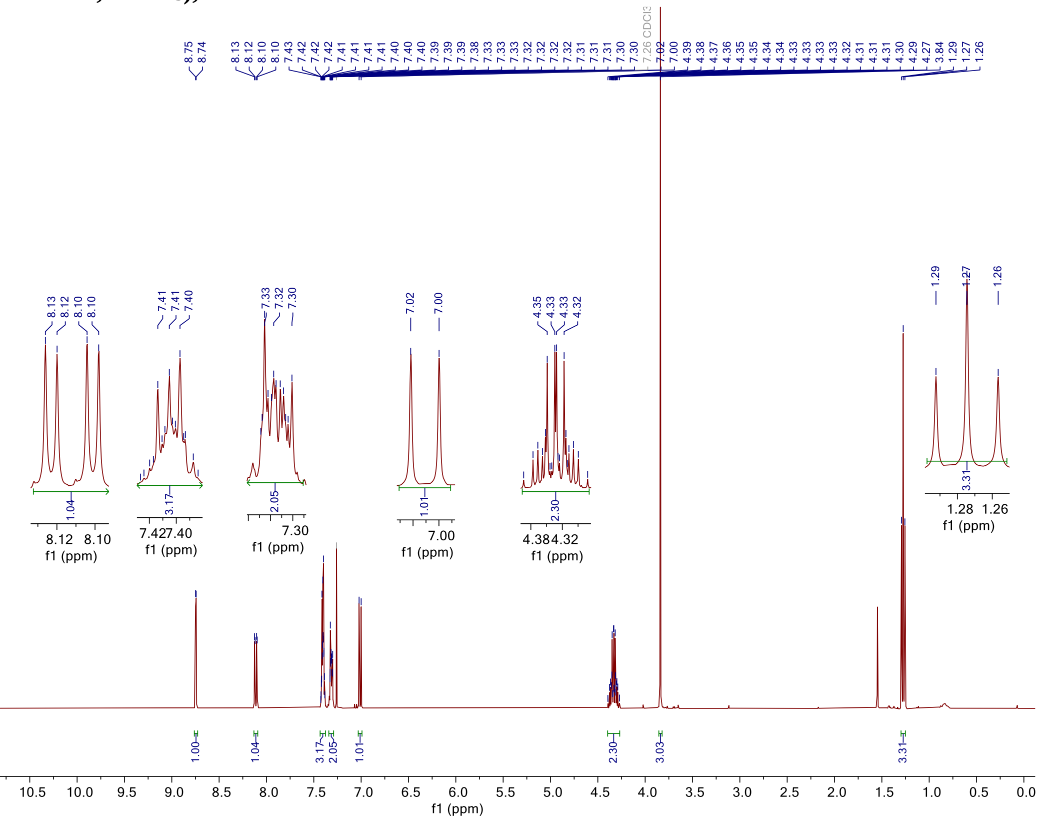

^{13}C NMR (101 MHz, CDCl$_3$), 4d:

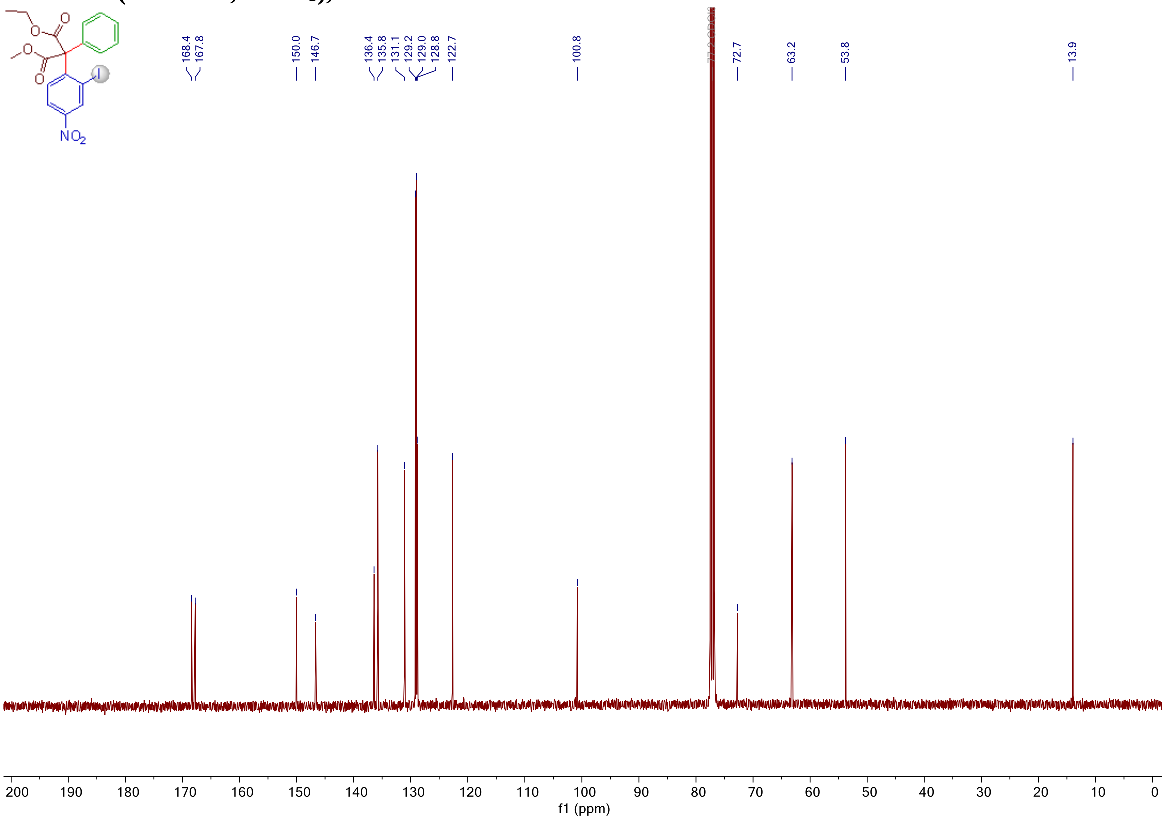

^{1}H NMR (400 MHz, CDCl$_3$), 4e:

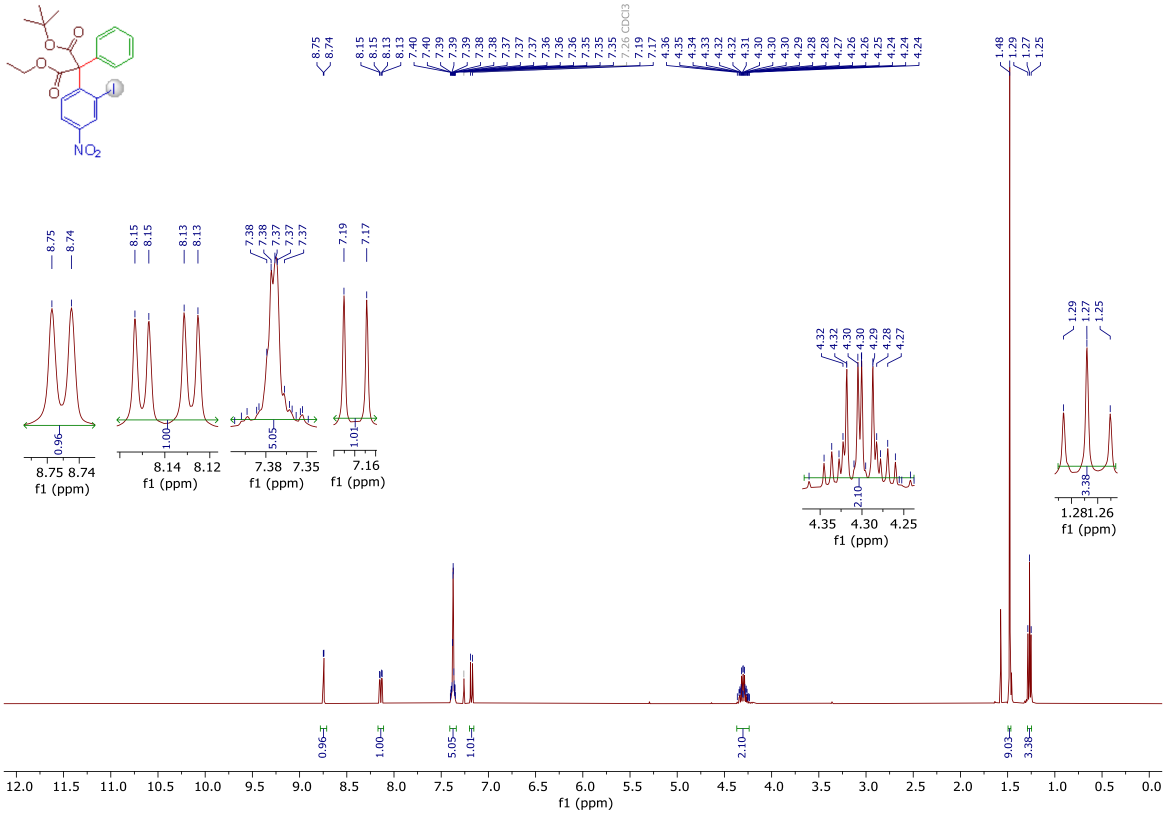

^{13}C NMR (101 MHz, $CDCl_3$), 4e:

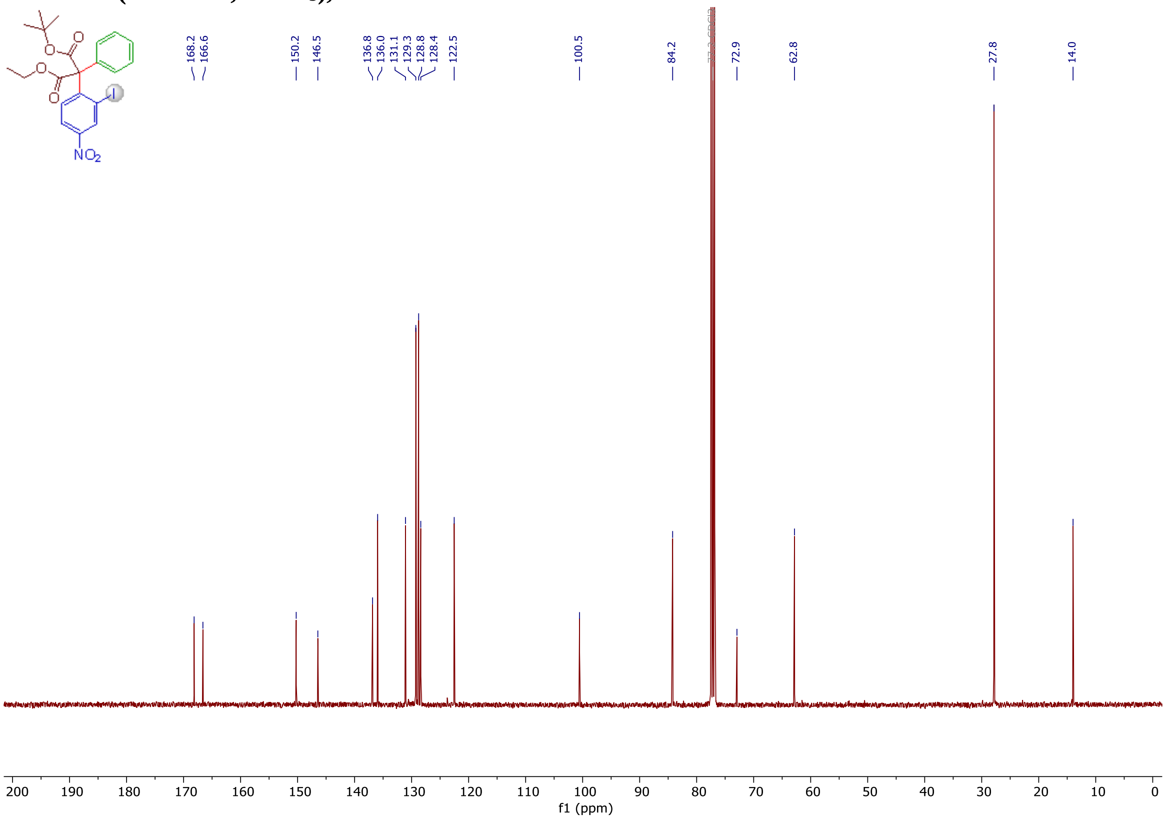

^{1}H NMR (400 MHz, $CDCl_3$), 4f:

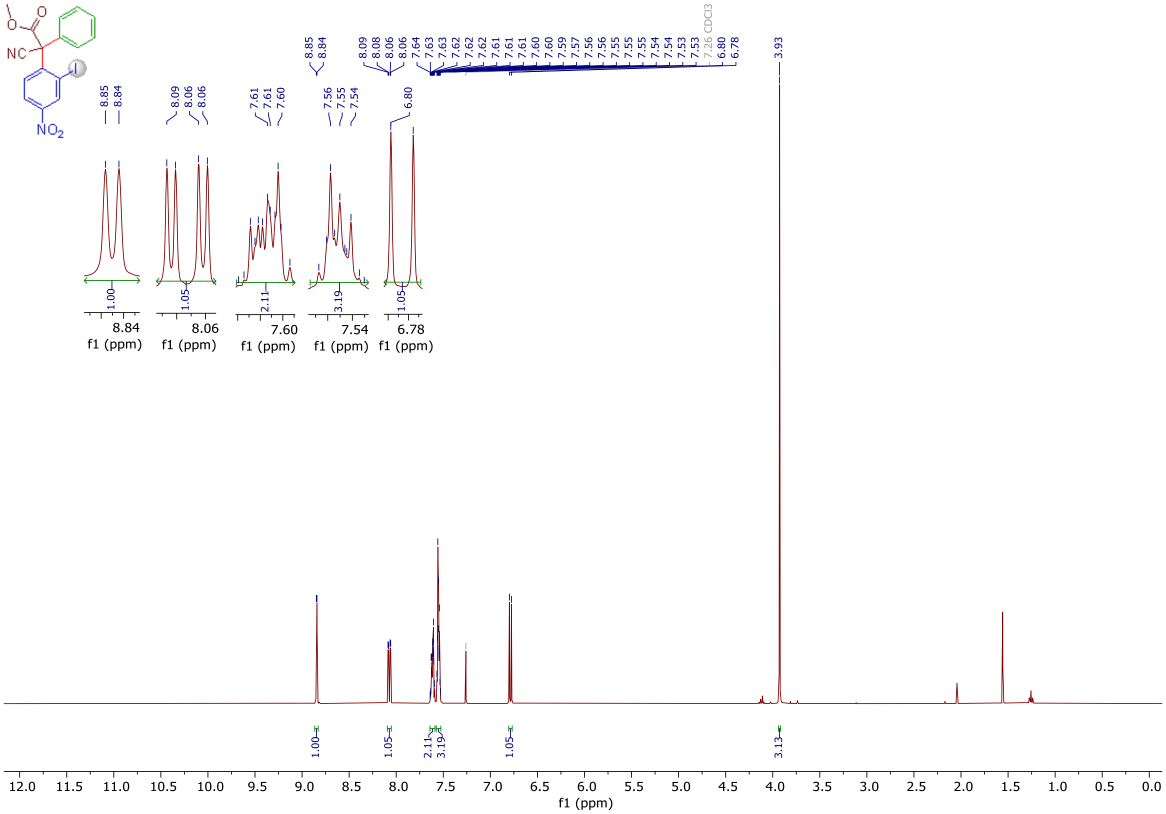

^{13}C NMR (101 MHz, CDCl$_3$), 4f:

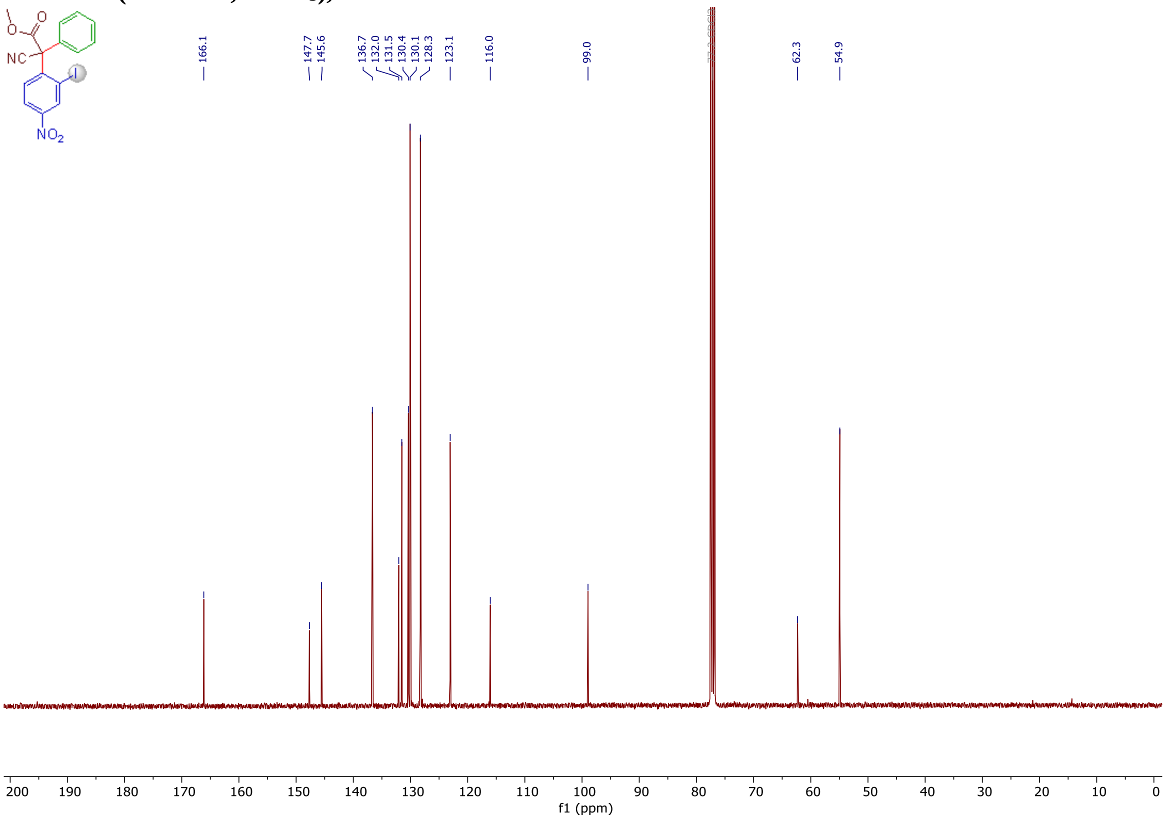

^{1}H NMR (400 MHz, CDCl$_3$), 4g:

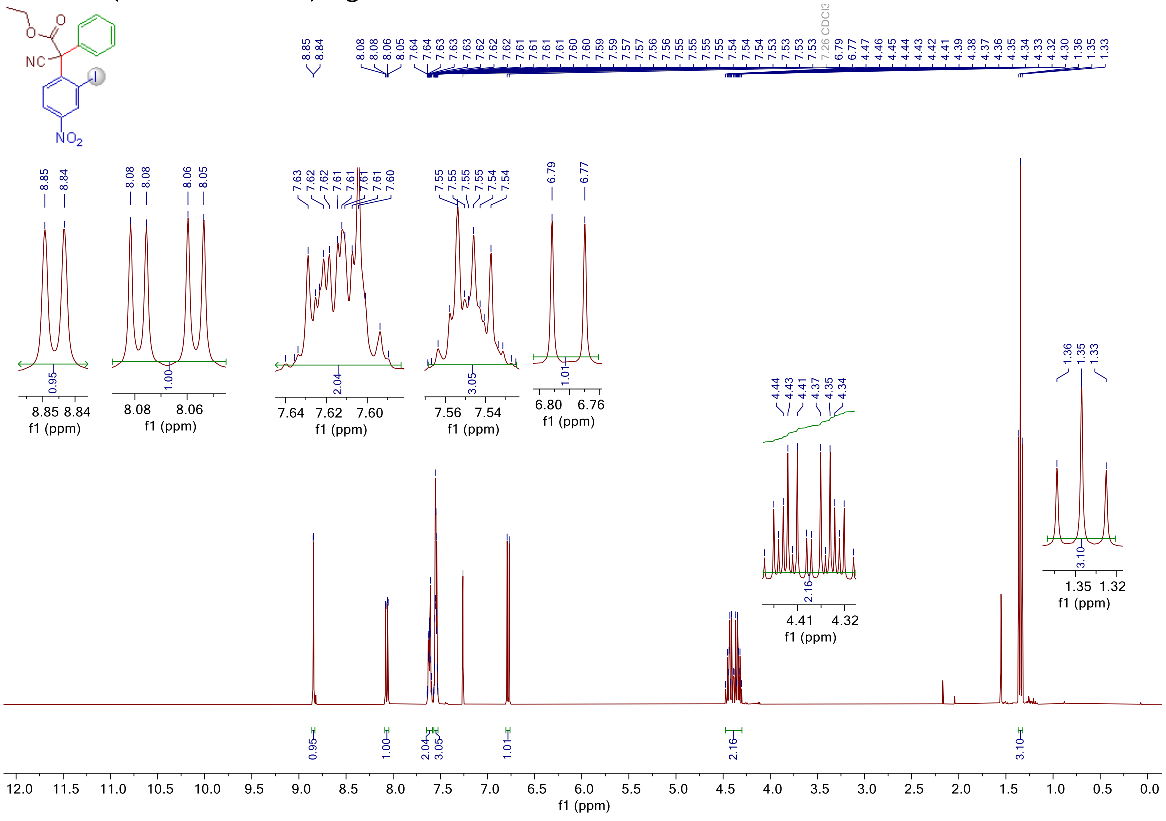

^{13}C NMR (101 MHz, $CDCl_3$), 4g:

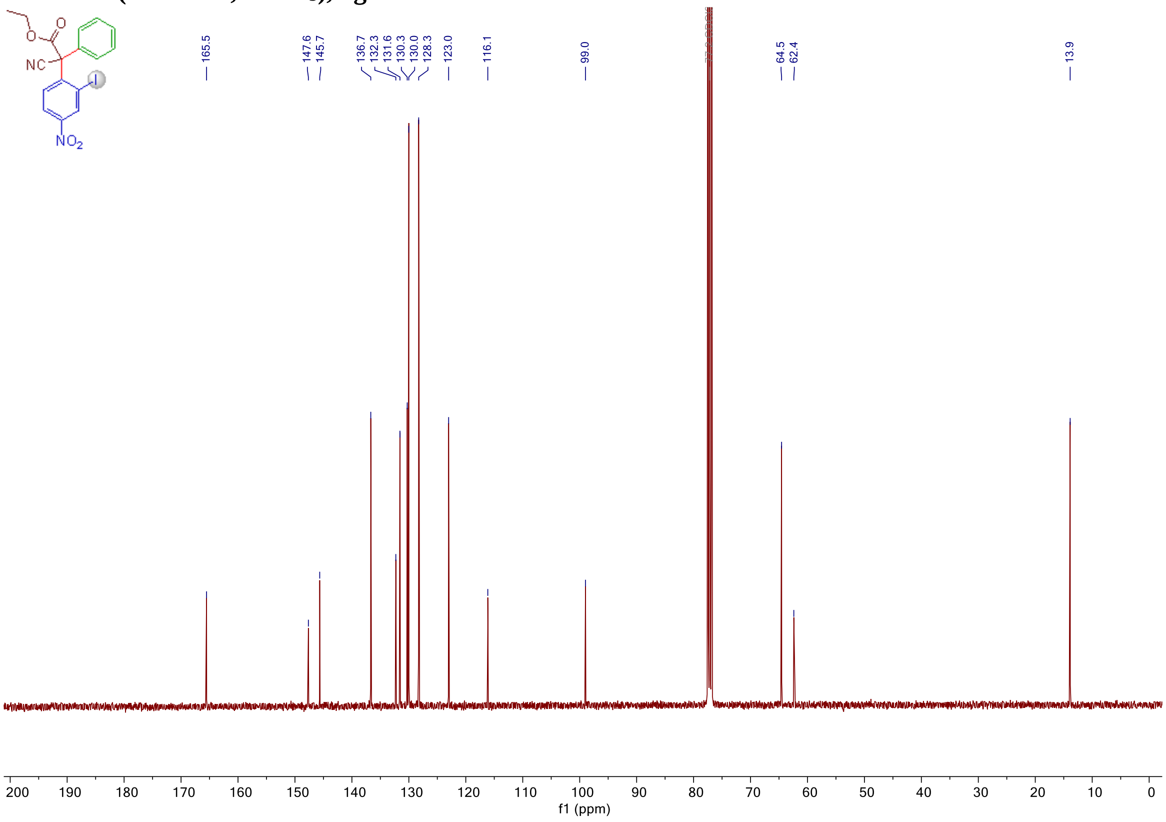

^{1}H NMR (400 MHz, $CDCl_3$), 4h:

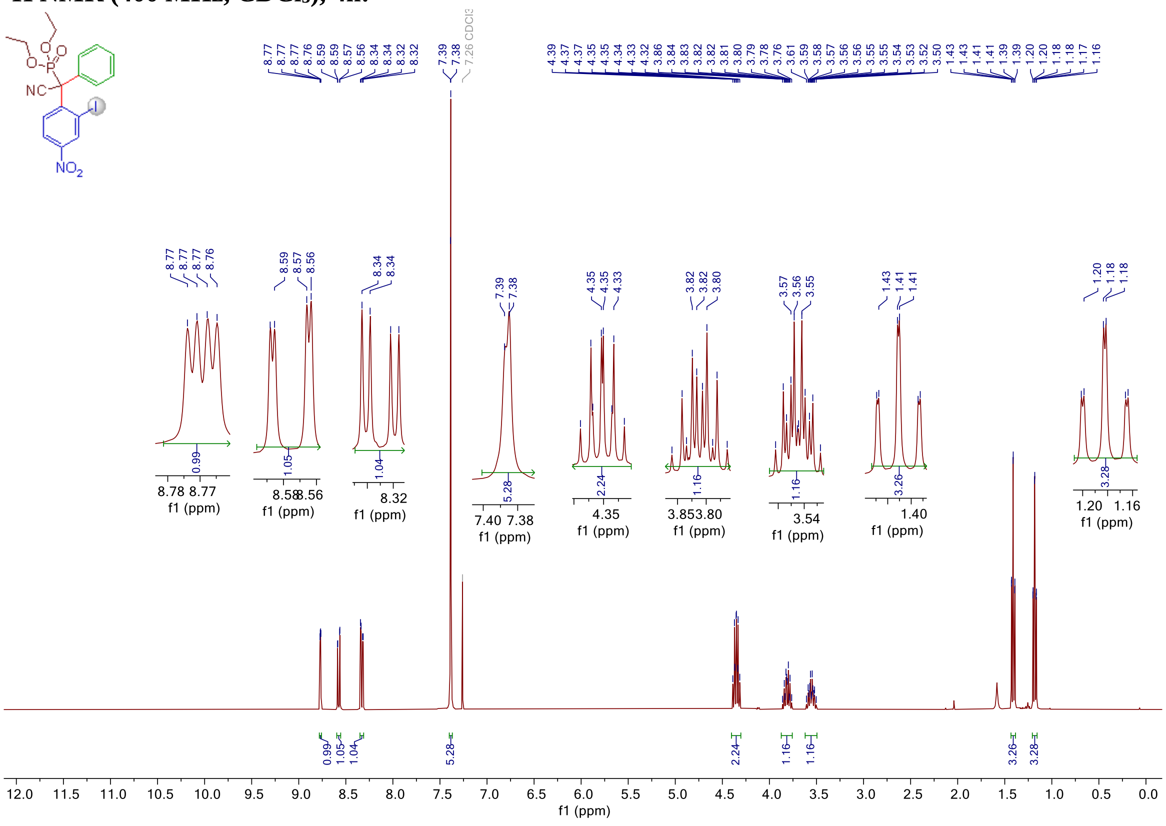

^{13}C NMR (101 MHz, $CDCl_3$), 4h:

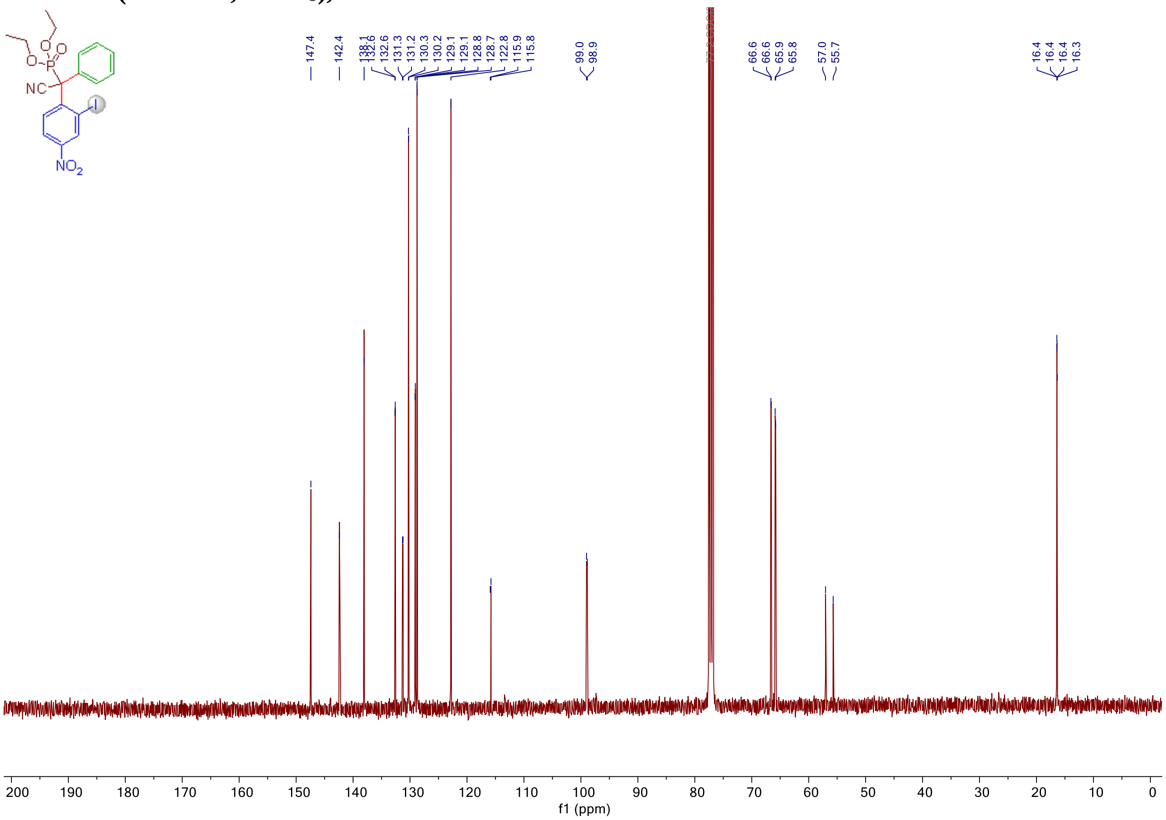

^{31}P NMR (162 MHz, $CDCl_3$), 4h:

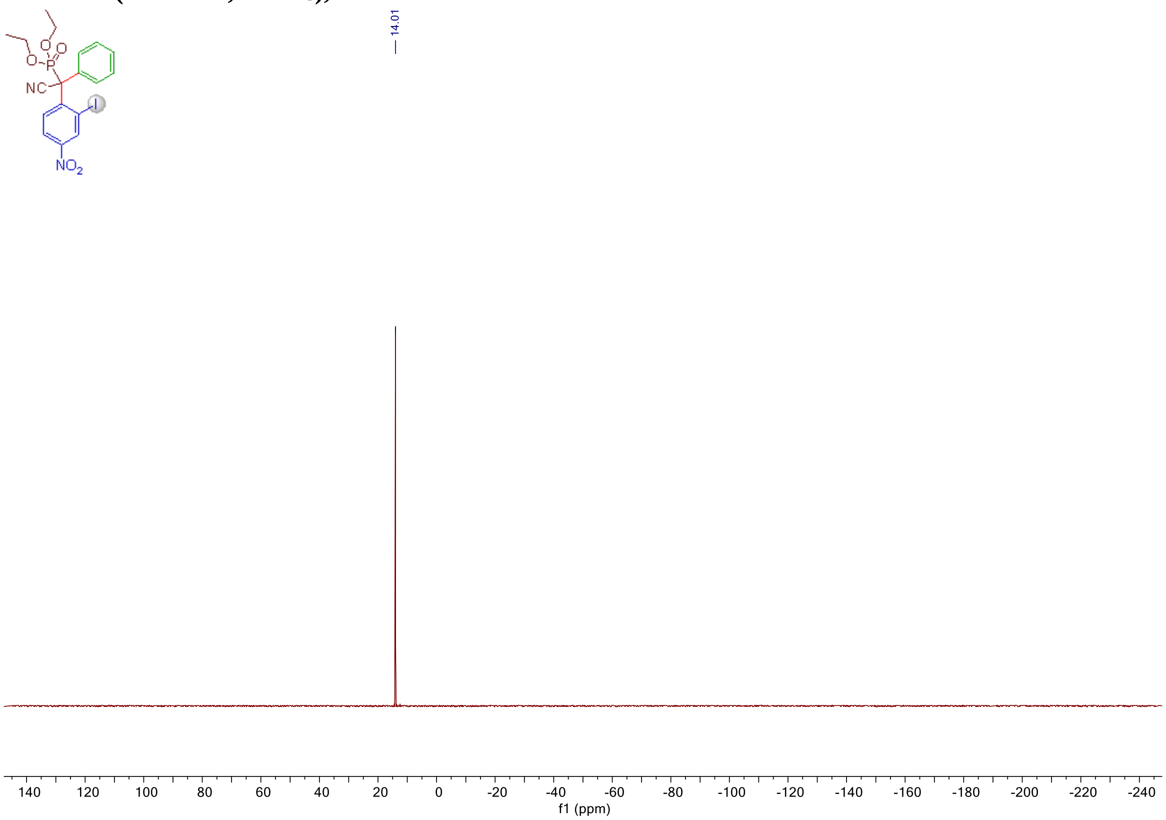

^{1}H NMR (400 MHz, $CDCl_3$), 4i:

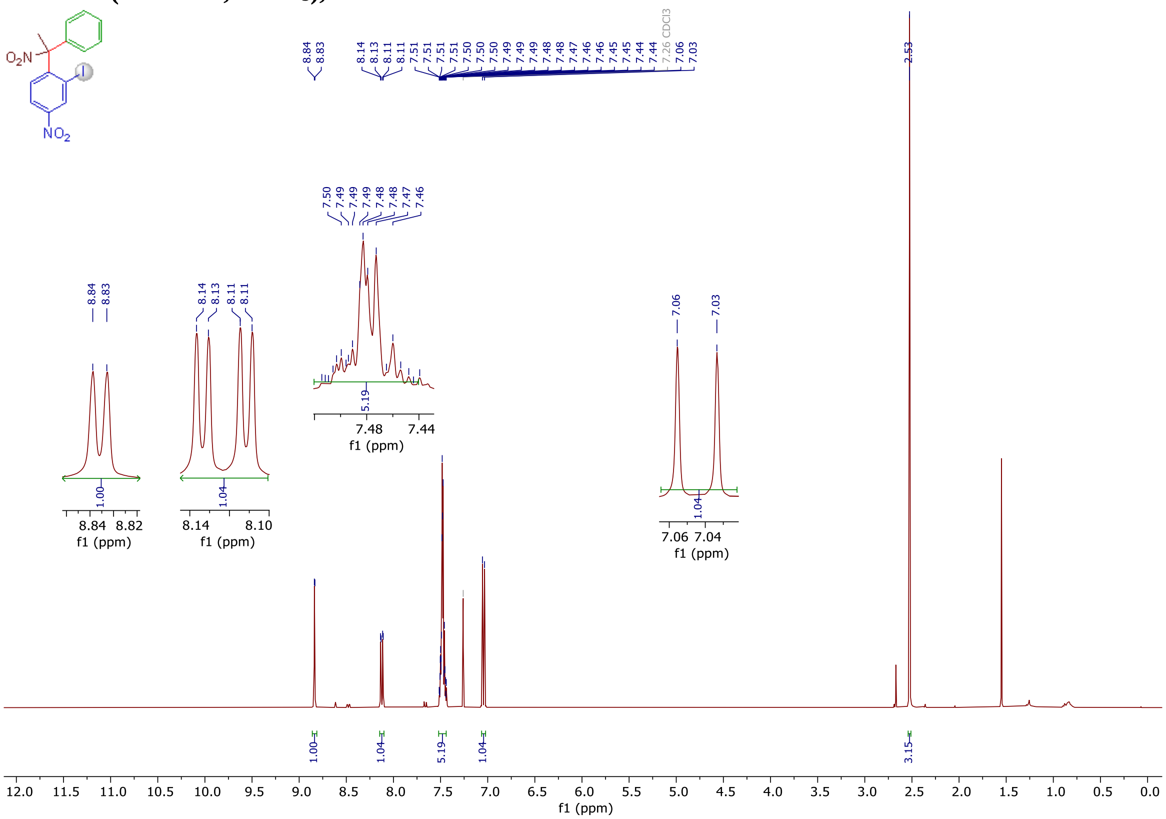

^{13}C NMR (101 MHz, $CDCl_3$), 4i:

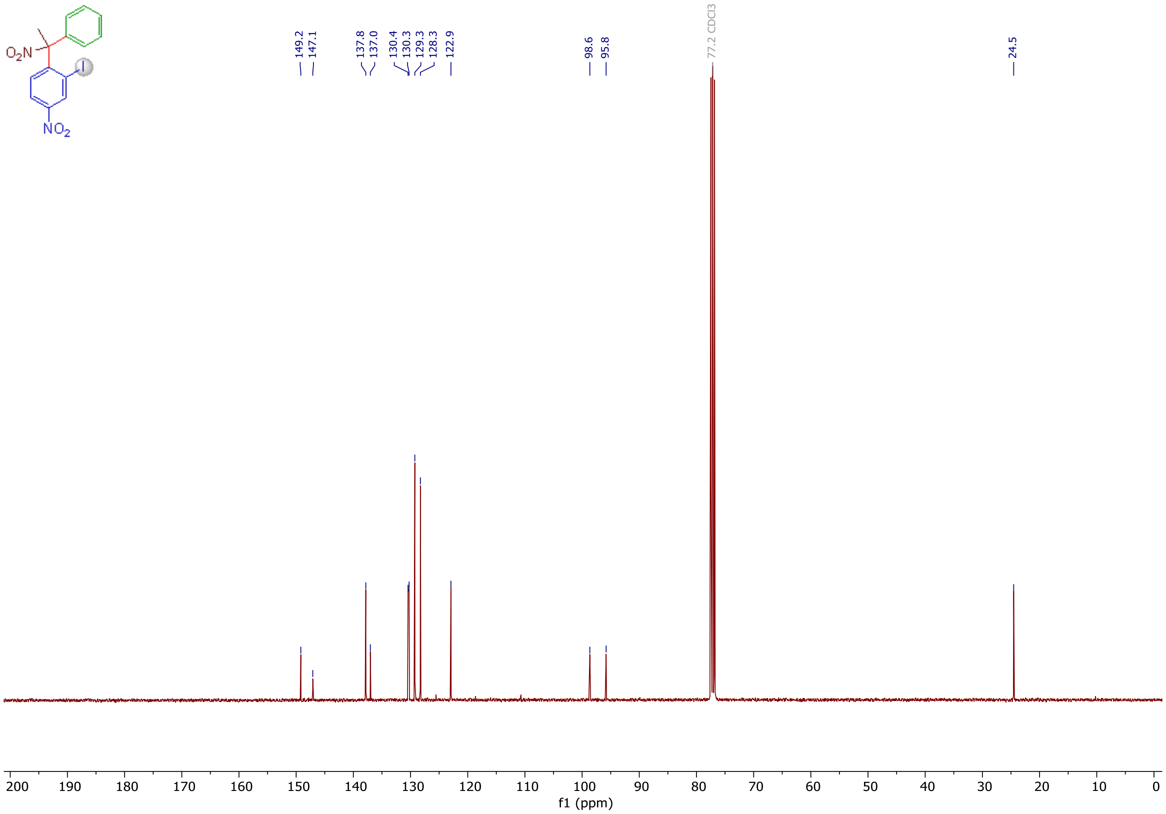

^{1}H NMR (400 MHz, CDCl₃), 4j:

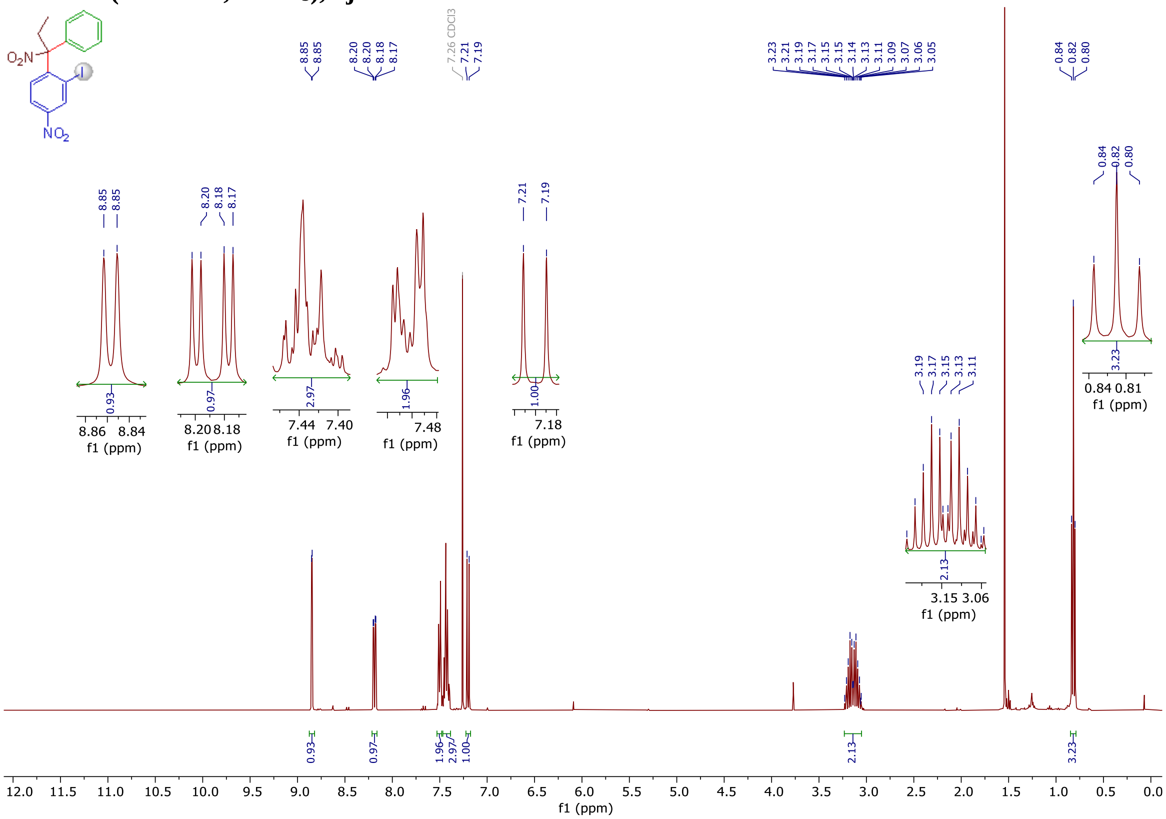

^{13}C NMR (101 MHz, CDCl₃), 4j:

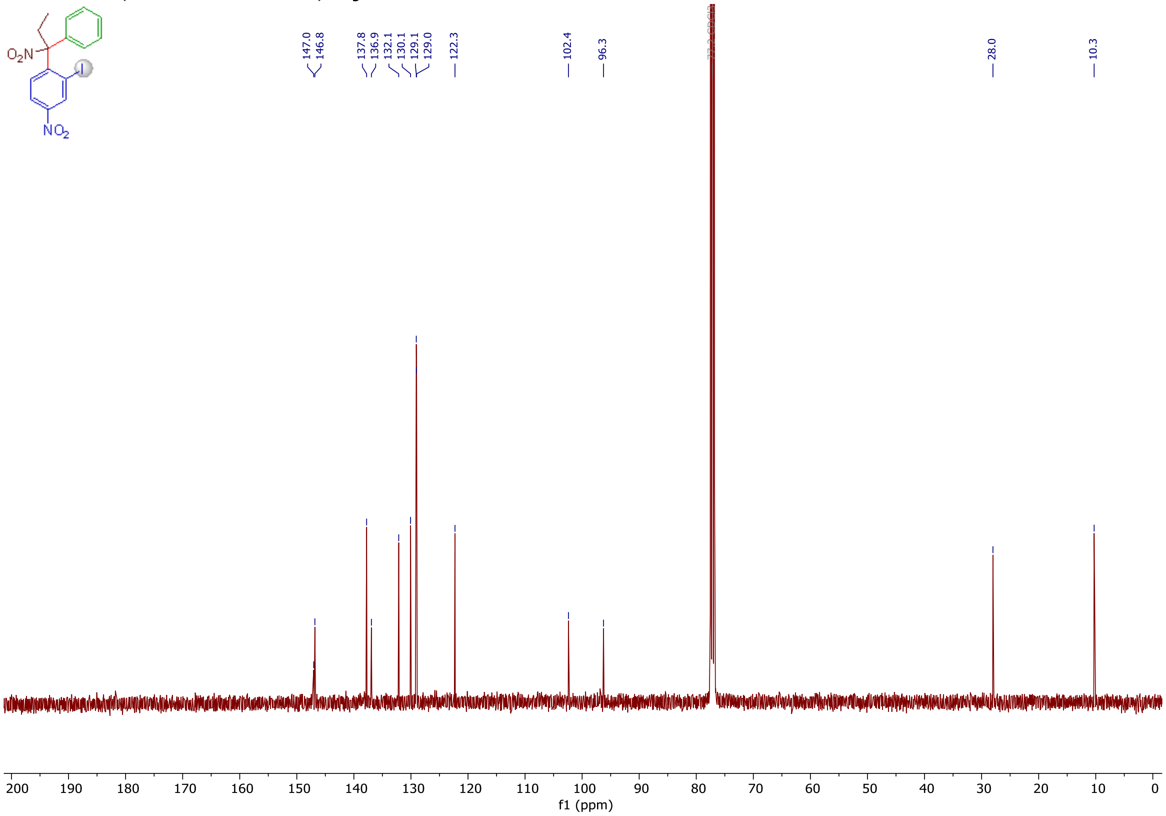

^{1}H NMR (400 MHz, $CDCl_3$), 6a:

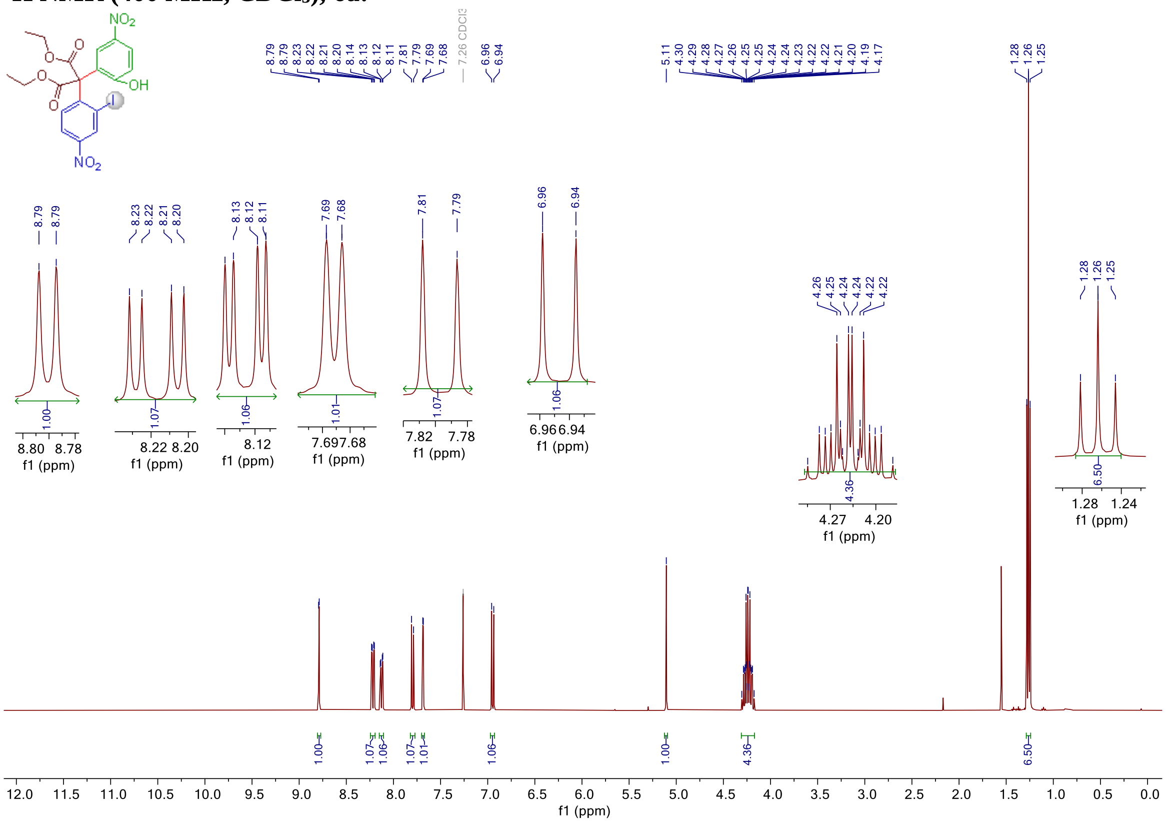

^{13}C NMR (101 MHz, $CDCl_3$), 6a:

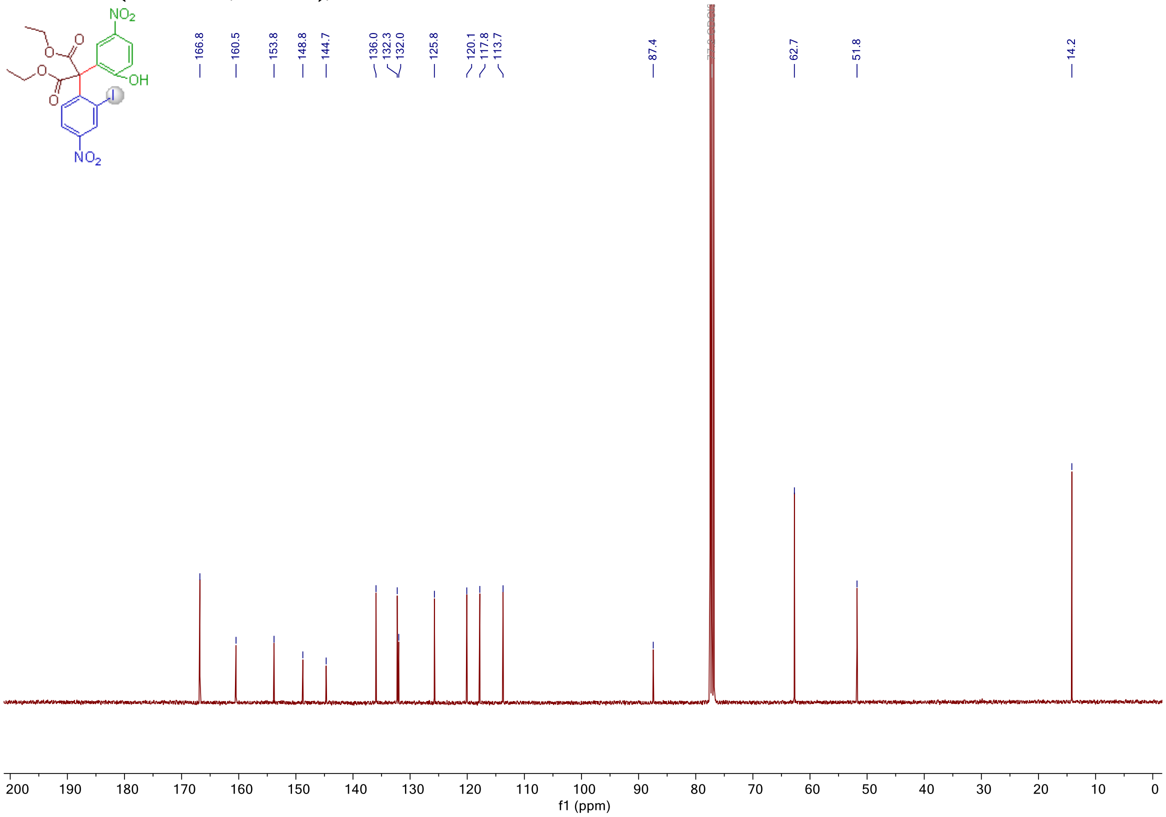

^{1}H NMR (400 MHz, CDCl$_3$), 6b:

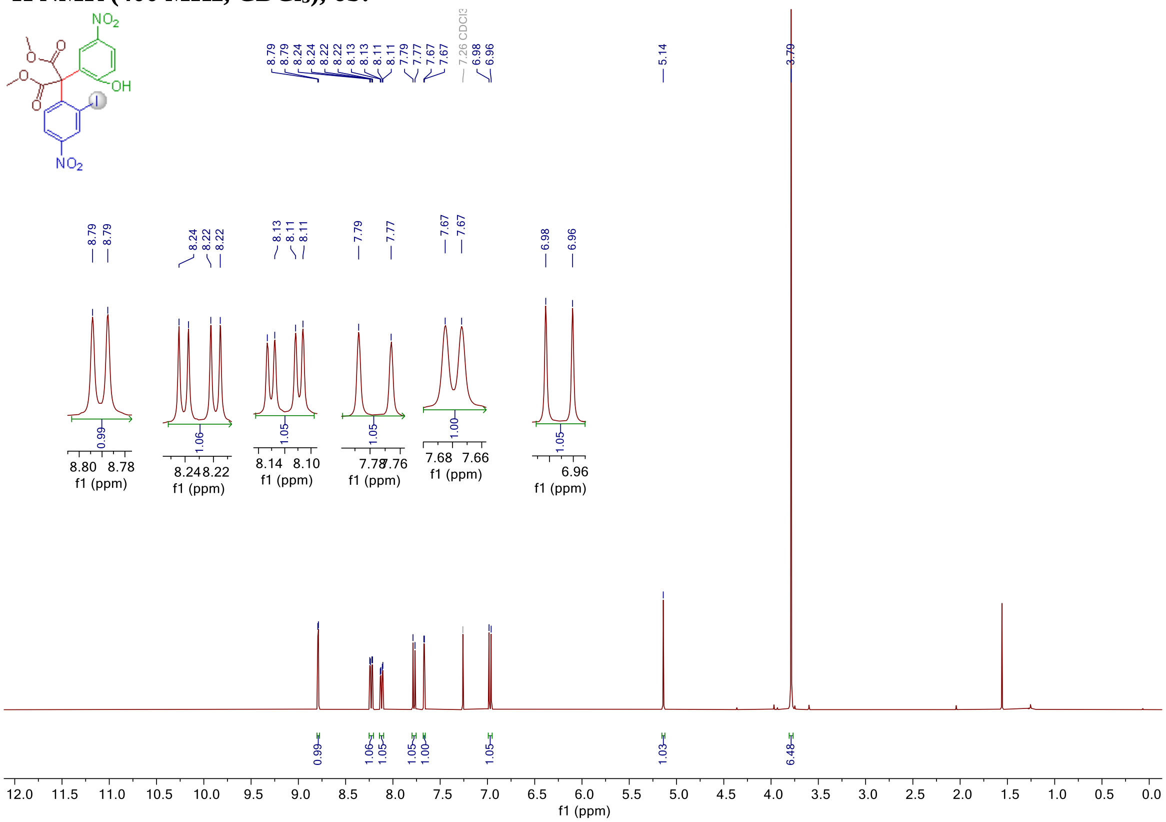

^{13}C NMR (101 MHz, CDCl$_3$), 6b:

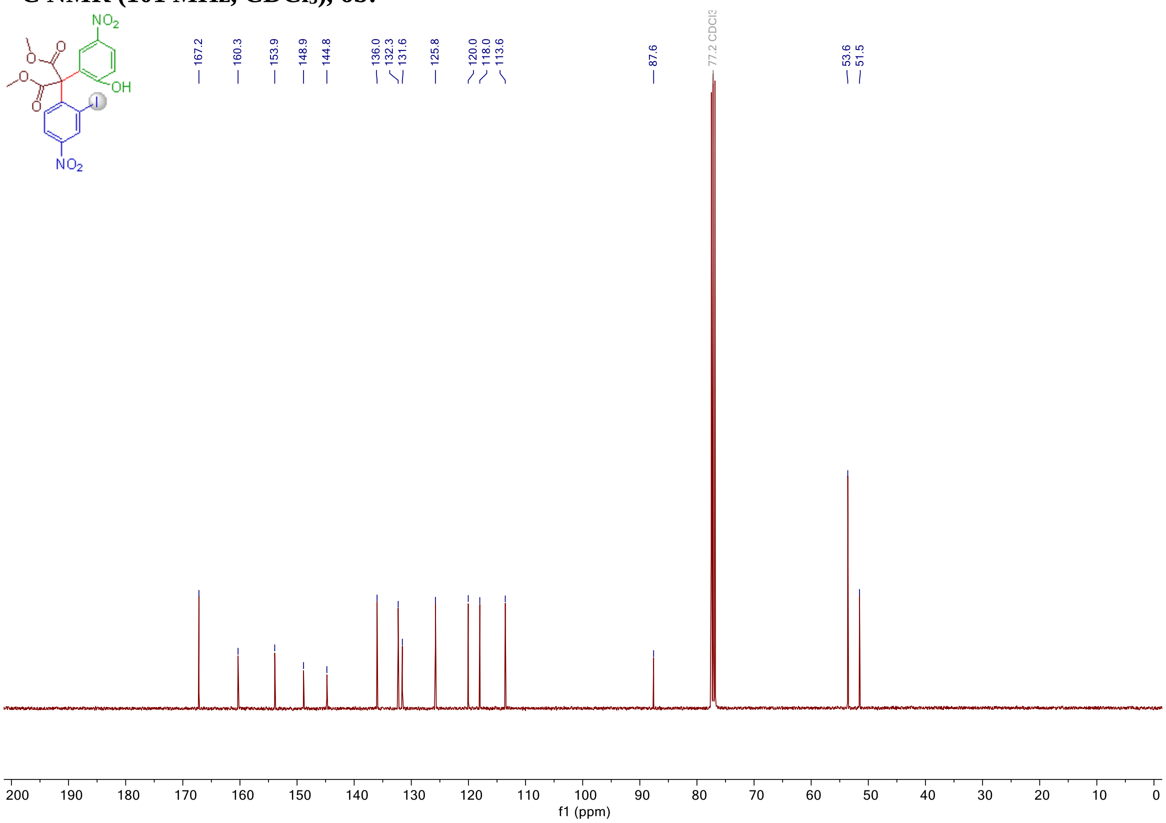

^{1}H NMR (400 MHz, CDCl3), 6c:

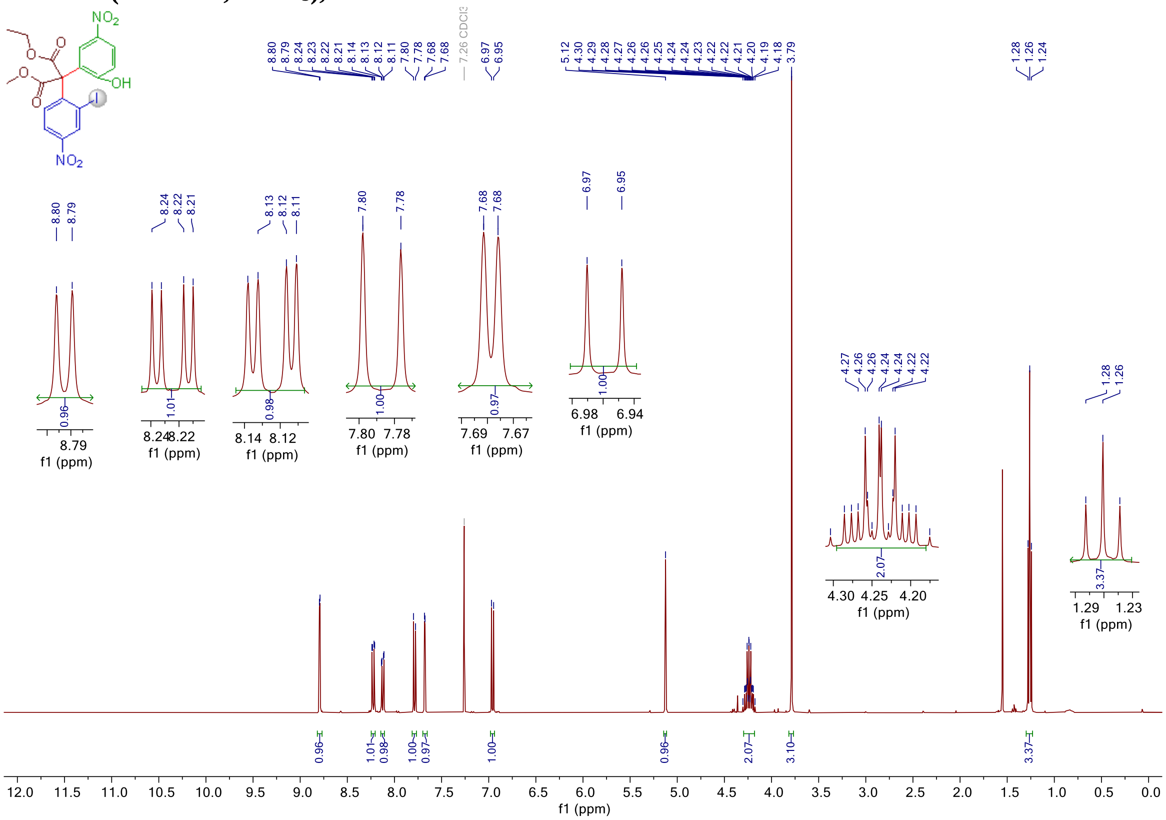

^{13}C NMR (101 MHz, CDCl3), 6c:

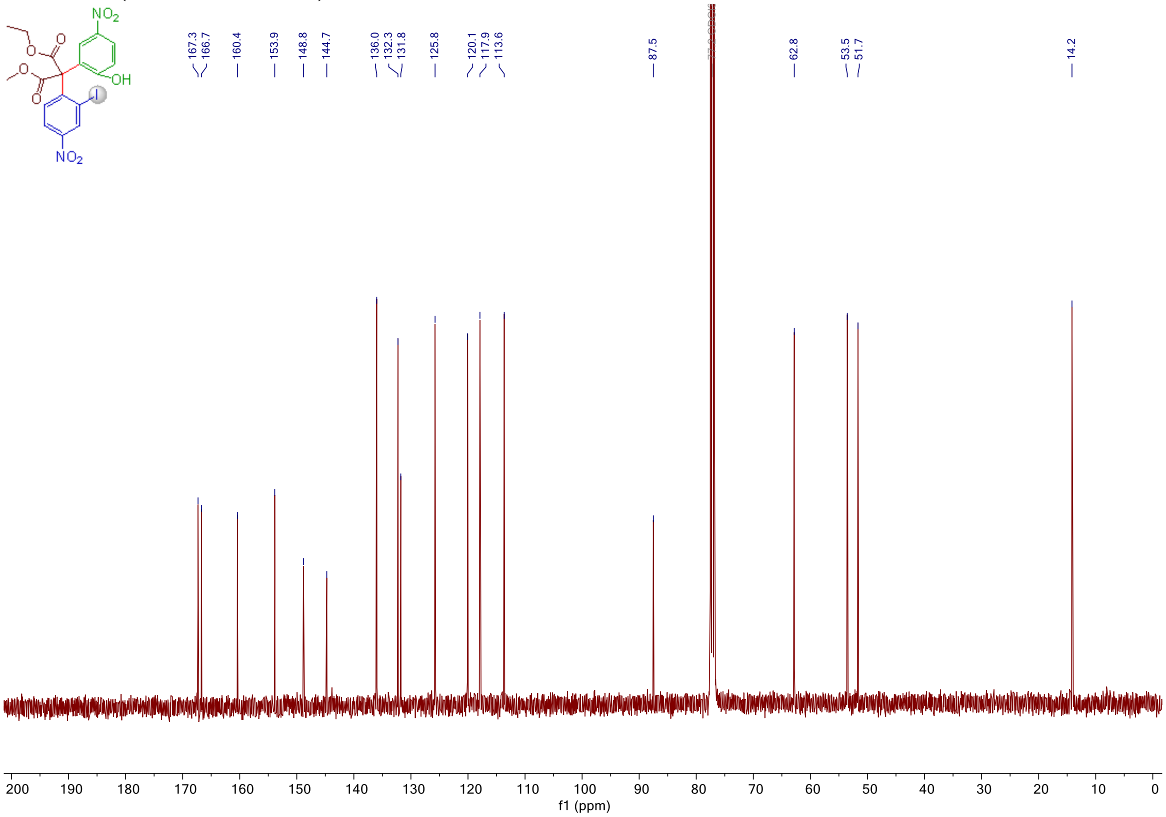

1H NMR (400 MHz, $CDCl_3$), 7a:

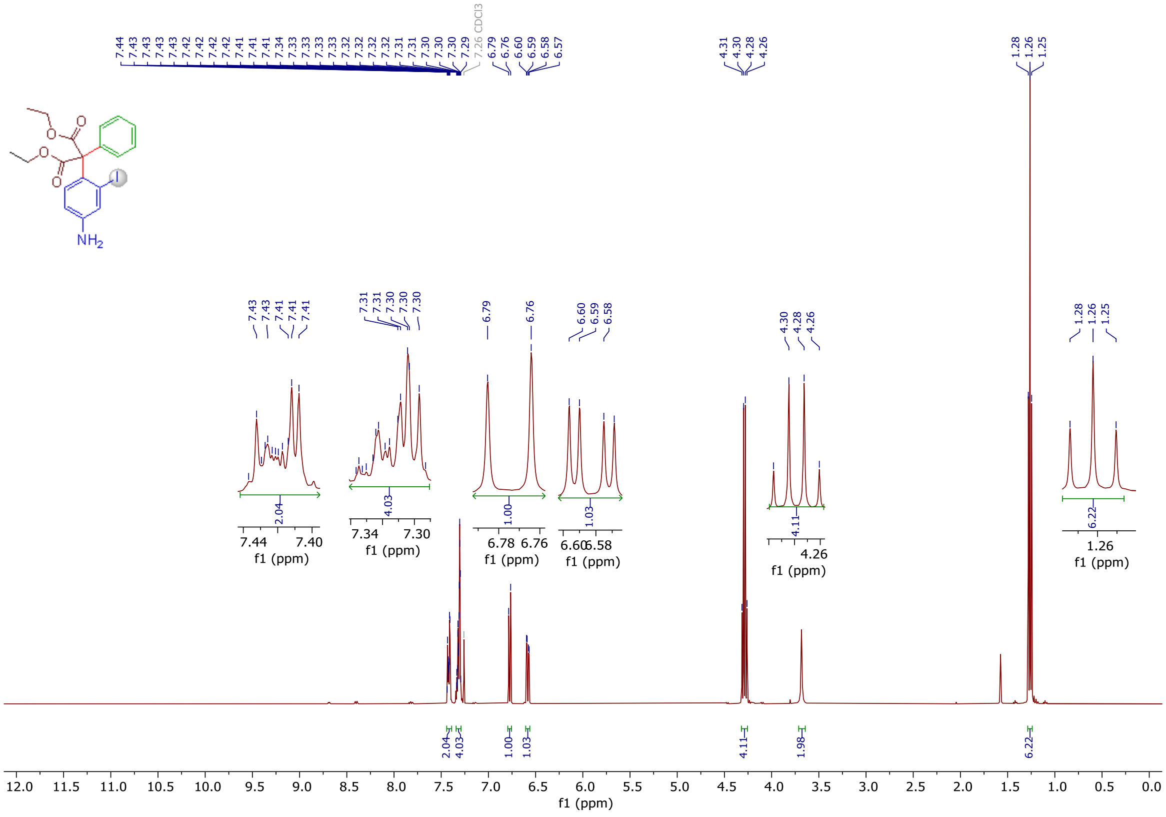

13C NMR (101 MHz, $CDCl_3$), 7a:

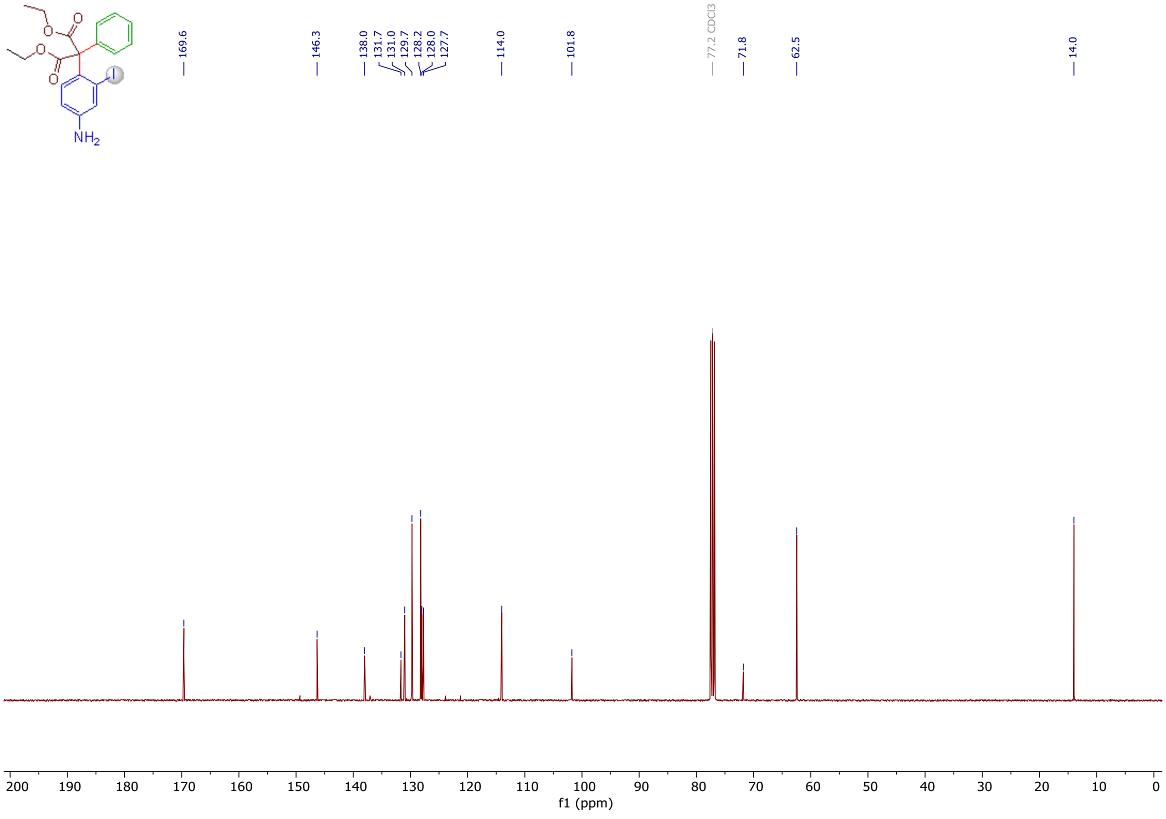

^{1}H NMR (400 MHz, $CDCl_3$), 7b:

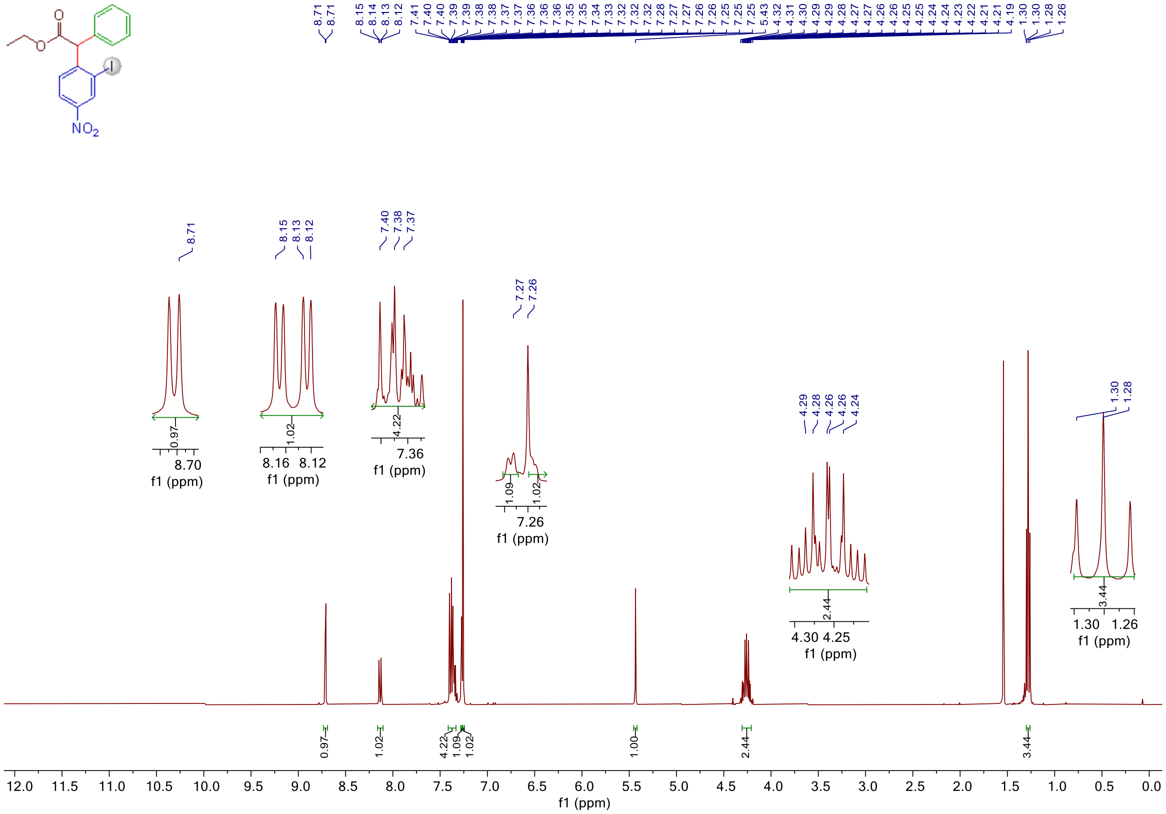

^{13}C NMR (101 MHz, $CDCl_3$), 7b:

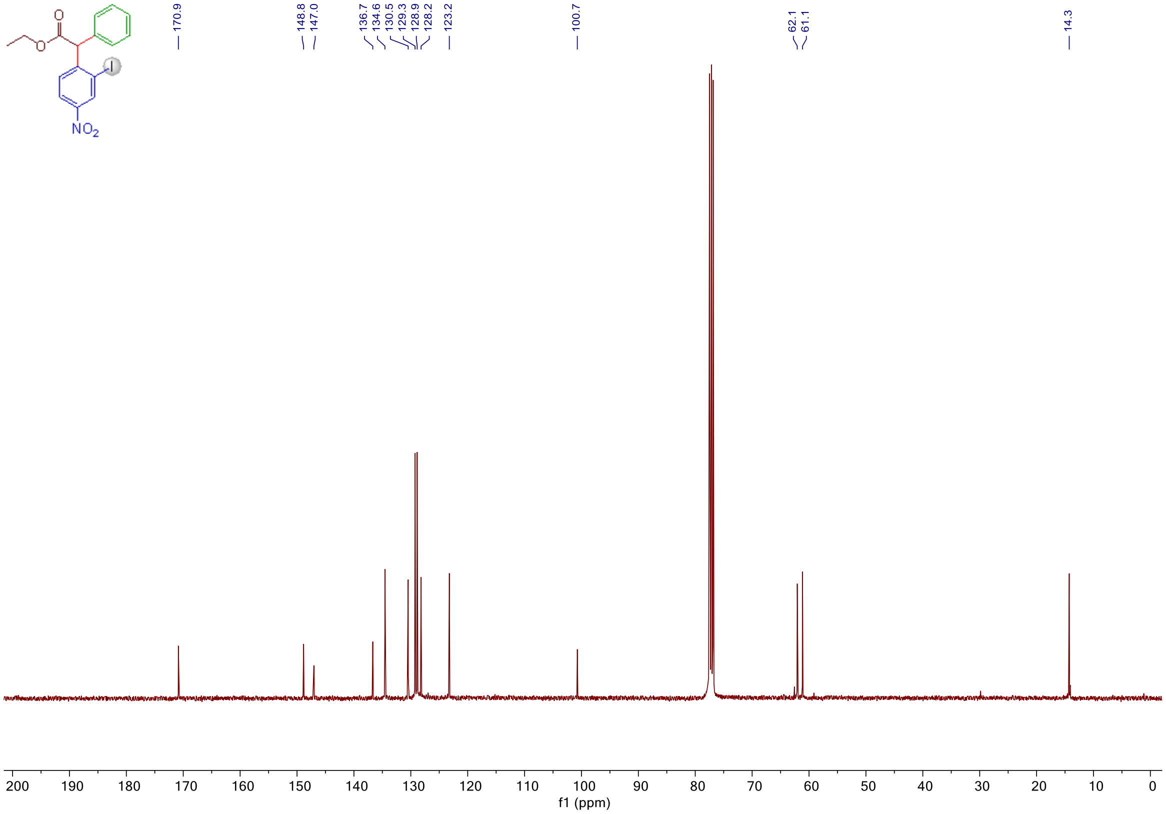

^{1}H NMR (400 MHz, $CDCl_3$), 7c:

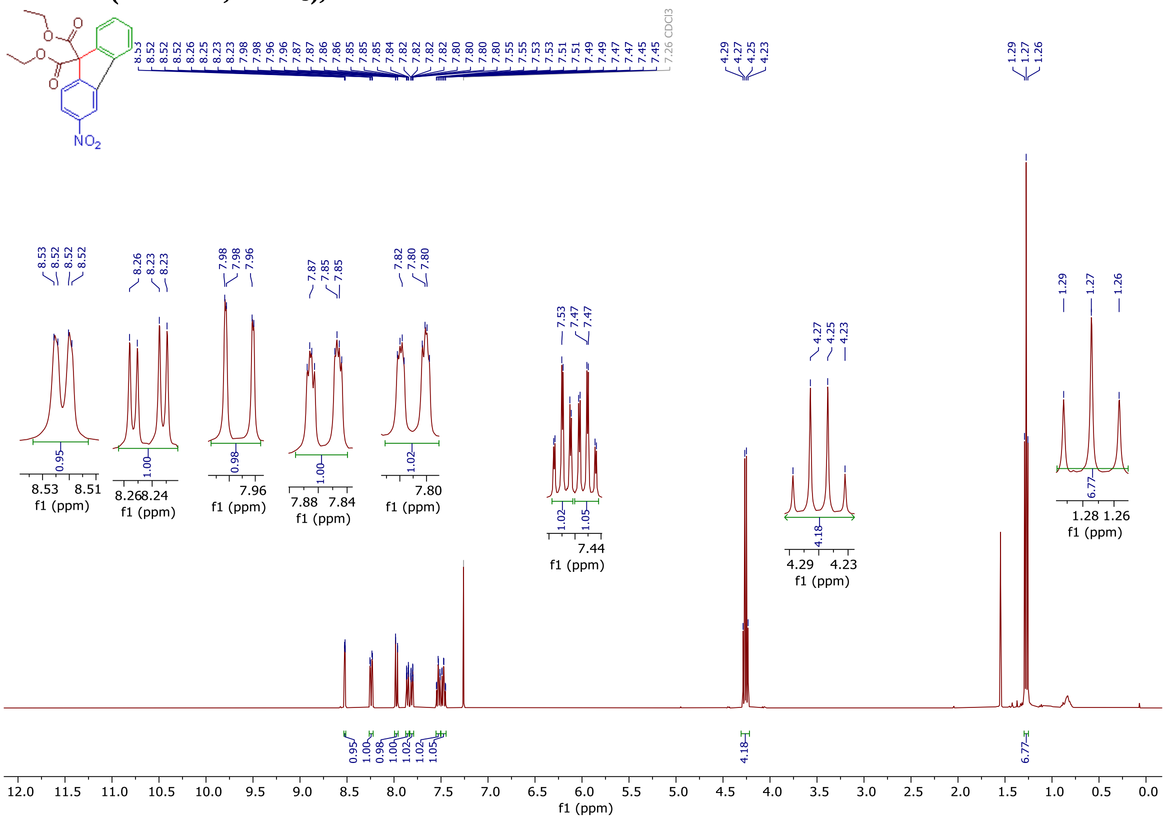

^{13}C NMR (101 MHz, $CDCl_3$), 7c:

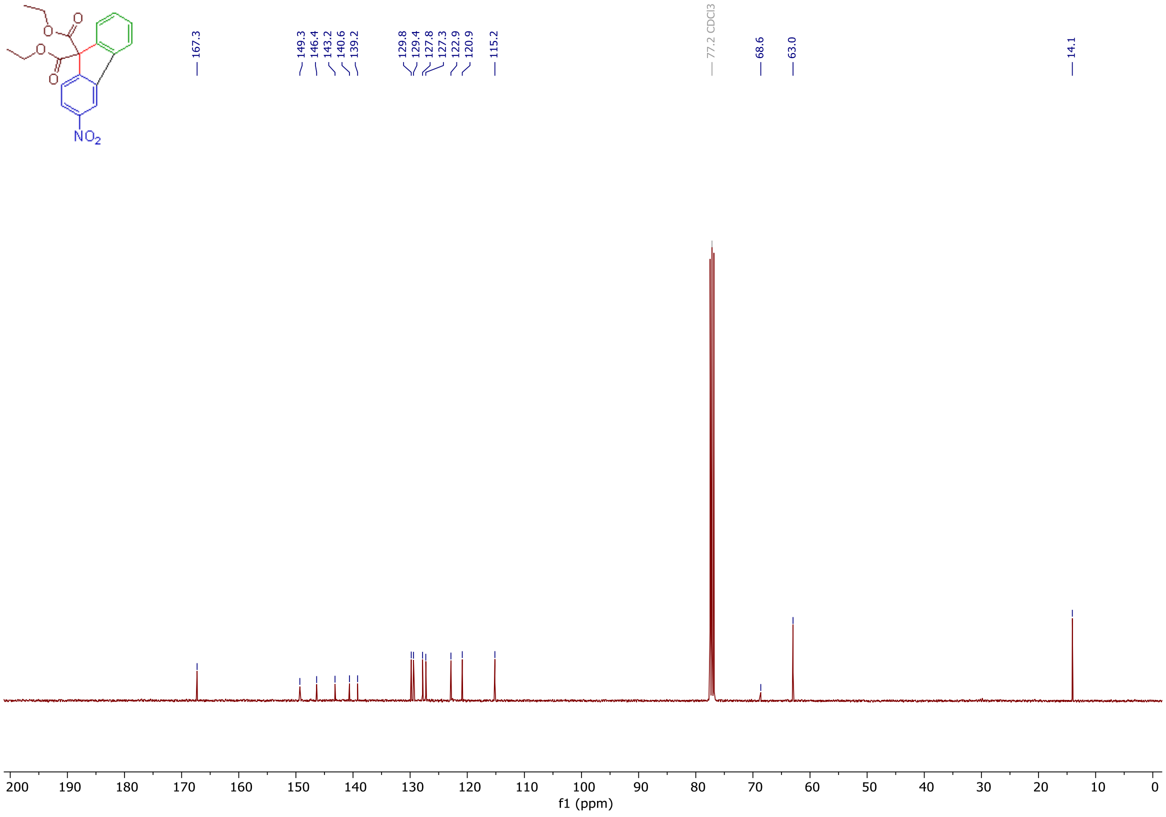

^{1}H NMR (400 MHz, $CDCl_3$), 7d:

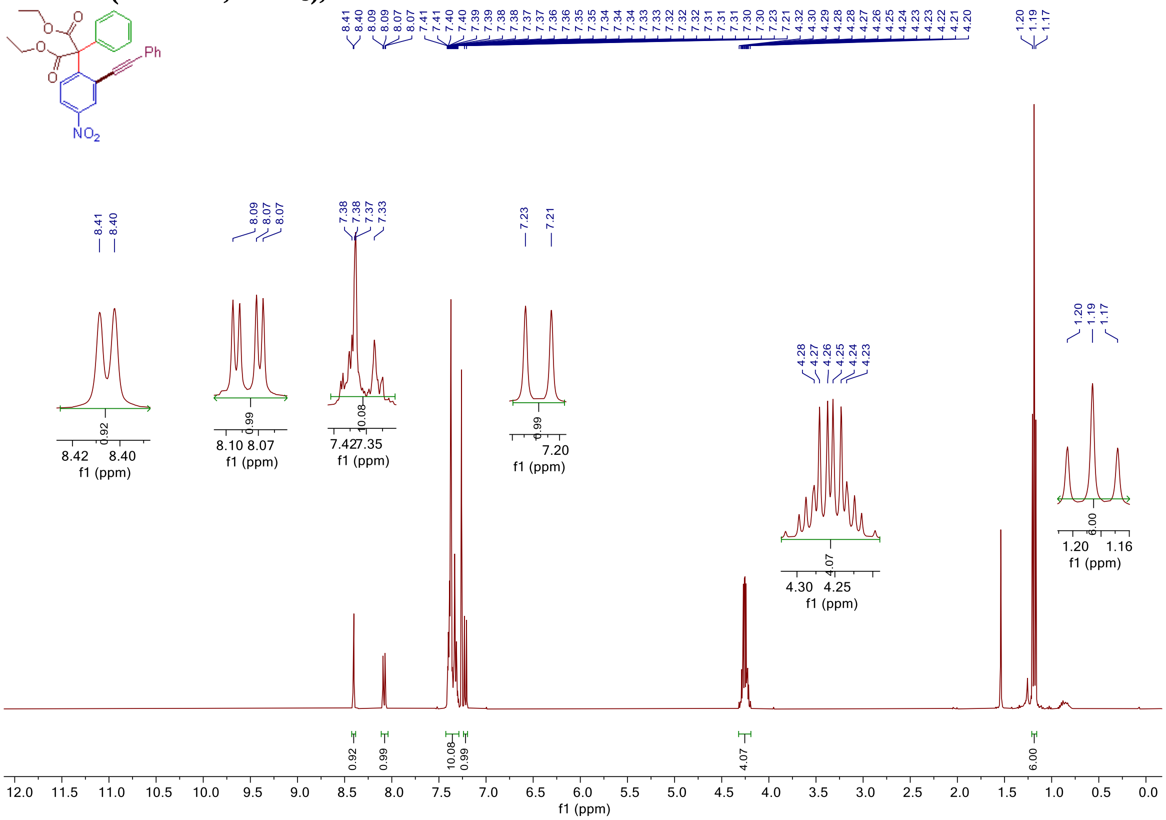

^{13}C NMR (101 MHz, $CDCl_3$), 7d:

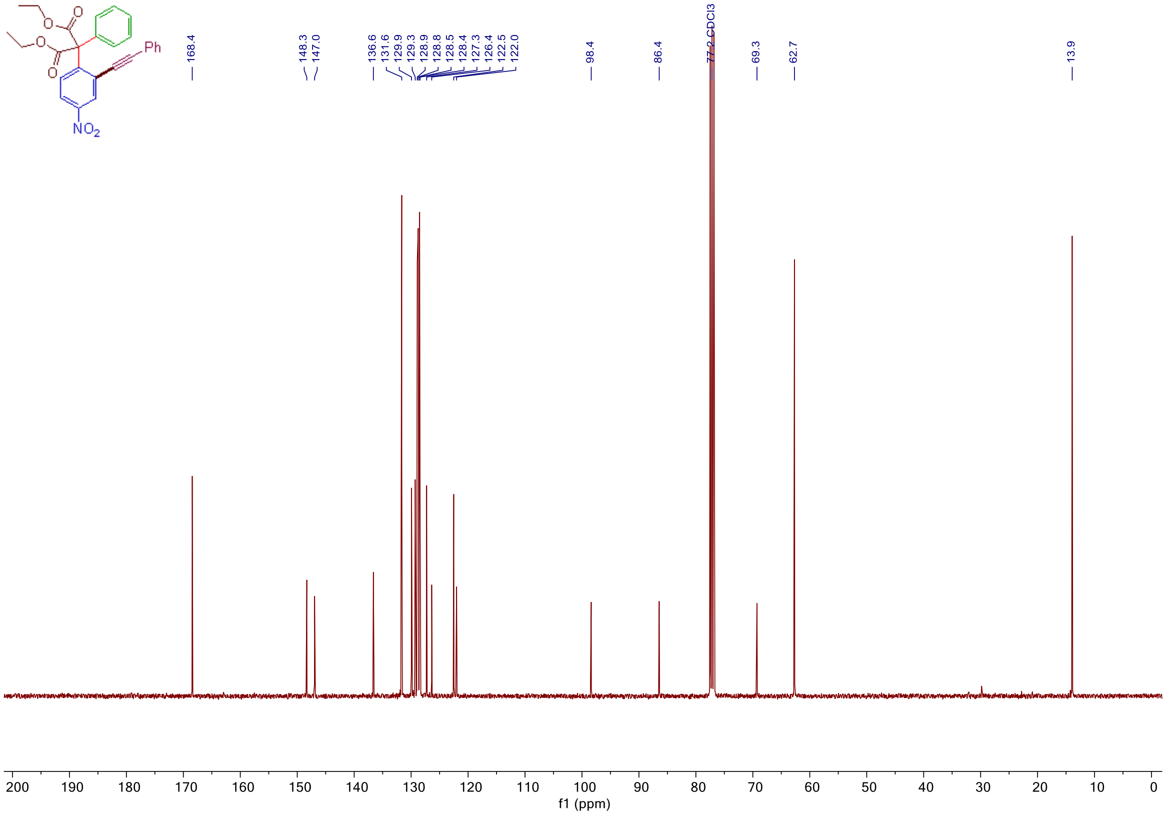

^{1}H NMR (400 MHz, DMSO-d_6), 8a:

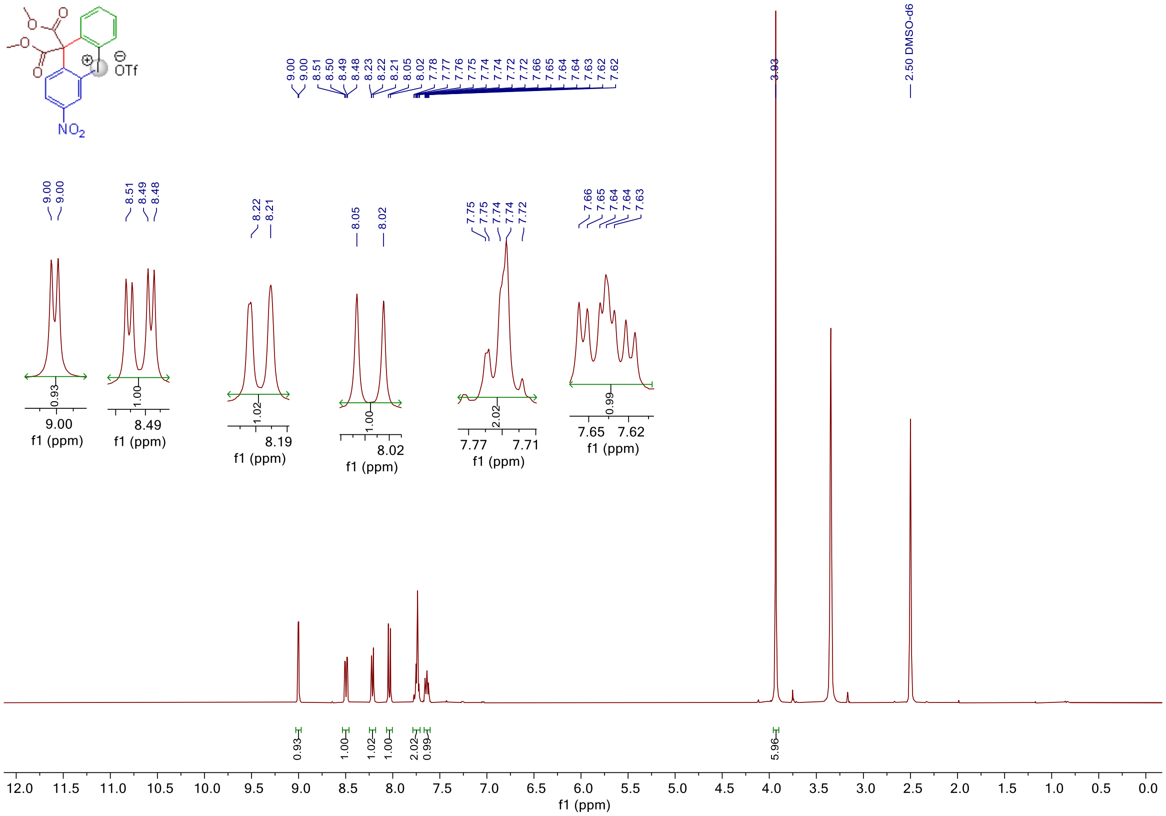

^{13}C NMR (101 MHz, DMSO-d_6), 8a:

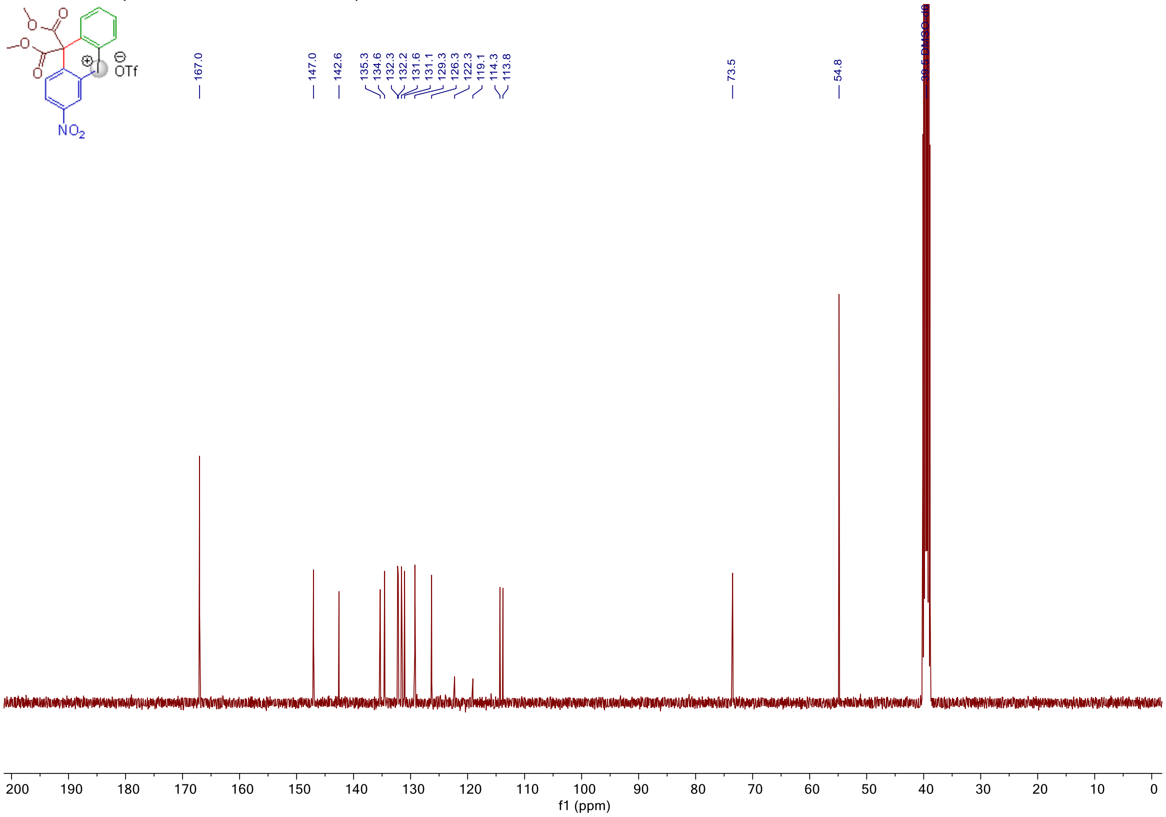

^{19}F NMR (377 MHz, DMSO-d_6), 8a:

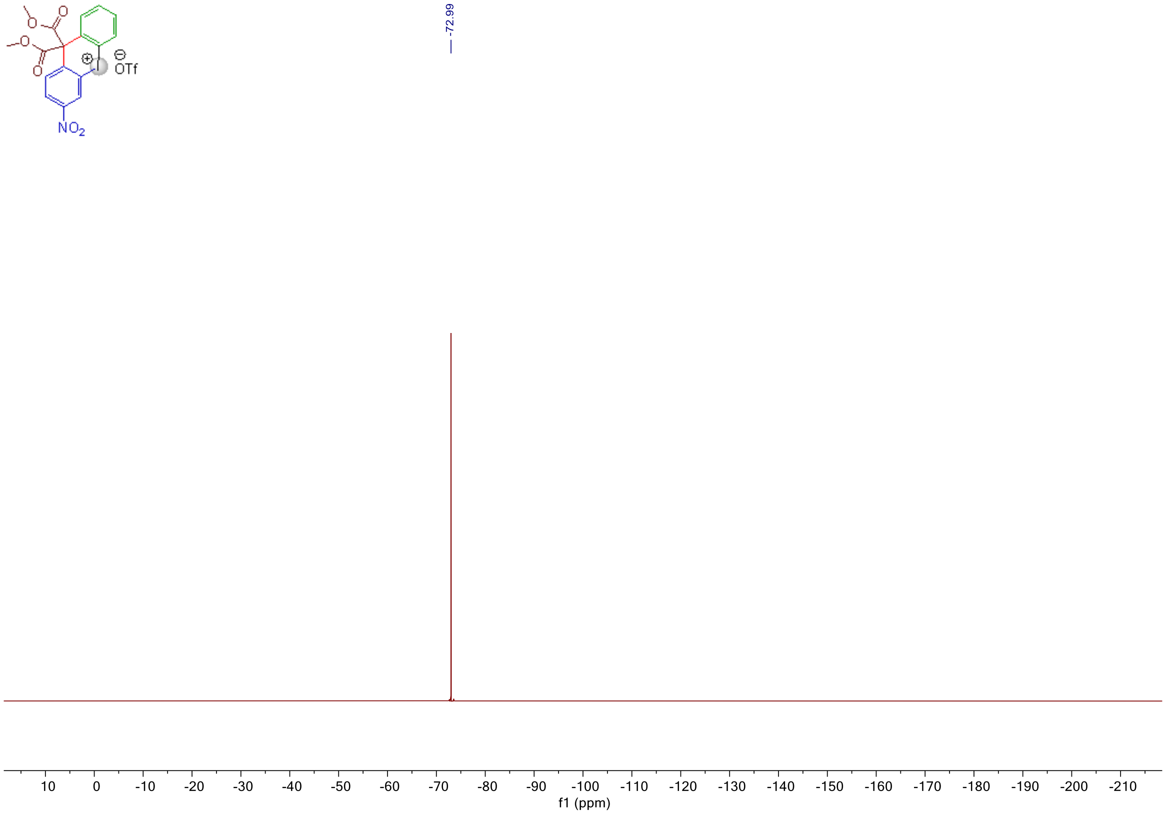

^{1}H NMR (400 MHz, DMSO-d_6), 8b:

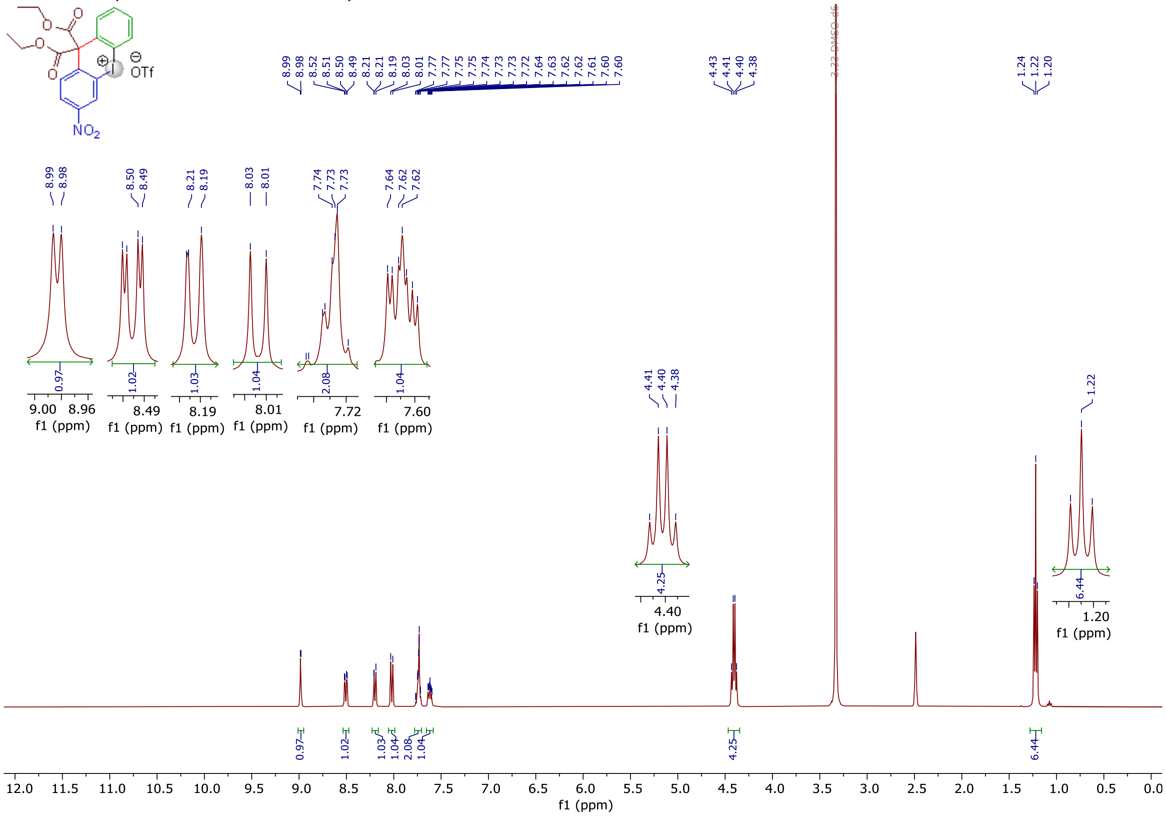

^{19}F NMR (377 MHz, DMSO-d_6), 8b:

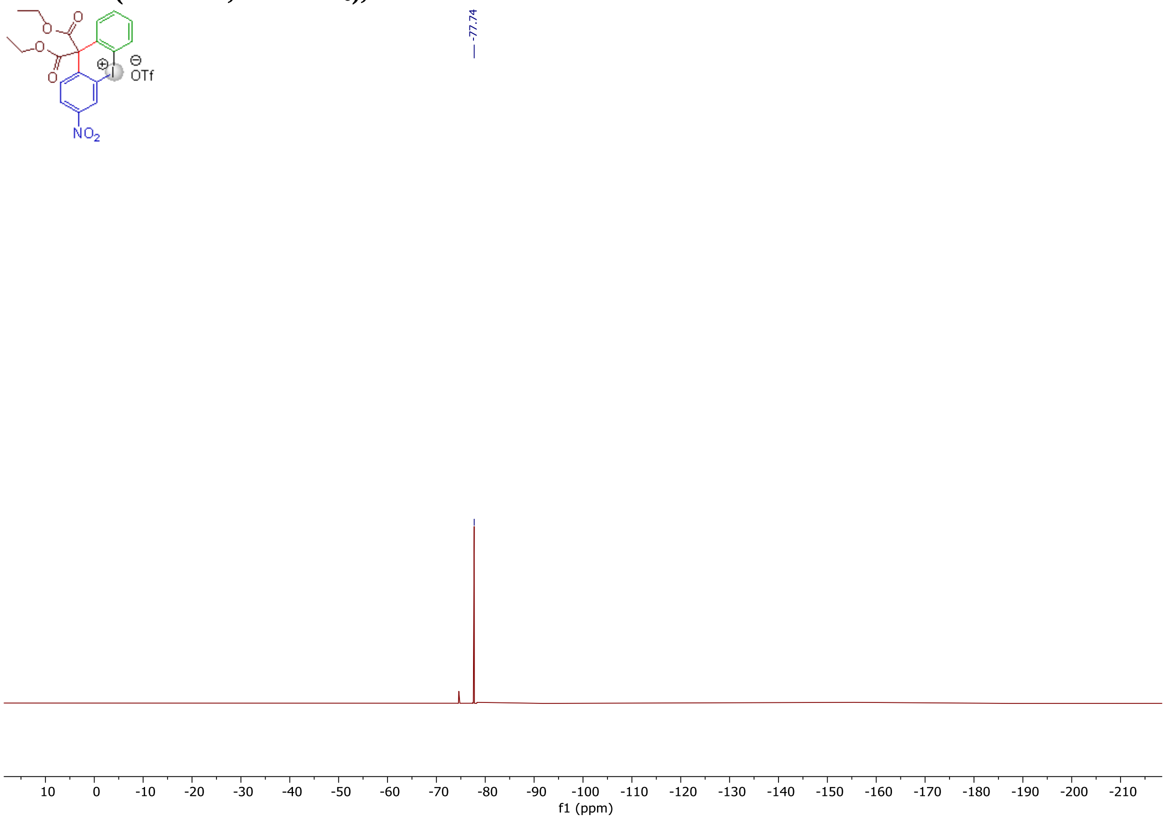

^{13}C NMR (101 MHz, DMSO-d_6), 8b:

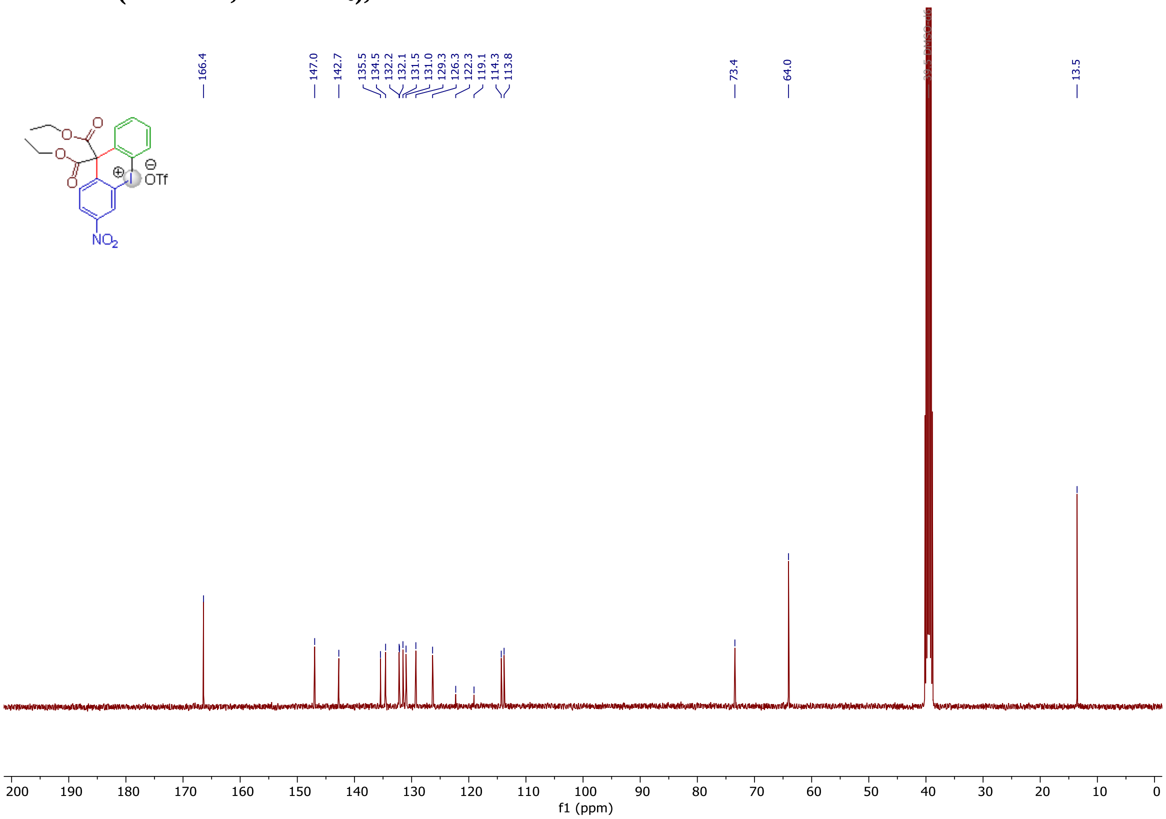

^{1}H NMR (400 MHz, MeOD-d_4), 8c:

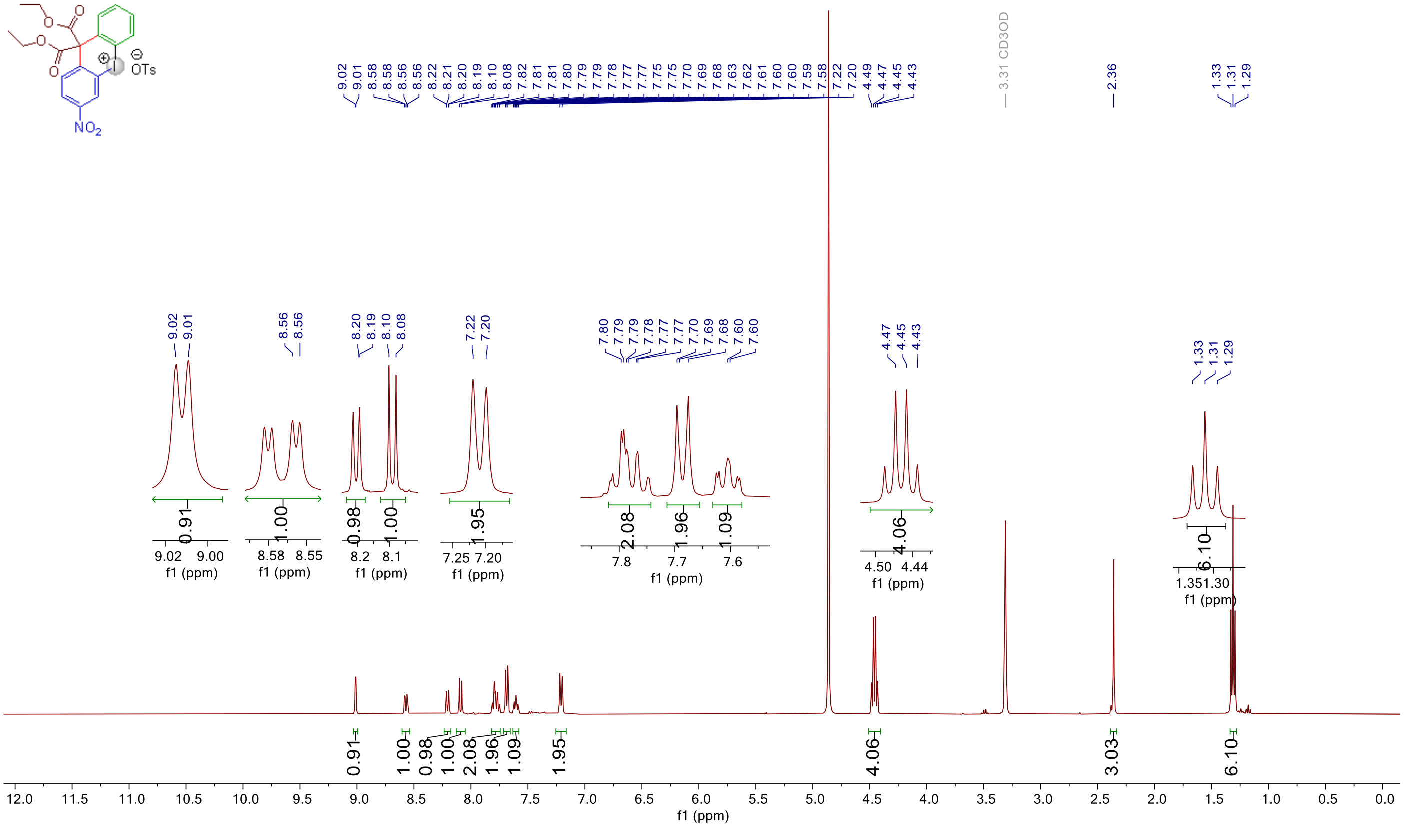

^{13}C NMR (101 MHz, MeOD-d_4), 8c:

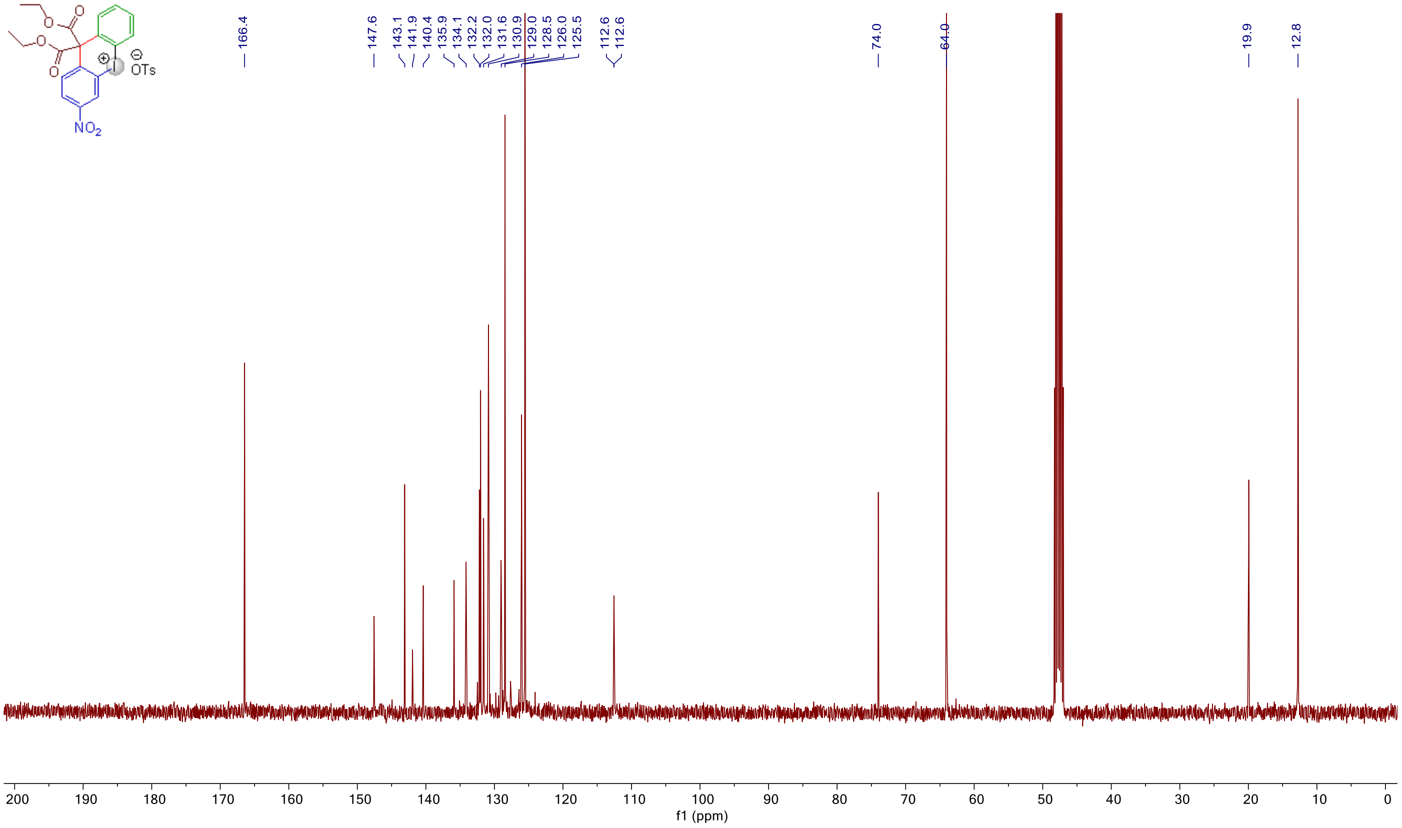

^{1}H NMR (400 MHz, DMSO-d_6), 8d:

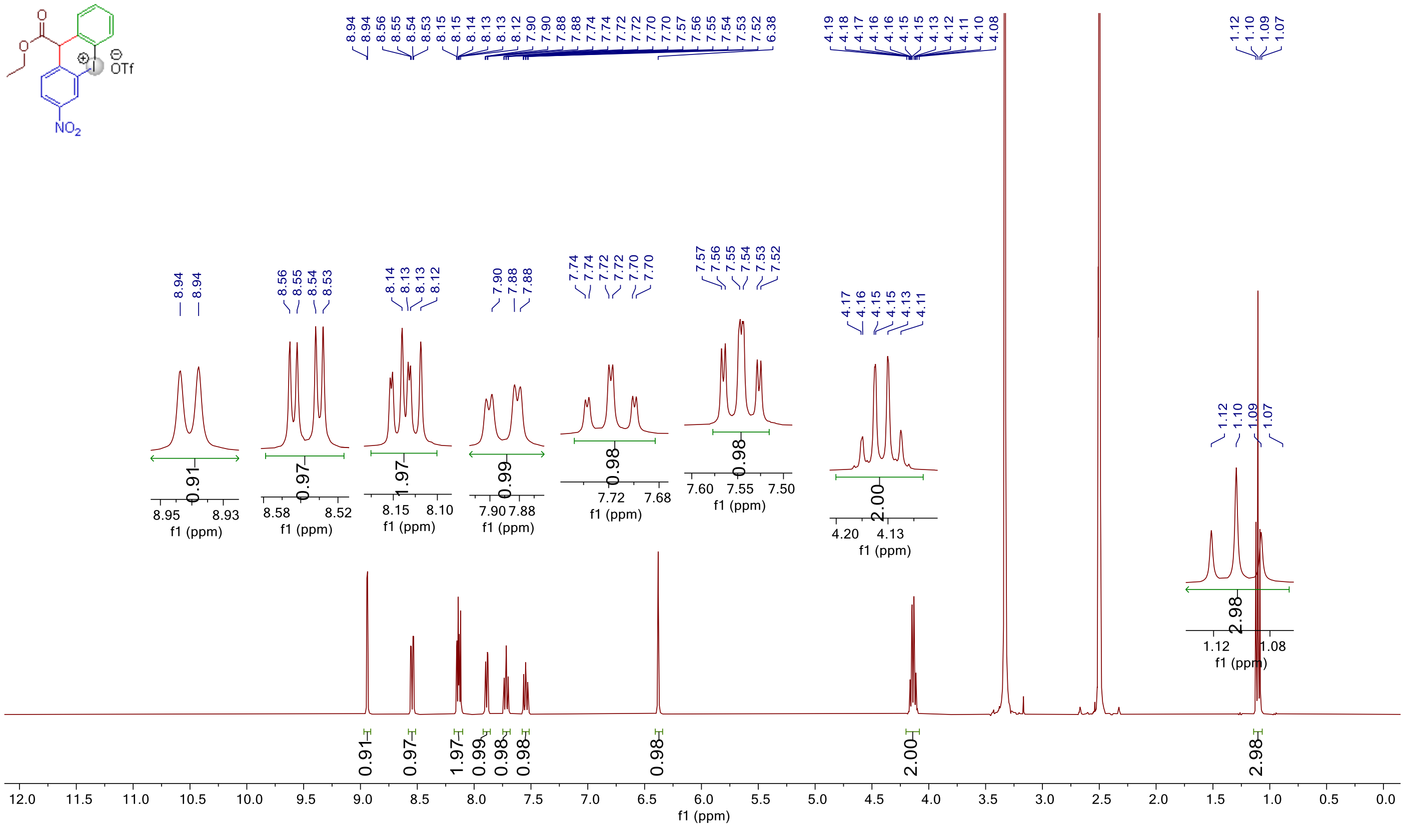

^{13}C NMR (101 MHz, DMSO-d_6), 8d:

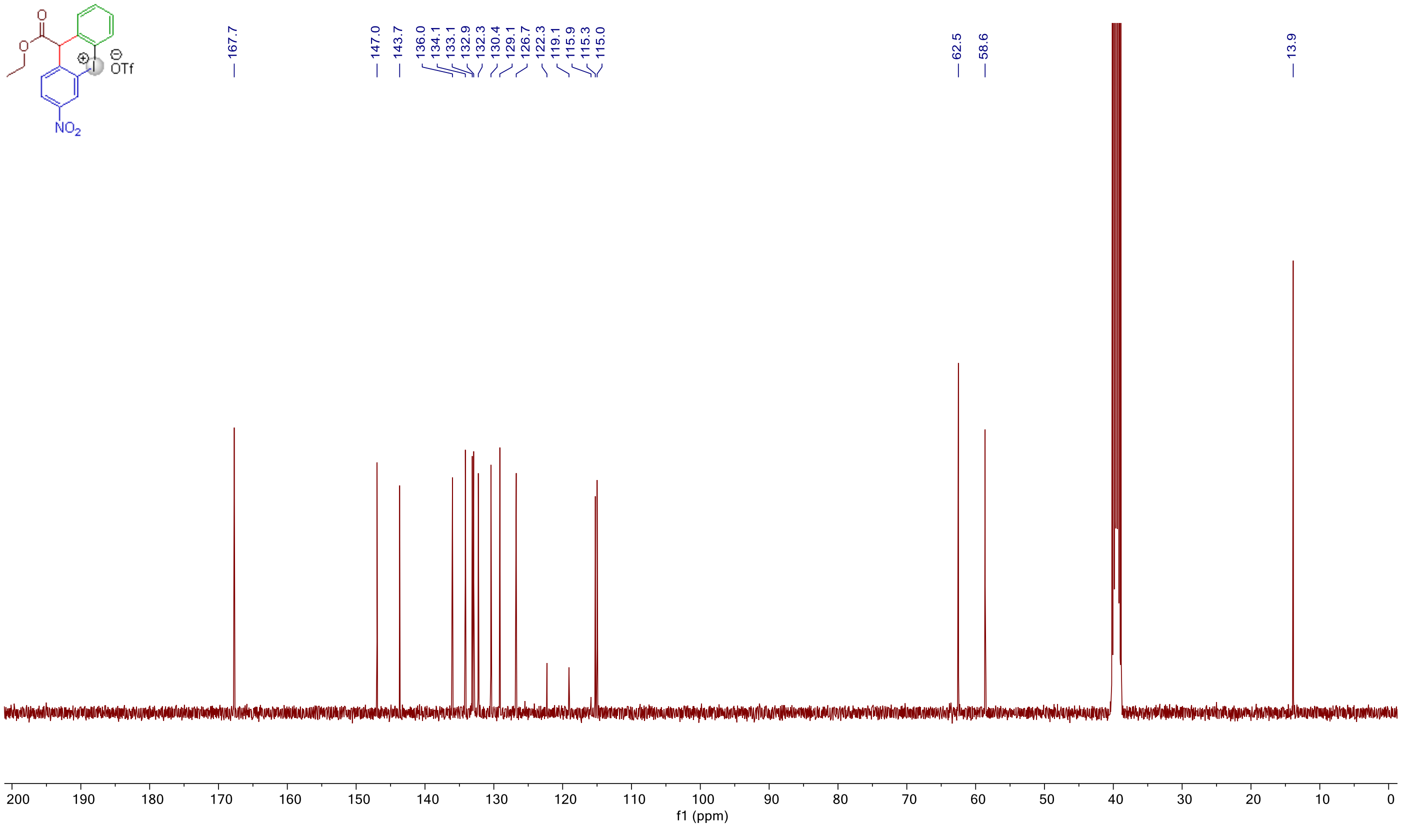

^{19}F NMR (377 MHz, DMSO-d_6), 8d:

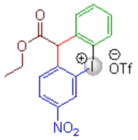

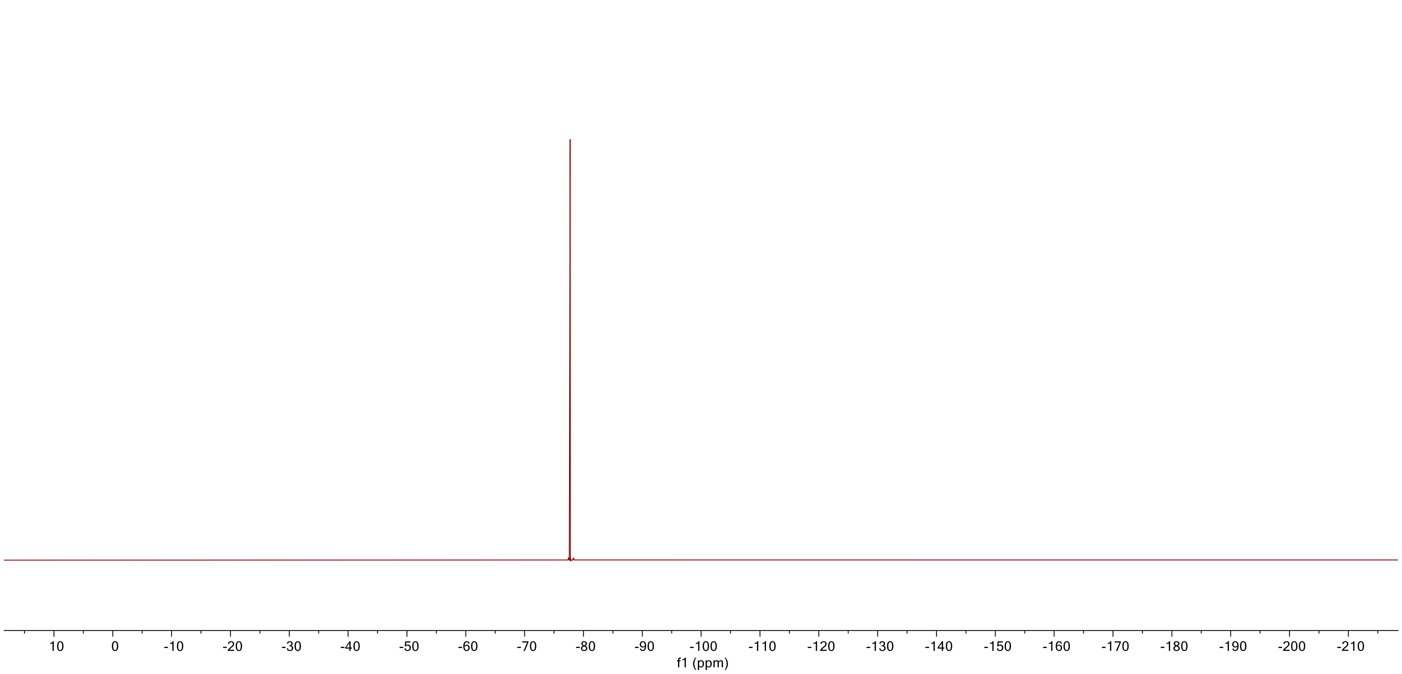

^{1}H NMR (400 MHz, $CDCl_3$), 7e:

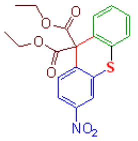

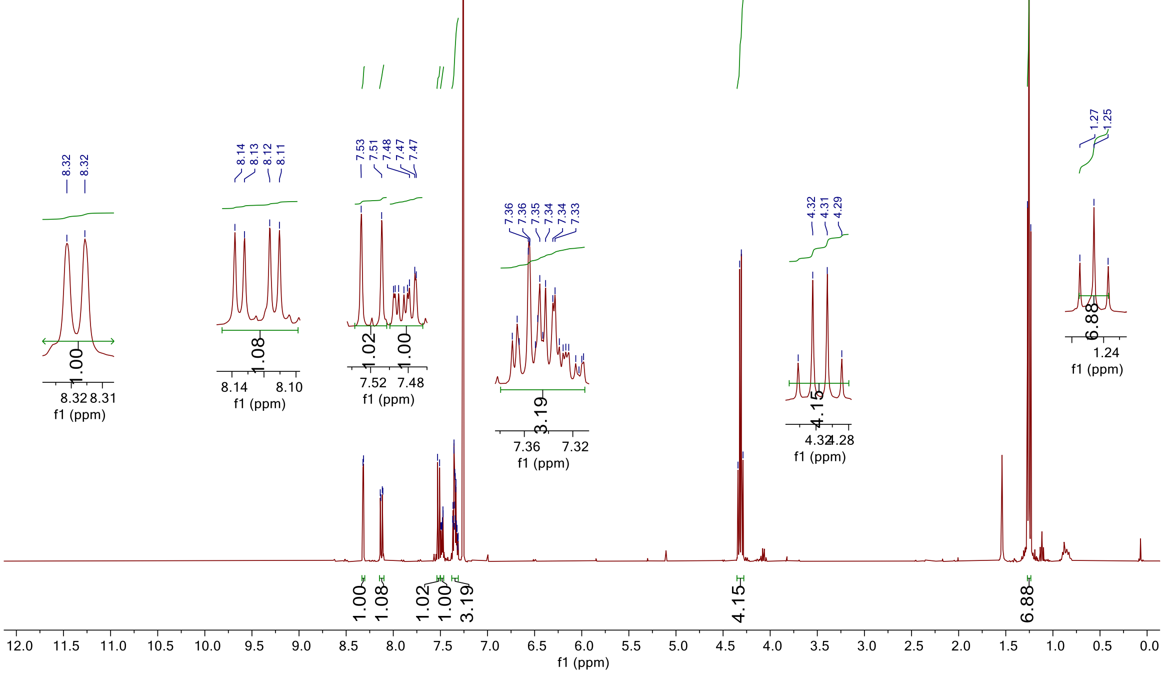

^{13}C NMR (101 MHz, CDCl₃), 7e:

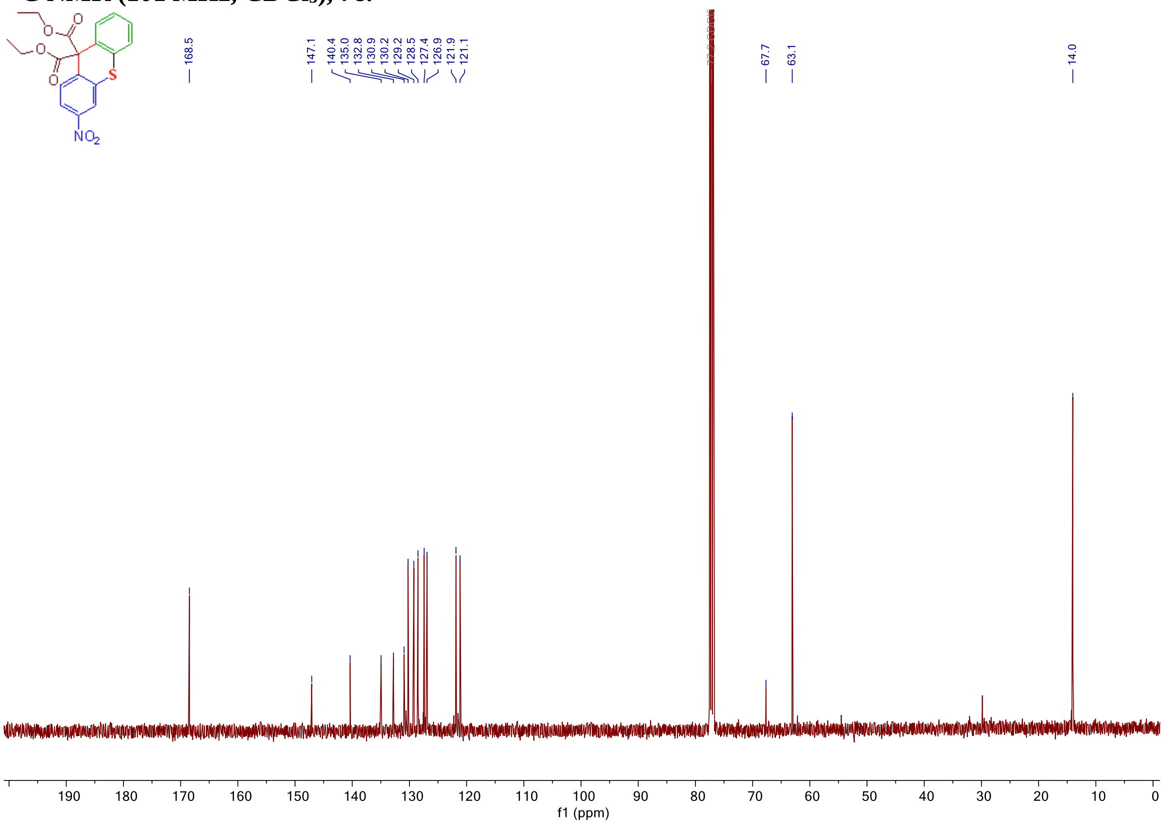

^{1}H NMR (400 MHz, CDCl₃), 7f:

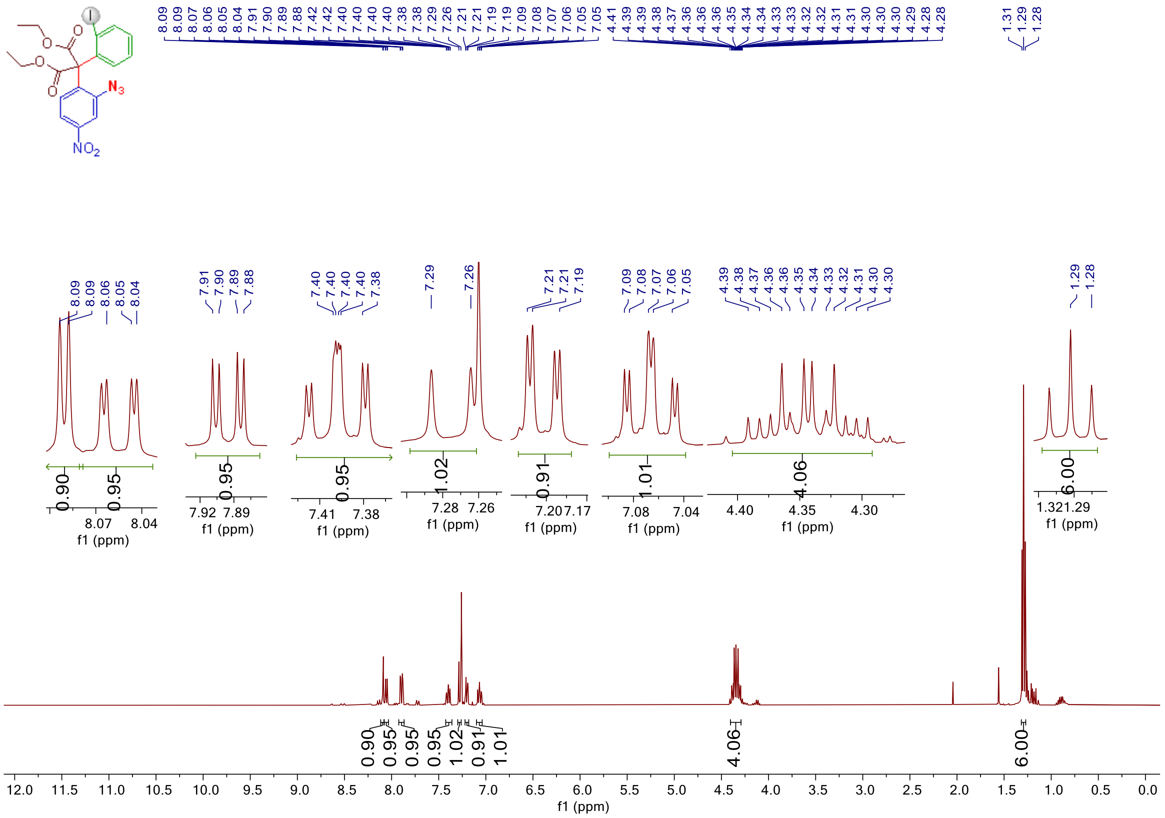

^{13}C NMR (101 MHz, CDCl$_3$), 7f:

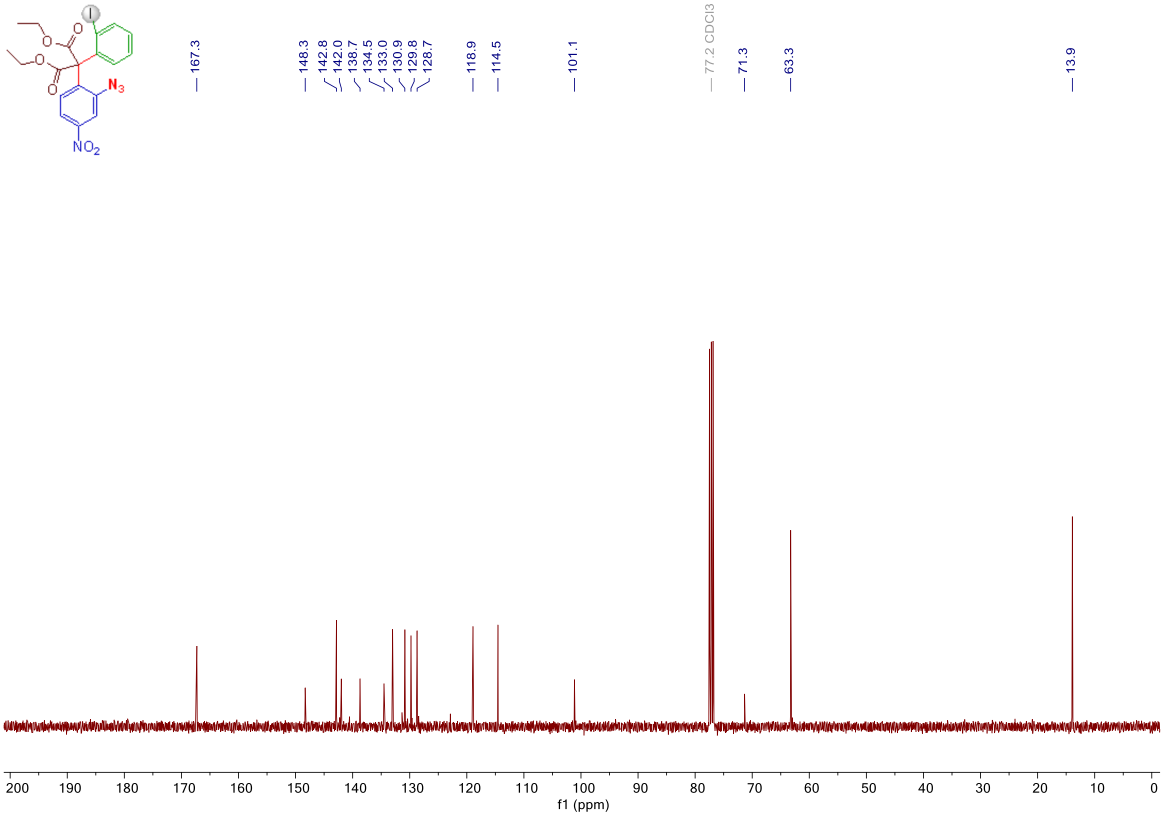

^{1}H NMR (400 MHz, DMSO-d_6), 1k:

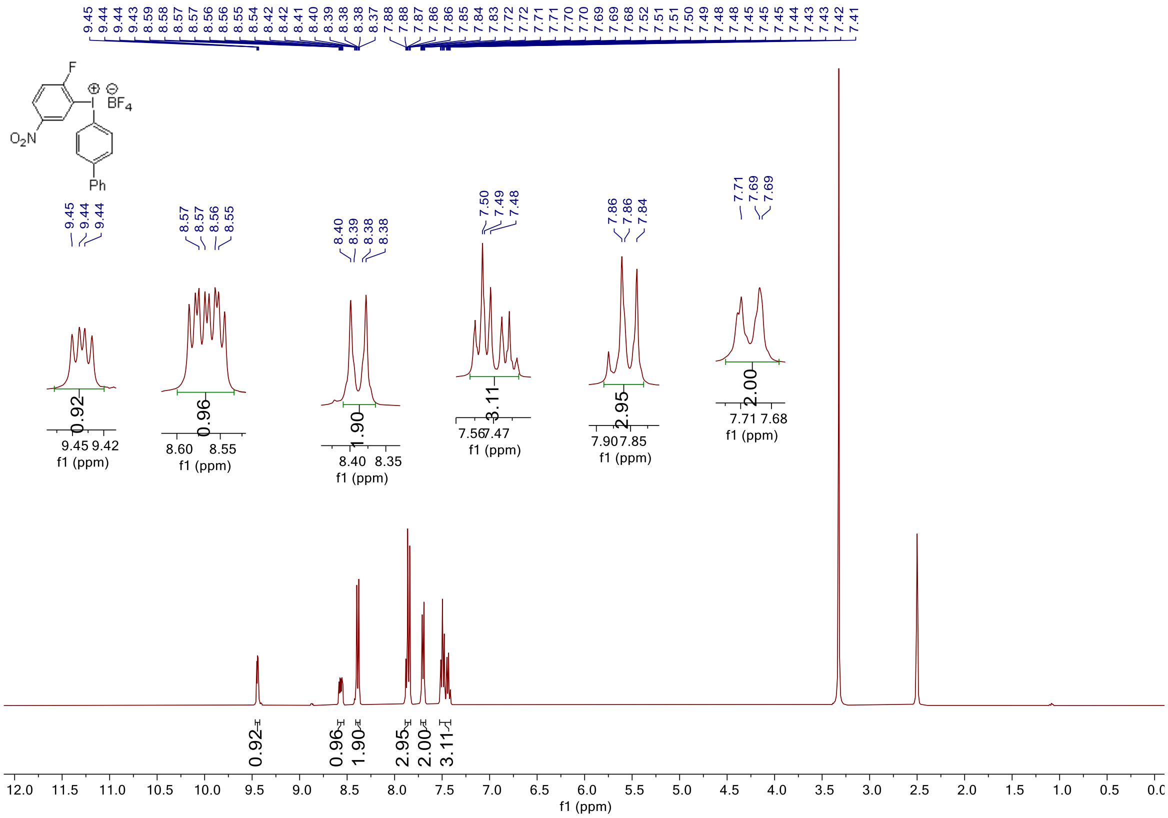

^{13}C NMR (101 MHz, DMSO-d_6), 1k:

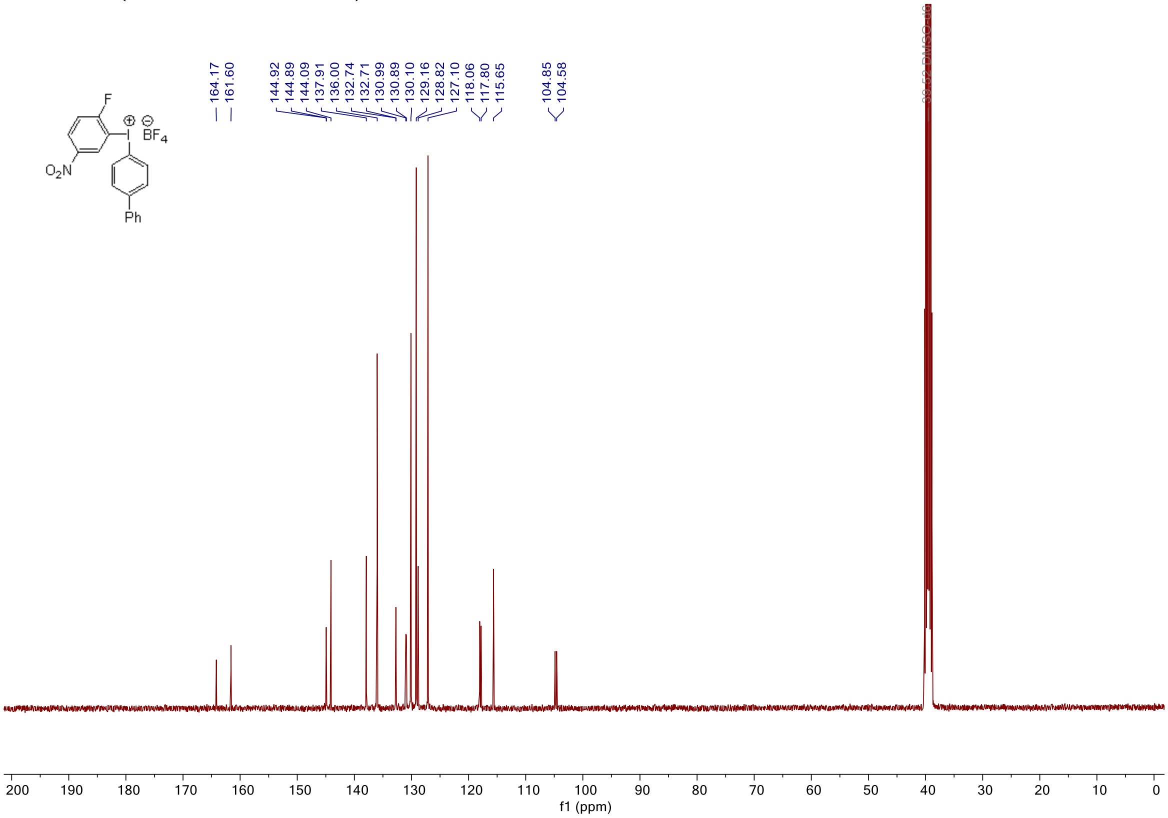

^{19}F NMR (377 MHz, DMSO-d_6), 1k:

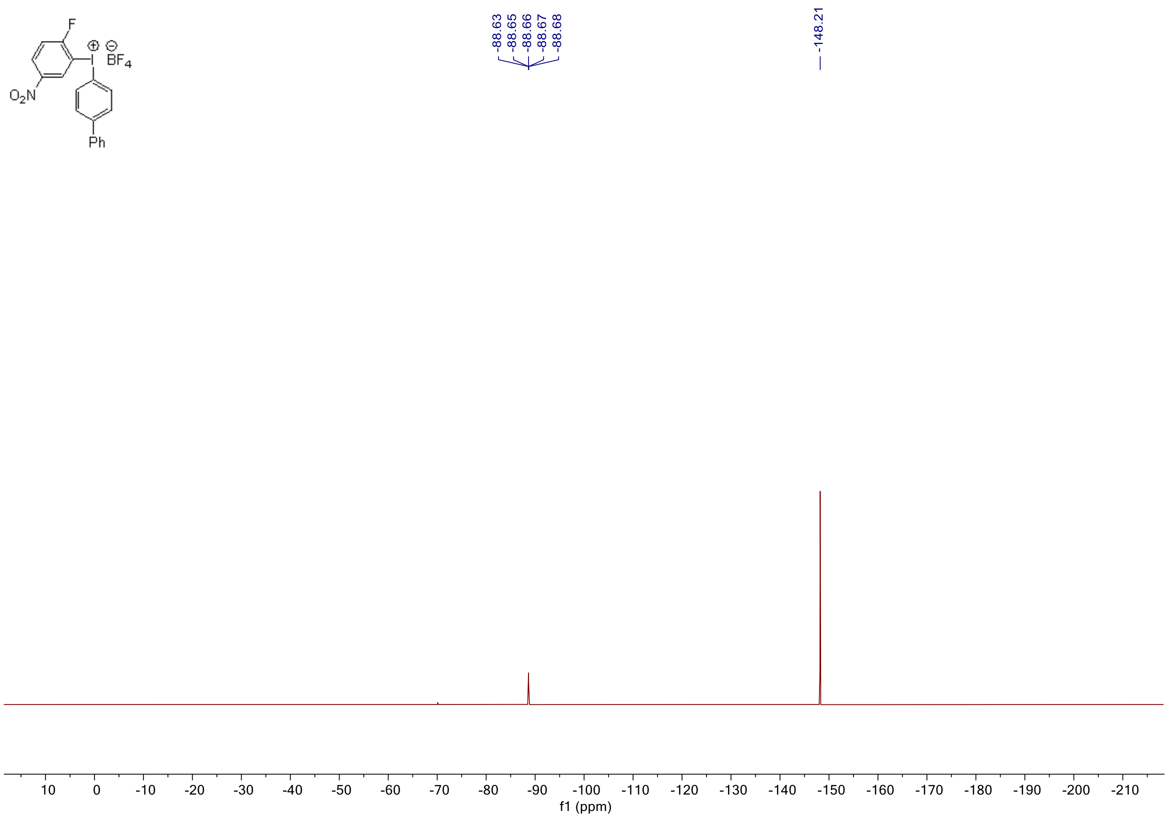

^{1}H NMR (400 MHz, DMSO-*d*$_6$), 5a:

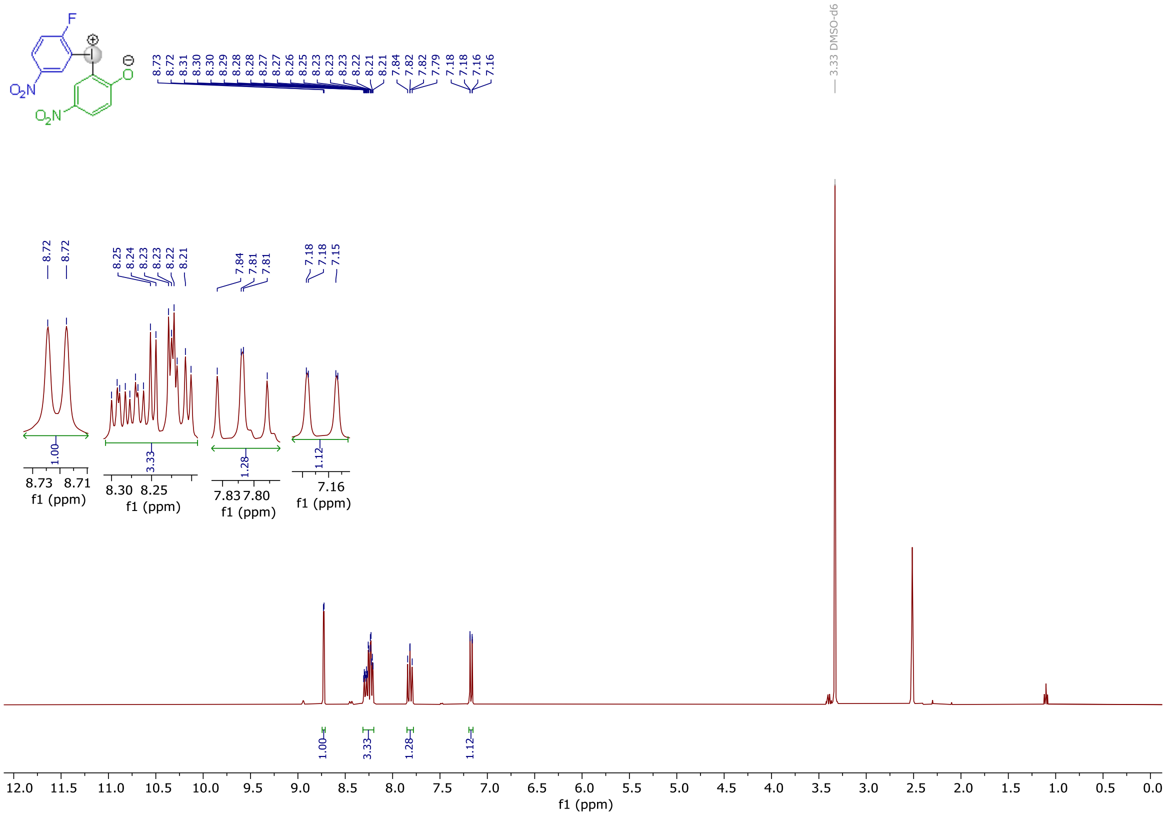

^{13}C NMR (101 MHz, DMSO-*d*$_6$), 5a:

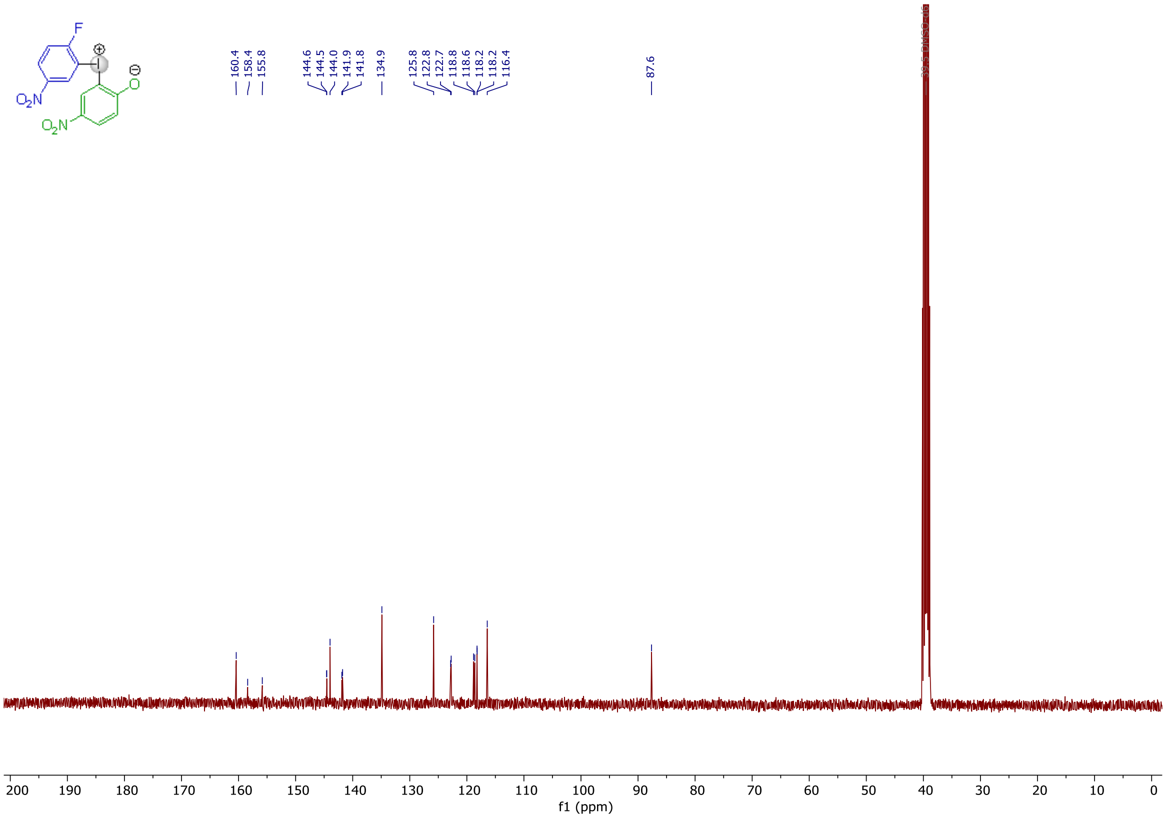

^{19}F NMR (377 MHz, DMSO-*d*$_6$), 5a:

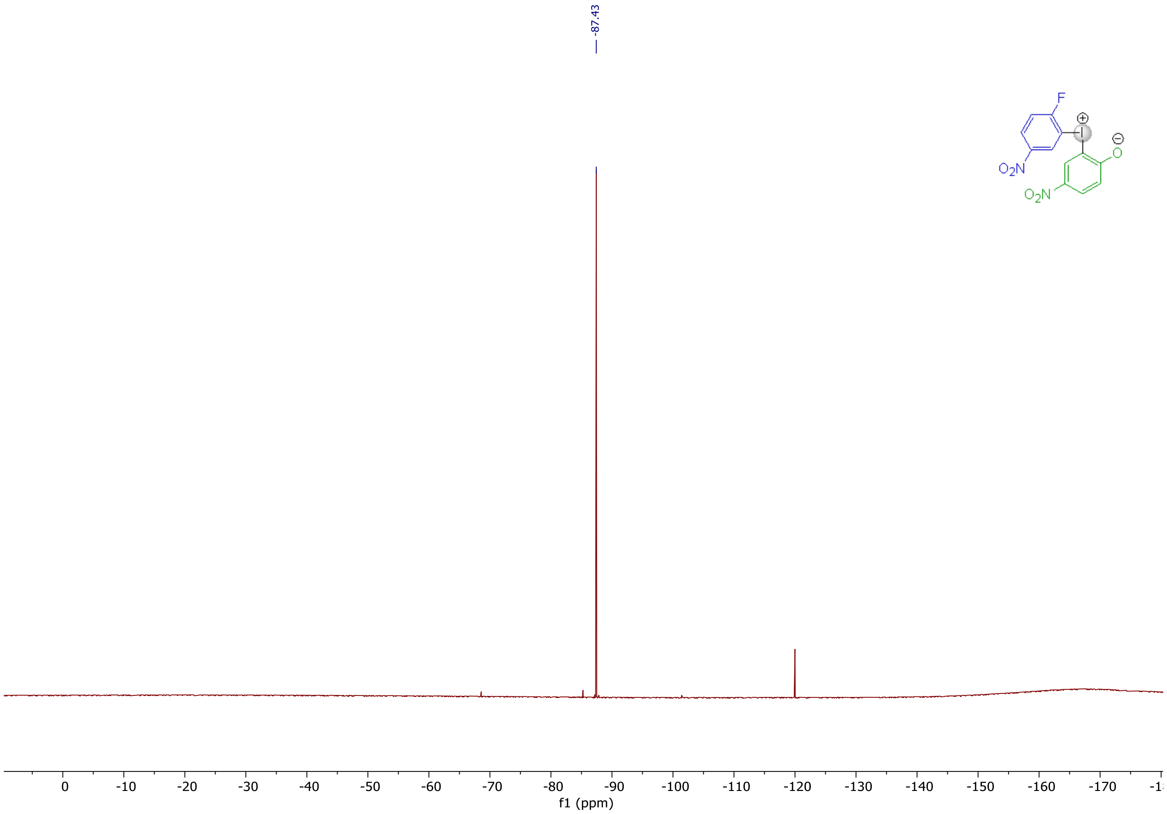

Supplement: Supplementary file 1 — au4c00500_si_001.pdf [file au4c00500_si_001.pdf]
